# Progress towards the 95–95–95 targets to end HIV by 2030 in Lebanon, 2023

**DOI:** 10.1371/journal.pone.0321868

**Published:** 2025-06-13

**Authors:** Mohammed Gouda ElMedrek, Joumana Hermez, Yvan Hutin, Abdinasir Abubakar, Ghada Muhjazi, Emmanuel Olatunji, Muhammad Shahid Jamil, Ahmed Sabry Alaama, Nevin Wilson, Firass Abiad, Hiam Yaacoub, Mostafa El Nakib

**Affiliations:** 1 World Health Organization (WHO), Regional Office for the Eastern Mediterranean Region, Cairo, Egypt; 2 WHO, Country Office for Lebanon, Beirut, Lebanon; 3 The Global Fund, Africa and Middle East (AME) Department, Grant Management Division, Geneva, Switzerland; 4 International Organization for Migration, Amman, Jordan; 5 Ministry of Public Health, Beirut, Lebanon; 6 National AIDS Programme Manager, NAP, Beirut, Lebanon; Saint-Joseph University of Beirut, LEBANON

## Abstract

**Background:**

Lebanon faces an HIV epidemic concentrated in key populations. The national AIDS programme [NAP] hosted by WHO since 1989 achieved substantial progress towards the 95-95-95 UNAIDS targets. In 2023, we reviewed the programme to guide its planned transition back into the structure of the Ministry of Health [MOH].

**Methods:**

In 2023, we reviewed programme documents, epidemiological information and interviewed relevant stakeholders. We compiled national data along with WHO and UNAIDS estimates to describe the evolution of programme and epidemiological indicators, along with the result chain of input, process, output, outcome and impact.

**Results:**

Domestic funding for the NAP increased from 73% in 2007 to 97% in 2018, before a drop in 2019 because of the financial crisis, when the NAP became dependent on international funding, including the Global Fund (commodities and services) and WHO (human resources). NAP core functions were governance, capacity building, monitoring and evaluation, anti-retro viral treatment [ART] dispensing and follow up for persons living with HIV [PLHIV] with some involvement in procurement, supply chain and laboratory testing. The NAP provided prevention, diagnosis and treatment services through Civil Society Organizations [CSOs]. In 2022, in Lebanon, 86% of PLHIV were diagnosed, among which 93% were on treatment and 95% virally suppressed. In 2022, NAP reported 232 new HIV infections, a 41% increase since 2010 and a 25% decrease in AIDS-related deaths during the same period. The estimated HIV incidence increased 4.4 times among MSM from 2008 to 2019, remained zero among commercial sex workers, and evolved from 0 to 0.11 per 1,000 to 0.9 per 1,000 in 2021 among PWIDs.

**Conclusions:**

Lebanon is on track to achieve the UNAIDS 95-95-95 by 2025 targets. After transition into the MoPH, the NAP will need to [1] identify ways to sustain its sources of domestic funding, [2] build on its collaborations with CSOs to expand prevention activities in key populations, and [3] address the evolving needs of the population, including among transgenders, migrants, displaced people, and refugees, 4) maintain good quality core functions (capacity building, monitoring and evaluation, and medications).

## Introduction

In 2020, UNAIDS released the 2025 95-95-95 targets; aiming for 95% of people living with HIV (PLHIV) to know their HIV status, 95% of those diagnosed to be on antiretroviral treatment (ART), and 95% of those on treatment to be virally suppressed.[1] In June 2021, United Nations Member states adopted these targets.[2] In 2022, UNAIDS estimated that, globally, there were 39 million PLHIV, 1.3 million people that became newly infected with HIV, and 630,000 people who died of AIDS. Prevalence is higher among key populations: 7.5% among men who have sex with men (MSM), 5.0% among people who inject drugs (PWIDS), and 10.3% among transgender persons.[3] In 2023, globally, 86% of PLHIV knew their HIV status. Of those, 89% were on treatment, and of those, 93% were virally suppressed.[3] Communities, especially civil society organizations (CSOs), have been the driving force for progress in HIV response.[4] For AIDS to be ended as a public health threat by 2030, CSOs need to get full support from governments and donors.[4]

In the WHO Eastern Mediterranean Region (EMR), the response to the HIV epidemic has not been that strong. In 2022, WHO/UNAIDS estimated that there were 490, 000 PLHIV. Of those, 38% were diagnosed, 27% of those diagnosed were receiving treatment, and 24% of those receiving treatment had viral load suppression.[5] The epidemic in EMR is concentrated in key populations, particularly in MSM, PWIDs, and commercial sex workers (CSWs).[6] Stigma and discrimination against key populations and PLHIVs in the region challenges behavioral research and CSO driven interventions.[7,8] This creates hidden populations that are difficult to reach and constitutes a major obstacle for the people most in need of prevention and treatment programmes to access support and care.[9]

In Lebanon, the national prevalence of HIV is low, but the burden is disproportionate in key populations. In 2019, WHO estimated that the DALYs attributed to HIV/AIDS in Lebanon was 2,684. [10] In 2022, UNAIDS estimated that the total number of PLHIV was 2,600 [Range: 2200–3100] in the country. The National Aids Programme (NAP) was established in 1989, fully funded by the Ministry of Public Health (MoPH), WHO and the Global Fund to Fight AIDS, Tuberculosis and Malaria (GFATM) and managed by WHO. In 1994, the Lebanese MOPH established a trust fund to finance the NAP that remained administered by WHO. Since this establishment, Lebanon faced challenges, including influx of refugees since 2011 (With nearly 900,000 registered Syrian refugees, another 500,000 informally settled and 174,000 Palestinians), an economic and political crisis in 2019, and a catastrophic blast in 2020. The economic crisis in 2019 made it impossible for the MOPH to continue financing the NAP through trust funds. As a result, WHO and GFATM provided temporary financial support to sustain the NAP operations and programme activities. In Lebanon, given the stigma and discrimination, the HIV response is based on collaborations with thematic non-governmental organizations (NGOs) who have a long history of working with key populations. Over the years, the NAP of Lebanon became one of the best success stories in EMR, because of [1] enabling policies, particularly for key and vulnerable populations, [2] commitment to resources from domestic financing, and [3] engagement of the civil society. As a result, Lebanon progressed well towards the 95-95-95 goals. In 2023, WHO and the MoPH of Lebanon agreed to transfer the NAP administration back to the MoPH structure as of 1 January 2024 in a way that would ensure ownership of the programme and protect and sustain achievements. At this important transition time, we documented achievements and challenges to make recommendations for sustainability of the NAP and provision of its services.

## Methods

### Annual reporting

We reviewed the UNAIDS Lebanon yearly country profiles from 2014–2022 that includes estimates of the number of PLHIV and AIDS-related deaths, notifications of new HIV infections, ART coverage, HIV prevalence among key populations, and progress against the 95-95-95 testing and treatment targets. We considered data from two processes; First, the Global AIDS Monitoring (GAM) mechanism compiled data on antiretroviral therapy, HIV-related behaviors, policies, expenditure data, and other indicators measuring progress toward global commitments. Second, WHO and UNAIDS generated HIV modeled estimates, including the numbers of new infections, PLHIV and AIDS-related deaths.[11] In Lebanon, sources of epidemiological data for HIV were mainly the HIV case notifications of the NAP and the results of the Integrated Bio-Behavioral Surveillance Study (IBBS) every 4–5 years.

### Programme review

From 29 November 2020 to 29 December 2020, WHO supported the NAP to conduct a remote programme review. Areas covered included HIV epidemiology and response in the context of the COVID19 situation, the national strategy, guidelines and policies, progress towards the global HIV targets, gaps and challenges, the funding landscape, and service delivery models.

### Review of documents

We conducted a review of available documents from 29 November 2020 to 29 December 2020. These included the National Strategic Plan (NSP) 2016–2020, the national HIV guidelines (2019), the test-treat-retain cascade analysis of 2019 (TTRC), the IBBS studies conducted in 2008, 2015 and 2019, the national sexually transmitted infections (STIs) assessment among MSM of 2019, the monitoring reports of the Global Fund funded Middle East Response (MER) projects, the national epidemiological reports, the pilot project for pre-exposure prophylaxis (PrEP) implementation from 2020, the WHO annual reports, administrative records, including budgets, and financial analysis from different sources including WHO, MER and UNAIDS.

### Field visits

We conducted field visits to institutions, facilities, health care providers and beneficiaries, NGOs, and other national partners.

### Interviews

We conducted interviews with policy makers, programme managers, representatives of affected populations, implementers and donors using an open-ended questionnaire addressing progress, challenges, and options to sustain the gains of the NAP. We also interviewed several stakeholders from different groups, including policy makers, CSOs/NGOs, and health government sectors including health system strengthening, maternal, newborn and child health, and harm reduction programme. We also interviewed officials from relevant non-health government sectors, key development partners, PLHIVs, and UN agencies.

### Integrated Bio-Behavioral Surveillance Studies

We obtained data from the reports of the last three IBBS done in Lebanon, 2008, 2015 and 2018 respectively.[12–14] In synthesis, these IBBS reports relied on a three pillars’ methodology, namely, a formative assessment, an IBBS, and a population size estimation. Furthermore, the IBBS relied on a mixture of methods to produce multiple estimates of the MSM and CSW population sizes in Lebanon as: 1) Literature Review; 2) Enumeration; 3) Unique Object Multiplier; and 4) Wisdom of Crowds. Additionally, this was followed by triangulation of these results to improve the accuracy of the final estimates of MSM and commercial sex workers.[13]

### Data analysis

**Fig 1:**
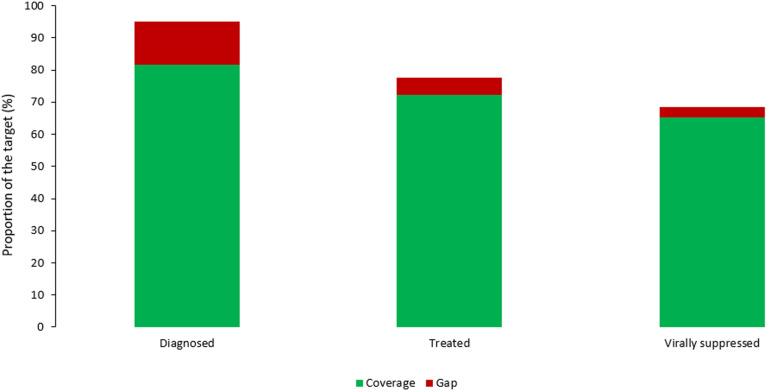
Proportion of HIV infected persons, diagnosed, treated, and virally suppressed in relation to the 95%, 95%, 95% targets, Lebanon, 2022 (Source: WHO and UNAIDS).

**Fig 2:**
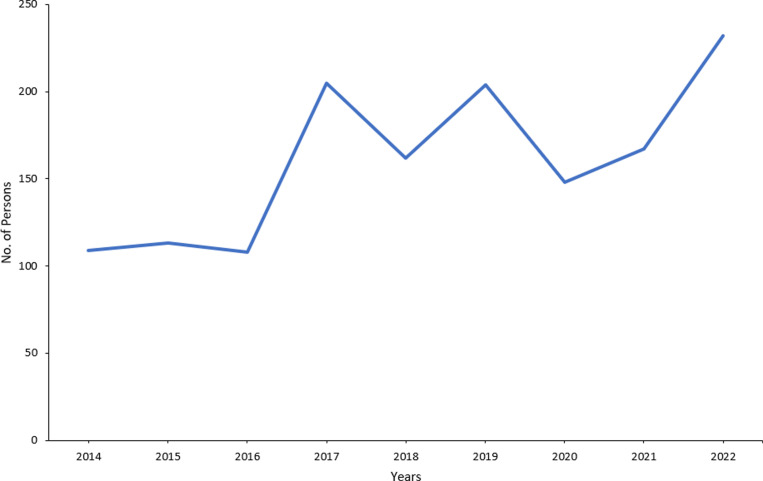
Newly annually reported cases of HIV infection, Lebanon, 2014–2022.

**Fig 3:**
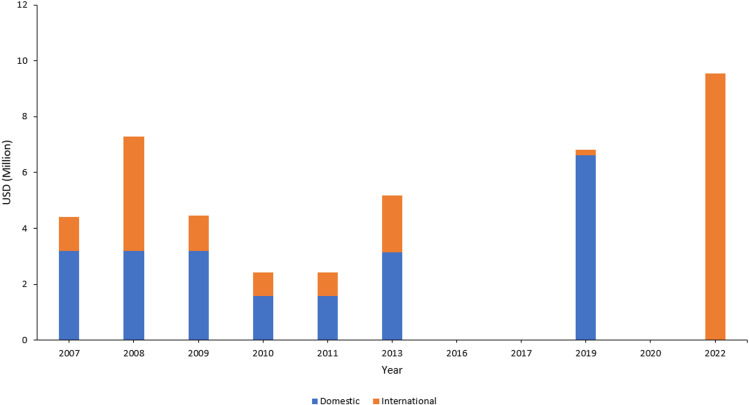
Expenditure of the NAP by financing source, Lebanon, 2007–2022 (Source: Global AIDS Mointoring, GARPR reports, July 2023, data not available for 2016, 2017, 2020).

We analyzed the programme data to describe the cascade of care, with estimates of those infected, diagnosed, on treatment and virally suppressed (Fig 1). We plotted the number of newly diagnosed HIV infections overtime (Fig 2). We plotted the expenditure of the programme between 2007 and 2022 according to the financing source, domestic or external (Fig 3).

We summarized estimates for the key HIV indicators according to the latest data available in Table 1.

**Table 1:** Key epidemiological indicators of the HIV epidemic, Lebanon, 2022 (Based on UNAIDS/WHO estimates).

Indicator	2022 estimates
*Prevalence of HIV in Adults [15–49) (%)*	<0.1 [<0.1–<0.1]
*Number of adults and children living with HIV*	2,600 [2200–3100]
*Number of new HIV infections*	<200 [<200–<500] (2021)
*Number of AIDS-related deaths*	<200 [<100–<200] (2021)
*Percent change in new HIV infections since 2010*	+41%
*Percent change in AIDS-related deaths since 2010*	−25%

We compared HIV incidence in MSMs, PWIDs and commercial sex workers according to the results of the IBBS of 2008, 2015 and 2019 Table 2.[12–14]

**Table 2:** HIV incidence in MSM, PWIDs and CSWs, Lebanon, IBBS 2008, 2015 and 2019.

Indicator	IBBS 2008	IBBS 2015	IBBS 2019	Comparison
HIV incidence among MSM (per 1000)	0.05	N/A	0.22	Increase 440% between 2008 and 2019
HIV incidence among commercial sex workers (per 1000)	0	0	0	No increase
HIV incidence among people who inject drugs (per 1000)	0	0.11	N/A	Increased 11% between 2008 and 2015
HIV incidence among other sub-populations (refugees, displaced, migrants, and transgender communities)	N/A	N/A	N/A	N/A

We obtained demographics on refugees and informally settled populations in Lebanon from the World Report of 2023 for Lebanon by the human rights watch organization. [15]

### Ethical statement

This is a retrospective study using the routine HIV data reported in the national AIDS programme review report, Lebanon’s HIV country profile by WHO and UNAIDS. No ethical approval was required. No data was collected from human participants by the author.

## Results

### Resources invested in the programme

#### Financing.

The NAP was able to mobilize domestic funding to cover 73% (3.2 USD million) of the needs in 2007 (4.4 USD million) up to 97% (6.6 USD million) of the needs (6.8 USD million) in 2019. Subsequently, domestic funding dropped, and the programme became fully dependent on international funding, including the Global Fund for commodities and services and WHO for human resources [16].

#### Personnel, planning and coordination.

The NAP key functions included governance, policy, coordination, strategic information, dispensing anti-retroviral medicines, training, procurement and supply chain, and laboratory investigations, including initial testing and viral load testing of patients on treatment. Overall, the NAP coordinated, and catalyzed activities implemented by other stakeholders, including the International Organization for Migration (that implemented the Global Fund grant in Lebanon) for procurement of commodities and services, CSOs for prevention, testing and ART dispensation. WHO hosted the NAP administratively and provided its core staff members with work contract. Those included a manager in charge of governance, policy, strategic information, and coordination, a pharmacist, dispensing anti-retroviral medicines, monitoring and evaluation officer, and an administrator.

#### Strategic information.

The NAP was responsible for collecting and analyzing data reported by providers, laboratories, and CSOs. Providers reported newly diagnosed cases to the NAP and filled out a paper application form to request ART. The medication distribution committee at the MoPH reviewed these applications to authorize treatment initiation. The NAP also established a reporting system by which NGOs reported epidemiological and programmatic data. The NAP implemented three rounds of IBBS in 2008, 2015 and 2019.[12–14] In 2019, population size estimates were generated for three key populations. These estimates suggested that there were 16,500 MSM (estimated HIV prevalence: 12%), 4,300 sex workers (prevalence of HIV unknown) and 3,100 people who inject drugs (PWID, estimated HIV prevalence: 0.9% in 2021). The NAP conducted TTRC analyses to identify gaps and missed opportunities to engage and retain PLHIV along the continuum of care.

### Service delivery

The NAP collaborated with various thematic NGOs and CSOs who had a long history of working with key populations including female sex workers, MSM and PWID. The role of contracted NGOs and CSOs included prevention through distribution of condoms, PrEP, testing on premises or through mobile clinics, case management of PLHIV, dispensation of ART, follow up with patients, hotline awareness, outreach and community engagement activities, implementation of IBBS, data collection and reporting. Overall, the NAP worked with around 20 NGOs. Among them, 16 were involved in prevention, testing, linkage and sometimes IBBS. Prevention was based on condoms, outreach activities, and educational sessions. In addition, in 2019, a pilot project for distribution of PrEP through these NGOs was started, serving 250 MSM. 13 NGOs were involved in voluntary counseling and testing (VCT) under contractual arrangements with the NAP, four followed up PLHIVs under treatment and dispensed ARTs. ARTs are dispensed through the NAP, directly to PLHIV (n = 1,500) or through NGOs which then dispense to PLHIVs in their catchment areas (n = 850). A contracted NGO, the Lebanese AIDS society, oversees PCR/viral load, laboratory supervision and technician training. Patients are also followed up on a yearly basis with new viral load tests to renew their prescription of medications that are then dispensed.

### Outcome indicators

#### Prevention.

In 2008, 2015 and 2019 the IBBS estimated that condom use frequency among MSM during the last anal sexual intercourse were <50%, 65%, and 62% respectively. By 2020, There were 360 on PrEP among all MSM.

#### Cascade of care.

In 2022, the Lebanon HIV cascade of care achieved diagnosis of 86% of PLHIV, among which 93% were treated and 95% were virally suppressed. Among key population (MSM), the proportion of people tested and know their HIV status increased from 11% in 2008 to 76% in 2019 according to the respective IBBS. The same comparison could not be done in the same way for commercial sex workers. The coverage of testing among commercial sex workers within 2019 was 59%. National coverage of testing was not disaggregated by vulnerable populations, including refugees, migrants and displaced communities.

### Impact

In 2022, NAP reported 232 new HIV infections, a 41% increase in the reported number of new HIV infections since 2010. During the same period, there was a 25% decrease in AIDS-related deaths. The incidence of HIV among MSM increased 4.4 times from 2008 to 2019, whereas the incidence among commercial sex workers remained at zero. The incidence among PWIDs increased from 0 to 0.11 per 1,000, between 2008 and 2015 then increased to 0.9 per 1,000 in 2021.The prevalence of HIV was unknown among the estimated 4,300 sex workers (2021 WHO estimate) and in the other vulnerable sub-populations.[17]

## Discussion

Lebanon progressed well towards the UNAIDS 95-95-95 goals, with 86% PLHIV diagnosed, 93% of those diagnosed treated and 95% of those treated virally suppressed according to 2022 estimates. In contrast, in the EMR, in 2022, only 38% of PLHIV were diagnosed, 27% of diagnosed were treated, and 24% of treated had viral load suppression. Lebanon’s cascade of care is the best performing in EMR with the highest proportion of PLHIVs diagnosed.[6] However, this progress faces challenges. First, the coverage of services is limited and centralized in central Lebanon (i.e., Beirut, Mount Lebanon), where most of the NGOs providing treatment and testing services are located. In contrast, the Bekaa, North and South areas are less covered by treatment services, physicians and NGOs. Second, HIV treatment services beyond ART are paid out of pocket. [17]. These include supporting laboratory tests (e.g., viral load and CD4 count), visit to infectious disease physicians, and care and treatment for opportunistic infections. The cost of these services is expensive in Lebanon, constituting economic obstacles to access for key populations. Third, the bottleneck of domestic funding threatens the sustainability of NAP services. The NAP suffered from a funding gap following the 2019 economic crisis where the programme became fully dependent on international funding. The Global Fund assisted with commodities and services and the investment in health system strengthening leveraging from dual stream of funding WHO helped with human resources in 2020–2023. However, to ensure sustainability and ownership, WHO planned to discontinue this funding after 2023 but to continue its technical advice and support. Overall, with this assistance in 2020–2023, the NAP managed to maintain services without stock-out. However, financial sustainability for the ARTs and human resources remained an issue.

In Lebanon, HIV infection is concentrated in key populations, including MSM, PWIDs, and female sex workers. Those are the focus of stigma and discrimination. Social rejection of key populations obstructs implementation of prevention, testing and treatment services. Our review indicated however that the engagement of CSOs allowed effective prevention, testing and treatment service delivery in these groups. Those have a long history of engaging key population in this complex context as well as community-based and community-led services are more effective and acceptable.[4] They can use rapid diagnostic tests that can provide same day results for people who test negative.[17] As a result, in Lebanon, there are opportunities to implement more activities in key populations than in most countries of the EMR.[17] Lebanon was able to implement a pilot project for PrEP that would benefit from a scale up given the role of MSM in the epidemic. However, stigma and discrimination remain and are more pronounced in governorates outside central Lebanon (i.e., Beirut and Mount Lebanon) that are already underserved by prevention, testing and treatment activities. The Lebanese penal code also prohibits sexual relations “contradicting the laws of nature”, which are punishable by up to a year in prison.[18] These laws are obstacles that prevent access to services and could evolve with the political environment. Hence, the status quo could evolve at any time, including more restrictions.

In Lebanon, the PLHIV who know their HIV status are not disaggregated by refugees, migrants, and displaced communities’ status. Moreover, according to the United Nations High Commissioner for Refugees (UNHCR), Lebanon remains the country hosting the largest number of refugees per capita worldwide. The government estimates that in 2022, there were 1.5 million Syrian refugees and 13,715 refugees of other nationalities in the country. The Bekaa region hosts the highest concentration of refugees.[19] Furthermore, the International Organization for Migration (IOM) also estimated that in 2023, Lebanon was hosting 160,738 migrants.[20] At the end of 2023, the United Nations Office for the Coordination of Humanitarian Affairs (OCHA) estimated that 55,183 individuals, among which 52% of females, had been internally displaced from south Lebanon due to ongoing hostilities.[21] The response to the HIV epidemic in Lebanon struggles to catch up with these recent demographic changes where the situation of HIV in these populations have not been described. Additionally, no needs assessment was conducted to identify comprehensive HIV prevention, testing and treatment packages. However, the presence of most of these people outside of central Lebanon limits their access to HIV prevention, testing and treatment services, as these locations are less served by physicians and CSOs. In such settings, HIV self-testing would have a potential to improve access for some populations while creating demand for more services.

Our review suffered from a main limitation where the modeled estimates of mortality and incidence were higher than what the service coverage could lead to expect. For instance, given the coverage of services and the status of the cascade, one would expect a decreasing incidence and lower mortality. This could be secondary to an inaccurate parameter estimate in the modelling used. Additionally, population size estimates by IBBS generally cannot estimate the true MSM and CSWs proportions who are hidden and/or who do not even acknowledge that they are MSM or CSWs. Furthermore, these IBBS fail to capture PLHIV who obtain their ARTs through private clinics and pharmacies, as well as MSM and CSWs under 18 years of age as they were limited to key populations above 18 years, which suggests a strong possibility that there is an underestimation of the true MSM and CSWs population size in Lebanon. Alternatively, high risk behaviors could remain common, pointing to the need to expand prevention activities, including pre-exposure prophylaxis. Other countries that have reached 90-90-90 coverage have also faced sustained incidence [22]. Unfortunately, available mortality data from civil registrations are not reliable enough to validate the estimates as stigma continues to prevent the mention of AIDS or HIV infection on death certificates. Further, increase in HIV notifications could reflect better diagnosis efforts. Finally, additional efforts need to continue to reconcile modeled estimates and reported data to understand the actual impact of prevention, testing and treatment efforts on incidence and mortality.

## Conclusions

Overall, we can draw several conclusions from this review. First, Lebanon is on track to achieve the UNAIDS 95-95-95 targets, although there are challenges with under-reporting, missing data, including sustainable financing. Second, the NAP is one of the unique success models in EMR by the way it is engaging and gaining the trust of key populations through collaborations with CSOs and NGOs. Third, the demographics of the beneficiary populations are evolving given the crises of refugees and displacement. Furthermore, the emergence of new groups may have led to the evolution of key populations that need to be addressed. In the light of these conclusions, we propose a number of ways forward. First, the funding for the HIV response needs to be sustained primarily through domestic funding, even if there will be external assistance in the short term. Second, we need to build on our successes and trust with key populations, especially MSM, to scale up prevention and adapt programmatic support through CSOs. This should include a scaling up of the PrEP to secure longer-term reduction in incidence. Third, the NAP needs to address the uncharted newly introduced key populations, including transgenders, migrants, displaced people, refugees and PWID/People who use drugs (PWUD) especially among migrants. Fourth, to enhance the under-reporting of deaths due to AIDS or HIV infection on death certificates by further outreach programmes tackling this stigma in the community. Fifth, to make sure to maintain good quality core functions such as capacity building, monitoring and evaluation, and medications.

## Supporting information

S1 File Expenditure_UNAIDS_2007–2022. **Lebanon HIV Expenditure by UNAIDS, 2007–2022**.
(PDF)

S2 File Programme review_Final Report_202. **HIV Programme review in Lebanon, Final Report, 2021**.
(PDF)

S3 File IBBS 2018 final report. **Lebanon HIV IBBS 2018 final report**.
(PDF)

S4 File IBBS 2015 final report. **Lebanon HIV IBBS 2015 final report**.
(PDF)

S5 File IBBS 2008 final report. **Lebanon HIV IBBS 2008 final report**.
(PDF)

S6 File Lebanon fact sheet UNAIDS. **Lebanon HIV country fact sheet by UNAIDS 2022**.
(PDF)

## HIV programme review in Lebanon final report team:

Dr Ismaël Maatouk (WHO national consultant), Dr Hamida Khattabi (UNAIDS contractual partner). Dr Nada Najem, Migration Health Physician (TB/HIV), IOM Country Office for technical contribution.

## IBBS 2015 final report team:

Robert Heimer, PhD, Professor, Kaveh Khoshnood, PhD, Associate Professor, Forrest Crawford, PhD, Assistant Professor, Fatma M. Shebl, MD, PhD, Assistant Professor, Russell Barbour, PhD, Assoc. Research Scientist, Danielle Khouri, MPH, Jacques Mokhbat, MD, Professor

## IBBS team 2008:

**Consultants:** Dr. Francisca Ayo Akala (Senior Public Health Specialist WB), Dr. David Wilson (Lead Health Specialist WB), Dr. Sarah Hersey (IBBS Specialist).

**Research team:** Dr. Kassem Kassak, Health Management and Policy Dept. (Team Leader); Dr. Jocelyn DeJong, Epidemiology and Population Health Dept. (AIDS expert); Dr. Ziyad Mahfoud, Epidemiology and Population Health Dept. (Biostatistician); Dr. Rima Afifi, Health Behavior and Education Dept. (Monitoring and Evaluation); Dr. Sawsan Abdurahim, Health Behavior and Education Dept. (Health Behavior); Dr. Sami Ramia, Medical Laboratory (Virologist and Medical Laboratory expert); Miss Farah El-Barbir (Research Assistant); Miss Maguy Ghanem (Research Assistant); Miss Sarah Shamra (Medical Laboratory Instructor); and, Mr. Khalil Kreidiyyeh (Medical Laboratory Technician).
