# Supplementary material for: Progress towards the 95–95–95 targets to end HIV by 2030 in Lebanon, 2023
Source: PLoS One. 2025 Jun 13;20(6):e0321868. doi: 10.1371/journal.pone.0321868 (PMC12165419; doi:10.1371/journal.pone.0321868)

HIV Expenditure

Total HIV Expenditure (country-reported)

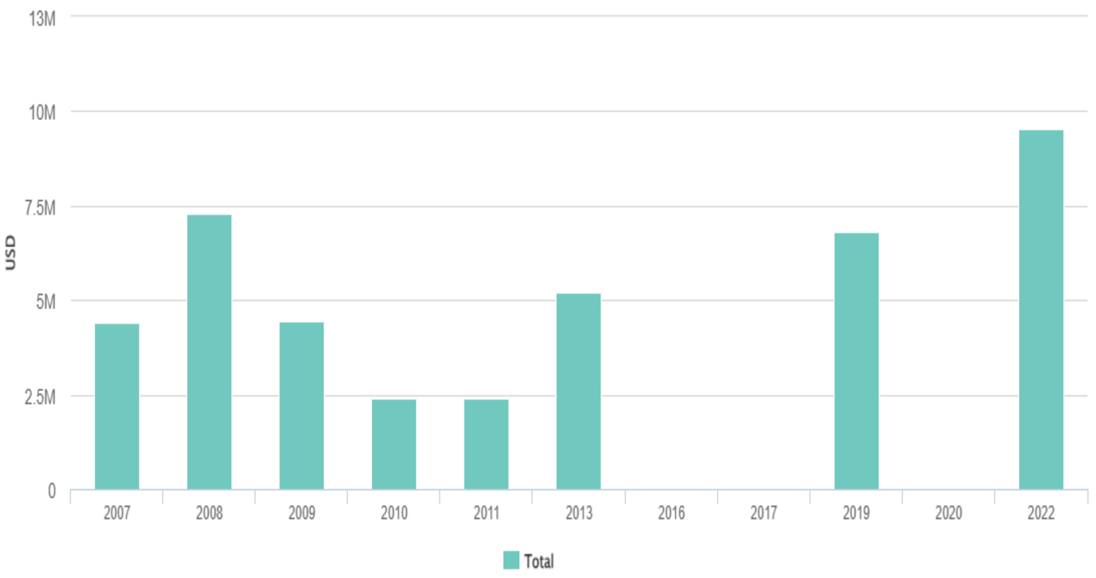

Country-reported HIV Expenditure by financing source

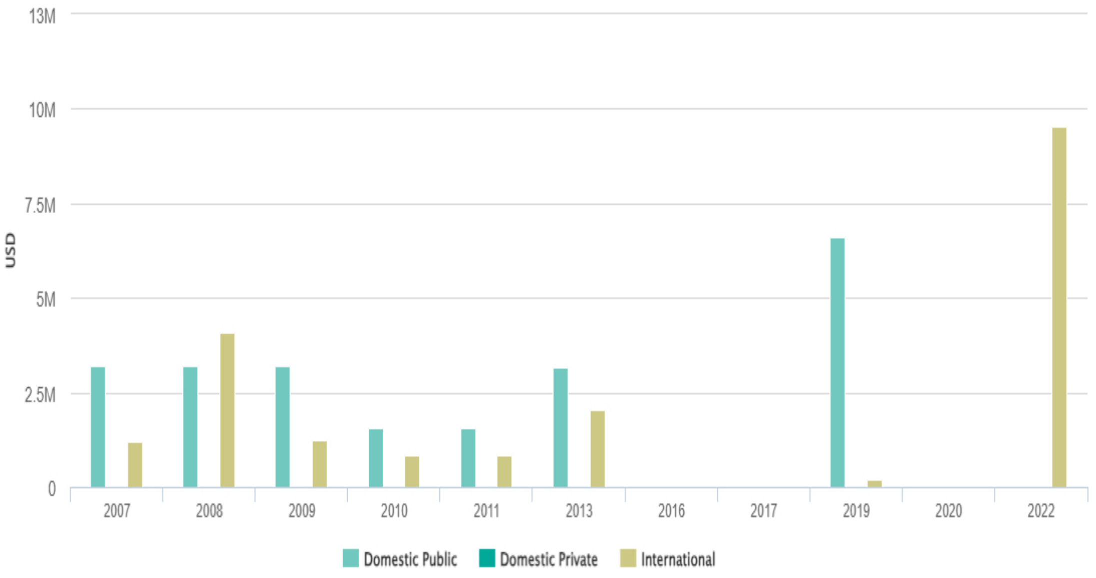

Supplement: S1 File — Lebanon HIV Expenditure by UNAIDS, 2007–2022. (PDF) [file pone.0321868.s001.pdf]
