# Supplementary material for: Progress towards the 95–95–95 targets to end HIV by 2030 in Lebanon, 2023
Source: PLoS One. 2025 Jun 13;20(6):e0321868. doi: 10.1371/journal.pone.0321868 (PMC12165419; doi:10.1371/journal.pone.0321868)
Supplement: S2 File — HIV Programme review in Lebanon, Final Report, 2021. (PDF) [file pone.0321868.s002.pdf]

Beirut, January 4th, 2021

HIV programme review in Lebanon

Final draft Report

Prepared by:

Dr Ismaël Maatouk (WHO national consultant)

Dr Hamida Khattabi (UNAIDS contractual partner)

Outline

ACKNOWLEDGMENTS 3

ABBREVIATIONS 4

LIST OF TABLES 5

LIST OF FIGURES 6

LIST OF ANNEXES 7

EXECUTIVE SUMMARY 8

1. INTRODUCTION 13

2. HIV PROGRAMME REVIEW IN LEBANON 14

2.1. RATIONALE 14

2.2. PURPOSE AND OBJECTIVES OF THE HIV PROGRAMME REVIEW 16

2.3 METHODS 17

STEPS OF THE HIV PROGRAMME REVIEW 18

DATA COLLECTION METHODS AND DATA SOURCES 18

LIMITATIONS OF THE ASSESSMENT 19

2.4. FINDINGS 20

2.4.1. OVERALL IMPACT OF THE PROGRAMME ON INCIDENCE AND MORTALITY 20

2.4.2. FINDINGS BY STRATEGIC DIRECTIONS OF THE NSP 2016-2020 20

2.4.2.1. SD1: STRENGTHENING THE AVAILABILITY AND USE OF STRATEGIC INFORMATION ON HIV 21

2.4.2.2. SD2: HIV PREVENTION AMONG KEY POPULATIONS, IN THE HEALTH SECTOR AND AMONG GENERAL POPULATION 22

2.4.2.3. SD3: TREATMENT, CARE AND SUPPORT FOR PERSONS LIVING WITH HIV 23

2.4.2.4. SD4: SUPPORTIVE SOCIAL, LEGAL AND POLICY ENVIRONMENTS 26

2.4.2.5. SD5: STRENGTHENING TECHNICAL, ORGANIZATIONAL, AND INSTITUTIONAL CAPACITY OF NAP AND NGOS 27

2.4.2.6. SD6: STRENGTHENING ACCESS TO KEY HIV SERVICES FOR INTERNALLY DISPLACED, MIGRANTS, REFUGEES AND MOST VULNERABLE HOSTING COMMUNITIES 28

2.4.3. COVID19, HIV SERVICES AND COPING STRATEGIES 29

2.4.4. KEY CHALLENGES AND OPPORTUNITIES FOR EFFECTIVE FUTURE HIV RESPONSE 30

3. KEY RECOMMENDATIONS 31

ANNEXES 35

INTRODUCTION AND BACKGROUND 35

2. Preparing for the programme review *36*

3. Data collection methods and data sources *37*

4. Collecting information *41*

4.1 DESK REVIEW AND ANALYSIS 41

4.2 FIELD REVIEW 42

A. Interviews *42*

B. Site visit *43*

Acknowledgments

The HIV programme review, from implementation to report of findings, has been conducted under the lead of the NAP manager Dr Mousatfa El Nakib and under the technical guidance of Joumana Hermez (Regional Advisor HIV, Hepatitis and STIs, WHO, EMRO) and Dr Elamin Eltayeb (Regional Programme adviser at UNAIDS Middle East).

The review could not be conducted without the valuable help of the technical working group established for this consultancy. The contribution of Dr Nada Najem, Migration Health Physician (TB/HIV), IOM Country Office, was particularly of great help.

Abbreviations

AIDS	Acquired immunodeficiency syndrome
ART	Antiretroviral therapy
COVID19	Coronavirus 19
EMR	Eastern Mediterranean Region
EMRO	Eastern Mediterranean Regional Office
HBV	Hepatitis B virus
HCV	Hepatitis C virus
HIV	Human immunodeficiency virus
HIVST	HIV self-testing
IBBS	Integrated Bio-Behavioral Surveillance Study
IOM	International Organization for Migration
KVPs	Key and vulnerable populations
M&E	Monitoring and evaluation
MENA	Middle East and North Africa
MER	Middle East Response
MOPH	Ministry of Public Health
MSM	Men who have sex with men
NAP	National AIDS Program
NGOs	Non-governmental organizations
NSP	National strategic plan
PEP	Post-exposure prophylaxis
PLHIV	People living with HIV
PrEP	Pre-exposure prophylaxis
PWIDs	People who inject drugs
SD	Strategic direction
STIs	Sexually transmitted infections
SWOT	Strengths, weaknesses, opportunities and threats
TB	Tuberculosis
TTRC	Test-treat-retain chain
TWG	Technical working group
UNAIDS	The Joint United Nations Programme on HIV/AIDS
VCT	Voluntary HIV counselling and testing
WHO	World Health Organization

List of tables

Table 1. Findings of HIV incidence among MSM, PWIDs and commercial sex workers from IBBS 2008, 2015 and 2019

Table 2. Condom usage rates during the last anal sex among MSM

Table 3. The 10 global core indicators for monitoring of national health sector responses to HIV identified in Lebanon

Table 4. The three 90s and MER-2 targets and achievements

List of figures

Figure 1. Trends of HIV by sexual orientation (2015-2020) as reported to the National AIDS Program.

Figure 2. The yearly new HIV cases with the cumulative cases as reported to the National AIDS Program.

Figure 3. Trends of HIV by age categories (2015-2020) as reported to the National AIDS Program.

Figure 4. New cases in 2020 by sexual orientation as reported to the National AIDS Program.

Figure 5. NGOs locations where testing and treatment services are available.

List of annexes

Annex 1: Assessment tool describing steps and tools to conducting programme review for the national response to HIV.

Annex 2: Technical working group members list.

Annex 3: Key informants list.

Annex 4: Table used for desk review information.

Annex 5: Questionnaire used for the key informants' interviews.

Annex 6: Minutes of the stakeholders meeting.

Executive summary

Great progress has been made in the global response to the AIDS epidemic. Unfortunately, many countries are still facing big challenges in achieving their programme targets towards ending AIDS by 2030. And these challenges are even more devastating in countries with humanitarian emergencies crisis. In our region, millions of people are affected by this crisis with numbers of refugees and displaced people increasing to unprecedented levels. The Middle East Response (MER) is the main supporter for essential TB, HIV and Malaria services in countries facing this crisis in our region. HIV programme review has been planned to be implemented in these countries generating findings to plan an HIV response tailored to the country context, progress to date and feasibility of scale up of high impact interventions. It also provides a good opportunity to optimize the resources from the current MER grant implementation for maximum impact, and allows to update the HIV response in the context of the new challenging pandemic of the coronavirus (COVID19).

This is particularly true for Lebanon where HIV response had major challenges such as the influx of refugees since 2011, the major economic and political crisis since 2019, a catastrophic blast in August 2020 on top of the COVID19 and the related healthcare system weaknesses.

In Lebanon, the HIV programme review has been implemented following the three main steps: i) desk review ii) field visits iii) key informants' interviews. A national technical working group has been established to coordinate the day-to-day implementation of the HIV programme review activities. A national stakeholders meeting has been conducted to discuss and endorse the draft findings and to agree on key priorities emerging from the HIV programme review findings.

The findings of the overall impact of the programme show that AIDS mortality, whether in the general population nor segregated by key populations, could not be determined. The incidence of HIV among men who have sex with men (MSM) increased 4.4 times from 2008 to 2019 as shown in the IBBS findings, whereas the incidence among commercial sex workers remained zero. Unfortunately, there is a gap in the updated incidence of HIV among people who inject drugs (PWIDs). The latest incidence among PWIDs was 0.11 per 1000 in the IBBS of 2015, showing an increase of 11% between 2008 and 2015. Furthermore, HIV incidence in other sub-populations such as refugees, displaced and migrants, transgender communities could not be determined at the national data level.

The findings by strategic direction of the national strategic plan highlight the following:

SD1: Strengthening the availability and use of strategic information on HIV

In the framework of the MER grant, a reporting system has been significantly strengthened, and NGOs are increasingly committed to report to the NAP some epidemiologic and programmatic data. NAP could implement three rounds of IBBS. And could frequently implement the TTRC analysis to identify gaps and miss opportunities to engage and retain PLHIV along the continuum of care. However, there are still some missing data to measure the whole 10 core global indicators. Strengthening the current information system for tracking the testing and treatment of HIV patients and unifying the existing HIV monitoring and

evaluation (M&E) system will help NAP to measure the 10 core global indictors and monitor the national response from inputs to impact.

SD2: HIV prevention among key populations, in the health sector and among general population.
The last IBBS showed a decrease of condom usage among MSM between 2015 (65%) and 2019 (61.9%). In fact, there regions are underserved by prevention activities among key populations. The majority of NGOs who provide free of charge condoms are based only in Lebanon (Beirut/Mount Lebanon). On the other hand, the new high prices of condoms secondary to the economic crisis are a challenge for people who cannot have it from NGOs. The COVID19 worsened the access to condoms from NGOs. The lack of funding prevents the sustainable availability of pre-exposure prophylaxis (PrEP) and post-exposure prophylaxis (PEP) interventions. Moreover, outreach activities and educational sessions have been dramatically impacted by the 2020 situation (economic, political and security situations) and by COVID19 restrictions. MER-2 grant could not achieve its training targets for the same reasons.

SD3: Treatment, care and support for persons living with HIV
Major efforts have been made to extend HIV testing approaches and services within MER-1 and MER-2 in the framework of NSP implementation, the first 90 was exceeded with the national testing coverage reaching 94.7%. Moreover, testing coverage among MSM increased from 11% in 2008 to 75.8% in 2019. Nevertheless, national testing coverage is not disaggregated by vulnerable populations such as refugees, migrants and displaced communities. And three (Bekaa, South, North) regions out of five are lacking HIV testing services and the big load (8 NGOs out of 10) is located in Beirut and Mount Lebanon.
ART are fully covered by the MOPH and provided by the NAP with no stock-out reported during the last couple of years. MER-2 grant provided significative support to HIV treatment in Lebanon. The MER-2 yearly target (1,500 PLHIV on treatment) was exceeded (1,707) in 2019. However, the second (64.5%[1]) and third (59.3%[2]) 90s were not reached. And treatment coverage is not disaggregated by key and vulnerable populations. It is worth to mention that like testing services; most treatment services activities are centralized in Beirut/Mount Lebanon. Even in Beirut/Mount Lebanon, economic challenges exist among the HIV treatment services and constitute big obstacles to reaching the treatment targets. The current situation in the country and the increase of prices along with COVID19 restrictions worsened these challenges.

SD4: Supportive social, legal and policy environments.
In Lebanon, NGOs constitute a safe place for key populations who seek health services in general and HIV services specifically. However, the existence of a discriminatory law against key populations is a major obstacle to reach many of these communities.
Tension and competition between the NGOs and the lack of national coordination mechanisms where the transparency in planning and implementing HIV activities by NGOs is coordinated constitutes a major

[1] TTRC, 2019
[2] TTRC, 2019

challenge. The economic crisis affected all sectors including health field and the expanses of the tests among the communities. The COVID19 and subsequent restrictions have worsened this economic crisis.

SD5: Strengthening technical, organizational, and institutional capacity of NAP and NGOs

The NAP leads the collaboration and coordination with the private sector, NGOs, ministries, media, religious leaders, UN agencies and other key stockholders to improve the situation of PLHIV and to halt the spread of the epidemic. The NAP has gained an expertise in planning and coordinating the implementation of the national HIV response. Yet, the major challenge faced by the NAP consists in the shortage of continuous and stable funding which leads to difficulties in recruiting skilled human resources. On the other hand, the influx of refugees and the consequent re-prioritization of resources towards other health concerns among refugees had always negatively impacted the financial resources of the HIV response. The Global Fund through MER-1 and 2 is the main financial support of the programme and could help the NAP achieving some of its targets in the period 2017-2020, but unfortunately, the current unstable political/economic situation and the COVID19 constitute the main obstacles for the MER-2 achievements and efforts.

At the NGOs' level, there is a clear commitment towards the NSP 2016-2020 and MER-1 and 2 grants implementation. There is a strong partnership between HIV thematic NGOs, NAP and other UN agencies. However, many challenges are facing the work of NGOs. There is a lack of a strategic plan highlighting the vision, targets and main strategic directions of the NGOs. There is a serious financial gap which impacts the human resources within NGOs due to the absence of sustainability of the grants/projects.

SD6: Strengthening access to key HIV services for internally displaced, migrants, refugees and most vulnerable hosting communities.

The estimation of HIV burden among the vulnerable populations has not been defined. And the needs assessment of the affected group among these populations have not been conducted to identify comprehensive HIV prevention, testing and treatment packages for each of them. MER grant could be a good opportunity to improve the strategic information data and strengthening testing and treatment activities among these populations, but, MER-2 could not unfortunately implement all its activities because of the 2019 and 2020 challenges in Lebanon.

Based on the main identified gaps, challenges and opportunities in the HIV programme review implementation, key recommendations have been developed to prioritize key high-impact interventions for Lebanon in preparation for MER-3 funding request and the development of the next National Strategic Plan. These recommendations were discussed and endorsed by the stakeholders during the stakeholders' consultation on the review finding that occurred on the 28th of December.

Recommendation 1: Make use of the findings of the HIV programme review to develop a national strategic plan for the next 3 years (2021-2023).

Recommendation 2: Strengthening multisectoral coordination mechanism to ensure a place at the table for all relevant partners and users and to ensure equality in decision making for an effective, multisectoral response to HIV in Lebanon.

Recommendation 3: Strengthening strategic information among key and vulnerable populations (size, HIV incidence, testing and treatment coverage) to set national targets for scale-up of high impact interventions tailored to the specific Lebanese context to monitor progress in the implementation of the national HIV response.

Recommendation 4: Addressing the gap between PLHIV diagnosed, those on treatment, and those who are virally suppressed.

4.1. Decentralizing the HIV treatment interventions by strengthening the technical HIV treatment capacity of NGOs located in the underserved regions (3 out of 5 governorates) to improve access to HIV laboratory testing and treatment services for PLHIV.

4.2. Pushing toward the change of the current medical health insurance policies and guidelines to improve access to HIV laboratory testing and treatment services for PLHIV.

4.3. Make available treatment information mainly for the key population mostly affected by HIV in Lebanon (MSM).

Recommendation 5: Scaling-up condom use among MSM.

Recommendation 6: Strengthening PrEP implementation.

6.1. Assessing the pilot phase of PrEP implementation.

6.2. Organizing stakeholders' consultation to discuss the assessment findings including strength and weaknesses.

6.3. Planning for the extension of PrEP implementation based on the main outcome of the stakeholders' consultation.

Recommendation 7: Strengthening the technical HIV capacity of the existing network of NGOs directly working with migrants, displaced persons and refugees.

Recommendation 8: Mapping and assessing availability and distribution of prevention, care and treatment services among the vulnerable populations.

Recommendation 9: Conducting needs assessment to inform the planning and prioritization of high impact interventions among the vulnerable populations.

Recommendation 10: Adapting HIV services to the new emerging COVID19 context.

10.1. Scaling-up community testing campaigns and HIV self-testing approaches based on the experience of the previous phase of HIVST implementation.

10.2. Building upon the experiences of the 2 NGOs that already deliver ART to communities, to empower and strengthen other NGOs to deliver ART and thus, increase the number of PLHIV receiving their treatment in the COVID19 context.

Recommendation 11: Encouraging communities to strengthen and accelerate HIV response in the context of COVID19 to enable them to maintain and build on their existing roles in service provision for HIV.

11.1. Strengthening technical capacities of communities to enable them delivering online counselling and large-scale prevention campaigns grounded in human rights, through internet technology and social media platforms.

11.2. Supporting NGOs with financial resources to improve their social media platforms to implement these large campaigns as well as educational sessions.

Recommendation 12: Developing resource mobilization strategy to overcome the financial and human resources challenges for the national HIV response.

1. Introduction

Human immunodeficiency virus (HIV) and acquired immunodeficiency syndrome (AIDS) epidemic had achieved great progress in the global response and millions of lives and new infections have been saved by the scale-up of both testing and antiretroviral therapy (ART). However, these achievements have not been equally distributed among countries and many national HIV responses are still facing big challenges in countries in achieving their programme targets towards ending AIDS by 2030[3]. National responses are even more challenging in countries facing the humanitarian emergencies crisis where preventable diseases like HIV, tuberculosis (TB) and malaria are not prioritized.

In the Eastern Mediterranean Region (EMR) of the World Health Organization (WHO) or the Middle East and North Africa (MENA) of The Joint United Nations Programme on HIV/AIDS (UNAIDS), millions of people are affected by this humanitarian crisis with numbers of refugees and displaced people increasing to unprecedented levels. The Middle East Response (MER) under the partnership of the International Organizations for Migration (IOM), is the main supporter for essential TB, HIV and Malaria services in countries facing this crisis in our region: Yemen, Syria, Jordan, Lebanon and Iraq. The two grants of the initiative (MER-1 2017-2018 and MER-2 2019-2021) aimed at improving responsiveness to the HIV-TB-Malaria in the context of emergencies in the region. However, HIV response in these countries needs to be tailored to the country context, progress to date and feasibility of scale up of high impact interventions. And this needs to be guided by robust multi-country programme reviews. Thus, a programme review provides a good opportunity to optimize the resources from the current MER grant implementation for maximum impact, and allows to include updates of the HIV response in the context of the new challenging and serious pandemic of the coronavirus (COVID19). For all these reasons, the WHO, UNAIDS, and Global Fund previewed multi-country programme reviews for countries in preparation for MER-3. Three countries are prioritized for full programme reviews: Lebanon, Palestine and Yemen.

[3] Miles to go: closing gaps, breaking barriers, righting injustices, UNAIDS, 2018

2. HIV programme review in Lebanon

2.1. Rationale

Lebanon is a small country of 10,452 km^2; it extends 217 km from north to south and spans 80 km at its widest point. The country is bounded by the Syrian Arab Republic in both the north and east and by the occupied Palestinian territory in the south.

Similar to other countries in the MENA, the UNAIDS data of 2020 estimate that Lebanon has a low-prevalence of HIV in the general population (0.1%). However, the epidemic is concentrated among men who have sex with men (MSM) in Lebanon with a prevalence of 12% (IBBS 2019).

The analysis of epidemiologic data of HIV in Lebanon is mainly based on the HIV case notification of the National AIDS Program (NAP) and on the implementation of Integrated Bio-Behavioral Surveillance Study (IBBS) each 4-5 years.

In fact, HIV case notification has been improved during the implementation of the two MER grants during the last three years (Figure 1). The cumulative of notified cases by 2020[4] was 2718, among which 148 occurred in 2020 (Figure 2). The majority of the new cases in 2020 are between 31 to 50 years old (50% of new cases) and there is a trend of new cases occurring in the younger population (Figure 3). Moreover, the 2020 reported cases indicate that 92.6% of new cases were among MSM (Figure 4). The remaining reported cases (7.4%) occurred among heterosexuals. No new cases of HIV infection due to other modes of transmission (namely among injecting drug users, mother-to-child transmission and blood transfusion) were reported.

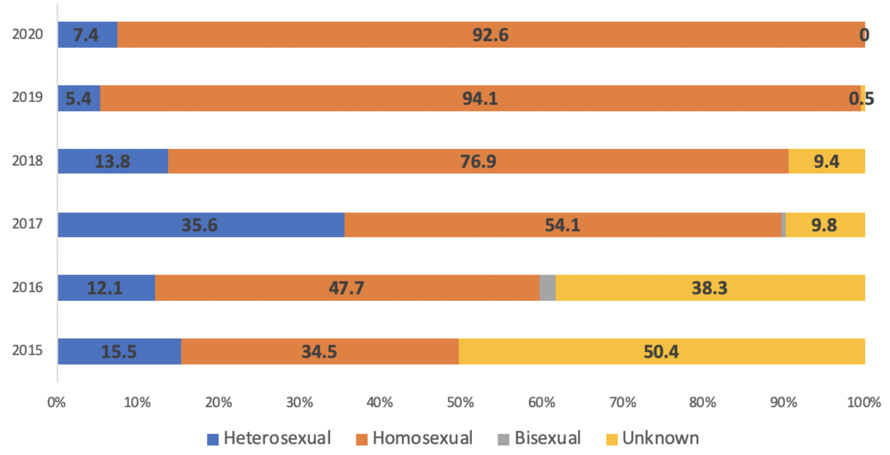

Figure 1. Trends of HIV by sexual orientation (2015-2020) as reported to the NAP.

[4] National AIDS Program report of HIV cases in 2020

The graph shows decreased trends in sexual orientation labelled as "unknown" and increased number of MSM among the yearly new HIV cases (source: World AIDS Conference, 2020).

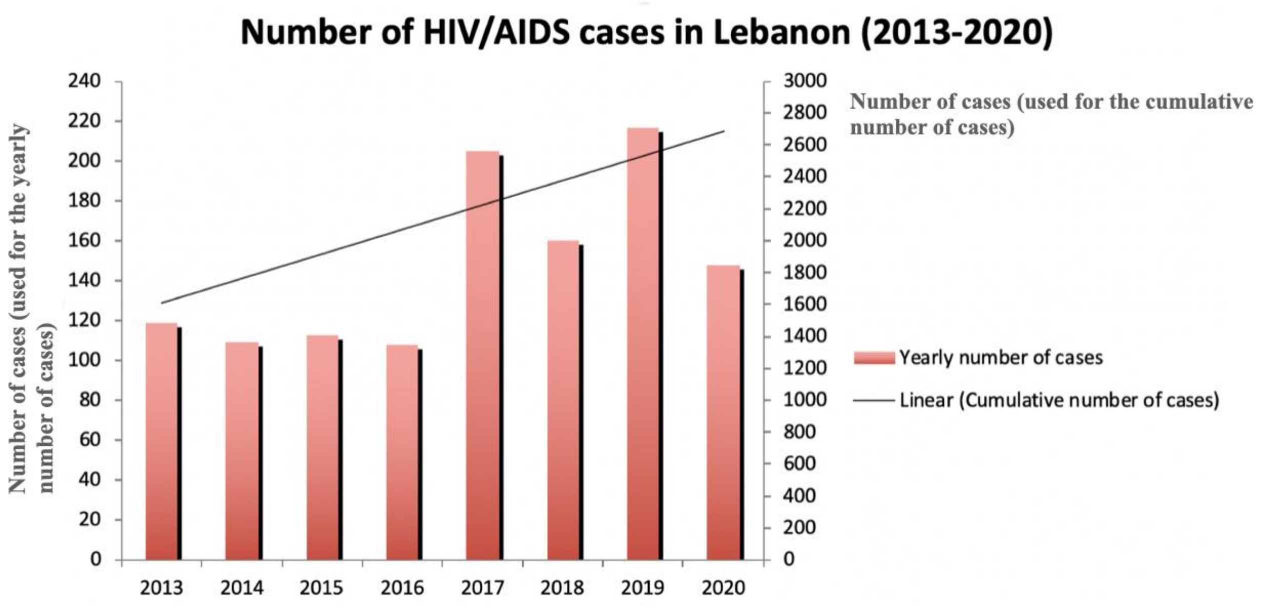

Figure 2. The yearly new HIV cases with the cumulative cases as reported to the NAP (source: World AIDS Conference, 2020).

number of cases (for the cumulative) and Number of cases (for the yearly cases)

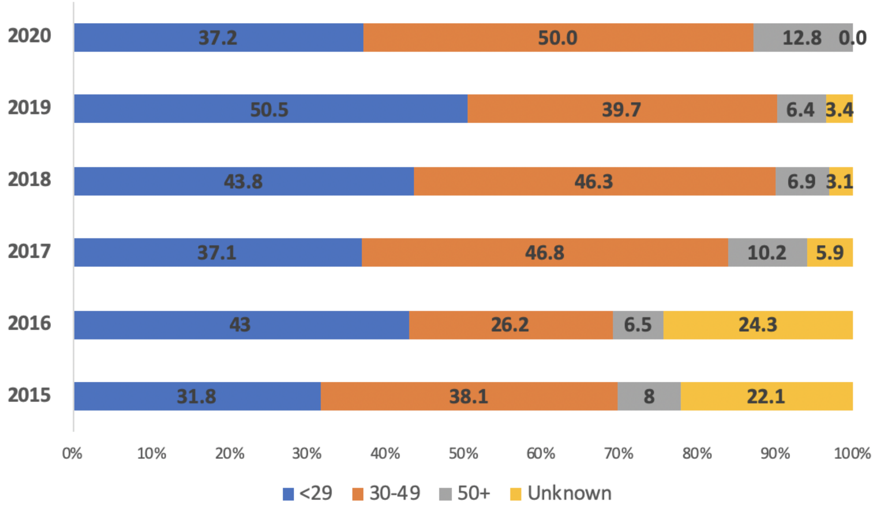

Figure 3. Trends of HIV by age categories (2015-2020) as reported to the NAP.

There is an increased number of new HIV infections among the young (<29 years) and middle population (30-50 years) (source: World AIDS Conference, 2020).

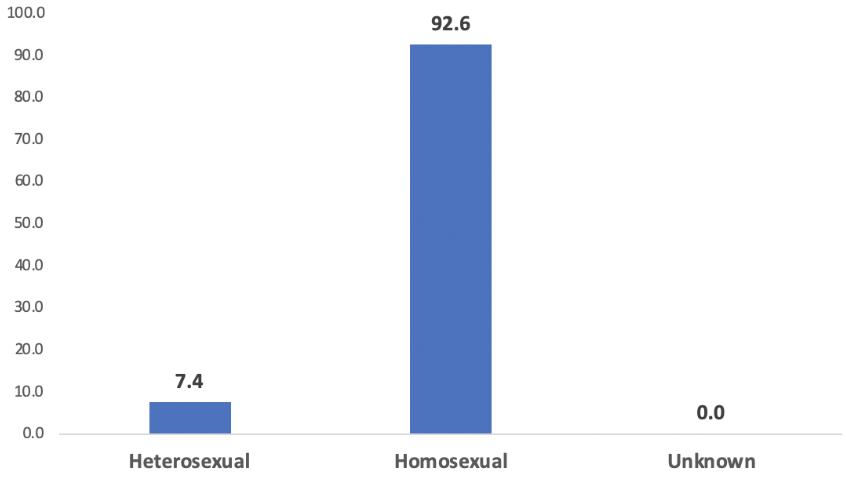

Figure 4. New cases in 2020 by sexual orientation as reported to the NAP.
92.6% of new HIV cases occurred among MSM (source: World AIDS Conference, 2020).

HIV response in Lebanon is based on collaborations between the NAP and various thematic non-governmental organizations (NGOs). In fact, the NAP has privileged a multi-sectorial approach in the national response as per the national strategic plan (NSP) 2016-2020 and has privileged also collaborations between the NAP and various thematic NGOs, academia, UN agencies and other stakeholders.
The Global Fund through the MER initiative provided the main support to the implementation of this NSP. MER initiative started in January 2017. It consisted of MER-1 grant (2017 and 2018) and MER-2 grant (2019 and 2020).

In all these contexts, the current programme review provides a good opportunity to optimize the resources from the ongoing MER grant implementation for maximum impact, for the coming three years and overviews the newly emerged challenges. It will provide priority interventions for the MER-3 grant of the Global Fund and will also inform for the next three years of the new NSP since the current one (NSP 2016-2020) comes to an end.

2.2. Purpose and objectives of the HIV programme review

In the framework of the MER initiative, an HIV programme review is planned to be implemented in the priority countries of the MER to provide a good opportunity to optimize the resources from the current

MER grant implementation for maximum impact. Thus, the main purpose of this consultancy was to conduct a country-specific HIV programme review for Lebanon in preparation for MER-3 funding request. The objectives were to:

- Review the HIV epidemiological and response with the context of the current emergencies including COVID19 situation (context review);
- Review the technical review panel comments/recommendations on the ongoing grant and propose best approaches for enhanced implementation with the context of each country;
- Review the existing national strategy, guidelines and policies, discuss their relevance to the global HIV targets and national context, and identify gaps and challenges (input review);
- Review the funding landscape and identify gaps (input review);
- Discuss service delivery models and packages of services (input review);
- Review progress to date in implementation, identify gaps and challenges (output/outcome review);
- Analyze the situation and response, identify gaps, strengths, weaknesses, opportunities and threats (SWOT) in terms of optimizing and scaling up the HIV response;
- Develop recommendations and a roadmap for addressing the gaps and scaling up the response including key strategic priorities to be included in the new funding request.

2.3 Methods

The evaluation builds upon a desk review and a country mission which took place from the 29 November 2020 to 29 December 2020. The preparation phase included a desk review and an analysis of available documents (WHO guidelines, national policy/strategy/plans, clinical guidelines, publications, reports, etc.), as well as a review of the NSP on HIV and other relevant documents.

During the mission, the consultant visited relevant institutions and facilities and liaised with key informants: policy makers, health care providers and beneficiaries, NGOs, other national partners where appropriate. The consultant participated in a national stakeholders meeting to discuss the draft HIV programme review and provide recommendations.

An assessment tool has been developed by the international consultant (Dr Hamida Khattabi) to conduct the HIV programme review in Lebanon, Palestine, and Yemen. A document describing steps and tools to conducting programme review for the national response to HIV was prepared and disseminated to the countries' consultants (Annex 1). It has been developed based on the terms of reference of the review and it uses mostly the WHO guide to conducting reviews for the health sector response and the WHO/EMRO HIV test-treat-retain cascade analysis, guide and tool. It also used other tools on specific aspects of HIV

strategic and operational planning, programme review, consolidated strategic information guide developed by WHO, UNAIDS and other partners.

The overall process was under the leadership of the NAP manager Dr Moustafa El Nakib. Based on the guidance of the international consultant, the NAP established the national technical working group (TWG). The TWG coordinated the day-to-day activities for the implementation of the programme review throughout the entire process. TWG list is attached in Annex 2 and consisted of the NAP manager, the surveillance of MER-2 activities focal point in the NAP, the national consultant, the international consultant, and IOM MER-2 technical officer. The TWG prepared the background documents, obtained relevant information for desk review, prepared a list of key informants (Annex 3), provided technical support to the organization of the national stakeholders meeting, discussed the review findings and identified the priorities for the future MER grant, and provided inputs on the draft recommendations of the national stakeholders meeting.

In the interest of fostering common ownership and shared responsibility and accountability, the NAP worked with major stakeholders in carrying out the review. A stakeholders' consultation was organized when the draft findings of the review was finalized. During this meeting, the primarily findings of the review were presented, discussed, completed, and endorsed by the stakeholders. Participants also identified main priority actions for the future MER grant based on the main findings of the review.

Steps of the HIV programme review

Three steps were identified by the international consultant and disseminated to the national consultants:

(1) Desk review of available documents relevant to HIV programme review;
(2) Field review involving a range of activities including technical briefing, stakeholder interviews;
(3) and site visits if needed and if possible (taking into consideration the unprecedented degrees of security and displacement challenges and on the other hand the COVID19 pandemic restrictions in Lebanon).

Data collection methods and data sources

Conducting programme review is based on the 5 questions that can guide the interpretation of programme performance through the review process (Are the right things being done?; Are they being done in the right way?; Are they being done on a large enough scale?; Are the right people being reached?; Is the programme making a difference?). These questions relate directly to the main information domains (inputs, process, outputs, outcomes, and impact). It assists in looking for and organizing the information that is required for the review. Annex 4 indicates the type of information required by basic review question and by information domain, it indicates the possible sources and compiles a list of required documents per type of required

information. All these questions were in line with the 10 global core indicators for monitoring of national health sector responses to HIV.

The desk review was conducted in coordination with all members of the TWG and a list of relevant documents was identified. These documents included the NSP 2016-2020, the National HIV guidelines 2019, the test-treat-retain cascade analysis in 2019 (TTRC), the IBBS studies conducted in 2008, 2015 and 2019, the national sexually transmitted infections (STIs) assessment among men who have sex with men (MSM) in 2019, the MER-1 and 2 monitoring reports, the national epidemiological reports, the pilot project for pre-exposure prophylaxis (PrEP) implementation in 2020, the WHO annual reports and other administrative records (budgets, administrative reports, …). Electronic copies of all these documents are available for any potential review.

Interviews provided qualitative information. They were conducted with a list of key informants (Annex 3). A questionnaire has been developed with the international consultant using standardized questions (Annex 5).

All findings were analysed with the international consultant and disseminated to the NAP manager. Following all these analyses, a stakeholders meeting was set remotely on December 28th 2020 from 10 AM to 12 PM. The national consultant used a power point document for the discussion and the endorsement of the key priority areas draft. The minutes of the meeting are provided in Annex 6.

Limitations of the assessment

Several limitations are identified while implementing the HIV programme review in Lebanon. The current analysis faced the lack of readily available data for some indicators. Accordingly, numerous assumptions were put in place to estimate where relevant indicators were not readily available. Moreover, one of the current analysis' limitation relates to those of the included reports where data is not available. Hence, the restrictions due to COVID19 could not allow numerous field visits or face-to-face interviews and imposed a remote meeting with the stakeholders which is a limitation of the normal long discussion between stakeholders.

2.4. Findings

2.4.1. Overall impact of the programme on incidence and mortality

AIDS mortality, whether in the general population nor segregated by key populations, could not be determined.

The incidence of HIV among MSM increased 4.4 times from 2008 to 2019 as shown in the IBBS findings, whereas the incidence among commercial sex workers remained zero. Unfortunately, there is a gap in the updated incidence of HIV among people who inject drugs (PWIDs). The latest incidence among PWIDs was 0.11 per 1000 in the IBBS of 2015, showing an increase of 11% between 2008 and 2015. Furthermore, HIV incidence in other sub-populations such as refugees, displaced and migrants, transgender communities could not be determined at the national data level. All these findings are summarized in **Table 1**.

Table 1. Findings of HIV incidence among MSM, PWIDs and commercial sex workers from IBBS 2008, 2015 and 2019

Indicator	IBBS 2008	IBBS 2015	IBBS 2019	Comparison
HIV incidence among MSM (per 1000)	0.05	N/A	0.22	Increase 440% between 2008 and 2019
HIV incidence among commercial sex workers (per 1000)	0	0	0	No increase
HIV incidence among people who inject drugs (per 1000)	0	0.11	N/A	Increase 11% between 2015 and 2008
HIV incidence among other sub-populations such as refugees, displaced and migrants, transgender communities	N/A	N/A	N/A	N/A

The years 2019 and 2020 were dramatically challenging for Lebanon and dramatically impacted the response as will be discussed through findings by strategic directions.

2.4.2. Findings by strategic directions of the NSP 2016-2020

The NSP 2016-2020 had set 6 strategic directions (SD) which are in line with the Global health sector strategy on HIV (2016-2021) of the WHO and with the 8-result areas of the UNAIDS strategy (2016–2021):

- SD1: Strengthening the availability and use of strategic information on HIV.
- SD2: HIV prevention among key populations, in the health sector and among general population.
- SD3: Treatment, care and support for persons living with HIV.
- SD4: Supportive social, legal and policy environments.
- SD5: Strengthening technical, organizational, and institutional capacity of NAP and NGOs.
- SD6: Strengthening access to key HIV services for internally displaced, migrants, refugees and most vulnerable hosting communities.

The HIV programme review findings are shaped by these strategic directions.

2.4.2.1. SD1: Strengthening the availability and use of strategic information on HIV

In Lebanon, the NAP is responsible for monitoring and collecting data reported by physicians, laboratories and NGOs. The physicians have to report diagnosed cases to the NAP for epidemiological purposes. They also have to fill an application form to request ART. This application is reviewed by the medication distribution committee at the Ministry of Public Health (MOPH).

However, health information system in general, and not only HIV, is suffering from fragmentation and under-reporting from different sectors[5]. Regarding the medication dispensing flow, the reporting system is running well. All patients are to submit yearly applications with requested viral load, and the medications are dispensed accordingly. However, the TTRC analysis conducted by the NAP pointed that “there is a weak information system for tracking the testing and treatment of HIV patients”. Many key informants expressed that “the existing HIV monitoring and evaluation (M&E) system is not unified, meaning that there is no systematic information sharing between the NAP, hospitals, labs and NGOs”.

Despite all these challenges, the NAP made huge efforts to generate data to measure some of the 10 global core indicators for monitoring of national health sector responses to HIV, which almost allows the NAP to implement the TTRC analysis. **Table 3** shows the main findings of the 10 global core indicators for monitoring of national health sector responses to HIV.

Despite the fact that the current surveillance system allows to generate data to measure some of the 10 global core HIV indicators, the current M&E system cannot generate data to measure the key strategic information among key and vulnerable populations. The HIV incidence among general, key and vulnerable populations and coverage of testing and treatment services among key and vulnerable populations are both not available.

[5] National strategic plan: page 28

Moreover, under MER-1 and 2 grants, surveillance visits from the NAP allowed the provision of technical assistance to improve HIV surveillance through continuous reporting (monthly) and through the already existing case reporting to ensure all cases are counted. These visits also strengthened data collection tool, data analysis and yearly dissemination for NGOs. These two facts strengthened the availability of other programmatic data that was of help for the NAP and NGOs. However, there is a major gap which is the absence of clear M&E frameworks at NGOs levels.

The recommended actions of the M&E system assessment should urgently be implemented to improve and strengthen the current M&E system.

2.4.2.2. SD2: HIV prevention among key populations, in the health sector and among general population

HIV prevention in Lebanon is based on condoms, prophylaxis, outreach activities and educational sessions. Condoms are distributed to the key populations for free through NGOs in partnership with the NAP who is providing condoms to all thematic NGOs. In the framework of the MER grant, a reporting system has been established where NGOs are reporting to the NAP the programmatic data related to condom distribution. However, this data is not disaggregated by key populations. Fortunately, Lebanon managed to conduct three rounds of IBBS in 2008, 2015 and 2019 which provided insight on condom use rates (in the last anal sex) among MSM. It has been noted that despite an improvement of condom use rates (in the last anal sex) among MSM between 2008 and 2015, there is a slight decrease of its usage between 2015 and 2019 (**Table 2**). In fact, the majority of NGOs who provide free of charge condoms are based in 2 out of 5 regions in Lebanon (Beirut/Mount Lebanon). More efforts should be done to assure condom is distributed in the 3 other regions (South, Bekaa, North) to improve the national condom use coverage. On the other hand, the new high prices of condoms secondary to the economic crisis are a challenge for people who cannot have it from NGOs.

Table 2. Condom usage rates during the last anal sex among MSM

**Findings from**	**Year**	**Condom use rate**
IBBS	2008	<50%
IBBS	2015	65%
IBBS	2019	61.9%
National STIs assessment	2019	52.2%

Other preventive tools such as pre-exposure prophylaxis (PrEP) and post-exposure prophylaxis (PEP) lack of sustainable funding.

Concerning outreach activities, two NGOs are actively reaching key populations in remote areas for the last 5 years. Unfortunately, these outreach activities have been dramatically impacted by the 2020 situation (economic, political and security situations) and by COVID19 restrictions. Moreover, maintaining outreach activities was possible but extremely difficult mainly for the key populations who would not go out during curfews or lockdown out of fear from governmental security officers and agents (PWIDs, transgender communities, migrants or refugees with illegal status, etc.). Neither the NSP 2016-2020 nor the MER grant had set specific targets regarding outreach activities among key populations.

However, educational sessions were successfully implemented by NGOs within the MER grants in the framework of the NSP implementation. In fact, with the MER-1 grant (2017-2018), there was an overall number of 280 healthcare workers in primary health care centers, sociocultural and NGO's health facilities who had received updated voluntary HIV counselling and testing (VCT) and HIV self-test (HIVST) training from the NAP. Participants were drawn from existing HIV thematic NGOs (contracted under MER-1); members of other United Nations agencies who are working with the WHO to reach MER objectives (UNHCR, IOM, UNRWA), members of the National TB program; and youth participants volunteering with the thematic NGOs. Trainings included updates on VCT, HIVST implementation and guidelines, assisted partner notification, and related technical guidance. With the MER-2 grant (2019-2020), 30 healthcare workers were trained in 2019 on HIVST, updates on VCT and partner notification. In 2020, classic trainings could not be executed due to the Lebanese situation and to the COVID19. To overcome these new challenges, some NGOs switched their trainings to the remote educational sessions. However, virtual or online sessions could not seem to be relevant or useful by the key informants interviewed.

2.4.2.3. SD3: Treatment, care and support for persons living with HIV

Major efforts have been made to extend HIV testing approaches and services (and ongoing) within MER-1 and MER-2 in the framework of NSP implementation. In fact, testing rate in the last year among MSM was 11% in 2008[6] and 75.8% in 2019[7] according to the respective IBBS. This was translated not only by achieving, but by exceeding the first 90 target. The latest TTRC 2019 showed that 94.7% of PLHIV were tested and know their HIV status (**Table 3**). Comparison of testing rate could not be done the same way for commercial sex workers. However, testing rate among commercial sex workers within the last year was 58.6% in 2019[8] and this rate can serve as a baseline data for evaluation of testing in this sub-population

[6] IBBS, 2008
[7] IBBS, 2019
[8] IBBS, 2019

in the next IBBS. Nevertheless, testing national coverage is not disaggregated by vulnerable populations such as refugees, migrants and displaced communities.

Table 3. The 10 global core indicators for monitoring of national health sector responses to HIV identified in Lebanon (highlighted are the three 90s).

10 global core indicators in Lebanon		Results
1	People living with HIV	2496 (in 2018)
2	Domestic finance	90% (shifted towards ART)
3	Prevention by key population	1. Condom use among MSM: 61.9% (IBBS 2019)<br>2. PrEP: not yet available; 1 ongoing pilot project (n=250 MSM)
4	HIV diagnosis (First 90)	2363; 94.7%
5	HIV care	1711; 68.5%
6	Currently on ART (Second 90)	1609; 64.5%
7	Currently retained and surviving on ART	not available
8	Viral suppression (Third 90)	1479; 59.3%
9	AIDS mortality	not available
10	New HIV infection	General population: 0.03%<br><br>0.05 per 1000 MSM (2008)<br>0.22 per 1000 MSM (2019)<br><br>0 per 1000 commercial sex workers (2008; 2015 and 2019)<br><br>0 per 1000 PWIDs (2008)<br>0.11 per 1000 PWIDs (2015)

Global Fund, through MER initiative, provided the main support for strengthening the HIV testing through the MER-1 (2017-2018) and MER-2 (2019-2021). A yearly target for testing (18,000 tests per year) has been set in the MER-2 grant (2019-2021) but has not been reached in 2019 (12,504/ 18,000 HIV tests (69.5%)) due to the political, social and economic situation in Lebanon (**Table 4**).

Table 4. MER-2 targets and achievements (highlighted in green are achieved).

Targets	Achievements
HIV testing targets: 18,000 per year (MER-2)	12,504 (69.5%) in 2019
Treatment targets: 1,500 PLHIV on treatment per year (MER-2)	1,707 (113.8%) at the end of 2019
Training targets: 80 healthcare workers trained per year (MER-2)	30 (37.5%) in 2019

The NAP has endorsed HIVST distribution among key and vulnerable populations at the beginning of COVID19's general lockdown (March 2020). Thus, HIVST which was implemented in 2018 within the MER-2 grant, has found a place in COVID19 times to maintain one of the HIV testing elements. Unfortunately, there was a resistance to its wide use at the beneficiaries and NGOs levels. Moreover, 3 regions out of 5 (Bekaa, South, North) are lacking HIV testing services and the big load (8 NGOs out of 10) is implementing HIV testing services in Beirut and Mount Lebanon, especially among key populations such as MSM and commercial sex workers.

Concerning care and treatment of PLHIV, ART are fully covered by the MOPH, and they are provided by the NAP at the ART dispensing center, with no stock-out reported during the last couple of years. Guidelines on therapy were updated[9] and included when to start ART, virologic assessment, prevention of mother to child transmission, what to start, drug formulary currently available in Lebanon, first-line regimen, alternative first-line regimens, dual therapy, contra-indicated regimens, response to therapy, pediatric management, therapeutic monitoring, ART failure and when to switch therapy, TB co-infection, PWIDs treatment, Hepatitis B and C co-infection, dosages of ART for adults and adolescents, management of ART toxicities and recommendations for drug resistance testing.

Lebanon has adopted the WHO guidelines focusing on medical management inclusively of all PLHIV, which includes an immediate, lifelong post-diagnosis treatment entitled "treat all". Moreover, there is a consistency between the NSP and the national HIV guidelines.
MER-2 grant provided significative support to HIV treatment in Lebanon. The MER-2 yearly target (1,500 PLHIV on treatment) was reached in 2019.

[9] National HIV guidelines 2019: pages 13-32

However, treatment of PLHIV faces many challenges. This is translated in the failure to reach the second (64.5%[10]) and third (59.3%[11]) 90s (**Table 3**). Hence, treatment national coverage is not disaggregated by key and vulnerable populations (it is not known if the right people are being reached). Among the challenges, centralization of HIV treatment services comes first. In fact, the three regions of Bekaa, North and South are heavily less covered by the treatment services by both physicians and NGOs. The majority of HIV cases are followed by around 10 specialists located in Beirut[12]. Moreover, most NGOs providing treatment services are located in Beirut and Mount Lebanon (8 NGOs out of 10), especially among key populations such as MSM and commercial sex workers (Figure 1). This is not the only challenge of PLHIV treatment. In fact, even in Beirut/Mount Lebanon, many economic obstacles exist among the available services. For instance, medical care for PLHIV is mainly delivered through the private sector. Viral load and CD4 tests for PLHIV that are requested on at least an annual frequency are expansive. Other services related to HIV are not covered. Co-morbidities of HIV are not covered neither. And finally, HIV treatment services: i) supporting laboratory tests, such as viral load and CD4 count, ii) seeing an Infectious disease physician, iii) opportunistic infections care and treatment, are paid out of pocket and not covered by any private or public health insurance while the cost of these services is very expansive in Lebanon.

2.4.2.4. SD4: Supportive social, legal and policy environments

Lebanon is known as a safer place for minorities in respect with their different lifestyles, believes and ideas. This is due to huge efforts made through the years by NGOs and activists to assure appropriate social, legal and policy environments in the country. Consequently, NGOs constitute a safe place for key populations who seek health services in general and HIV services specifically. They provide access and use of services that are free of stigmatization and discrimination. Moreover, there is a clear and strong participation of community actors in forums, guidelines and policies in Lebanon.

Nevertheless, many key community-level challenges and barriers were raised. The existence of a discriminatory law (article 534) against key populations is a major obstacle to reach many of these communities that remain vulnerable and hidden. The lack of legal protection of some key populations (PWIDs, PLHIV, MSM, etc.) affect the outreach activities and the access to care. Tension and competition between the NGOs and the lack of national coordination mechanisms where the transparency in planning and implementing HIV activities by NGOs is coordinated have also been mentioned as a major challenge. The economic crisis affected all sectors including health field and the expanses of the tests among the

[10] TTRC, 2019
[11] TTRC, 2019
[12] National strategic plan 2016-2020: page 27

communities. It also affected the key populations working in the artistic field or those who have freelance jobs who found themselves jobless or with a devaluated salary with the devaluation of the Lebanese currency. The COVID19 and subsequent restrictions have worsened this economic crisis.

2.4.2.5. SD5: Strengthening technical, organizational, and institutional capacity of NAP and NGOs

NAP

NAP, which is operating through a joint agreement between the MOPH and WHO, is in charge of every aspect of the HIV response in Lebanon. The activities of the NAP also include awareness about the disease and its management, fighting stigma and discrimination. Another major activity of the NAP is collaboration and coordination with the private sector, with NGOs, ministries, media, religious leaders, UN agencies and other key stockholders to improve the situation of PLHIV and to halt the spread of the epidemic. Partnerships and collaborative project with the NAP have become very frequent and have led to successful results and establishment of different projects on prevention, testing and stigma reduction. The NAP has gained an expertise in planning and coordinating the implementation of HIV activities. The current HIV programme review coincides with the last year of the 3rd NSP of the programme (the first NSP was launched in 2004; the second in 2009; the third in 2016).

Yet, the NAP suffers from shortage of continuous and stable funding which leads to difficulties in recruiting skilled human resources. Currently there are 3 staff including the NAP manager himself.

The Global Fund through MER-1 and 2 is the main financial support of the programme and could help the NAP achieving some of its targets in the period 2017-2020, but unfortunately, the current Lebanese situation and the COVID19 negatively impacted the MER-2 achievements and efforts. Moreover, series of financial challenges slowed the progress of the national response to HIV: the influx of refugees which had resulted in a general re-prioritization of resources towards other health concerns among refugees, the unstable political/economic situation, the unstable internal security of the country. On top of these, the new pandemic of COVID19 exacerbated these financial challenges.

NGOs

There is a clear commitment from the NGOs' side towards the NSP 2016-2020 and MER-1 and 2. In fact, a strong partnership exists between HIV thematic NGOs and other NGOs working remotely, and the NAP and other UN agencies, respectively. Moreover, the NGOs helped the NAP providing the treatment on its behalf following the Beirut blast of August 4th 2020 and the destruction of the NAP premises.

However, many challenges can be identified at the NGOs level. There is a lack of clear and bold targets related to HIV services and a lack of a strategic plan highlighting the vision, targets and main strategic directions of the NGOs. There is a serious financial gap which impacts the human resources within NGOs due to the absence of sustainability of the grants/projects. This induces a non-interest of volunteers and staff to remain at their temporary positions. Furthermore, there is a clear centralization of services, mainly in Beirut and Mount Lebanon. Despite some outreach activities, dramatically affected by COVID19, the 3 areas of Bekaa, South and North are not equally covered with Beirut and Mount Lebanon. Moreover, although the NAP already adopted the WHO recommendation of community ART provision, this could not lead to a decentralization of treatment and the main load of treatment is at the NAP. Hence, a mapping of community health and social support services has not been reported and only a list of referrals is being used if needed by each NGO.

The obstacles to accessing and using available services that were raised are mainly (1) financial challenges and lack of funding to have sustainable projects and services; (2) non-interest of volunteers and staff in contracts that are not secured or sustainable which consequently allows an easy quit of the job. Thus, finding a serious staff is difficult and leads to shortage of healthcare providers interested in HIV related issues.

2.4.2.6. SD6: Strengthening access to key HIV services for internally displaced, migrants, refugees and most vulnerable hosting communities

In Lebanon as well as in many neighboring countries, the presence of high numbers of refugees is alarming, especially that health, social and behavioral determinants create a favorable environment for transmission of disease like TB, STIs, HBV, HCV as well as HIV. Thus, these communities have been the priority group of the NSP 2016-2020. However, there is no testing and treatment coverage disaggregated by these populations. It is worth mentioning that the estimation of HIV burden among these populations has not been defined. And the needs assessment of the affected group among these populations have not been conducted to identify comprehensive HIV prevention, testing and treatment packages for each of them.

MER grant could be a good opportunity to improve the strategic information data and strengthening testing and treatment activities among these populations, but, MER-2 could not unfortunately implement all its activities because of the 2019 and 2020 challenges in Lebanon.

2.4.3. COVID19, HIV services and coping strategies

At the beginning of COVID19 cases (end of February), the government implemented a general lockdown in the country. The NGOs replaced their services in the field and on-site by a remote work along with implementation of hotlines and referrals of emergency cases to physicians. The NAP requested during this period to scale up HIVST which became almost the only possible testing service in the country. Moreover, the NAP requested that counsellors follow-up by phone calls with people who received HIVST on their usage and results. By the beginning of May, the general lockdown was relaxed and NGOs could resume their work. The NAP asked NGOs to resume delivering essential HIV services to key and vulnerable populations while prioritizing the safety of health-care workers and beneficiaries. With the direct support of the IOM through the MER-2, the NAP provided NGOs with personal protective equipment (PPEs), hang hygiene, respiratory hygiene, and general physical distancing guidelines. Through reprogramming efficiencies, WHO Lebanon and IOM were able to coordinate the successful procurement of essential PPEs to national programmes. Implementing NGO partners are continuously supplied with essential PPEs from this stock proportionally to the nature and volume of their activities. These activities were steadily returning to normal frequencies around July 2020, few weeks before the Beirut blast of August 4th 2020 which resulted in a second paralysis of HIV services. By July 2020, activities had ranged from on-site testing and counseling services in NGO premises to outreach testing, counseling, and awareness raising activities in refugee camps and other locations with populations identified to have a high risk of acquiring HIV. The availability of PPEs has reportedly improved the bilateral engagement of sexual health care providers and beneficiaries amid this overarching health crisis. WHO Lebanon and IOM have successfully coordinated the replenishment of this PPE stock to ensure continuation of the safe delivery of essential HIV services throughout this health crisis. It is true that COVID19 times have witnessed noticeable drops in HIV testing volume and a partial paralysis of HIV testing services, but efficient coordination and continued distribution of essential supplies (including PPEs) allowed many activities to slowly resume. The NAP closely monitored developments through regular assessment of gaps and challenges. It is worth mentioning that ART services were prioritized in the ART dispensing center at the NAP without any shortage as well as in two NGOs that helped the NAP and became ART dispensing centers with the COVID19 general lockdown and mainly after the Beirut blast of August 2020. One crosscutting challenge continuously reported long before the current crisis is the difficulty in access to key and vulnerable populations. In a context where stigma and discrimination continue to prevail, the COVID-19 health crisis has created more pockets and gaps in access to an already marginalized population. This is especially true for rural areas outside of Beirut where already underserved populations are feeling the added strain of a protracted economic and health crisis.

2.4.4. Key challenges and opportunities for effective future HIV response

2.4.4.1. Key challenges

The series of tragic events that happened during 2019 and 2020 dramatically impacted the HIV response in Lebanon. In fact, the national currency started a slight devaluation which gradually aggravated on a monthly basis to reach a loss of 80% of its value by November 2020. Moreover, bank restrictions, credit cards limitations, US dollars selling in the black market have all contributed to this economic crisis, never seen in the history of the country.

Since October 2019, the political scene has experienced a major crisis with series of strikes that were bloody at some points. The country could not see a government expected to implement strict and bold reform which should have helped the recovery from corruption and political misconduct.

A massive explosion occurred in the port of Beirut (August 4th, 2020) killing hundreds of citizens, injuring thousands and leaving hundreds of thousands homeless.

The COVID19 pandemic was accompanied by lockdown policies which are having instantaneous and dramatic effects on the already drained health system and on the daily economic activity.

All these challenges would make HIV testing and treatment the least priority for the Lebanese society, including KVPs. Moreover, the increase of prices which included condoms and lubes would allow the society and KVPs to undertake condomless behavior. Finally, as mental health outcomes are considered a challenge during pandemics, lockdowns, economic crisis, and political instability, risky behavior especially among KVPs would be expected as a negative coping strategy. From a programmatic angle, human and financial resources in the health system in general were all shifted towards COVID19 related challenges.

These challenges have made the situation of the hosting population in Lebanon more critical than the refugees, in general and in HIV services. For instance, refugees have fixed income from UN agencies and have full coverage under MER grant (coverage of ART and all HIV-related testing); whereas for the Lebanese populations, testing is not covered. This is a major change that happened in 2019-2020 and disbalanced the hosting-foreign equilibrium.

The 2019-2020 challenges were added to the already challenging factors that compromise HIV prevention, diagnosis and treatment services in Lebanon:

(1) Coexistence of two challenging populations: the vulnerable populations (refugees, displaced and migrants) and the hosting population (the latest crises in Lebanon made this group even more

vulnerable than the mentioned vulnerable populations), making the national HIV response more problematic;

(2) Lack of financial and human resources which constitutes a huge challenge for the NAP to lead the HIV planning and monitoring progress for the national HIV response;
(3) Lack of strategic information data disaggregated by key and vulnerable populations;
(4) Lack of coordination of the national HIV response planning and implementation between stakeholders including NGOs;
(5) Centralized services which constitutes main treatment challenges: strengthening the NGOs to implement treatment services in the three underserved regions (North, Bekaa, South) will significantly contribute to improve the national treatment coverage;
(6) Non health coverage of HIV services expanses by both the public and private insurances.

2.4.4.2. Key opportunities

- The existence of NGOs in the three under-served regions which are technically and administratively strong in implementing health interventions.

  Involving and strengthening HIV testing and treatment technical capacity of these NGOs will contribute largely in improving the testing and treatment coverage.

- The existence of a network of NGOs directly working with the communities of refugees, migrants and displaced along with the UNHCR and IOM support.

  Empowering the technical HIV capacity of these NGOs in regard with their geographical are of work will strengthen the HIV prevention packages among these populations.

- The willingness of donors to contribute technically and financially to improve the national HIV response.

  The development of the i) next NSP based on the key findings of the HIV programme review, setting national targets and prioritizing main high-impact interventions to reach these targets, and ii) M&E plan to monitor progress, should together be the basis of resource mobilization for national and international donors.

3. Key recommendations

The national HIV response in Lebanon has achieved significant progress in terms of HIV testing: coverage (exceeded) and availability of disaggregated testing coverage by the most key population group (MSM). Three rounds of IBBS (2008, 2015, 2019) as well as the TTRC analysis were implemented and findings have been used for re-planning activities during the implementation of the NSP 2016-2020. Moreover, the MER-1 and MER-2 grants largely contributed to the implementation of these activities. However, many challenges are identified and were worsened by the dramatic events of the last two years.
While efforts should still be made to maintain the testing coverage achievement, more work should be undertaken to strengthen the HIV continuum of care from prevention to viral suppression. Thus, the below recommendations are built upon the analyses of the main findings of the HIV programme review implementation in order to optimize and scale up the HIV response in Lebanon. These recommendations were discussed and endorsed by the stakeholders during the stakeholders' consultation on the review finding that occurred on the 28th of December.

Recommendation 1: Make use of the findings of the HIV programme review to develop: i) a national strategic plan for the next three years (2021-2023), ii) a costed operational plan for the NSP implementation and iii) a national M&E plan to monitor progress of the operational plan implementation.

Recommendation 2: Strengthening multisectoral coordination mechanism to ensure a place at the table for all relevant partners and users and to ensure equality in decision making for an effective, multisectoral response to HIV in Lebanon. This should be the core of the NSP development and implementation.

Recommendation 3: Strengthening strategic information among key and vulnerable populations (size, HIV incidence, testing and treatment coverage) to set national targets for scale-up of high impact interventions tailored to the specific Lebanese context as well as progress to date and to monitor progress in the implementation of the national HIV response. This should be the fundamental of the NSP development.

Recommendation 4: Addressing the gap between PLHIV diagnosed, those on treatment, and those who are virally suppressed.
4.1. Decentralizing the HIV treatment interventions by strengthening the technical HIV treatment capacity of NGOs located in the underserved regions (3 out of 5 governorates) to improve access to HIV laboratory testing and treatment services for PLHIV.
4.2. Pushing toward the change of the current medical health insurance policies and guidelines to improve access to HIV laboratory testing and treatment services for PLHIV.

4.3. Make available treatment information mainly for the key population mostly affected by HIV in Lebanon (MSM).

Recommendation 5: Scaling-up condom use among MSM.

Recommendation 6: Strengthening PrEP implementation.
6.1. Assessing the pilot phase of PrEP implementation.
6.2. Organizing stakeholders' consultation to discuss the assessment findings including strength and weaknesses.
6.3. Planning for the extension of PrEP implementation based on the main outcome of the stakeholders' consultation.

Recommendation 7: Strengthening the technical HIV capacity of the existing network of NGOs directly working with migrants, displaced persons and refugees.

Recommendation 8: Mapping and assessing availability and distribution of prevention, care and treatment services among the vulnerable populations.

Recommendation 9: Conducting needs assessment to inform the planning and prioritization of high impact interventions among the vulnerable populations.

Recommendation 10: Adapting HIV services to the new emerging COVID19 context.
10.1. Scaling-up community testing campaigns and HIV self-testing approaches based on the experience of the previous phase of HIVST implementation.
10.2. Building upon the experiences of the 2 NGOs that already deliver ART to communities, to empower and strengthen other NGOs to deliver ART and thus, increase the number of PLHIV receiving their treatment in the COVID19 context.

Recommendation 11: Encouraging communities to strengthen and accelerate HIV response in the context of COVID19 to enable them to maintain and build on their existing roles in service provision for HIV.
11.1. Strengthening technical capacities of communities to enable them delivering online counselling and large-scale prevention campaigns grounded in human rights, through internet technology and social media platforms.

11.2. Supporting NGOs with financial resources to improve their social media platforms to implement these large campaigns as well as educational sessions.

Recommendation 12: Developing resource mobilization strategy to overcome the financial and human resources challenges for the national HIV response.

Annexes

Annex 1: Assessment tool describing steps and tools to conducting programme review for the national response to HIV.

Assessment tool describing steps and tools to conducting programme review for the national response to HIV.

Introduction and background

The Global Fund is the main financial mechanism supporting the provision of essential HIV, TB and Malaria Services in countries affecting by Humanitarian crisis in MENA. The Middle East Response (MER) under the PRship of IOM, is the main supporter for essential TB, HIV and Malaria services in Yemen, Syria, Jordan, Lebanon and Iraq. The MER initiative uses one regional grant management platform, with the aim of improving responsiveness to the three diseases in the context of emergencies in these countries. UNAIDS and WHO are co-chairing the Technical Support Group that provides technical oversight and support to the MER grant implementation and funding request Within the context of the Middle East Response (MER) grant, the HIV response in the priority countries needs to be tailored to the country context, progress to date and feasibility of scale up of high impact interventions and services towards the goal of ending AIDS by 2030.

In the framework of the MER initiative, an HIV programme review is planned to be implemented in the priority countries of the MER to provide a good opportunity to optimize the resources from the current MER grant implementation for maximum impact.

An assessment tool has been developed to conduct the HIV programme review in Lebanon, Palestine, and Yemen. The document describes steps and tools to conducting programme review for the national response to HIV. It has been developed based on the terms of reference of the review and it uses mostly the WHO guide to conducting reviews for the health sector response and the WHO/EMRO HIV test-treat-retain cascade analysis, guide and tool as well as other tools, on specific aspects of HIV strategic and operational planning, programme review, consolidated strategic information guide developed by WHO, UNAIDS and other partners.

The assessment tool is intended to be used by and for individuals and entities involved in planning and managing the response to the HIV epidemic. It is primarily aimed at national level planning but can also be used for other levels such as regional/province or district. The audience includes Ministries of Health, other government sectors, nongovernmental organizations, private sector, academic institutions, and other civil society organizations.

2. Preparing for the programme review

Conducting a programme review for the national HIV response involves a wide range of actors, requires various types of skills, uses various types of information and requires significant amount of time and resources. The key to a successful review is often the work done before the review. Reviews that are not well planned are likely to run into numerous difficulties which could compromise the integrity of the findings.

A participatory process for conducting the review is required in order to achieve broad stakeholder engagement, consensus on the findings of the review and ownership of the conclusions and recommendations resulting from it.

The following considerations and steps have been planned to undertake the review:

2.1 Establishment of a regional technical working group.

This group is composed by UNAIDS/MENA/RST, WHO/EMRO, IOM and the lead consultant. The main tasks of the group are technical oversight and regional coordination including validation of tools and methods and reports.

2.2 Establishment of the national technical working group (TWG).

The TWG will coordinate the day-to-day activities for the implementation of the programme review throughout the entire process.

Composition of the TWG

The TWG will be shared by the national consultant and will comprise:

- ✓ National AIDS Programme Manager
- ✓ Monitoring and evaluation focal point in the NAP
- ✓ Health system focal point in the ministry of Health
- ✓ Nongovernmental organization representative
- ✓ National consultant
- ✓ International consultant
- ✓ WHO Country Office
- ✓ IOM Country Office

Roles and responsibilities

The TWG has the following tasks:

- ✓ Prepare the background documents.
- ✓ Obtain relevant information for a desk review.
- ✓ Prepare a list of key informants

- ✓ Provide technical support to the organization of the national stakeholders meeting to review and discuss the review findings and to identify and agree on the priorities for the future MER grant
- ✓ Provide inputs on the draft recommendations of the national stakeholders meeting.

2.3 Organizing the national stakeholders' consultation.

In the interest of fostering common ownership and shared responsibility and accountability, the NAP will work with major stakeholders in carrying out the review. A stakeholders consultation will be organized when the draft findings of the review are finalized. During this meeting, the primarily findings of the review will be presented, discussed, completed, and endorsed by the stakeholders. Participants will also identify main priority actions for the future MER grant will be identified and endorsed based on the main findings of the review.

Objective of the consultation

The main objectives of the meeting:

1. Discuss and complete the information obtained from the programme review.
2. Agree on the key priorities taking into consideration the review findings

Participants

Participants should be key stakeholders, including representatives of institutions and organizations whose collaboration and contributions are required to accelerate HIV testing and treatment scale-up. Stakeholders may include:

- ➢ Civil society/nongovernmental organizations.
- ➢ Health government sectors: health system strengthening, MNCH, Harm reduction programme ….
- ➢ Relevant non-health government sectors
- ➢ key development partners.
- ➢ PLHIV
- ➢ UN Agencies

Expected outputs

- ➢ Consensus on main findings,
- ➢ Agreement on the key priories for the future of the MER grant

2.4 Developing timeline and assigning responsibilities by actions for the review implementation

3. Data collection methods and data sources

In conducting programme reviews, the primary focus should be on identifying and improving on the results of the programme. The aim of a programme review should be to assess the results a programme is

producing in relation to the priorities defined in the strategic and operational plans. A programme review needs to assess all levels of the results chain (Fig.1). The review should, first and foremost, consider the impact the programme is having in changing HIV incidence and mortality among the people being served.

Fig. 1 Results chain

Source: Guide to conducting programme reviews for the health sector response to HIV; WHO Geneva

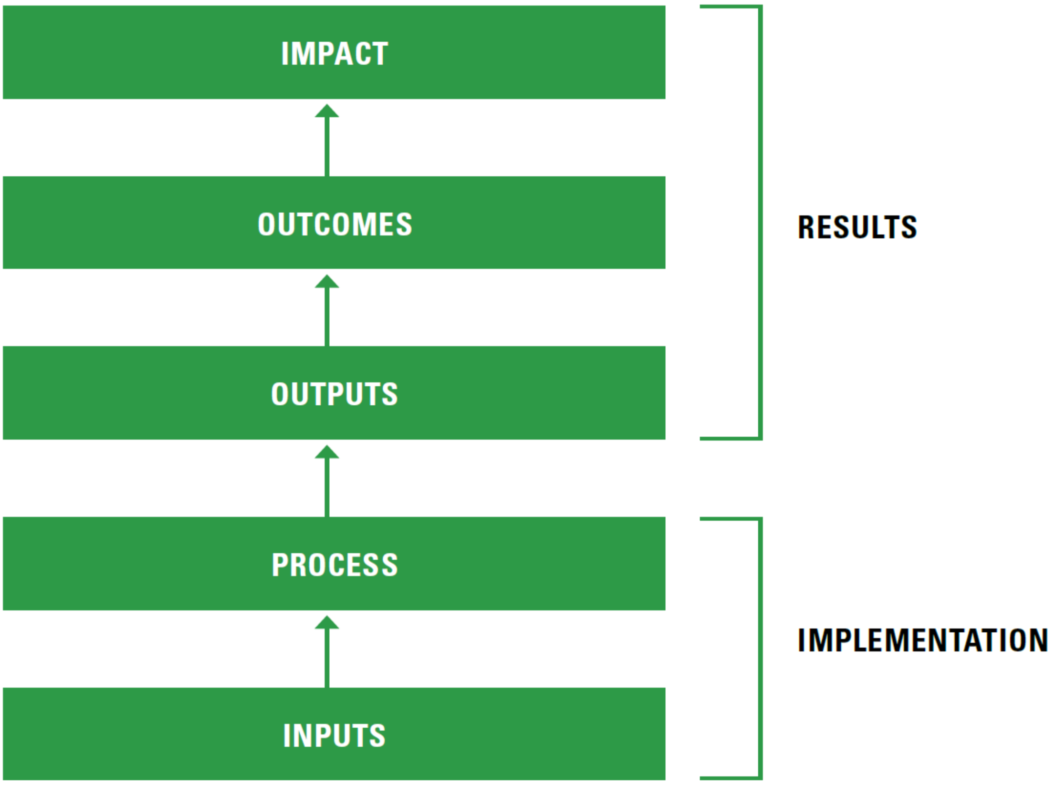

The results chain describes the relationship between different levels of results. Figure 1 shows how different levels of results are linked in a results chain. The elements at each level of the results chain must be necessary and sufficient to produce the required change at the next level.

WHO published consolidated strategic information guidelines for HIV in the health sector in 2015. These guidelines propose a monitoring and evaluation framework built on measuring indicators that reflect the epidemic and health system context, inputs, outputs, outcomes and impact. This framework has at its centre the HIV care cascade and related indicators (Fig. 2).

Fig 2. Global top 10 indicators

Source: Consolidated strategic information guidelines for HIV in the health sector. Geneva: World Health Organization; 2015

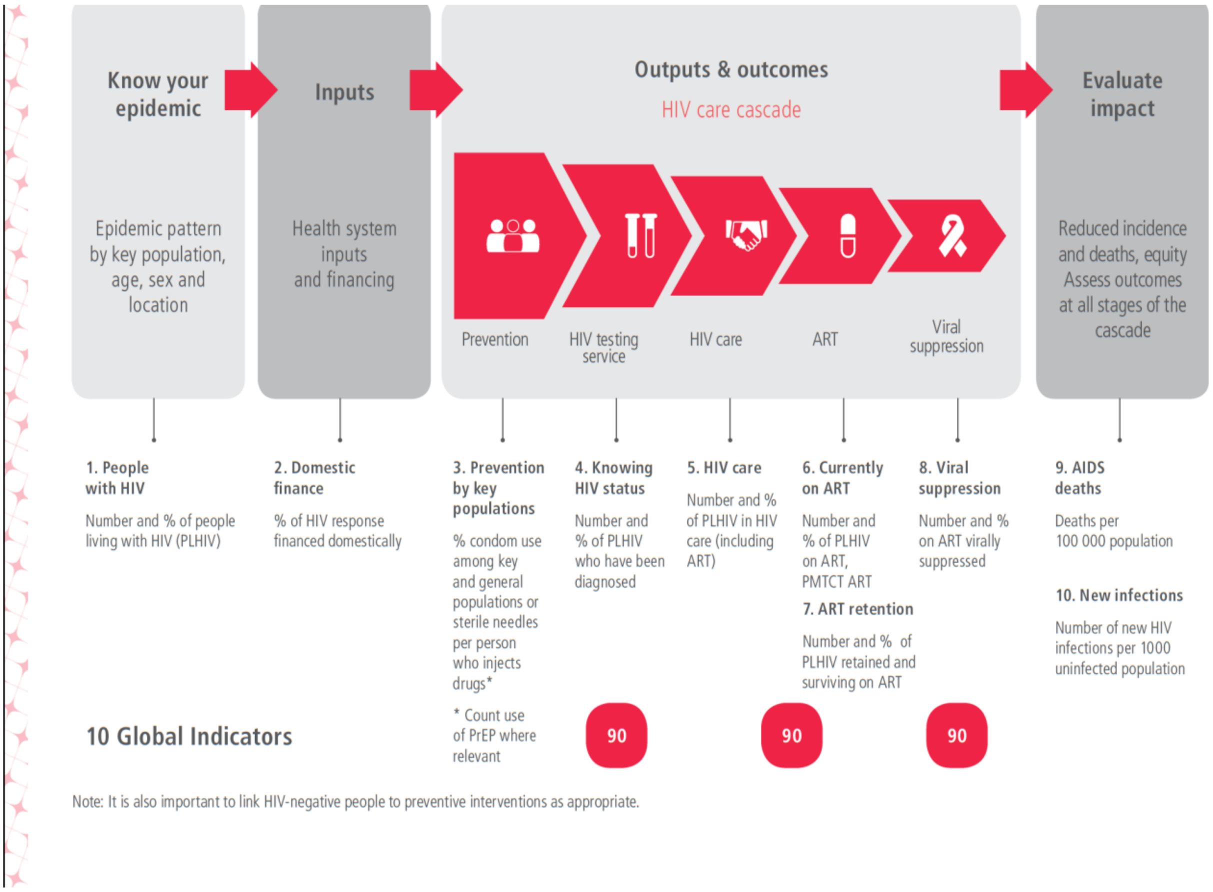

Programme review must assess performance of the national HIV programme across the results chain. Reviews must begin with analysing the impact achieved by the programme in terms of HIV incidence and mortality. The impact should be linked back to the programme outcomes, which, in turn, should be linked back to inputs and outputs. Recommendations of the review should highlight critical changes at the various levels of the results chain that are necessary to increase impact and improve programme performance.

Annex1 shows how to approach the review. It is based on the five questions that can guide the interpretation of programme performance through the review process. These questions relate directly to the main information domains (inputs, process, outputs, outcomes, and impact) defined in the common M&E framework above (Fig 2.). It assists in looking for and organizing the information that is required for the review. The table indicates the type of information required by basic review question and by information domain, it indicates the possible sources and compiles a list of required documents per type of required information. Annex1 can be considered as a tool that will help for the three stage of the review including the desk review.

Regardless of how well a review is planned, new questions or lines of enquiry may arise while gathering the information. These new questions can be added to the tool, as necessary.

There are essentially three stages to conducting national programme reviews. However, we might need to be flexible in the implementation of the review particularly with data collection. The three countries are

on one hand facing unprecedented degrees of security and displacement challenges and on the other suffering from the COVID-19 pandemic.

➢ ***The desk analysis stage***:

The desk review constitutes an important step in the process of reviewing the national HIV programme. It provides the evidence base for the review. It entails reviewing all existing documentation relating to the issues covered in the review to develop as complete a picture as possible of the current state of the programme. The desk review should be undertaken before and should inform the field review.

➢ ***The second stage is the field review:***

Its purpose is to assess the organization, capacity and delivery of services in real time. The field review involves a range of activities including technical briefing, stakeholder interviews and site visits.

Table 2 shows source of information related to the data collection methods mentioned above.

Table 2. Data collection methods and sources of information

Source: HIV test-treat-retain cascade analysis; Guide and tools 2017 version; WHO/Regional Office for the Eastern Mediterranean

Data collection method	Sources of information
Desk review	National policies and strategies; guidelines for HIV testing and counseling and HIV clinical management; standard operating procedures; annual programme reports; programme reviews and monitoring and evaluation reports; clinic registers; cohort analysis reports
Interviews/discussions with stakeholders	Policy makers, public, private and nongovernmental organization health service providers, social service providers, clients of services, PLHIV associations, civil society organizations, law enforcement, prison authorities, individuals from key populations, health workers (e.g. voluntary counselling and testing [HCT] staff, HIV clinic staff, antenatal staff, tuberculosis [TB] clinic staff, nongovernmental organization staff), national AIDS programme team
Site visits and observations	Observations of service organizations, patient flow in health facilities and community services, client and patient registers

➢ ***The third stage of a programme review includes an overall analysis of the findings***

The analysis will be framed by the five questions that can guide the interpretation of programme performance through the review process. As mentioned before, the questions are as follows:

- **Are the right things being done?**
- **Are they being done in the right way?**
- **Are they being done on a large enough scale?**
- **Are the right people being reached?**
- **Is the programme making a difference?**

4. Collecting information

It is necessary to first identify the information and data that are available and examine the quality of existing data. The quality of information available usually depends on the strength of the information systems that are in place. Strong monitoring and evaluation systems are more likely to produce high-quality data and vice versa. The following can be considered with respect to the quality of data to be included in the review:

- ✓ Data generated by reputable national and international institutions are likely to be of good quality.
- ✓ Data that have gone through international validation processes, such as through global reporting mechanisms are likely to be of good quality.

Gathering the right information is one of the most important steps in conducting a programme review. The analysis, findings and conclusions from the review largely depend on the quality of information gathered in the process.

4.1 Desk review and analysis

The desk review constitutes an important step in the process of reviewing the national HIV programme. It provides the evidence base for the review. It entails reviewing all existing documentation relating to the issues covered in the review to develop as complete a picture as possible of the current state of the programme. The desk review should be undertaken before and should inform the field review.

The desk review involves compiling available data on the areas to be covered in the review (annex1). This should start with impact data (incidence and mortality) at national and subnational levels. It should include data on related outcomes (coverage of HIV services, behaviour change and risk reduction) and inputs (policies, plans, resources and service availability). The review should use the global 10 indicators of the WHO monitoring and evaluation framework (Fig 2.):

- **HIV prevalence data** that are granular and disaggregated (and costing data) to focus efforts on the epidemic (Indicators 1 and 2)
- **Key population and outreach data** (Indicators 3 and linked to 4 and 5)
- **Case and patient reporting**: patient, testing and PMTCT data that are increasingly individual and linked the delivery of services (Indicators 4-8)
- **Practical impact evaluation**: to assess impact on incidence and mortality and adjust programmes accordingly (Indicators 9 and 10).

The desk review will also identify gaps in the information from the desk review and information that needs to be verified further.

The findings from the desk review are complemented by additional real time information collected during the review through methods such as interviews and site visits.

4.2 Field review

The field review should build on the findings of the desk review. It serves as a means of verifying the findings of the desk review, seeking explanations for these findings and filling information gaps. it complements the desk review to provide a more complete picture of the national programme. It involves interviews and discussions with key informants at the various levels of the health system as well as inspection and assessment of facilities and service delivery processes.

A. Interviews

Interviews provide qualitative information. They can be conducted

- with individuals in person or by completing a questionnaire
- and with groups as focus group discussion

Individual interviews are conducted with key informants such as policy-makers, programme managers, representatives of affected populations, implementers and donors. A questionnaire has been developed using standardized questions (Annex 3).

Table 2 shows possible people to be interviewed.

Table 2: Possible people to be interviewed

**Government**
• Programme managers and other programme personnel
• Policy-makers
• Related institutions (such as regulatory) and other sectors
**Service providers**
• Public
• Civil society
• Private
**Service users and beneficiaries**
• Women, men and young people living with HIV
• Key populations
• Local communities
**Interest groups**
• Local leaders
• Advocacy groups
• Professional associations
• Experts

Development partners
• Donors
• Technical assistance providers

The review team members will use the same questionnaire (annex 2) to collect information through the interviews and group discussion. This will make analysing the information easier. However new questions may arise while gathering the information. These new questions can be added to the questionnaire, as necessary.

B. Site visit

Site visits are conducted to observe how HIV services are being delivered and assess the existing capacity to deliver them. They can sometimes be used to verify the information obtained through desk review and interviews.

Few sites will be selected. Site selection will be mainly based on the relevance of the site to the purpose of the review. Table 3 shows examples of sites to be visited.

Table 3: Examples of services to be assessed in site visits

Level	What to look for
**Community**	✓ Type of community activities being carried out<br>✓ Who is involved<br>✓ Links between the community and the health system
**Health facility**	✓ Type of services, including diagnostics<br>✓ Service providers<br>✓ Type of facilities<br>✓ Equipment<br>✓ Availability of drugs<br>✓ Use of services<br>✓ Records management
**District**	As above plus<br>✓ Organization of district health services
**Province**	As above plus<br>✓ Referral system<br>✓ Management and supervision
**National**	As above plus<br>✓ Resource allocation

	✓ Procurement and distribution of commodities<br>✓ Training and capacity-building

Annex 1

Organization of the information required for the review by basic review question and by information domain

Basic review question	Information domain	Type of information required	Sources of information
Are the right things being done?	Inputs	- Adequate policies, plans and targets defined.<br>- Appropriate interventions for the type of epidemic and right population groups identified.<br>- Adequate resources available and allocated in line with priorities.<br>- Sufficient human resources, infrastructure, equipment, supply chain and<br>- information systems	- National development strategy or plan<br>- Health sector policies, strategies and plans<br>- National HIV policies, strategies and plans<br>- United Nations global or country reports
Are they being done in the right way?	Process	- Service delivery models appropriate for reaching the right population groups.<br>- Decentralization of services and community empowerment.<br>- Partnership and inclusive planning, implementation, and accountability.<br>- Integration with other health and development programmes.<br>- Effective programme management, coordination, and quality assurance.	- Operational and intervention plans<br>- Service delivery guidelines and protocols<br>- Estimates of resources needed<br>- Administrative records<br>- Programme budgets<br>- Donor commitments<br>- National AIDS spending assessments<br>- Logistics Management Information System
Are they being done on a large enough scale?	Outputs	- Type and quantity of services and products provided.<br>- Number and distribution of service delivery sites relative to target populations	- Facility records and reports<br>- Service availability and readiness index<br>- Implementation progress reports
Are the right people being reached?	Outcomes	- Coverage of services.<br>- Behaviour change.	- Monitoring and evaluation reports<br>- Facility records and reports<br>- Population surveys (Integrated Biological and Behavioural Surveillance, key populations)<br>- Research and study papers

Is the programme making a difference?	Impact	- HIV incidence in general population and specific populations.<br>- AIDS mortality in general population and specific populations.	- HIV surveillance reports<br>- AIDS indicator surveys<br>- Mode-of-transmission surveys<br>- Vital registration reports<br>- Demographic and Health Surveys<br>- Cost–effectiveness and cost–benefit analysis<br>- Research and study papers<br>- Other studies

Annex 2

Checklist for collecting information

✓Comprehensive list of information required and sources is developed

✓Tools for data collection are developed

✓Programme briefing is arranged

Desk review

✓Documents are listed

✓Documents are compiled

✓Documents are allocated to reviewers

Field review

✓ People to be interviewed are identified

✓ Interview questions and questionnaires are developed

✓ Interview schedule is developed

✓Interviewees are informed

✓Sites are identified

✓ Visit tools and checklists are developed

✓Visit teams are defined

✓Sites are informed

✓Travel logistics are arranged

Annex 3

QUESTIONNAIRE

Questions are arranged by HIV intervention areas and by main domain. It is an indicative list of questions and should not be necessarily be taken in its entirety.

Questions have been adapted to the current programme review purpose and context from the WHO guide for conducting an HIV programme review and from the WHO/EMRO HIV test-treat-retain cascade analysis, guide, and tools.

**HIV services**	
**Main domains**	**Key questions and areas to examine**
**1. Are we doing the right things?**	**Are there adequate and updated policies, plans and guidelines in place?**<br>• National Strategic plan (NSP) based on the epidemiological situation in the country<br>• Populations at higher risk of HIV infection prioritized in the NSP<br>• National targets defined in the NSP<br>• National costed operational plan<br>• Updated testing and clinical guidelines<br>• Consistency between national policies/operational guidelines and international standards<br>**Are there adequate funds and humane resources available to implement the operational plan?**

	• Funds available and sources (domestic and external)<br>• Financial gap<br>• Infrastructure<br>• Equipment<br>• Supply and chain<br>**Is there adequate information system to inform policy, planning and implementation of HIV services?**<br>• National HIV monitoring and evaluation system<br>• National HIV monitoring and evaluation plan/framework<br>• Key HIV indicators defined<br>• Use of evidence for informed policy, strategies and plan on national HIV/AIDS programme<br>• Use of data for estimating effectiveness and impacts of national HIV/AIDS
**2. Are we doing them right?**	**Is the model of service delivery appropriate to scale up HIV testing and treatment as required?**<br>• Distribution of services relative to target population(s)<br>• Levels of service delivery<br>• Primary<br>• Secondary<br>• Tertiary<br>• Community support<br>**Is there an effective HIV programme management?**<br>• Decentralization of services and community empowerment.<br>• Partnership and inclusive planning, implementation, and accountability.<br>• Integration with other health and development programmes.<br>• Coordination.

	• Quality assurance.
**3. Are we doing them on sufficient scale?**	**Are there adequate number and distribution of sites providing testing and treatment services?**<br>• Number and distribution of testing services.<br>• Number and distribution of health facilities that offer treatment services.<br>• Number and distribution of service delivery sites relative to target population.
**4. Are we reaching the right people?**	**Is there sufficient access to HIV services by the affected populations?**<br>▪ **Coverage services**<br>• Coverage of services.<br>• Number and % of PLHIV who have been diagnosed<br>• Number and % of PLHIV in HIV care (including ART)<br>• Number and % of PLHIV ART PMTCT ART<br>• Number and % of PLHIV retained and surviving on ART<br>• Number and % on ART virally suppressed<br>▪ **Behaviour change.**<br>• % condom use among key and general populations or sterile needles per person who injects drugs
**5. Are we making a difference?**	**What is the impact of HIV services?**<br>• AIDS deaths**:** Deaths per 100 000 population.<br>• HIV new infections: Number of new HIV infections per 1000 uninfected population
**Community Systems**	
**Enabling environment**	• Are there adequate efforts to develop an enabling and responsive

	environment through community-led documentation, policy dialogue and advocacy?<br>- Monitoring and documentation of community interventions<br>- Participation of community actors in national consultative forums<br>- Issues of key affected populations reflected in national policies, strategies, and plans<br>- Documentation of key community-level challenges and barriers to delivering and accessing services<br>-
**Community activities and service delivery**	• Are community organizations being supported to delivery and use quality services?<br>- Mapping of community health and social support services<br>- Identification of obstacles to accessing and using available services<br>- Technical and financial support
**Monitoring, evaluation, and planning**	• Do community organizations have sufficient capacity for monitoring and evaluation and evidence-building?<br>- M&E staff in community organizations<br>- Exchange visits and peer-to-peer learning and support on community M&E<br>- National plans, strategies, and policies relevant to communities<br>- Community-level M&E and operational plans, including reporting systems,
**Health system**	
**Leadership and governance**	• To what extent are HIV related issues addressed at higher levels of

	government?<br>- Executive<br>- Legislature<br>• Which stakeholders have influence on policy and strategy development – and which not?<br>• Are other government sectors, civil society, community members and private sector providers involved?<br>• Is the development of policies and strategies for HIV integrated in health policy/strategy development processes in the country – or is it happening through separate processes?<br>• Which body has administrative responsibility for HIV service delivery? (same body as for other health services? National AIDS Programme?)<br>• Does any of the above have an impact on access and/or utilization of HIV services?<br>• Is there adequate coordination between activities of partners involved in delivering HIV services?<br>- Public sector<br>- Private sector<br>- Non-governmental organizations<br>- Academia<br>- Development partners
**Financing**	• Is there a National Strategic Plan and costed operational plan for it implementation?<br>• Is the HIV/AIDS program appropriately costed in line with current and projected requirements of the National Strategic Plan and costed operational plan?<br>• Which bodies decide on allocation of: (1) national budget; and (2) larger donor funds?

	• Are there obstacles to investing in certain public health approaches due to restrictions imposed by government or donors? (such as obstacles to contracting nongovernmental organizations or private sector providers)<br>• What are the financing modalities for HIV services? (this may vary between services)<br>- Government budget?<br>- Health insurance/pooling/ pre-payment?<br>- User out-of-pocket?<br>- External donor funds (such as The Global Fund)?<br>• What are the positive/negative effects of the financing modalities for HIV services on access and/or utilization?
**Human resources**	• Are human resources for HIV service delivery adequate in number, professional skills and competences?<br>• Which body is responsible for human resources planning for HIV? Is human resources planning for HIV integrated in overall human resources planning for health?<br>• Is the staff of HIV services an integral part of the overall pool of staff of the health services where they work?<br>• On whose payroll is staff providing HIV service? (this question is linked to the financing mechanism)<br>- Service provider?<br>- National AIDS Programme?<br>- Nongovernmental organization?<br>- United Nations?<br>- Other?<br>• Are staff/volunteers sufficiently motivated? Is there a strategy/system in place to keep health workers motivated? Is there a special strategy/system for staff providing HIV services?<br>• Does any of the above have an impact on access and/or utilization of HIV services

**Procurement/supply management**	• Are there appropriate and sustainable PSM policy, strategies and plans?<br>• Is procurement forecasting evidence-based?<br>- Estimation of quantities of commodities necessary for expanded services from longer-term perspective<br>- Balancing ART procurement between treatment and preventive purposes<br>• Are appropriate procurement methods adopted and applied, according to the national PMS policy and international procurement guidelines?<br>- Adequate application of nationally/internationally competitive bidding process<br>- Function of procurement panel/committee
**Health information system**	• Is the national Health Information System measuring key disease burden and service coverage indicators<br><br>• Is accurate, strategic information available and accessible to all stakeholders, and used for evidence-informed policy and program planning, and resource allocation.
**Special issues to be explored**	
**COVID-19 Challenges for ensuring continuity of HIV services**	• How does the COVID-19 situation affect the country ability to achieve the annual targets for this year?<br>• What are the main HIV services affected by the COVID-19 situation?<br>• Is there a plan for recovery of disrupted services and identify alternative approaches to service delivery with and without COVID-19 lockdown?<br>• Is there a system to monitor closely the impact of COVID-19 on service delivery and take timely remedial actions to mitigate service disruption?

	• What is the biggest COVID-19 related challenge for ensuring continuity of HIV services?
**Emergency, security, and displacement challenges**	• How does the current situation and context affect country ability to plan and to implement the national HIV response?<br>• What are the main HIV services affected by the current situation?<br>• Is there a system to monitor the impact of current situation on service delivery and take timely remedial actions to mitigate service disruption?<br>• Is there an emergency plan for recovery of disrupted services?<br>• Are alternative approaches, measures put in place to maintain continuity of HIV services?<br>• What is the biggest situation related challenge for ensuring continuity of HIV services?

Annex 5: Questionnaire used for the key informants' interviews.

Questionnaire – Key informants' interviews
Start with an introduction: IOM/WHO/UNAIDS, occasion for the next 3 years; MER, and we want to hear from you for a better planification.

1. **<u>Input</u>**

1.1. Are there adequate and updated policies, plans and guidelines in place?

Do you have a strategic plan for your organization? An operational plan with detailed activities, targets, tools and guidelines? If yes, can we get it? Is it linked to the national strategic plan?

1.2.Are there adequate funds and human resources available to implement their operational plan?

- What are the available funds available and human resources (domestic and external) to implement your plan?
- What are your financial gaps?
- Infrastructure; equipment

1.3.Procurement, supply and management

Are appropriate procurement methods adopted and applied, according to the PMS policy?

Condoms? Test kits? How do you stock it?

1.4. Is there adequate information system to inform policy, planning and implementation of HIV services?

HIV monitoring and evaluation plan/framework? Key HIV indicators defined? Use of evidence for informed policy, strategies and plan for your organization?

Any system to monitor your activities? Follow-up the progress of your activities? Platform link? Usage of own data? Informed or informing?

2. ***<u>Process:</u>*** *Is the model of service delivery appropriate to scale up HIV testing and treatment as required?*

- Distribution of services relative to your target population(s)
- Which regions? Why this region is covered or not? What are the barriers?
- Levels of service delivery: centralized or decentralized? If decentralized: which regions?
- Partnership and coordination with the NAP and other programmes? *(surtout ID: national guidelines? Partnership?); tertiary (surtout ID: national guidelines? Partnership?)*

3. **Outputs:** *Are there adequate number and distribution of sites providing testing and treatment services?*

- Number of MSM? KVPs? tested? treated? Correct the denominators if possible; which NGO does the most?
- Number and distribution of testing services (sites) for the year 2020
- Number and distribution of health facilities that offer treatment services for the year 2020
- Number and distribution of service delivery sites relative to the population you are working with for the year 2020

(Distribution refers to the geographic distribution)

4. **Outcomes:** *Is there sufficient access to HIV services by the affected populations?*

What is the number of people tested in 2020? The number of people treated? The percentage of people retained in care? Virally suppressed? What is the percentage in regards with their targets? If not reached, why? Are there missed opportunities? Are there plans for the next years?

5. **Enabling environment:** *Are there adequate efforts to develop an enabling and responsive environment through community-led documentation, policy dialogue and advocacy?*

- Is there a participation of community actors in national consultative forums: guidelines? Policies?
- Do you document key community-level challenges and barriers to delivering and accessing services: get the documents? If not, get an oral answer.

6. **Community activities and service delivery:** *Are community organizations being supported to delivery and use quality services?*

- Is there a mapping of community health and social support services?
- Identification of obstacles to accessing and using available services *(Only NGOs)*
- Technical and financial support *(Only NGOs)*

7. **COVID19 challenges**

- How does the COVID-19 situation affect your agency ability to achieve your annual targets for this year?
- What are your main HIV services affected by the COVID-19 situation?
- Is there a plan for recovery in your institution of disrupted services and identify alternative approaches to service delivery with and without COVID-19 lockdown?

- Is there a system to monitor closely the impact of COVID-19 on service delivery and take timely remedial actions to mitigate service disruption?
- What is the biggest COVID-19 related challenge for ensuring continuity of HIV services?

8. **Emergency, security and displacement**

- How does the current situation and context affect country ability to plan and to implement the national HIV response?
- What are the main HIV services affected by the current situation?
- Is there a system to monitor the impact of current situation on service delivery and take timely remedial actions to mitigate service disruption?
- Is there an emergency plan for recovery of disrupted services?
- Are alternative approaches, measures put in place to maintain continuity of HIV services?
- What is the biggest situation related challenge for ensuring continuity of HIV services?

Annex 6: Minutes of the stakeholders meeting.

Stakeholders meeting – HIV programme review Lebanon

Presentation and discussion of draft findings of the HIV programme review in Lebanon

28 December 2020

Objectives of the meeting:

- Present and discuss the draft findings of HIV programme review;
- Complete the findings;
- Endorse the results;
- Identify and agree on key priorities for the future MER grant.

Participants (check full list of organizations, names and email of attendees at the end):

- The NGOs that are contracted by the NAP as partners for the implementation of the MER-2 initiative;
- UNHCR focal point on HIV;
- IOM technical officer on MER initiative;
- NAP team;
- The international and national consultants

Minutes of the meeting (10 AM – 12 PM):

The NAP manager (Dr Mostapha El Nakib) introduced the attendees, the session and its objectives.
The international consultant (Dr Hamida Khattabi) introduced the main objectives of the HIV programme review, and how it is timely for Lebanon since it coincides with the NSP 2016-2020 end.
The national consultant (Dr Ismael Maatouk) delivered the power point presentation:

- Thanking the international consultant, the NAP manager, WHO and UN agencies, the NGOs and key informants who collaborated to complete the review;
- Objectives of the meeting;
- Background: Global AIDS response progress, regional situation with the emergency human crisis, MER initiative, the need of robust multi-country programme reviews and countries prioritized;
- Rationale of the programme review in Lebanon;

Methods used for the review based on the comprehensive programme review spectrum (inputs to impact) and the 10 global core indicators; the development of the assessment tool for data collection by the international consultant that was organized by basic review question and by information domain and that consisted of 3 phases: desk review, key informants' interviews and field visit;

- Main findings:
  - Overall findings with overall impact of the programme on incidence,
    - And mortality: HIV incidence in the general population and among some key populations, both indicators not determined in vulnerable populations such as migrants, displaced and refugees;
    - Overall findings of the three 90s targets and how the first 90 was exceeded whereas the two others were not reached;

- Overall findings of the MER-2 three targets and how the treatment of PLHIV was reached whereas the testing and training targets were not due to the major challenges that happened in Lebanon since October 2019 from the economic and political crisis to the COVID19 challenges and restrictions;
- Findings by strategic directions of the NSP 2016-2020 that go in line with the Global Health Sector strategy of HIV 2016-2020 of the WHO and with the 8-result areas of the UNAIDS strategy 2016-2021;
- For each of the 6 findings by strategic directions of the NSP 2016-2020, the achievements were shown followed by main challenges and main gaps;
- The COVID19 challenges and the Beirut blast (August 4th, 2020) were disseminated at the end of the findings;
- The draft of the 5 key priority areas that were proposed by the consultants were given along with 3 key recommendation on the development of the next NSP 2021-2023 based on the HIV programme review findings

The session was followed by a discussion with the stakeholders and the following notes were highlighted:

- Full agreement on the 5 key priority areas proposed and endorsement of the HIV programme review draft of results;
- The need of strong coordination between the NAP and the NGOs to reach a common understanding of what everyone is doing to avoid any duplication of activities. remarks by the NAP manager, the international consultant and UNHCR focal point on HIV, and 2 other NGOs and endorsed by participants;
- The problem of HIV co-morbidities treatment and management which have been ignored to date and which add expanses to the already expansive chain of tests of PLHIV;
- the international consultant explained how this programme review can be used to have a 3-year national strategic plan where targets are set including coverage and impact and to mobilize resources from future MER grant, 5% French initiative and other donors and partners through a cooperation between the NGOs and the NAP to avoid duplicate activities.

List of participants

Organization/Name	Email
**Proud Lebanon/**Bertho Makso	berthomakso@gmail.com
**Marsa/**Sara Abu Zaki	sara.az@marsa.me
**SIDC/**Patrick Farah	p.farah@sidc-lebanon.org
**SIDC/**Nadia Badran	nbadran@sidc-lebanon.org
**Skoun/**Sabine Sadaka	sabine@skoun.org
**Ajem/**Josee El Hayek	joseehayek@gmail.com

**MENAHRA**/Elie Aaraj	eaaraj@menahra.org
**NISCVT/**Samah Al Hussein	peereducation@socialcare.org
**UNHCR**/Marie AKiki	akiki@unhcr.org
**IOM**/Dr Nada Najem	nnajem@iom.int
**From the NAP:**<br>- Dr Moustafa El Nakib<br>- Dr Rima Ferzli<br>- Abir Hassrouni<br>- Moubadda Assi	
**International consultant : Dr Hamida Khattabi**	

<u>Were absent :</u>
Sabah Association;
Lebanese AIDS Society;
Dar El Fatwa;
Dar El Amal.
