# Supplementary material for: Progress towards the 95–95–95 targets to end HIV by 2030 in Lebanon, 2023
Source: PLoS One. 2025 Jun 13;20(6):e0321868. doi: 10.1371/journal.pone.0321868 (PMC12165419; doi:10.1371/journal.pone.0321868)
Supplement: S3 File — Lebanon HIV IBBS 2018 final report. (PDF) [file pone.0321868.s003.pdf]

An Integrated Bio-Behavioral Surveillance Study among Two Vulnerable Groups in Lebanon: Men Who Have Sex with Men and Commercial Sex Workers

Final Report

Table of Contents

ABBREVIATIONS AND ACRONYMS 4
LIST OF TABLES 5
LIST OF FIGURES 6
ADOPTED DEFINITIONS 7
EXECUTIVE SUMMARY 8
PROJECT BACKGROUND 9
Rationale and Survey Objectives 10
METHODOLOGY 11
Formative Assessment 11
IBBS Implementation 14
Population Size Estimation 15
QUALITY CONTROL AND ASSURANCE 15
ETHICAL CONSIDERATIONS 16
MAIN FINDINGS 17
Formative Assessment 17
Description of KIIs 17
MSM Community in Lebanon 17
CSW Community in Lebanon 19
IBBS Results for MSM 20
Eligibility Screening 20
Background Characteristics 21
Sexual History and Practices 22
Condoms and Lubricants 27
STIs 33
Knowledge, Opinions, and Attitudes towards HIV/AIDS 36
Stigma, Discrimination and Violence 44
Alcohol and Drug Use 45
IBBS Results for CSWs 48
Eligibility Screening 48
Background Characteristics 48

Sexual History and Practices 50
Condoms and Lubricants 53
STIs 58
Knowledge, Opinions, and Attitudes towards HIV/AIDS 61
Stigma, Discrimination and Violence 68
Alcohol and Drug Use 69
Global AIDS Monitoring Indicators 72
Population Size Estimation 73
Estimated Population Size for MSM 74
Estimated Population Size for CSWs 74
LIMITATIONS OF THE STUDY 75
RECOMMENDATIONS 76
APPENDICES 78

ABBREVIATIONS AND ACRONYMS

AIDS	Acquired Immunodeficiency Syndrome
ART	Antiretroviral Therapy
CRD	Connecting Research to Development
CSW	Commercial Sex Worker
HIV	Human Immunodeficiency Virus
KI	Key Informant
KII	Key Informant Interview
KP	Key Population
IBBS	Integrated Biological and Behavioral Survey
IOM	International Organization for Migration
IRB	Institutional Review Board
L-CSW	Lebanese Commercial Sex Worker
L-MSM	Lebanese Man who Have Sex with Men
MSM	Man who Have Sex with Men
NAP	National AIDS Control Program
NGO	Non-Governmental Organization
NL-CSW	Non-Lebanese Commercial Sex Worker
NL-MSM	Non-Lebanese Man who Have Sex with Men
PSE	Population Size Estimates
SPSS	Statistical Package for Social Sciences
STI	Sexually Transmitted Infection
TLS	Time Location Sampling
UNAIDS	Joint United Nations Programme on HIV/AIDS
UNHCR	United Nations High Commissioner for Refugees

LIST OF TABLES

Table 1. Types and Geographical Distribution of Localities (N=152). 12
Table 2. Description of Key Informant Interviews (N=30). 17
Table 3. Eligibility Screening of MSM (N=912). 20
Table 4. Background Characteristics of MSM. 21
Table 5. Self-categorization of MSM. 23
Table 6. Sexual History and Practices of MSM with Females Partners. 24
Table 7. Sexual History and Practices of MSM with Male Partners. 25
Table 8. Access to Male Condoms for MSM. 28
Table 9. Access to Female Condoms for MSM. 31
Table 10. Access to Lubricants for MSM. 32
Table 11. STIs among MSM. 34
Table 12. HIV/AIDS among MSM. 38
Table 13. Access to HIV and Syphilis Testing among MSM. 41
Table 14. Results of the HIV and Syphilis Rapid Tests for MSM. 42
Table 15. Stigma, Discrimination and Violence for MSM. 44
Table 16. Alcohol and Drug Use among MSM. 46
Table 17. Eligibility Screening of CSWs (N=536). 48
Table 18. Background Characteristics of CSWs. 49
Table 19. Sexual History and Practices of CSWs. 51
Table 20. Access to Male Condoms for CSWs. 53
Table 21. Access to Female Condoms for CSWs. 55
Table 22. Access to Lubricants for CSWs. 56
Table 23. STIs among CSWs. 59
Table 24. HIV/AIDS among CSWs. 62
Table 25. Access to HIV and Syphilis Testing among CSWs. 66
Table 26. Results of the HIV and Syphilis Rapid Tests for CSWs. 67
Table 27. Stigma, Discrimination and Violence for CSWs. 68
Table 28. Alcohol and Drug Use among CSWs. 70
Table 29. Global AIDS Monitoring Indicators. 72
Table 30. Population Size Estimates for MSM. 74
Table 31. Population Size Estimates for CSWs. 74

LIST OF FIGURES

Figure 1. Relationship Status of MSM, by Nationality. 23
Figure 2. First Male Partner of MSM, by Nationality. 27
Figure 3. Reasons for Not Using a Condom During the Last Anal Sex with a Male Commercial Partner for MSM, by Nationality. 30
Figure 4. Reasons for Not Using a Condom During the Last Anal Sex with a Male Non-Commercial Partner for MSM, by Nationality. 30
Figure 5. Reasons for Not Using Lubricants for MSM, by Nationality. 33
Figure 6. Prevalence of at Least one STI Symptom in the Last 12 Months among MSM, by Nationality. 36
Figure 7. Prevalence of All STI Symptoms in the Last 12 Months among MSM, by Nationality.... 36
Figure 8. Knowledge of at Least One Necessary Information about HIV/AIDS among MSM, by Nationality. 40
Figure 9. Knowledge of All Necessary Information about HIV/AIDS among MSM, by Nationality. 41
Figure 10. Final HIV Result for Lebanese MSM. 43
Figure 11. Final HIV Result for Non-Lebanese MSM. 43
Figure 12. Relationship Status of CSWs, by Nationality. 53
Figure 13. Reasons for Not Using a Condom During the Last Vaginal or Anal Sex with a Commercial Partner for CSWs, by Nationality. 55
Figure 14. Reasons for Not Using Lubricants for CSWs, by Nationality. 58
Figure 15. Prevalence of at Least one STI Symptom in the Last 12 Months among CSWs, by Nationality. 60
Figure 16. Prevalence of All STI Symptoms in the Last 12 Months among CSWs, by Nationality. 61
Figure 17. Knowledge of at Least One Necessary Information about HIV/AIDS among CSWs, by Nationality. 65
Figure 18. Knowledge of All Necessary Information about HIV/AIDS among CSWs, by Nationality. 65
Figure 19. Final HIV Result for Lebanese CSWs. 67
Figure 20. Final HIV Result for Non-Lebanese CSWs. 68

ADOPTED DEFINITIONS

Bisexual	A person who is attracted to and/or has sex with both men and women, and who identifies with this as a cultural identity.
Client	Female or male adult who buys sex
Commercial Sex Worker	Female or male adult or young person over the age of 18 who receives money or goods in exchange for sexual services, either regularly or occasionally
Consistent Condom Use	Using condoms for every act of sex and correct use as consistent use without any of the following: beginning sex without a condom, taking it off before finishing sex, flipping it over, condom breakage, or condom slippage
Forced Sex	An act of unwanted sex against a person's consent, compelled by physical force or threats of force
Key Informant	A person with whom an interview about a particular organization, social program, problem, or interest group is conducted
Key Population	Gay men and other men who have sex with men, sex workers and their clients, transgender people, people who inject drugs and prisoners and other incarcerated people are the main key population groups who often suffer from punitive laws or stigmatizing policies, and they are among the most likely to be exposed to HIV.
MSM	Male or transgender adult who has sex with males, regardless of whether he has sex with women or has a personal or social gay or bisexual identity
Sexually Transmitted Infection	Infections that are spread by the transfer of organisms from person-to-person during sexual contact
Survival Sex	Exchange of sex for material support

EXECUTIVE SUMMARY

Existing data indicate that Lebanon is a low-prevalence HIV country at the level of the general population. Concentrated epidemic is witnessed among Men who have Sex with Men (MSM); no data is present for Commercial Sex Workers (CSWs). Similar to other low-HIV prevalence countries, these key populations in Lebanon exhibit unique structural and behaviors risk factors, which put them at heightened risk for HIV infection, and transmission to members of their sexual networks. Despite the NAP's efforts to expand its HIV response mechanisms and scale-up prevention, testing, and treatment services, available information suggests limited services utilization by key populations and thus permits further exploration of where and among whom new HIV infections are most likely to occur.

An Integrated Biological and Behavioral Survey (IBBS) was conducted to explore high risk behaviors among Men who have Sex with Men (MSM) and Commercial Sex Workers (CSWs), to determine the prevalence of HIV and syphilis, and to provide estimates of the size of MSM and CSWs living in Lebanon.

Thirty key informant interviews were conducted to identify the localities where MSM and CSWs gather or work and detect potential collaborators who can act as gatekeepers to accessing MSM and CSWs. A behavioral survey was also carried out with 745 MSM and 525 CSWs. Data were collected by trained fieldworkers. Ethical guidelines were followed throughout the preparation and implementation stages.

Surveyed participants were relatively young. They had their sexual experience at a young age. Their educational level, employment status and self-identification on the basis of sexual orientation varied among MSM and CSWs.

Participants practiced a cluster of high risk behaviors. Engagement in sexual relationships with multiple partners, inconsistency in condom use with commercial and non-commercial partners, consuming alcohol and using and injecting drugs were common. Practices of risk behaviors were coupled with a limited knowledge on sexually transmitted infections (STIs) and Human Immunodeficiency Virus (HIV) transmission. Nevertheless, HIV testing was common.

The prevalence of HIV in the MSM sample was 12.0% for Lebanese MSM and 3.0% for non-Lebanese MSM. As for CSWs, 0.8% of Lebanese CSWs were found to be HIV-positive.

Population size estimates resulting from an IBBS are inaccurate and disposed to many potential biases due to the absence of a gold standard method. This study suggested a mixture of methods to reinforces confidence in estimates, provides upper and lower plausibility bounds, and reduces the likelihood that biases of any single method impact results: 1) Literature Review; 2) Enumeration; 3) Unique Object Multiplier; and 4) Wisdom of Crowds.

PROJECT BACKGROUND

Lebanon is a very low-prevalence Human Immunodeficiency Virus (HIV) country with estimates believed to be around 0.1% among the adult population (15 to 49 years of age)[1]. The latest spectrum estimates 2100 (1900-2400) people ages 15 and above to be living with HIV[1]. As of November 2017, 205 new cases of HIV were reported with most of the cases (46.8%) being within the 30-49 years old age group[2]. A cumulative number of 1304 individuals, of which 186 were new cases, were reported to be on antiretroviral Therapy (ART) in 2017[3]. Therapy coverage has increased by 16.6% in 2017 when compared to 2016[3].

Based on 2017 statistics from the National AIDS Program (NAP), the mode of transmission of HIV was totally through sexual activity[2]. A closer look at the classification among the sexual transmission mode, clearly identifies a dominant homosexual transmission at 54.1%[2]. Statistics also show that men who have sex with men (MSM) have a 75% condom use and an HIV prevalence ranging between 12.3% and 27.5%[1,4] . On the other hand, data on commercial sex workers (CSWs) is scarce however the Joint United Nations Programme on HIV/AIDS (UNAIDS) was able to estimate an HIV prevalence of 1% among CSWs and a 96.3% condom use[1].

Several factors, such as taboo and stigma, pose as recruitment barriers and hinder the availability of reliable epidemiological data related to key populations in Lebanon[5,6]. Additionally, the majority of the collected epidemiological information with regards to key populations is still limited to the area of Greater Beirut[6]. Regardless of organized efforts, the current surveillance system for sexually transmitted infections (STIs), mainly HIV, is barely satisfactory due to its sole reliance on passive reporting which often leads to inconsistency and lack of timeliness in the available epidemiological data, thus limiting a comprehensive understanding of the dynamics and trends of STIs[7].

Few bio-behavioral studies were carried out in the past in order to understand the behaviors of key populations in Lebanon. All conducted studies confirmed the engagement of MSM in risky behaviors such as not using condoms regularly, having multiple partners, participating in group

---

[1] UNAIDS. (2017). Country Factsheets - Lebanon | 2017. UNAIDS.
[2] NAP. (2018). National AIDS Control Program in Lebanon. Retrieved from Ministry of Public Health: https://www.moph.gov.lb/en/Pages/2/4000/aids
[3] Kattar, S. A., & Dosh, L. (2018). HIV/AIDS: Where Do We Stand in 2017. Human & Health, 48-50.
[4] Heimer, R., Barbour, R., Khouri, D., Crawford, F. W., Shebl, F., Aaraj, E., & Khoshnood, K. (2017). HIV Risk, Prevalence, and Access to Care Among Men Who Have Sex with Men in Lebanon. AIDS research and human retroviruses, 33(11), 1149-1154.
[5] NAP; & MoPH. (2008). An integrated bio-behavioral surveillance study among most at risk populations in Lebanon: Female sex workers, injecting drug users, men who have sex with men, and prisoners.
[6] Crossroads. (2015). Size estimation, risk behavior assessment, and disease prevalence in populations at high risk for HIV infection in Lebanon. Beirut.
[7] UNAIDS. (2014). Country Progress Report - Lebanon.

sex, selling sex, and not getting tested for HIV[4,5,6,8]. A bio-behavioral study conducted in 2008 showed that having more than 5 different clients per month is considered as a risky behavior for female sex workers. However, the same study demonstrated that preventive measures such as condom use was relatively high among female sex workers[5].

The United Nations High Commissioner for Refugees (UNHCR) estimates that Lebanon hosts around 1.1 million Syrian Refugees within its territory[9], in addition to 500,000 Palestinian refugees and 50,000 Iraqis also residing in the country[9]. The influx of refugees has challenged the Lebanese health services and increased the risk of acquiring different infectious diseases[9]. In 2013, 9% of patients sought treatment for STIs at primary health care centers[7]. STIs account for 4.55% of the total consultations of refugees and includes Hepatitis B and syphilis[7]. Additionally, according to research conducted by ABAAD in 2012, refugee women and girls have turned to prostitution as a mean for income generation and for meeting basic needs[9]. Furthermore, the results of a study conducted in 2013 among MSM (including Syrian and Iraqi refugees) in Beirut showed that 64% of them reported unprotected anal intercourse and of whom 23% had unprotected anal intercourse with men of HIV positive or unknown status[10]. Refugees were less likely to participate in unprotected intercourse (48%) when compared to Lebanese MSM (69%) and were also less likely to have been tested for HIV (46% vs 67%)[10]. Another study conducted in 2016 among MSM refugees in Beirut Lebanon showed that 84.6 % of respondents reported unprotected sex with 56.7 % of them having done so with men of positive or unknown HIV status. Furthermore, 2.7 % tested positive for HIV, and 36 % reported having engaged in sex work[11].

Finally, when it comes to population size estimates (PSE) data is lacking. The only available estimate available was carried out by UNAIDS in 2017, which showed that the number of CSWs in Lebanon is estimated to be 4220 while that of MSM is estimated to be 3114[1].

Rationale and Survey Objectives

The Lebanese NAP has been working hard to expand its HIV response mechanisms and scale-up prevention, testing, and treatment services. However, despite these efforts, available information suggests limited services utilization by key populations and thus warrants further in-depth

[8] Mahfoud, Z., Afifi, R., Ramia, S., Khoury, D. E., Kassak, K., Barbir, F. E., . . . DeJong, &. J. (2010). HIV/AIDS among female sex workers, injecting drug users and men who have sex with men in Lebanon: results of the first biobehavioral surveys. AIDS, 24(suppl 2):S45-S54.

[9] Hussein, I. H., Mortada, I., Geagea, A. G., & Jurjus, &. A. (2017). Communicable diseases among refugees with a focus on the Middle East. IntechOpen, 209-2018.

[10] Wagner, G. J., Tohme, J., Hoover, M., Frost, S., Ober, A., Khouri, D., . . . Mokhbat, &. J. (2014). HIV Prevalence and Demographic Determinants of Unprotected Anal Sex and HIV Testing Among Men Who Have Sex with Men in Beirut, Lebanon. Archives of sexual behavior, 43(4), 799-788.

[11] Tohme, J., Egan, J. E., Stall, R., Wagner, G., & Mokhbat, &. J. (2016). HIV Prevalence and Demographic Determinants of Unprotected Anal Sex and HIV Testing among Male Refugees Who have Sex with Men in Beirut, Lebanon. AIDS and Behavior, 20(3), 408-416.

exploration of where and among whom new HIV infections are most likely to occur. This will help plan a targeted HIV response and focus preventive efforts and resources in settings where they will have the greatest impact.

Thus, and in response to that gap, Connecting Research to Development (CRD) has conducted an Integrated Biological and Behavioral Survey (IBBS), with the support of the NAP and the International Organization for Migration (IOM), to explore high risk behaviors among MSM and CSWs, to determine the prevalence of HIV and syphilis, and to provide estimates of the size of MSM and CSWs living in Lebanon.

This study was planned to develop a better understanding of the prevention, care, and treatment needs of the MSM and CSW populations and to develop programs targeted toward MSM and CSWs.

The specific objectives of this survey were to:

- Estimate the population size and distribution of MSM and CSWs among the populations living in Lebanon.
- Estimate the prevalence of HIV, syphilis, and associated risk behaviors among the MSM and CSWs within this study.

METHODOLOGY

The methodology relied on three main pillars:

1. A formative assessment, which informed the methodological and logistical requirements for the successful implementation of the IBBS;
2. An IBBS, which permitted to gather data that was used to understand the characteristics and behaviors of MSM and CSWs and provide information on the prevalence of HIV, syphilis, and risk behaviors in these populations;
3. A population size estimation, which provided the NAP more information on the scope of the HIV epidemic allowing them to plan appropriate interventions and allocate sufficient resources.

The tools and forms created for this study are available in Appendices A to J.

Formative Assessment

Formative assessment was a significant component of getting ready to conduct the IBBS. The construction and depth of the formative assessment depended in part on how much was previously known about the target population, and on the strength of the present relationship between the target population and the investigators.

This step was guided by the NAP. It aimed at exploring the localities where MSM and CSWs gather or work, collecting demographic and other general data about the two populations, and identifying

potential collaborators from active organizations who can act as gatekeepers to accessing MSM and CSWs.

This step began by identifying a purposive sample of Key Informants (KIs). A total number of 30 Key Informant Interviews (KIIs) were conducted between September and November 2018 with informants at the national and district levels who are knowledgeable about MSM and CSW's networks in the country. This step resulted in a list of 152 localities, divided based on their geographical distribution, as seen in Table 1.

Table 1. Types and Geographical Distribution of Localities (N=152).

Area	Type of Venue	Number of Venues
Tripoli	Bathhouse/Spa	1
	Hotel	2
	Park	1
	Prison	2
	Pub/Night club	1
	Restaurant/Café/Bar	3
	Gym	1
	Street	11
Bekaa/Baalback	Bathhouse/Spa	1
	Hospital	1
	Hotel	7
	Informal settlement	12
	Non-governmental organization	3
	Prison	2
	Pub/Night club	1
	Restaurant/Café/Bar	2
	Street	2
Sin El Fil	Hotel	3
	Non-governmental organization	1
	Street	2
	Theaters/Cinema	1
Sodeco	Hotel apartments	1
	Restaurant/Café/Bar	2
Corniche El Nahr	Restaurant/Café/Bar	1
	Street	1
	Theaters/Cinema	2
Manara/Ain El Mreisseh	Hotel	2
	Pub/Night club	1
	Restaurant/Café/Bar	1
	Street	1
Baabda/Hazmieh	Commercial center	1
	Hotel	1
	Prison	1
	Restaurant/Café/Bar	1
Jal El Dib	Casino	2
	Pub/Night club	1
Burj Hammoud	Bathhouse/Spa	1

	Pub/Night club	1
	Restaurant/Café/Bar	1
Gemmayzeh	Guesthouse	1
	Non-governmental organization	1
	Pub/Night club	1
	Restaurant/Café/Bar	5
Badaro	Hotel	1
	Non-governmental organization	4
	Pub/Night club	2
Beirut	Commercial center	1
	Hotel	1
	Informal settlement	2
	Non-governmental organization	3
	Pub/Night club	2
	Restaurant/Café/Bar	2
	Street	7
Jdeideh	Non-governmental organization	1
Furn El Chebbak	Non-governmental organization	1
	Library	1
Dora	Non-governmental organization	1
	Restaurant/Café/Bar	1
	Street	5
Rawche	Restaurant/Café/Bar	2
	Street	1
Monot	Parking	1
	Pub/Night club	1
	Restaurant/Café/Bar	1
Zalka	Prison	1
	Pub/Night club	1
	Restaurant/Café/Bar	2
Hamra	Bathhouse/Spa	1
	Hotel	4
	Political party	1
	Restaurant/Café/Bar	6
	Street	2
Jounieh/Jbeil	Apartments	1
	Chalets	1
	Pub/Night club	1

Data were collected through face-to-face interviews, using an interview guide. The KII guide entailed questions about the nationalities of sex workers and MSM (separately), their preferred gathering places and times, the venues where they prefer taking the survey, their estimated number, and their behaviors. Informed consent was obtained from each participant prior to data collection. Personnel specifically trained on the study protocol, carried out the interviews in Arabic. They digitally recorded and transcribed them as notes in English. When stakeholders refused to be recorded, notes were taken during the interviews.

Data analysis was directed by a grounded theory methodology, which is a form of qualitative data analysis that uses a constant comparative method to generate theories of human behavior. The coding of the data used the main themes found in the interview guide as the analytical categories. Other emerging categories were also included in the analysis. Finally, themes uniting the categories were identified.

IBBS Implementation

A two-stage Time Location Sampling (TLS) was adopted as the method of choice to conduct a cross-sectional community-based survey linked with bio-behavioral data generated for MSM and CSWs. The study involved any MSM or CSW who was present at the localities where the study took place and who consented to participate. Eligibility was assessed using a short questionnaire included in the survey instrument.

Four samples were planned for this survey: 1) a sample for Lebanese MSM; 2) a sample for non-Lebanese MSM; 3) a sample for Lebanese CSWs; and 4) a sample for non-Lebanese CSWs.

The total sample size for MSM was 912 (456 Lebanese participants and 456 non-Lebanese participants). The total sample size for CSWs was 536 (268 Lebanese participants and 268 non-Lebanese participants).

TLS, a probability-based method for enrolling members of a Key Population (KP) at times and places where they gather, was used to recruit participants among MSM and CSWs. The sampling frame created during the formative assessment stage consisted of a comprehensive list of venues where the KPs can be found and the days of the week and the hours of the day when they can be found. In the first stage, venues were randomly selected from the sampling frame separately for MSM and CSWs. Appropriate days and time were identified through the verification visits. Selected venues were visited during the specified period, and those attending the venue were systematically recruited.

The data collection tools were compiled using several regional and international questionnaires, reports and articles. They addressed the following topics: background characteristics; marriage and live-in partnerships; sexual history; condoms and lubricants; STIs; knowledge, opinions and attitudes towards HIV/AIDS; stigma, discrimination and violence; alcohol and drug use; and population size estimation.

Interviewers were recruited from the pool of human resources previously trained by the NAP on the national guidelines for HIV counseling, testing, and referral services. Data collection took place at specified days and times. Interviews and counseling were conducted discreetly and in privacy, followed by testing, and if needed service referrals. Once all the study information was explained and the information sheet was provided to the participants, verbal consent was obtained. Data

collection was carried out on electronic tablets by trained fieldworkers using the KoboCollect application.

Following the survey, participants received pre-test risk reduction counseling. A finger-prick rapid test for HIV (Chembio, USA) and a rapid point-of-care test for syphilis (Abon Biopharm, China) followed. Participants with reported positive HIV (reported a positive HIV status) were not tested for HIV. Results disclosure and post-test counseling followed the rapid testing. All those with positive HIV and/or syphilis status were provided with a referral coupon to take a confirmation test and a health brochure including the necessary information regarding referral to care and treatment.

All data were merged, cleaned, and analyzed using Statistical Package for Social Sciences (SPSS) version 24, IBM. Simple descriptive statistical analysis was conducted. Bivariate analysis was also carried out and the results can be found in Appendix K. Data was presented for the Lebanese and non-Lebanese samples separately. Valid percentages were presented taking into account the skip patterns found in the questionnaires.

Population Size Estimation

Population size estimates resulting from an IBBS are inaccurate and disposed to many potential biases due to the absence of a gold standard method[12]. The use of multiple methods strengthens confidence in estimates, provides upper and lower plausibility bounds, and reduces the likelihood that biases of any single method markedly impact results. This study suggested a mixture of methods to produce multiple estimates of the MSM and CSW population sizes in Lebanon: 1) Literature Review; 2) Enumeration; 3) Unique Object Multiplier; and 4) Wisdom of Crowds. Triangulation of results was applied to improve the accuracy of the final estimates of MSM and CSWs.

QUALITY CONTROL AND ASSURANCE

All members engaged in analyzing and handling the formative assessment data were trained to adhere to the whole process of data collection, management, and analysis strategies.

Three trainings (1 in Beirut, 1 in Bekaa and 1 in Tripoli) were implemented for the members of the implementation team in order to standardize data collection amongst them and ensure data quality. Three refresher trainings took place in Beirut and Bekaa attended by selected qualified fieldworkers to refresh them about the data collection procedures and fieldwork tips. The training report, package, agendas and evaluation forms are available in Appendices L to O.

[12] Global Strategic Information. (2014). Toolbox for conducting integrated HIV bio-behavioral surveillance (IBBS) in key populations. University of California, San Francisco.

ETHICAL CONSIDERATIONS

Investigators of this study were formally trained on the protocol and research ethics in this field. Verbal informed consent was obtained from each participant prior to data collection. Protecting participants through anticipating harms and avoiding undue intrusion were secured. Rights to confidentiality and anonymity were maintained through the study. All collected information was de-identified, kept confidential and stored in locked cabinets within CRD premises. Any expense, lost time, and efforts made was acknowledged and, if appropriate, reimbursed in form of an appreciation payment ($10 in cash) and provision of preventive materials, free testing via the referral pathways for laboratory testing, and free services after consented participation.

The review and approval of Transforming Research to Development Institutional Review Board (IRB) was obtained, as per the reference number *TRDIRB091818A*, to ensure that the study was consistent with the ethical policies and considerations related to the protection of the research subjects.

MAIN FINDINGS

Formative Assessment

Description of KIIs

In total, the research team carried out 30 KIIs with stakeholders who provide services to MSM and CSWs in Lebanon.

As displayed in Table 2, the mean age of key informants was 32.4; most participants did not wish to disclose their sex and were not comfortable in sharing their demographic information. The participants also varied in their roles; interviews were carried out with coordinators, psychologists, social workers, and volunteers among others.

Table 2. Description of Key Informant Interviews (N=30).

	N	Mean
**Age**	19	32.4
	**N**	**%**
**Sex**		
Male	9	30.0%
Female	10	33.3%
Did not disclose	11	36.7%
**Role**		
Coordinator	5	16.7%
Psychologist	2	6.7%
Social worker	3	10.0%
Health educator	1	3.3%
Volunteer	2	6.7%
Data entry administrator	1	3.3%
MSM gatekeeper	1	3.3%
Did not disclose	15	50.0%

MSM Community in Lebanon

Key informants provided important insights into the MSM community residing in Lebanon to help implement the IBBS study and formulate the necessary tools. The main themes generated from the carried-out interviews are elaborated below.

1. *Acceptance of the MSM Community in Lebanon*

The key informants unanimously agreed that the MSM community in Lebanon is rejected and marginalized, and faces discrimination from the Lebanese society due to social norms, morals, and religion; such societal beliefs refuse MSM and consider them deviants to what is considered *"normal."* Despite these hurdles, the MSM are gaining momentum and advocating for their sexuality via spreading awareness, and preaching tolerance and acceptance; *"Gladly nowadays they are coming out more, the people are getting to know the MSM community and the associations are working*

on advocating for their rights and their freedom." Key informants also stressed that MSM feel most comfortable when they are around other MSM, allowing them to remove the masks they wear in their daily lives and express their individuality.

2. *MSM Population Characteristics*

Key informants could not describe the MSM population in details, stressing on how they are diverse. Reported age ranged from early teens to above 60 years' old. Key informants stressed how there are MSM who are highly educated "*you can find many doctors, engineers, college professors who are MSM,"* and others who are illiterate. Income also varied, where key informants reported many MSM to belong to a high socio-economic class versus those who are financially very unstable. The majority of MSM were said to reside in urban settings to interact with other MSM for social or sexual purposes without being judged by conservative societies where they were born. The areas where MSM reside the most in Lebanon include Beirut, Hamra, Burj Hammoud, Dora, Dbayeh, Jounieh, and Tripoli.

3. *MSM Community Networking*

The data generated indicates differences in meeting points for MSM depending on whether they have exposed their identity or are still discreet about their gender orientation. The MSM who have embraced their gender identity either encounter other MSM via dating applications such as Grindr (https://www.grindr.com/) and Scruff (https://www.scruff.com/) or meet face-to-face. The meeting locality depends on the purpose; if the purpose is sexual, MSM meet in private places such as apartments, hotels, and chalets, whereas if the purpose is for social interaction, MSM meet in pubs, nightclubs, and gay-friendly restaurants. On the other hand, the discreet MSM meet other MSM via dating applications by creating fake accounts, and if they are to meet in person, they will do so in known private gay-friendly places such as spas, "hammams" (public Turkish baths places), and chalets; *"They meet secretly in discreet gay-friendly places in fear of getting caught or revealed by strangers."*

It is worth noting that the majority of key informants emphasized drug use to be associated with such encounters. Some also raised the concern of the spread of sexually transmitted infections including HIV due to the risky behaviors practiced by these MSM when they meet; *"HIV is becoming more common among MSM in Lebanon, thus the higher risk of others getting infected as well."*

4. *MSM who Carry Out Commercial Sex Work*

MSM who carry out commercial sex work prefer to be named escorts as reported by key informants. The majority were said to be less than 18 years' old, studying at the university, and carrying such work to support themselves financially and become independent. Worth noting that one key informant stressed how these escorts identify themselves as heterosexual, but court MSM for the financial benefits solely; consequently, these escorts keep a professional relationship with other MSM because they consider them potential clients.

CSW Community in Lebanon

Similar to MSM community, key informants provided important insights into the CSW community residing in Lebanon. The main themes generated from the carried-out interviews are elaborated below.

1. *Acceptance of the CSW Community in Lebanon*

Unlike the MSM community, key informants perceived the CSWs to be more accepted and tolerated by the communities in Lebanon; despite this fact, CSWs still face discrimination, stigma, hatred, and social judgment.

2. *CSW Population Characteristics*

Key informants provided a detailed description of the CSW population in Lebanon. The majority were reported to be female and of different nationalities including Lebanese, Russian, Ukrainian, Palestinian, Syrian, and Iraqi. The CSWs' reported age ranged from 15 to 40 years' old. Key informants stressed that all CSWs had attained little or no education, and belonged to a poor socio-economic status. The majority of CSWs were reported to reside in urban settings such as Hamra, Burj Hammoud, Dora, Jounieh, Maameltein, Dbayeh, and Rawche since this would allow easier access to clients and help CSWs avoid shame and scandal they would face if they are to live in the areas where they were born and known by their communities.

Per key informants, CSWs engage in sex work to escape poverty and poor living conditions, cope with the sexual harassment and/or rape they experienced by a member of the family, and since they are widows or single mothers who need to support their families and become financially independent. Key informants also stressed that Syrian CSWs specifically engage in such work to ensure survival and residency within the internal settlements that are ruled by the Shawish, who is the leader of the camp and the most powerful decision maker, labeling the work as *"survival sex"* or *"forced sex."*

3. *CSW Community Networking*

There is minimal or no interaction among the CSW community members, as expressed by key informants; jealousy and competition over clientele rarely allow for social interaction; "*They are in constant jealousy and competition. Who's sexier, who has bigger body parts, who has the biggest clientele.*" And due to this lack of networking, CSWs only meet their clients in settings arranged by their pimps, or on the streets and at restaurants if they work independently. The purpose for these meetings is always to negotiate the pricing of the sexual encounter and the locality preferred by the client for the sex work.

IBBS Results for MSM

Eligibility Screening

All participants were required to complete a screening questionnaire to determine their eligibility. MSM were eligible to participate in the survey if they were 18 years of age or older, were Lebanese or non-Lebanese living in Lebanon, did not participate previously in this round of IBBS, were born biologically males, had anal or oral sex with a man in the last 12 months, and were capable and willing to provide informed consent to participate.

Of those who completed the initial screening questionnaire (N=912), 167 (18.3%) were excluded for not meeting initial eligibility (35 were not eligible and 132 refused to participate); 745 (81.7%) were determined to be eligible.

Of the 745 participants deemed eligible to participate, 376 (50.5%) were Lebanese MSM (L-MSM) and 369 (49.5%) were non-Lebanese MSM (NL-MSM), as indicated in Table 3.

Table 3. Eligibility Screening of MSM (N=912).

	N	%
**Was above 18 years old**		
Yes	905	99.2%
No	7	0.8%
**Participated in the study before**		
Yes	5	0.6%
No	900	99.4%
**Was born biologically male**		
Female	0	0.0%
Male	900	100.0%
**Had anal or oral sex with a man in the last 12 months**		
Yes	887	98.6%
No	13	1.4%
**Was under the influence of alcohol, drugs, or other substance**		
Yes	10	1.1%
No	877	98.9%
**Agreed to participate in the study**		
Yes	745	84.9%
No	132	15.1%
**Nationality**		
Lebanese	376	50.5%
Non-Lebanese	369	49.5%

Background Characteristics

This section discusses the demographic and social characteristics of MSM participants.

As reported in Table 4, the MSM who participated in this survey had a mean age of 28 years, with 30.6% of L-MSM being less than 24 years and 35.0% of NL-MSM being between 24 and 29 years. Little difference was noticed between L-MSM and NL-MSM with regard to their literacy status. University education was observed among more than half (59.3%) of L-MSM, while 24.1% of NL-MSM had achieved primary education levels. In total, two in ten of MSM were unemployed or students (22.6% of L-MSM and 19.2% of NL-MSM). Twenty-one percent of L-MSM ran their own business while 25.5% of NL-MSM were casual laborers (p<0.001). The majority of MSM (97.6% of L-MSM and 94.0% of NL-MSM) had lived in Lebanon for the past 6 months. Moreover, 56.6% of L-MSM and 22.8% of NL-MSM (p<0.001) resided with their parents with the majority living in residences (97.6% of L-MSM and 61.0% of NL-MSM; p<0.001).

Table 4. Background Characteristics of MSM.

	Lebanese (N=376)		Non-Lebanese (N=369)	
	N	%	N	%
**Governorate**				
Baalbak/Hermel	10	2.7%	30	8.1%
Beirut	130	34.6%	101	27.4%
Bekaa	84	22.3%	118	32.0%
Mount Lebanon	90	23.9%	67	18.2%
North	62	16.5%	53	14.4%
	N	Mean ± 95% CI	N	Mean ± 95% CI
**Age (in years)**	376	28.9±0.7	369	28.2±0.7
	N	%	N	%
**Age groups**				
Less than 24 years	88	23.4%	96	26.0%
24 – 29 years	139	37.0%	142	38.5%
30 – 34 years	74	19.7%	67	18.2%
35 + years	75	19.9%	64	17.3%
**Educational level**				
Doesn't know how to read and write	2	0.5%	49	13.3%
Knows how to read and write	11	2.9%	63	17.1%
Primary school	29	7.7%	89	24.1%
Complementary school	33	8.8%	58	15.7%
Secondary school	36	9.6%	35	9.5%
Vocational school	41	10.9%	23	6.2%
University	223	59.3%	52	14.1%
Other	0	0.0%	0	0.0%
Don't know	1	0.3%	0	0.0%
No response	0	0.0%	0	0.0%
**Main occupation**				
Not working/Student	85	22.6%	71	19.2%
Casual laborer	30	8.0%	94	25.5%

Various ^	57	15.2%	125	33.9%
Self-employed	79	21.0%	32	8.7%
Service	71	18.9%	18	4.9%
Other	51	13.6%	28	7.6%
No response	3	0.8%	1	0.3%
**Lived in Lebanon in the past 6 months**				
Yes	367	97.6%	347	94.0%
No	9	2.4%	22	6.0%
No response	0	0.0%	0	0.0%
**People with whom the participant stays**				
Alone	68	18.1%	59	16.0%
With spouse/Live-in partner	38	10.1%	67	18.2%
With parents	213	56.6%	84	22.8%
With other relatives	12	3.2%	40	10.8%
With employer	1	0.3%	12	3.3%
With co-worker/students	5	1.3%	50	13.6%
With male/female friend	36	9.6%	55	14.9%
Other	2	0.5%	1	0.3%
No response	1	0.3%	1	0.3%
**Housing**				
Residence	367	97.6%	225	61.0%
Hotel/Hostel	1	0.3%	15	4.1%
Informal settlement	1	0.3%	85	23.0%
Collective shelter	4	1.1%	29	7.9%
Other public spaces	3	0.8%	13	3.5%
Other	0	0.0%	1	0.3%
No response	0	0.0%	1	0.3%
**Perception of socio-economic status**				
Very poor	11	2.9%	74	20.1%
Poor	41	10.9%	182	49.3%
Moderate	190	50.5%	88	23.8%
Good	96	25.5%	20	5.4%
Very good	36	9.6%	5	1.4%
Don't know	1	0.3%	0	0.0%
No response	1	0.3%	0	0.0%

^ Domestic servant, agricultural labor, skilled/ semi-skilled laborer in manufacturing/ processing industry, cultivator, petty business/ small shop owner, truck drivers/ cleaners, local transport worker

Sexual History and Practices

This section deals with the sexual behavior of MSM. It focuses particularly on risky sexual behavior, type and number of partners.

Self-categorization of MSM

The usual classification of MSM is largely based on their perceived sexual roles. The study participants were asked how they would like to be identified on the basis of their sexual orientation/behavior. In response, 70.2% of L-MSM and 52.6% of NL-MSM ($p<0.001$) said they

regarded themselves as men who have sex with men, while 25.8% of L-MSM and 41.7% of NL-MSM (p<0.001) preferred to be referred to as bisexual, as displayed in Table 5.

Respondents were also asked how they would identify from a gender perspective; the majority of MSM (86.7% of L-MSM and 77.2% of NL-MSM; p<0.001) identified as males, while around 8% (8.5% of L-MSM and 8.7% of NL-MSM) identified as women.

Seventy-two percent (71.5%) of L-MSM and 61.8% of NL-MSM were single (p=0.005). Seven percent (7.2%) of L-MSM and 24.9% of NL-MSM were married at the time of the assessment (p<0.001). While 8.2% of L-MSM and 3.0% of NL-MSM (p=0.002) were living with a partner without being married, and 12.5% of L-MSM and 6.8% of NL-MSM (p=0.008) had stable partners but were not living together, as depicted in Figure 1.

Table 5. Self-categorization of MSM.

	Lebanese (N=376)		Non-Lebanese (N=369)	
	**N**	**%**	**N**	**%**
**Self-identification on the basis of sexual orientation/behavior**				
Man who has sex with men	264	70.2%	194	52.6%
Bisexual	97	25.8%	154	41.7%
Other	6	1.6%	3	0.8%
Don't know	9	2.4%	13	3.5%
No response	0	0.0%	5	1.4%
**Self-identification on the basis of gender**				
Man	326	86.7%	285	77.2%
Woman	32	8.5%	32	8.7%
Other	4	1.1%	3	0.8%
Don't know	13	3.5%	43	11.7%
No response	1	0.3%	6	1.6%

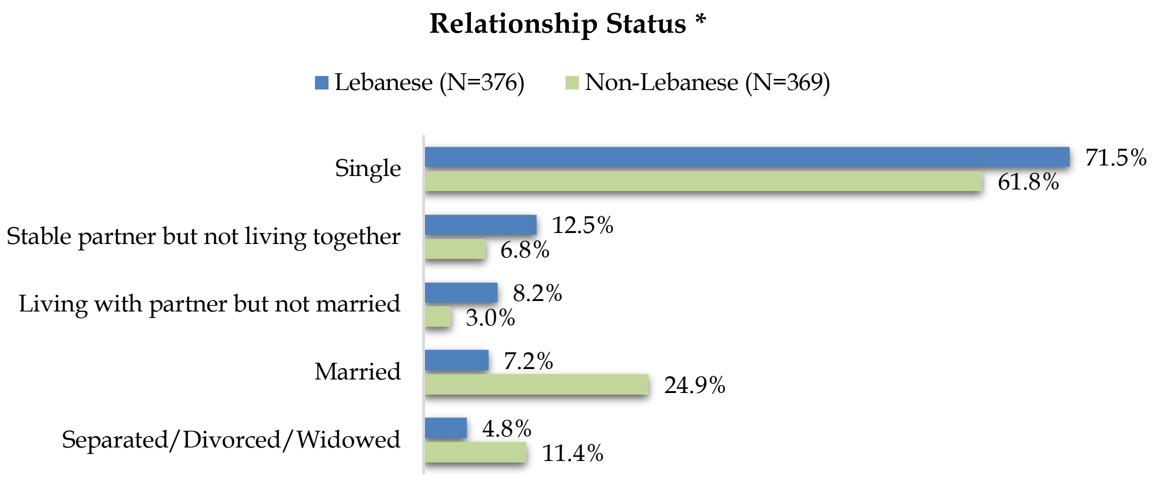

* Answers might not add up to 100 due to multiple possible answers.

Figure 1. Relationship Status of MSM, by Nationality.

Sexual Behavior, Type and Number of Partners

Less than one third (11.7% of L-MSM and 35.8% of NL-MSM; p<0.001) of MSM reported ever being married to a female partner, as described in Table 6.

While all of the MSM participants had had anal or oral sex with a male partner in the past year, more than half of them had sexual contact with female partners too. A larger proportion of NL-MSM (59.6%) than L-MSM (51.1%) (p=0.019) have had sexual contact with female partner in the past year.

On average, the first sexual experience of the MSM participants with a female partner was at the age of 19. L-MSM and NL-MSM had the same age at the time of this experience. Moreover, L-MSM had 1.9 female sex partners in the past 12 months, while NL-MSM had 2.5 female sex partners in the past year.

Table 6. Sexual History and Practices of MSM with Females Partners.

	Lebanese (N=376)		Non-Lebanese (N=369)	
	N	%	N	%
**Ever been married to a female partner**				
Yes	44	11.7%	132	35.8%
No	332	88.3%	235	63.7%
No response	0	0.0%	2	0.5%
**Currently married or living with a female sexual partner**				
Currently married, living with female spouse	20	5.3%	55	14.9%
Currently married, living with other female sexual partner	2	0.5%	14	3.8%
Currently married, not living with spouse or any other female sexual partner	5	1.3%	26	7.0%
Not married, living with female sexual partner	13	3.5%	28	7.6%
Not married, not living with female sexual partner	318	84.6%	221	59.9%
No response	18	4.8%	25	6.8%
**Sexual intercourse with a female partner**				
Yes	192	51.1%	220	59.6%
No	180	47.9%	144	39.0%
Don't know	0	0.0%	0	0.0%
No response	4	1.1%	5	1.4%
	**N**	**Mean ± 95% CI**	**N**	**Mean ± 95% CI**
**Age at first sexual experience with a female partner**	184	19.6±0.6	191	19.3±0.5
**Number of female partners with whom the participant had sexual intercourse in the last 12 months**	179	1.9±0.9	173	2.5±0.5

On average, the first sexual experience of the MSM participants with any male partner was at the age of 16. L-MSM and NL-MSM had the same age at the time of this experience, as illustrated in

Table 7. More than one third (38.6% of L-MSM and 32.2% of NL-MSM) of MSM reported their friend as being their first male partner, as shown in Figure 2.

Anal sex with commercial male partners was less common than with non-commercial partners in the past year (27.9% vs. 81.4% for L-MSM and 35.0% vs. 62.9% for NL-MSM). However, in the past month, anal sex with commercial male partners was as common as with non-commercial partners (64.8% vs. 71.2% for L-MSM and 74.4% vs. 76.7% for NL-MSM).

Moreover, L-MSM had less male commercial partners than NL-MSM in the past month (1.0 for L-MSM and 2.9 for NL-MSM) and week (1.6 for L-MSM and 3.7 for NL-MSM) whereas they had more male non-commercial partners at both time intervals (2.3 for L-MSM and 1.7 for NL-MSM in the past month; 1.0 for L-MSM and 0.9 for NL-MSM in the past week).

The mean number of commercial partners with whom the L-MSM had oral and anal sex was lower than that of non-commercial ones in the last month (2.0 vs. 3.2 and 2.2 vs. 3.7, respectively) whereas the mean number of commercial partners with whom the NL-MSM had oral and anal sex was higher than that of non-commercial ones in the last week (1.3 vs. 0.9 and 1.3 vs. 0.9, respectively).

L-MSM reported a mean number higher than NL-MSM for both types of partners with whom they had oral and anal sex, except in the last week, where sex with commercial partners was more reported among NL-MSM.

Table 7. Sexual History and Practices of MSM with Male Partners.

	Lebanese (N=376)		Non-Lebanese (N=369)	
	**N**	**%**	**N**	**%**
**Type of sexual experience ***				
Manual	228	60.6%	253	68.6%
Oral	209	55.6%	174	47.2%
Anal	144	38.3%	188	50.9%
No response	11	2.9%	15	4.1%
	**N**	**Mean ± 95% CI**	**N**	**Mean ± 95% CI**
**Age at first sexual experience with any male partner**	364	16.1±0.5	325	16.7±0.5
**Age of first sexual male partner**	321	21.7±0.9	219	24.5±1.3
**Number of male commercial partners the participant had in the last one month**	329	1.0±0.6	286	2.9±1.0
**Number of male commercial partners the participant had in the last seven days**	80	1.6±0.4	151	3.7±0.8
**Number of male non-commercial partners the participant had in the last one month**	320	2.3±0.5	222	1.7±0.2
**Number of male non-commercial partners the participant had in the last seven days**	245	1.0±0.1	159	0.9±0.1

Male Commercial Partners				
	N	%	N	%
**Anal sex with male commercial partner in the last 12 months**				
Yes	105	27.9%	129	35.0%
No	268	71.3%	221	59.9%
No response	3	0.8%	19	5.1%
**Anal sex with male commercial partner(s) in the last one month**				
Yes	68	64.8%	96	74.4%
No	34	32.4%	27	20.9%
No response	3	2.9%	6	4.7%
	**N**	**Mean ± 95% CI**	**N**	**Mean ± 95% CI**
**Age at first sexual intercourse with any male commercial partner**	86	21.8±1.4	85	21.1±1.2
**Number of male commercial partners with whom the participant had oral sex in the last one month**	86	2.0±0.9	75	1.9±0.5
**Number of male commercial partners with whom the participant had oral sex in the last seven days**	63	1.0±0.3	67	1.3±0.3
**Number of male commercial partners with whom the participant had anal sex in the last one month**	58	2.2±0.7	66	2.1±0.5
**Number of male commercial partners with whom the participant had anal sex in the last seven days**	59	1.3±0.4	71	1.3±0.3
**Male Non-Commercial Partners**				
	**N**	**%**	**N**	**%**
**Anal sex with male non-commercial partner in the last 12 months**				
Yes	306	81.4%	232	62.9%
No	63	16.8%	119	32.2%
No response	7	1.9%	18	4.9%
**Anal sex with male non-commercial partner(s) in the last one month**				
Yes	218	71.2%	178	76.7%
No	86	28.1%	49	21.1%
No response	2	0.7%	5	2.2%
	**N**	**Mean ± 95% CI**	**N**	**Mean ± 95% CI**
**Age at first sexual intercourse with any male non-commercial partner**	294	16.9±0.5	180	18.0±0.8
**Number of male non-commercial partners with whom the participant had oral sex in the last one month**	282	3.2±1.0	170	1.9±0.3
**Number of male non- commercial partners with whom the participant had oral sex in the last seven days**	233	1.4±0.6	145	0.9±0.2

**Number of male non- commercial partners with whom the participant had anal sex in the last one month**	200	3.7±1.6	144	2.2±0.2
**Number of male non-commercial partners with whom the participant had anal sex in the last seven days**	202	1.3±0.5	142	0.9±0.1

* Answers might not add up to 100 due to multiple possible answers.

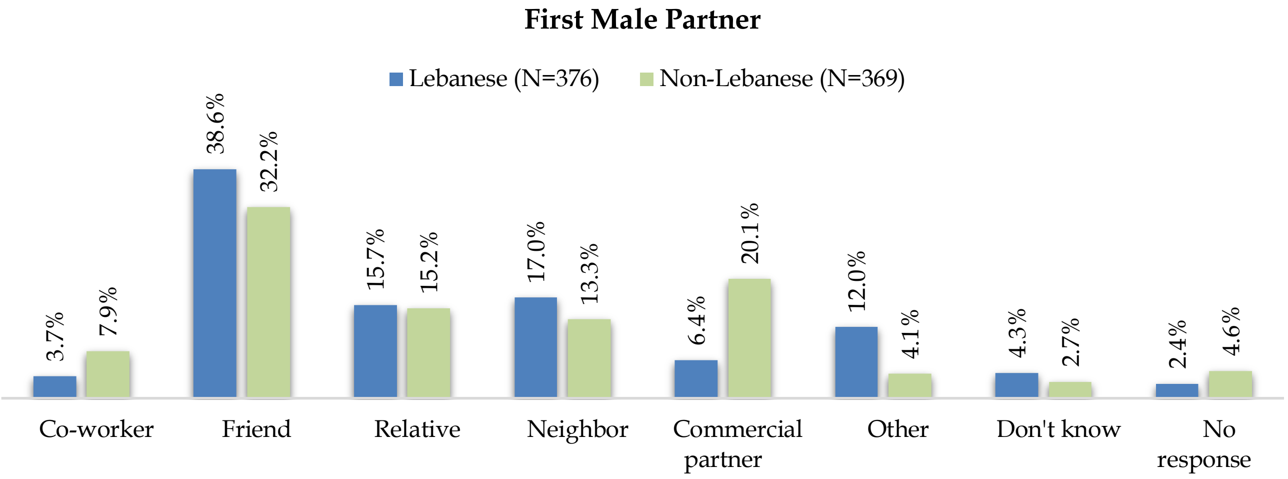

Figure 2. First Male Partner of MSM, by Nationality.

Condoms and Lubricants

This section focuses particularly on MSM's access to and use of condoms and lubricants in different sexual acts.

Nearly all participants had heard of male condoms (93.7% of L-MSM and 92.1% of NL-MSM) and knew a source from where they could get one (97.2% of L-MSM and 86.0% of NL-MSM; p<0.001). The reported sources included pharmacies (96.6% of L-MSM and 90.4% of NL-MSM; p=0.001), shops (21.8% of L-MSM and 8.6% of NL-MSM; p<0.001), non-governmental organizations (NGOs) (29.5% of L-MSM and 12.6% of NL-MSM; p<0.001) and markets (21.0% of L-MSM and 7.6% of NL-MSM; p<0.001), among others.

Table 8 shows condom use during the last sex act with either a female, male commercial or male non-commercial partner. More L-MSM (48.6%) than NL-MSM (38.9%) had used condom in their last sexual contact with a female partner. Almost half of L-MSM (48.5%) and 42.7% of NL-MSM did not use condoms during the last anal sex encounter with a male commercial partner. The majority of MSM (61.9% of L-MSM and 51.7% of NL-MSM) had used a condom the last time they had anal sex with a male non-commercial partner. The main causes of non-utilization varied between both

groups and were mostly attributed to not liking it, perceiving it as unnecessary and/or not available, or objection of commercial partner, as seen in Figure 3 and Figure 4.

MSM were asked about the consistent condom use with male partners. Overall consistent condom use was the highest with male non-commercial partners among both L-MSM (78.0%) and NL-MSM (71.9%).

Table 8. Access to Male Condoms for MSM.

	Lebanese (N=376)		Non-Lebanese (N=369)	
	N	%	N	%
**Ever heard of a male condom**				
Yes	192	93.7%	211	92.1%
No	10	4.9%	10	4.4%
Don't know	1	0.5%	6	2.6%
No response	2	1.0%	2	0.9%
**Use during sex**				
Yes	169	88.0%	160	75.8%
No	23	12.0%	48	22.7%
Don't know	0	0.0%	1	0.5%
No response	0	0.0%	2	0.9%
**Knowing any place or person from where/whom male condoms can be obtained**				
Yes	353	97.2%	302	86.0%
No	9	2.5%	41	11.7%
No response	1	0.3%	8	2.3%
**Places or persons from where/whom male condoms can be obtained ***				
Shop	77	21.8%	26	8.6%
Pharmacy	341	96.6%	273	90.4%
Market	74	21.0%	23	7.6%
Clinic	46	13.0%	15	5.0%
Hospital	22	6.2%	8	2.6%
Family planning center	12	3.4%	15	5.0%
Bar/Guest house/Hotel	20	5.7%	31	10.3%
Peer educator	16	4.5%	20	6.6%
Friend	59	16.7%	48	15.9%
Non-governmental organization	104	29.5%	38	12.6%
National AIDS Control Program	19	5.4%	28	9.3%
Other	4	1.1%	2	0.7%
Don't know	1	0.3%	0	0.0%
No response	0	0.0%	0	0.0%
**Use with Female Partners**				
**Use during last sexual intercourse with a female partner in the last 12 months**				
Yes	53	48.6%	70	38.9%
No	46	42.2%	86	47.8%
Don't know	3	2.8%	17	9.4%
No response	7	6.4%	7	3.9%

**Frequency of use with all female partners in the last 12 months**				
Always	34	31.2%	36	20.0%
Not always	39	35.8%	79	43.9%
Never	29	26.6%	48	26.7%
Don't know	1	0.9%	12	6.7%
No response	6	5.5%	5	2.8%
**Use with Male Commercial Partners**				
**Use during last anal sex with a male commercial partner**				
Yes	28	41.2%	34	35.4%
No	33	48.5%	41	42.7%
Don't remember	5	7.4%	20	20.8%
No response	2	2.9%	1	1.0%
**The person who suggested condom use that time**				
Yourself	13	46.4%	13	38.2%
The commercial partner	3	10.7%	10	29.4%
Joint decision	11	39.3%	11	32.4%
No response	1	3.6%	0	0.0%
**Condom is generally used with the male commercial partners**				
Yes	41	60.3%	62	64.6%
No	22	32.4%	23	24.0%
No response	5	7.4%	11	11.5%
**Frequency of use with all male commercial partners in the last 12 months**				
Always	16	23.5%	17	17.7%
Not always	34	50.0%	54	56.3%
Never	15	22.1%	17	17.7%
Don't know	2	2.9%	4	4.2%
No response	1	1.5%	4	4.2%
**Use with Male Non-Commercial Partners**				
**Use during last anal sex with a male non-commercial partner**				
Yes	135	61.9%	92	51.7%
No	76	34.9%	56	31.5%
Don't remember	6	2.8%	28	15.7%
No response	1	0.5%	2	1.1%
**The person who suggested condom use that time**				
Yourself	57	42.2%	36	39.1%
The non-commercial partner	13	9.6%	15	16.3%
Joint decision	65	48.1%	41	44.6%
No response	0	0.0%	0	0.0%
**Condom is generally used with the male non-commercial partners**				
Yes	170	78.0%	128	71.9%
No	37	17.0%	33	18.5%
No response	11	5.0%	17	9.6%
**Frequency of use with all male non-commercial partners in the last 12 months**				

Always	93	42.7%	65	36.5%
Not always	100	45.9%	90	50.6%
Never	19	8.7%	16	9.0%
Don't know	3	1.4%	5	2.8%
No response	3	1.4%	2	1.1%

* Answers might not add up to 100 due to multiple possible answers.

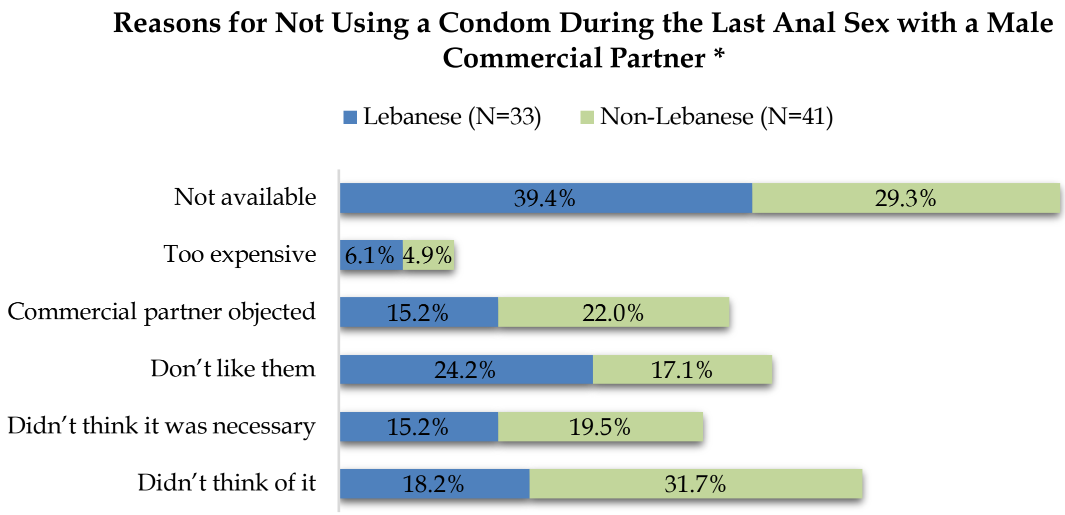

* Answers might not add up to 100 due to multiple possible answers.

Figure 3. Reasons for Not Using a Condom During the Last Anal Sex with a Male Commercial Partner for MSM, by Nationality.

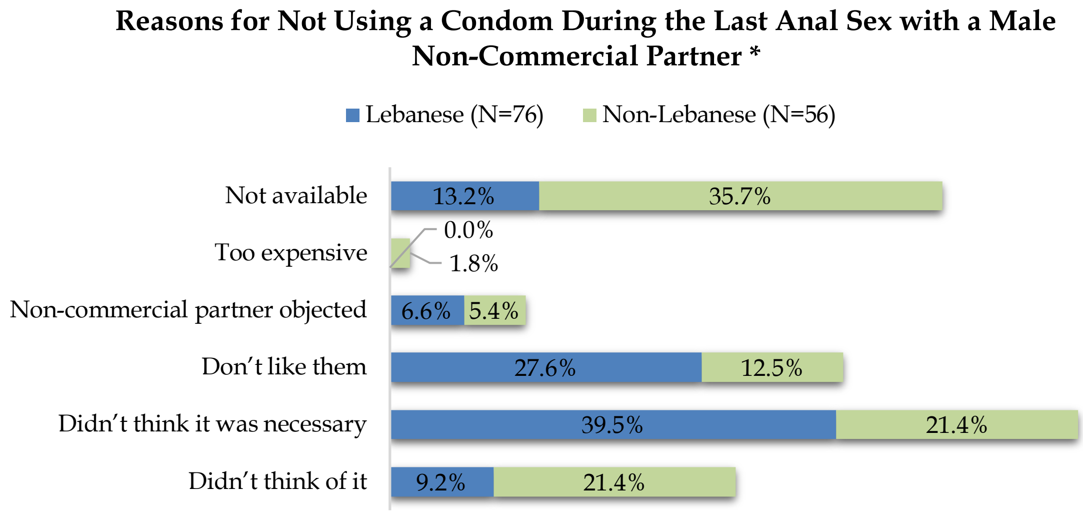

* Answers might not add up to 100 due to multiple possible answers.

Figure 4. Reasons for Not Using a Condom During the Last Anal Sex with a Male Non-Commercial Partner for MSM, by Nationality.

As exhibited in Table 9, more L-MSM (52.4%) than NL-MSM (23.6%) (p<0.001) had heard about the female condom. Of those, 13% (8.1% of L-MSM and 26.4% of NL-MSM; p<0.001) had ever used it. All L-MSM (100.0%) and 87.0% of NL-MSM who used a female condom knew sources from where they could obtain one which consisted mainly of pharmacies (100.0% of L-MSM and 95.0% of NL-MSM).

Table 9. Access to Female Condoms for MSM.

	Lebanese (N=376)		Non-Lebanese (N=369)	
	N	%	N	%
**Ever heard of a female condom**				
Yes	197	52.4%	87	23.6%
No	149	39.6%	200	54.2%
Don't know	23	6.1%	74	20.1%
No response	7	1.9%	8	2.2%
**Use during sex**				
Yes	16	8.1%	23	26.4%
No	177	89.8%	63	72.4%
Don't know	1	0.5%	0	0.0%
No response	3	1.5%	1	1.1%
**Knowing any place or person from where/whom female condoms can be obtained**				
Yes	16	100.0%	20	87.0%
No	0	0.0%	3	13.0%
No response	0	0.0%	0	0.0%
**Places or persons from where/whom female condoms can be obtained** *				
Shop	0	0.0%	1	5.0%
Pharmacy	16	100.0%	19	95.0%
Market	0	0.0%	0	0.0%
Clinic	0	0.0%	0	0.0%
Hospital	0	0.0%	1	5.0%
Family planning center	0	0.0%	2	10.0%
Bar/Guest house/Hotel	0	0.0%	2	10.0%
Peer educator	0	0.0%	1	5.0%
Friend	0	0.0%	2	10.0%
Non-governmental organization	0	0.0%	2	10.0%
National AIDS Control Program	1	6.3%	3	15.0%
Other	0	0.0%	0	0.0%
Don't know	0	0.0%	0	0.0%
No response	0	0.0%	0	0.0%

* Answers might not add up to 100 due to multiple possible answers.

Use of lubricants during anal sexual intercourse was common among MSM (82.2% of L-MSM and 71.3% of NL-MSM; p<0.001), as shown in Table 10. Commonly used lubricants included Aqualube (37.9% of L-MSM and 22.1% of NL-MSM; p<0.001), Vaseline (32.7% of L-MSM and 48.3% of NL-MSM; p<0.001), and K-Y Jelly (35.6% of L-MSM and 26.2% of NL-MSM; p=0.016).

Half of those who did not use lubricant stated they did not like them (50.0% of L-MSM and 49.0% of NL-MSM). Objection of the partners and fear to use them were other obstacles mentioned by the respondents, as seen in Figure 5.

Nearly all participants knew places from where they could access lubricants (98.7% of L-MSM and 93.5% of NL-MSM; p=0.001). These sources consisted of pharmacies (94.1% of L-MSM and 85.8% of NL-MSM; p=0.001), shops (29.8% of L-MSM and 33.7% of NL-MSM) and markets (20.3% of L-MSM and 24.4% of NL-MSM), among others.

Table 10. Access to Lubricants for MSM.

	Lebanese (N=376)		Non-Lebanese (N=369)	
	N	%	N	%
**Use during anal intercourse with men**				
No anal sex with men	7	1.9%	4	1.1%
Yes	309	82.2%	263	71.3%
No	52	13.8%	93	25.2%
Don't know	3	0.8%	4	1.1%
No response	5	1.3%	5	1.4%
**Commonly used lubricant(s) ***				
Aqualube	117	37.9%	58	22.1%
Vaseline	101	32.7%	127	48.3%
Ky Jelly	110	35.6%	69	26.2%
Hand lotion	30	9.7%	52	19.8%
Vaginal gel	7	2.3%	27	10.3%
Baby oil	27	8.7%	41	15.6%
Butter	1	0.3%	25	9.5%
Cooking oil	11	3.6%	40	15.2%
Other	41	13.3%	11	4.2%
Don’t know	8	2.6%	5	1.9%
No response	3	1.0%	2	0.8%
**Frequency of use in the last 12 months**				
Always	151	48.9%	111	42.2%
Not always	147	47.6%	148	56.3%
Never	6	1.9%	2	0.8%
Don't know	4	1.3%	2	0.8%
No response	1	0.3%	0	0.0%
**Knowing any place or person from where/whom lubricants can be obtained**				
Yes	305	98.7%	246	93.5%
No	3	1.0%	16	6.1%
No response	1	0.3%	1	0.4%
**Places or persons from where/whom lubricants can be obtained ***				
Shop	91	29.8%	83	33.7%
Pharmacy	287	94.1%	211	85.8%
Market	62	20.3%	60	24.4%
Clinic	26	8.5%	5	2.0%
Hospital	16	5.2%	0	0.0%

Family planning center	8	2.6%	9	3.7%
Bar/Guest house/Hotel	9	3.0%	10	4.1%
Peer educator	10	3.3%	5	2.0%
Friend	37	12.1%	23	9.3%
Non-governmental organization	39	12.8%	22	8.9%
National AIDS Control Program	12	3.9%	7	2.8%
Other	3	1.0%	1	0.4%
Don't know	2	0.7%	0	0.0%
No response	0	0.0%	0	0.0%

* Answers might not add up to 100 due to multiple possible answers.

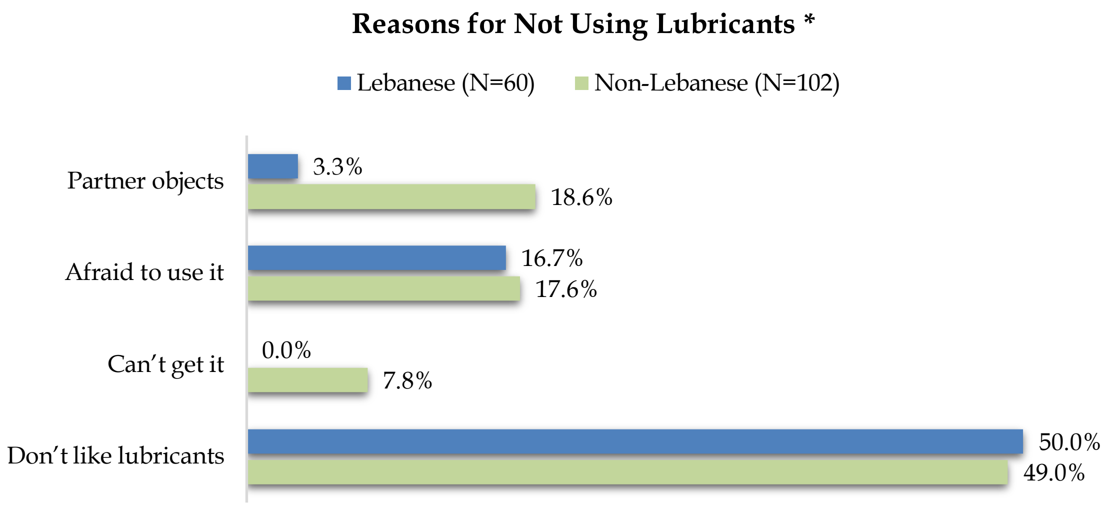

* Answers might not add up to 100 due to multiple possible answers.

Figure 5. Reasons for Not Using Lubricants for MSM, by Nationality.

STIs

This section deals with the level of knowledge among MSM regarding STIs. Along with HIV/AIDS awareness, knowledge about STIs is also crucial to reducing the risk of HIV transmission.

Less than a quarter of the respondents never heard of STIs (9.3% of L-MSM and 23.0% of NL-MSM; $p<0.001$), as summarized in Table 11.

About half of MSM (56.4% of L-MSM and 42.3% of NL-MSM; $p<0.001$) were aware of at least one of the STI symptoms in women. The proportion of respondents reporting to be aware of all symptoms of STIs in women was higher among L-MSM (4.5%) than NL-MSM (0.8%) ($p=0.002$). Most of the L-MSM and NL-MSM (43.3% and 36.8%, respectively) cited genital discharge as an STI symptom in women. The other most commonly cited symptoms were burning during urination, genital ulcers and itching in genital area.

More L-MSM (54.8%) than NL-MSM (39.6%) (p<0.001) were able to recognize STI symptoms in males. For instance, a larger proportion of L-MSM cited genital discharge (63.8% of L-MSM and 54.4% of NL-MSM; p=0.019), burning while urinating (59.9% of L-MSM and 54.4% of NL-MSM) and genital ulcers/sores (55.8% of L-MSM and 40.1% of NL-MSM; p<0.001) as STI symptoms in men. Other symptoms were also cited by more L-MSM than NL-MSM. Only 7.4% of L-MSM and 1.1% of NL-MSM (p<0.001) had good knowledge of all the STI symptoms in men.

The MSM were then asked if they had ever experienced symptoms such as genital discharge and genital ulcers in the past year. Less than one third (16.8% of L-MSM and 33.9% of NL-MSM; p<0.001) of MSM said that they had genital discharge, while 10.1% of L-MSM and 24.1% of NL-MSM (p<0.001) said they had experienced genital ulcers/sores in the past year. A relatively larger proportion of NL-MSM (26.3%) than L-MSM (13.6%) (p<0.001) had anal ulcers/sores in the past year. Likewise, anal discharge was reportedly experienced by more NL-MSM (24.7%) than L-MSM (9.6%) (p<0.001).

As seen in Figure 6 and Figure 7, 26.6% of L-MSM and 43.4% of NL-MSM (p<0.001) had reportedly experienced at least one symptom of STI in the past year while 3.2% of L-MSM and 15.4% of NL-MSM (p<0.001) had experienced all STI symptoms in the past 12 months.

Table 11. STIs among MSM.

	Lebanese (N=376)		Non-Lebanese (N=369)	
	N	%	N	%
**Ever heard of illnesses that can be transmitted through sexual intercourse**				
Yes	337	89.6%	272	73.7%
No	35	9.3%	85	23.0%
No response	4	1.1%	12	3.3%
**Description of any STIs symptoms in women ***				
Abdominal pain	59	17.5%	51	18.8%
Genital discharge	146	43.3%	100	36.8%
Foul smelling discharge	117	34.7%	51	18.8%
Burning pain on urination	115	34.1%	59	21.7%
Genital ulcers/sores	111	32.9%	54	19.9%
Swellings in groin area	48	14.2%	16	5.9%
Itching	110	32.6%	61	22.4%
Other	12	3.6%	7	2.6%
No response	119	35.3%	110	40.4%
**Knowledge of all STIs symptoms in women ^**				
Yes	17	4.5%	3	0.8%
No	359	95.5%	366	99.2%
**Knowledge of at least one STI symptom in women ^**				
Yes	212	56.4%	156	42.3%
No	164	43.6%	213	57.7%
**Description of any STIs symptoms in men ***				
Genital discharge	215	63.8%	148	54.4%
Burning pain on urination	202	59.9%	148	54.4%

Genital ulcers/sores	188	55.8%	109	40.1%
Swellings in groin area	89	26.4%	40	14.7%
Can't retract foreskin	53	15.7%	35	12.9%
Ulcers/sores on the anus	148	43.9%	79	29.0%
Other	27	8.0%	16	5.9%
No response	37	11.0%	43	15.8%
**Knowledge of all STIs symptoms in men ‡**				
Yes	28	7.4%	4	1.1%
No	348	92.6%	365	98.9%
**Knowledge of at least one STI symptom in men ‡**				
Yes	206	54.8%	146	39.6%
No	170	45.2%	223	60.4%
**Genital discharge in the last 12 months**				
Yes	63	16.8%	125	33.9%
No	306	81.4%	221	59.9%
Don't know	6	1.6%	19	5.1%
No response	1	0.3%	4	1.1%
**Genital ulcer in the last 12 months**				
Yes	38	10.1%	89	24.1%
No	329	87.5%	255	69.1%
Don't know	6	1.6%	18	4.9%
No response	3	0.8%	7	1.9%
**Anal ulcer or sore in the last 12 months**				
Yes	51	13.6%	97	26.3%
No	312	83.0%	241	65.3%
Don't know	10	2.7%	24	6.5%
No response	3	0.8%	7	1.9%
**Anal discharge in the last 12 months**				
Yes	36	9.6%	91	24.7%
No	327	87.0%	246	66.7%
Don't know	11	2.9%	28	7.6%
No response	2	0.5%	4	1.1%

* Answers might not add up to 100 due to multiple possible answers.

^ Abdominal pain, genital discharge, foul smelling discharge, burning pain on urination, genital ulcers/sores, swellings in groin area, itching

‡ Genital discharge, burning pain on urination, genital ulcers/ sores, swellings in groin area, can't retract foreskin, ulcers/sores on the anus

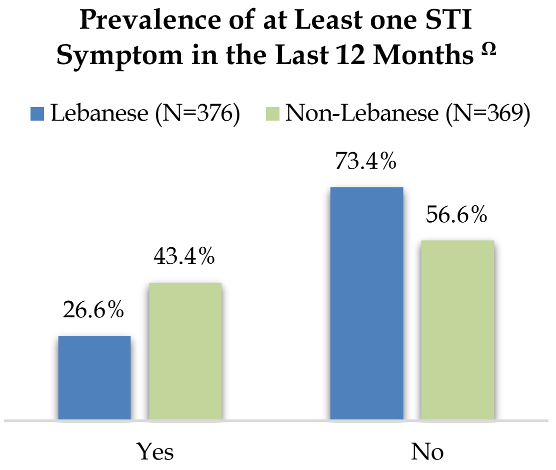

ΩGenital discharge, genital ulcers, anal ulcer, anal discharge in the last 12 months

Figure 6. Prevalence of at Least one STI Symptom in the Last 12 Months among MSM, by Nationality.

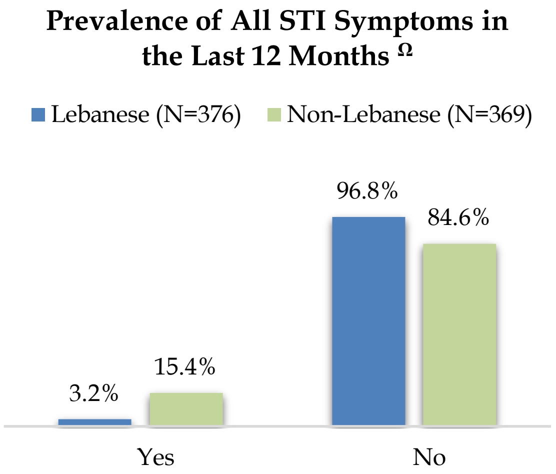

ΩGenital discharge, genital ulcers, anal ulcer, anal discharge in the last 12 months

Figure 7. Prevalence of All STI Symptoms in the Last 12 Months among MSM, by Nationality.

Knowledge, Opinions, and Attitudes towards HIV/AIDS

This section deals with the level of knowledge among MSM regarding HIV/AIDS and the prevalence of HIV among MSM participants.

The majority of participants heard of HIV/AIDS (94.1% of L-MSM and 81.0% of NL-MSM; $p<0.001$). More L-MSM (47.5%) than NL-MSM (23.7%) ($p<0.001$) knew someone living with HIV/AIDS or

who had died of an AIDS related illness. When asked about the type of relationship they shared with such individuals, 70.4% of L-MSM and 57.7%of NL-MSM said they were close friends. On the other hand, 21.3% of L-MSM and 11.3% of NL-MSM of the MSM (50.6%) had heard/seen such people but did not have any relationship with them, as displayed in Table 12.

Respondents were asked about measures to prevent HIV/AIDS. Their understanding of the major HIV/AIDS prevention measures was assessed, including abstinence from sex, being faithful to one sex partner, and consistent condom use. The majority of MSM knew that consistent use of condoms (88.2% of L-MSM and 87.3% of NL-MSM) and being faithful to one partner (80.9% of L-MSM and 79.3% of NL-MSM) will reduce the risk of HIV/AIDS. Six in ten stated that abstinence from sexual contact (63.8% of L-MSM and 63.3% of NL-MSM) was one of the ways of preventing HIV.

Additionally, 74.2% of L-MSM and 54.3% of NL-MSM ($p<0.001$) knew that a healthy-looking person can be infected with HIV, 85.4% of L-MSM and 68.0% NL-MSM ($p<0.001$) knew that HIV cannot be transmitted while sharing meals with an HIV-infected person. However, a relatively smaller proportion of MSM agreed that a person with STIs has an increased chance to be infected with HIV (67.7% of L-MSM and 62.0% of NL-MSM).

The respondents' perception on HIV/AIDS and its different modes of transmission were further tested with the help of certain probing questions. Almost all respondents knew the correct condom usage for each act of anal sex reduces the risk of HIV transmission (86.0% of L-MSM and 83.3% of NL-MSM) and that injecting with a previously used needle (95.5% of L-MSM and 91.0% of NL-MSM; $p=0.020$) will transmit the virus. However, a lower proportion of MSM (46.9% of L-MSM and 54.3% of NL-MSM) believed that a woman with HIV/AIDS can transmit the virus to her new-born child through breastfeeding.

More than three quarters of respondents (81.5% of L-MSM and 75.3% of NL-MSM) were also aware about the risk of pregnant women with HIV/AIDS transmitting the virus to their children in the womb. Among those who were aware of the risk (N=290 L-MSM; N=226 NL-MSM), 61.0% L-MSM and 69.1% NL-MSM knew about antiretroviral therapy.

Furthermore, all participants, except for 1 NL-MSM, knew at least one way by which one can protect oneself against HIV or in which it cannot be transmitted, yet only 8.7% of L-MSM and 6.3% of NL-MSM had complete knowledge about HIV transmission, as seen in Figure 8 and Figure 9.

The availability of and awareness about confidential HIV testing allows people to undertake HIV tests promptly and without the fear of being exposed. More than two thirds of MSM (75.6% of L-MSM and 68.7% of NL-MSM) knew about the existence of a confidential HIV testing facility in their community.

Half of the participants (57.4% of L-MSM and 49.0% of NL-MSM) who reported engaging in commercial anal sex did not discuss STIs and HIV with their partners, while 42.2% of L-MSM and

31.5% of NL-MSM (p=0.028) who reported engaging in non-commercial male-to-male sex discussed STIs and HIV with some of their partners.

Table 12. HIV/AIDS among MSM.

	Lebanese (N=376)		Non-Lebanese (N=369)	
	**N**	**%**	**N**	**%**
**Ever heard of HIV or the disease called AIDS**				
Yes	354	94.1%	299	81.0%
No	20	5.3%	69	18.7%
No response	2	0.5%	1	0.3%
**Knowing anyone who is infected with HIV or who has died of AIDS**				
Yes	169	47.5%	71	23.7%
No	164	46.1%	174	58.0%
Don't know	22	6.2%	51	17.0%
No response	1	0.3%	4	1.3%
**Having a close relative, close friend or partner who is infected with HIV or has died of AIDS ***				
A close relative	12	7.1%	14	19.7%
A close friend	119	70.4%	41	57.7%
A partner	23	13.6%	15	21.1%
No	36	21.3%	8	11.3%
No response	1	0.6	3	4.2
**Knowledge about HIV/AIDS**				
**People can protect themselves from HIV the virus that causes AIDS by using a condom correctly every time they have sex**				
Yes	314	88.2%	262	87.3%
No	32	9.0%	15	5.0%
Don't know	9	2.5%	23	7.7%
No response	1	0.3%	0	0.0%
**People can protect themselves from HIV by avoiding anal sex**				
Yes	193	54.2%	168	56.0%
No	139	39.0%	76	25.3%
Don't know	18	5.1%	49	16.3%
No response	6	1.7%	7	2.3%
**People can protect themselves from HIV by using a condom correctly every time they have anal sex**				
Yes	306	86.0%	250	83.3%
No	37	10.4%	19	6.3%
Don't know	11	3.1%	29	9.7%
No response	2	0.6%	2	0.7%
**A person can get HIV from mosquito bites**				
Yes	55	15.4%	51	17.0%
No	253	71.1%	158	52.7%
Don't know	45	12.6%	84	28.0%
No response	3	0.8%	7	2.3%
**A person can get HIV through saliva**				
Yes	41	11.5%	56	18.7%

No	267	75.0%	163	54.3%
Don't know	42	11.8%	74	24.7%
No response	6	1.7%	7	2.3%
**People can protect themselves from HIV by having one uninfected faithful sex partner**				
Yes	288	80.9%	238	79.3%
No	48	13.5%	35	11.7%
Don't know	16	4.5%	22	7.3%
No response	4	1.1%	5	1.7%
**People can protect themselves from HIV by abstaining from sexual intercourse**				
Yes	227	63.8%	190	63.3%
No	106	29.8%	71	23.7%
Don't know	19	5.3%	36	12.0%
No response	4	1.1%	3	1.0%
**A person can get HIV by sharing a meal with someone who is infected**				
Yes	19	5.3%	32	10.7%
No	304	85.4%	204	68.0%
Don't know	30	8.4%	59	19.7%
No response	3	0.8%	5	1.7%
**A person can get HIV by getting injections with a needle that was already used by someone else**				
Yes	340	95.5%	273	91.0%
No	5	1.4%	5	1.7%
Don't know	10	2.8%	17	5.7%
No response	1	0.3%	5	1.7%
**A healthy-looking person can be infected with HIV, the virus that causes AIDS**				
Yes	264	74.2%	163	54.3%
No	54	15.2%	73	24.3%
Don't know	38	10.7%	57	19.0%
No response	0	0.0%	7	2.3%
**A person with STIs has an increased chance to be infected with HIV**				
Yes	241	67.7%	186	62.0%
No	40	11.2%	24	8.0%
Don't know	74	20.8%	84	28.0%
No response	1	0.3%	6	2.0%
**A pregnant woman infected with HIV/AIDS can transmit the virus to her unborn child**				
Yes	290	81.5%	226	75.3%
No	20	5.6%	15	5.0%
Don't know	46	12.9%	55	18.3%
No response	0	0.0%	4	1.3%
**Actions that pregnant woman can do to reduce the risk of transmission of HIV to her unborn child ***				
Take medication (antiretroviral)	177	61.0%	159	69.1%
Other	12	4.1%	5	2.2%
Don't know	99	34.1%	61	26.5%
No response	5	1.7%	8	3.5%

**A woman with HIV/AIDS can transmit the virus to her newborn child through breastfeeding**				
Yes	167	46.9%	163	54.3%
No	77	21.6%	49	16.3%
Don't know	107	30.1%	84	28.0%
No response	5	1.4%	4	1.3%
**It is possible in the community for someone to get a confidential test to find out if they are infected with HIV**				
Yes	269	75.6%	206	68.7%
No	40	11.2%	32	10.7%
Don't know	45	12.6%	54	18.0%
No response	2	0.6%	8	2.7%
**Discussion of STIs and HIV/AIDS with Partners**				
**Ever discussed HIV/AIDS or STIs with any of the male commercial partners**				
Yes, all	11	16.2%	13	13.5%
Yes, some	15	22.1%	20	20.8%
No, none	39	57.4%	47	49.0%
Don't know	2	2.9%	11	11.5%
No response	1	1.5%	5	5.2%
**Ever discussed HIV/AIDS or STIs with any of the male non-commercial partners**				
Yes, all	38	17.4%	26	14.6%
Yes, some	92	42.2%	56	31.5%
No, none	79	36.2%	73	41.0%
Don't know	2	0.9%	14	7.9%
No response	7	3.2%	9	5.1%

* Answers might not add up to 100 due to multiple possible answers.

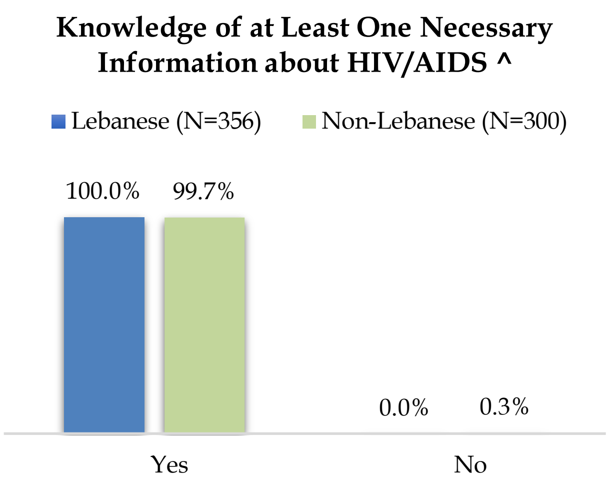

^ Using a condom can protect from HIV/AIDS, avoiding anal sex can protect from HIV/AIDS, having one faithful partner can protect from HIV/AIDS, abstaining from sex can protect from HIV/AIDS, HIV/AIDs cannot be caught from mosquito bites, HIV/AIDS cannot be caught through saliva, sharing a meal with an infected person cannot transmit HIV/AIDS, reusing a needle after someone else can transmit HIV/AIDS, a healthy looking person can be infected with HIV, a person with STIs has an increased chance to be infected with HIV/AIDS, a pregnant woman infected with HIV/AIDS can transmit the virus to her unborn child, a woman with HIV/AIDS can transmit the virus to her newborn child through breastfeeding

Figure 8. Knowledge of at Least One Necessary Information about HIV/AIDS among MSM, by Nationality.

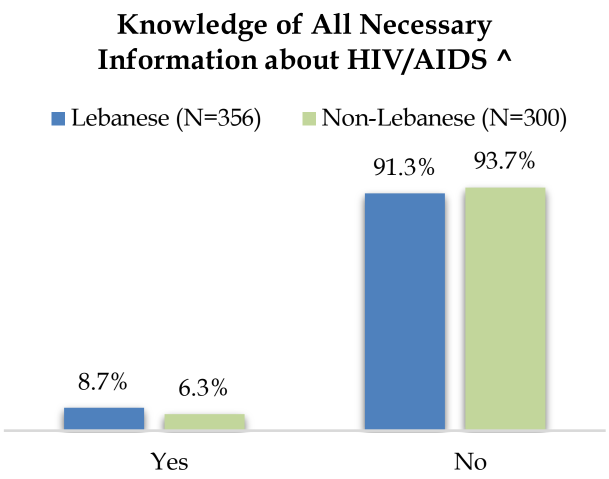

^ Using a condom can protect from HIV/AIDS, avoiding anal sex can protect from HIV/AIDS, having one faithful partner can protect from HIV/AIDS, abstaining from sex can protect from HIV/AIDS, HIV/AIDs cannot be caught from mosquito bites, HIV/AIDS cannot be caught through saliva, sharing a meal with an infected person cannot transmit HIV/AIDS, reusing a needle after someone else can transmit HIV/AIDS, a healthy looking person can be infected with HIV, a person with STIs has an increased chance to be infected with HIV/AIDS, a pregnant woman infected with HIV/AIDS can transmit the virus to her unborn child, a woman with HIV/AIDS can transmit the virus to her newborn child through breastfeeding

Figure 9. Knowledge of All Necessary Information about HIV/AIDS among MSM, by Nationality.

Around sixty percent of respondents reported ever being tested for HIV. This was less common among NL-MSM (48.3%) when compared with L-MSM (69.7%) (p<0.001), as demonstrated in Table 13. HIV testing seems to be by choice rather than being required, mostly among L-MSM (89.1%) (p<0.001). Almost all of those tested received their results of which 16.5% of L-MSM and 7.6% of NL-MSM (p=0.012) were found to be HIV-positive. The majority of MSM (75.8% of L-MSM and 66.2% of NL-MSM; p=0.040) had their most recent HIV test within the last one year. Others were tested more than one year before.

Additionally, more L-MSM (45.7%) than NL-MSM (18.2%) (p<0.001) reported ever being tested for syphilis of which more than 90% (92.4% of L-MSM and 97.0% of NL-MSM) had negative or non-reactive results.

Table 13. Access to HIV and Syphilis Testing among MSM.

	Lebanese (N=376)		Non-Lebanese (N=369)	
	N	%	N	%
**Ever had an HIV test**				
Yes	248	69.7%	145	48.3%
No	105	29.5%	150	50.0%
No response	3	0.8%	5	1.7%
**Most recent HIV test**				
Within the past year	188	75.8%	96	66.2%
Between 1-2 years	24	9.7%	33	22.8%
Between 2-4 years	12	4.8%	7	4.8%
More than 4 years ago	23	9.3%	6	4.1%
Don't know	0	0.0%	0	0.0%
No response	1	0.4%	3	2.1%
**Nature of HIV testing**				
Voluntary	221	89.1%	103	71.0%

Required	26	10.5%	42	29.0%
No response	1	0.4%	0	0.0%
**Result of the last HIV test**				
HIV-negative	200	80.6%	129	89.0%
HIV-positive	41	16.5%	11	7.6%
Indeterminate	2	0.8%	4	2.8%
Didn't get the result	1	0.4%	1	0.7%
Don't know	1	0.4%	0	0.0%
No response	3	1.2%	0	0.0%
**Ever had a syphilis test**				
Yes	172	45.7%	67	18.2%
No	194	51.6%	288	78.0%
No response	10	2.7%	14	3.8%
**Result of the last syphilis test**				
Negative/Non-reactive	159	92.4%	65	97.0%
Positive/Reactive	12	7.0%	1	1.5%
Don't know	0	0.0%	1	1.5%
No response	1	0.6%	0	0.0%

Serological testing for HIV and syphilis was offered to all participants. Participants with reported positive HIV (reported a positive HIV status) were not be tested for HIV. Non-reactive or indeterminate tests were reported as negative, whereas participants showing reactive results were considered as positive.

Not all study participants were tested for HIV; 123 L-MSM and 98 NL-MSM did not seek HIV testing after completing the interview, as presented in Table 14. Of those who did seek testing, there was 4 (1.9%) HIV-positive test results among L-MSM and none among NL-MSM.

As with HIV testing, not all participants completing the interview agreed to be tested for syphilis. Of the 224 L-MSM tests, positive results were found for 8 (3.6%) individuals. Of the 245 NL-MSM who sought syphilis testing, one (0.4%) individual tested positive.

Table 14. Results of the HIV and Syphilis Rapid Tests for MSM.

	Lebanese (N=376)		Non-Lebanese (N=369)	
	**N**	**%**	**N**	**%**
**Agreed to be tested for HIV**				
Yes	212	63.3%	260	72.6%
No	123	36.7%	98	27.4%
**Result of the HIV test**				
HIV-negative	208	98.1%	260	100.0%
HIV-positive	4	1.9%	0	0.0%
Indeterminate	0	0.0%	0	0.0%
**Agreed to be tested for syphilis**				
Yes	224	59.6%	245	66.4%
No	152	40.4%	124	33.6%
**Result of the syphilis test**				

Negative/Non-reactive	216	96.4%	243	99.2%
Positive/Reactive	8	3.6%	1	0.4%
Indeterminate	0	0.0%	1	0.4%

Thus, the prevalence of HIV in the MSM sample was 7.5%. More L-MSM (12.0%) than NL-MSM (3.0) were found to be HIV-positive, as per Figure 10 and Figure 11.

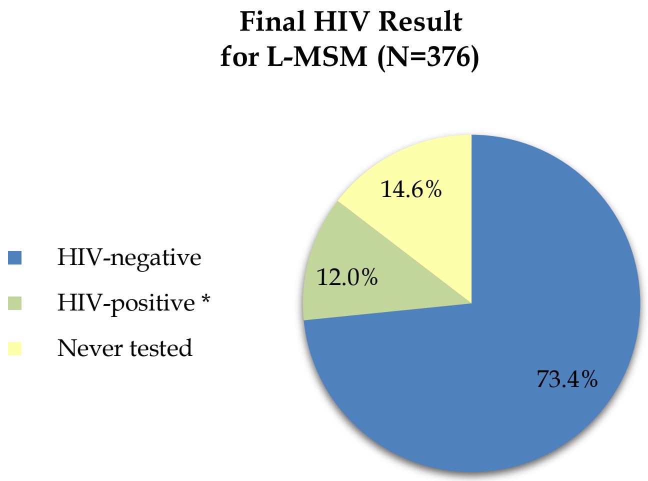

* Includes all those with reported positive HIV status and reactive or indeterminate result to the finger-prick rapid test for HIV.

Figure 10. Final HIV Result for Lebanese MSM.

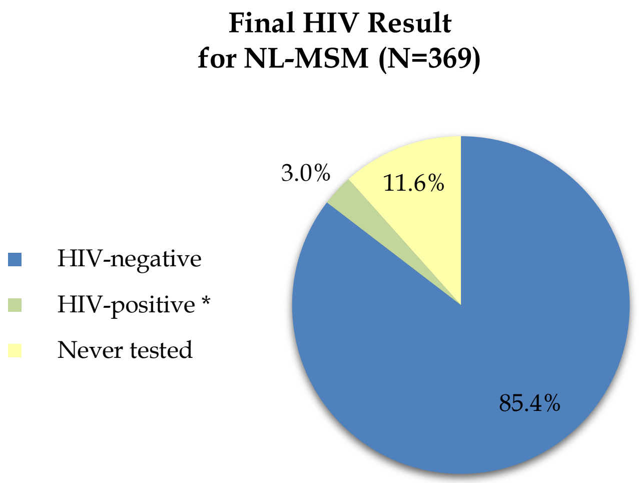

* Includes all those with reported positive HIV status and reactive or indeterminate result to the finger-prick rapid test for HIV.

Figure 11. Final HIV Result for Non-Lebanese MSM.

Stigma, Discrimination and Violence

Many studies have shown that MSM are also subjected to stigma, discrimination and violence because of their sexual orientation. This study tried to find out if any of the study participants were subjected to violence and discrimination in the past year.

The questionnaire included nine items focusing on denial of opportunities to participants – housing, employment, legal, worship, medical care, and food service – and experiences of abuse – verbal, physical, and sexual – in the year prior to interview. Overall, NL-MSM experienced greater degrees of stigma than L-MSM. As shown in Table 15, 36.4% of L-MSM and 45.3% of NL-MSM (p=0.014) were verbally abused, 12.2% of L-MSM and 32.2% of NL-MSM (p<0.001) were physically abused, and 7.2% of L-MSM and 23.6% of NL-MSM (p<0.001) were sexually assaulted.

Moreover, more NL-MSM than L-MSM were denied employment (13.3% of L-MSM and 36.6% of NL-MSM; p<0.001) or housing (9.8% of L-MSM and 35.8% of NL-MSM; p<0.001).

Table 15. Stigma, Discrimination and Violence for MSM.

	Lebanese (N=376)		Non-Lebanese (N=369)	
	N	%	N	%
**Been refused health care in the last 12 months**				
Yes	14	3.7%	33	8.9%
No	342	91.0%	300	81.3%
Don't know	7	1.9%	7	1.9%
No response	13	3.5%	29	7.9%
**Been refused employment in the last 12 months**				
Yes	50	13.3%	135	36.6%
No	307	81.6%	204	55.3%
Don't know	7	1.9%	12	3.3%
No response	12	3.2%	18	4.9%
**Been refused religious service in the last 12 months**				
Yes	34	9.0%	54	14.6%
No	308	81.9%	253	68.6%
Don't know	7	1.9%	27	7.3%
No response	27	7.2%	35	9.5%
**Been refused restaurant/bar service in the last 12 months**				
Yes	44	11.7%	68	18.4%
No	312	83.0%	251	68.0%
Don't know	4	1.1%	16	4.3%
No response	16	4.3%	34	9.2%
**Been refused housing in the last 12 months**				
Yes	37	9.8%	132	35.8%
No	322	85.6%	211	57.2%
Don't know	4	1.1%	9	2.4%
No response	13	3.5%	17	4.6%
**Been refused police assistance in the last 12 months**				
Yes	20	5.3%	51	13.8%
No	338	89.9%	269	72.9%

Don't know	2	0.5%	13	3.5%
No response	16	4.3%	36	9.8%
**Had verbal insults in the last 12 months**				
Yes	137	36.4%	167	45.3%
No	227	60.4%	172	46.6%
Don't know	3	0.8%	5	1.4%
No response	9	2.4%	25	6.8%
**Been hit, kicked or beaten in the last 12 months**				
Yes	46	12.2%	119	32.2%
No	319	84.8%	222	60.2%
Don't know	1	0.3%	2	0.5%
No response	10	2.7%	26	7.0%
**Forced to have sex with someone by sexual assault or rape in the last 12 months**				
Yes	27	7.2%	87	23.6%
No	335	89.1%	251	68.0%
Don't know	1	0.3%	1	0.3%
No response	13	3.5%	30	8.1%

Alcohol and Drug Use

Alcohol consumption and drug use are often associated with risky sexual behavior, leading to HIV and STI transmission. This section attempts to analyze the extent of drug use and alcohol consumption by MSM participants.

Use of Alcohol

Thirty-nine percent (39.1%) of L-MSM and 18.4% of NL-MSM ($p<0.001$) consumed alcohol at least once a week one month prior to the assessment. Less than one third of MSM did not drink 4 weeks before the start of the study: this included a slightly higher proportion of NL-MSM (32.5%) than L-MSM (23.1%) ($p=0.004$).

Moreover, 38.0% of L-MSM and 32.5% of NL-MSM reported consuming sometimes alcoholic drinks before having sex with partners, as exhibited in Table 16. No significant difference was noticed between L-MSM and NL-MSM.

Use and Injection of Drugs

Overall, 18.5% of MSM had used drugs in the past one month. A slightly higher proportion of L-MSM (20.7%) than NL-MSM (16.3%) reported doing so. Party drugs were the most popular drugs, used by 48.8% of L-MSM and 21.9% of NL-MSM ($p=0.001$). Other oral/inhaled drugs used by the respondents are listed in Table 16.

However, only a few respondents (1.6% of L-MSM and 3.8% of NL-MSM) had injected illicit drugs in the past month. MSM mainly injected a combination of cocaine and heroin (37.5% of L-MSM and 44.4% of NL-MSM), while a minority injected cocaine, heroin and buprenorphine.

More than half of MSM (55.1% L-MSM and 59.4% NL-MSM) used drugs up to 3 times during the past month. Similarly, about two thirds of MSM (62.5% of L-MSM and 66.7% of NL-MSM) injected drugs up to 3 times in the past month.

Table 16. Alcohol and Drug Use among MSM.

	Lebanese (N=376)		Non-Lebanese (N=369)	
	N	%	N	%
**Frequency of having drinks containing alcohol in the last one month**				
Every day	30	8.0%	36	9.8%
At least once a week	147	39.1%	68	18.4%
Less than once a week	106	28.2%	108	29.3%
Did not drink in the last 4 weeks	87	23.1%	120	32.5%
Other	0	0.0%	0	0.0%
Don't know	1	0.3%	11	3.0%
No response	5	1.3%	26	7.0%
**Frequency of taking alcoholic drinks before having sex with partners**				
Every time	34	9.0%	49	13.3%
Sometimes	143	38.0%	120	32.5%
Rarely	93	24.7%	101	27.4%
Never	100	26.6%	76	20.6%
Don't know	2	0.5%	2	0.5%
No response	4	1.1%	21	5.7%
**Use or injection of drugs in the last one month**				
Used drugs	78	20.7%	60	16.3%
Injected drugs	6	1.6%	14	3.8%
Used and injected drugs	2	0.5%	4	1.1%
No	285	75.8%	266	72.1%
Don't know	1	0.3%	4	1.1%
No response	4	1.1%	21	5.7%
**Types of drugs used in the last one month ***				
Heroin (not in combination with cocaine)	1	1.3%	7	10.9%
Cocaine (not in combination with heroin)	20	25.0%	7	10.9%
Heroin and cocaine together	5	6.3%	11	17.2%
Crack	2	2.5%	2	3.1%
Buprenorphine	7	8.8%	8	12.5%
Dextropropoxyphene	2	2.5%	4	6.3%
Amphetamines	10	12.5%	3	4.7%
Tranquilizers	6	7.5%	9	14.1%
Barbiturates	1	1.3%	4	6.3%
Party drugs	39	48.8%	14	21.9%
Other	31	38.8%	8	12.5%
Don't know	0	0.0%	1	1.6%
No response	3	3.8%	2	3.1%

**Types of drugs injected in the last one month ***				
Heroin (not in combination with cocaine)	2	25.0%	2	11.1%
Cocaine (not in combination with heroin)	2	25.0%	5	27.8%
Heroin and cocaine together	3	37.5%	8	44.4%
Crack	0	0.0%	0	0.0%
Buprenorphine	2	25.0%	2	11.1%
Dextropropoxyphene	1	12.5%	1	5.6%
Amphetamines	0	0.0%	1	5.6%
Tranquilizers	1	12.5%	0	0.0%
Barbiturates	1	12.5%	0	0.0%
Party drugs	0	0.0%	0	0.0%
Other	1	12.5%	0	0.0%
Don't know	0	0.0%	0	0.0%
No response	0	0.0%	0	0.0%
**Frequency of drug use in the last one month**				
Up to 3 times	43	53.8%	38	59.4%
Once or more weekly	21	26.3%	15	23.4%
Once or more daily	11	13.8%	5	7.8%
Don't know	0	0.0%	3	4.7%
No response	5	6.3%	3	4.7%
**Frequency of drug injection in the last one month**				
Up to 3 times	5	62.5%	12	66.7%
Once or more weekly	3	37.5%	6	33.3%
Once or more daily	0	0.0%	0	0.0%
Don't know	0	0.0%	0	0.0%
No response	0	0.0%	0	0.0%

* Answers might not add up to 100 due to multiple possible answers.

IBBS Results for CSWs

Eligibility Screening

All participants were required to complete a screening questionnaire to determine their eligibility. CSWs were eligible to participate in the survey if they were 18 years of age or older, were Lebanese or non-Lebanese living in Lebanon, did not participate previously in this round of IBBS, had vaginal, anal or oral sex in exchange for money or goods or services in the last 12 months, and were capable and willing to provide informed consent to participate.

Of those who completed the initial screening questionnaire (N=536), 11 (2.1%) were excluded for not meeting initial eligibility (4 were not eligible and 7 refused to participate); 525 (97.9%) were determined to be eligible.

Of the 525 participants deemed eligible to participate, 259 (49.3%) were Lebanese CSWs (L-CSWs) and 266 (50.7%) were non-Lebanese CSWs (NL-CSWs), as indicated in Table 17.

Table 17. Eligibility Screening of CSWs (N=536).

	N	%
**Was above 18 years old**		
Yes	536	100.0%
No	0	0.0%
**Participated in the study before**		
Yes	0	0.0%
No	536	100.0%
**Had vaginal, anal or oral sex in exchange for money or goods or services in the last 12 months**		
Yes	534	99.6%
No	2	0.4%
**Was under the influence of alcohol, drugs, or other substance**		
Yes	2	0.4%
No	532	99.6%
**Agreed to participate in the study**		
Yes	525	98.7%
No	7	1.3%
**Nationality**		
Lebanese	259	49.3%
Non-Lebanese	266	50.7%

Background Characteristics

This section discusses the demographic and social characteristics of CSW participants.

As reported in Table 18, the CSWs who participated in this survey had a mean age of 28 years, with 37.5% of L-CSWs being between 24 and 29 years and 30.8% of NL-CSWs being less than 24 years.

Complementary education was observed among 28.2% of L-CSWs, while 32.3% of NL-CSWs had achieved primary education levels. Additionally, 39.4% of L-CSWs and 29.3% of NL-CSWs were unemployed or students. Nearly all CSWs (100.0% of L-CSWs and 97.4% of NL-CSWs) had lived in Lebanon for the past 6 months. Moreover, more than half of CSWs resided with their parents or spouses with 89.6% of L-CSWs and 48.5% of NL-CSWs living in residences (p<0.001).

Table 18. Background Characteristics of CSWs.

	Lebanese (N=259)		Non-Lebanese (N=266)	
	**N**	**%**	**N**	**%**
**Governorate**				
Baalbak/Hermel	17	6.6%	13	4.9%
Beirut	71	27.4%	75	28.2%
Bekaa	79	30.5%	74	27.8%
Mount Lebanon	50	19.3%	71	26.7%
North	42	16.2%	33	12.4%
	**N**	**Mean ± 95% CI**	**N**	**Mean ± 95% CI**
**Age (in years)**	259	28.1±0.7	266	28.5±0.8
	**N**	**%**	**N**	**%**
**Age groups**				
Less than 24 years	61	23.6%	67	25.2%
24 – 29 years	108	41.7%	87	32.7%
30 – 34 years	51	19.7%	55	20.7%
35 + years	39	15.1%	57	21.4%
**Educational level**				
Doesn't know how to read and write	15	5.8%	53	19.9%
Knows how to read and write	39	15.1%	86	32.3%
Primary school	52	20.1%	86	32.3%
Complementary school	73	28.2%	27	10.2%
Secondary school	37	14.3%	11	4.1%
Vocational school	29	11.2%	2	0.8%
University	13	5.0%	1	0.4%
Other	0	0.0%	0	0.0%
Don't know	0	0.0%	0	0.0%
No response	1	0.4%	0	0.0%
**Main occupation**				
Unemployed/not working/retired student	102	39.4%	78	29.3%
Casual laborer	63	24.3%	68	25.6%
Various ^	60	23.2%	98	36.8%
Self-employed	13	5.0%	6	2.3%
Service	3	1.2%	1	0.4%
Other	18	6.9%	12	4.5%
No response	0	0.0%	3	1.1%
**Lived in Lebanon in the past 6 months**				
Yes	259	100.0%	259	97.4%
No	0	0.0%	7	2.6%
No response	0	0.0%	0	0.0%
**People with whom the participant stays**				

Alone	32	12.4%	22	8.3%
With spouse/Live-in partner	62	23.9%	86	32.3%
With parents	112	43.2%	74	27.8%
With other relatives	24	9.3%	41	15.4%
With employer	5	1.9%	13	4.9%
With co-worker/students	7	2.7%	6	2.3%
With male/female friend	17	6.6%	24	9.0%
Other	0	0.0%	0	0.0%
No response	0	0.0%	0	0.0%
**Housing**				
Residence	232	89.6%	129	48.5%
Hotel/Hostel	2	0.8%	10	3.8%
Informal settlement	18	6.9%	119	44.7%
Collective shelter	6	2.3%	5	1.9%
Other public spaces	1	0.4%	2	0.8%
Other	0	0.0%	0	0.0%
No response	0	0.0%	1	0.4%
**Perception of socio-economic status**				
Very poor	45	17.4%	97	36.5%
Poor	119	45.9%	146	54.9%
Moderate	79	30.5%	22	8.3%
Good	12	4.6%	1	0.4%
Very good	4	1.5%	0	0.0%
Don't know	0	0.0%	0	0.0%
No response	0	0.0%	0	0.0%

^ Domestic servant, agricultural labor, skilled/ semi-skilled laborer in manufacturing/ processing industry, cultivator, petty business/ small shop owner, truck drivers/ cleaners, local transport worker

Sexual History and Practices

This section deals with the sexual behavior of CSWs. It focuses particularly on risky sexual behavior and number of partners.

Self-Categorization

The usual classification of CSWs is largely based on their perceived sexual roles. The study participants were asked how they would like to be identified on the basis of their sexual orientation/behavior. In response, 76.1% of L-CSWs and 80.8% of NL-CSWs regarded themselves as female sex workers, while 1.2% of L-CSWs and 0.8% of NL-CSWs identified themselves as male sex workers, as displayed in Table 19.

Respondents were also asked how they would identify themselves from a gender perspective; the majority of CSWs (93.8% of L-CSWs and 91.4% of NL-CSWs) said they were females. No significant differences were noticed between L-CSWs and NL-CSWs.

Forty-two percent (42.1%) of L-CSWs and 27.8% of NL-CSWs were single (p=0.001). Seventeen percent (17.4%) of L-CSWs and 32.3% of NL-CSWs were married at the time of the assessment

(p<0.001). On the other hand, 30.9% of L-CSWs and 40.2% of NL-CSWs (p=0.026) were separated, divorced or widowed, as shown in Figure 12.

Sexual Behavior and Number of Partners

The majority of CSWs reported ever having vaginal (95.8% of L-CSWs and 98.9% of NL-CSWs; p=0.027) or anal sex (78.8% of L-CSWs and 80.1% of NL-CSWs), as described in Table 19. On average, the first vaginal and anal sexual experiences of CSWs were at the age of 17. NL-CSWs reported being younger than L-CSWs at the time of both experiences (18.5 for L-CSWs and 16.7 for NL-CSWs for first vaginal experience; 18.3 for L-CSWs and 17.2 for NL-CSWs for first anal experience). Moreover, L-CSWs and NL-CSWs had sold sex for the first time at the ages of 19.6 and 18.3, respectively.

CSWs reported different reasons that led to exchange sex for money or goods or services the first time. The most commonly cited reason among both L-CSWs and NL-CSWs was the need of money (72.2% of L-CSWs and 88.0% of NL-CSWs; p<0.001).

L-CSWs reported a mean number lower than NL-CSWs for commercial partners (6.3 for L-CSWs and 8.8 for NL-CSWs) but equal for non-commercial partners (1.1 for L-CSWs and 1.2 for NL-CSWs) with whom they had vaginal, anal or oral sex in the last three months.

The last time CSWs received an amount of money, the majority had performed vaginal sex. A higher proportion of NL-CSWs (74.1%) than L-CSWs (65.3%) (p=0.028) had performed such a sexual act.

Table 19. Sexual History and Practices of CSWs.

	Lebanese (N=259)		Non-Lebanese (N=266)	
	N	%	N	%
**Self-identification on the basis of sexual orientation/behavior**				
Female sex worker	197	76.1%	215	80.8%
Male sex worker	3	1.2%	2	0.8%
Man who have sex with men	1	0.4%	1	0.4%
Other	4	1.5%	2	0.8%
Don't know	37	14.3%	34	12.8%
No response	17	6.6%	12	4.5%
**Self-identification on the basis of gender**				
Man	4	1.5%	5	1.9%
Woman	243	93.8%	243	91.4%
Other	1	0.4%	2	0.8%
Don't know	9	3.5%	13	4.9%
No response	2	0.8%	3	1.1%
**Ever had vaginal sex**				
Yes	248	95.8%	263	98.9%
No	10	3.9%	3	1.1%
Don't know	0	0.0%	0	0.0%

No response	1	0.4%	0	0.0%
**Ever had anal sex**				
Yes	204	78.8%	213	80.1%
No	49	18.9%	52	19.5%
Don't know	2	0.8%	0	0.0%
No response	4	1.5%	1	0.4%
**Reason(s) that led to exchange sex for money or goods or services the first time ***				
Needed money	187	72.2%	234	88.0%
Didn't have any other job	58	22.4%	82	30.8%
Grew up around people who did sex work	39	15.1%	44	16.5%
Was forced/pressured into it	28	10.8%	52	19.5%
Like it/for pleasure	54	20.8%	30	11.3%
Was encouraged by friends	54	20.8%	38	14.3%
It pays well/can make a lot of money	27	10.4%	27	10.2%
Was abandoned by parents/siblings	32	12.4%	37	13.9%
Was abandoned by husband/wife	30	11.6%	26	9.8%
Can have extra money to buy things	56	21.6%	55	20.7%
Orphan	13	5.0%	13	4.9%
Other	0	0.0%	1	0.4%
Don't know	0	0.0%	0	0.0%
No response	3	1.2%	0	0.0%
	**N**	**Mean ± 95% CI**	**N**	**Mean ± 95% CI**
**Age at first vaginal sexual experience**	206	18.5±0.7	243	16.7±0.8
**Age at first anal sexual experience**	175	18.3±0.7	186	17.2±0.8
**Age at first time money or goods or services were received for sex**	211	19.6±0.4	217	18.3±0.4
**Number of non-commercial partners with whom the participant had vaginal, anal or oral sex in the last three months**	156	1.1±0.5	157	1.2±0.5
**Number of commercial partners with whom the participant had vaginal, anal or oral sex in the last three months**	172	6.3±0.8	207	8.8±1.2
**Average amount of money received for a commercial sexual act (in Lebanese pounds)**	234	98,326.9± 9,299.9	246	64,258.1± 8,006.5
	**N**	**%**	**N**	**%**
**Services provided the last time the participant received an amount of money ***				
Oral sex	131	50.6%	150	56.4%
Vaginal sex	169	65.3%	197	74.1%
Anal sex	124	47.9%	145	54.5%
Short term companionship (hours)	138	53.3%	160	60.2%
Long term companionship (nights)	79	30.5%	41	15.4%
Other	5	1.9%	5	1.9%
Don't know	0	0.0%	1	0.4%

No response	1	0.4%	3	1.1%

* Answers might not add up to 100 due to multiple possible answers.

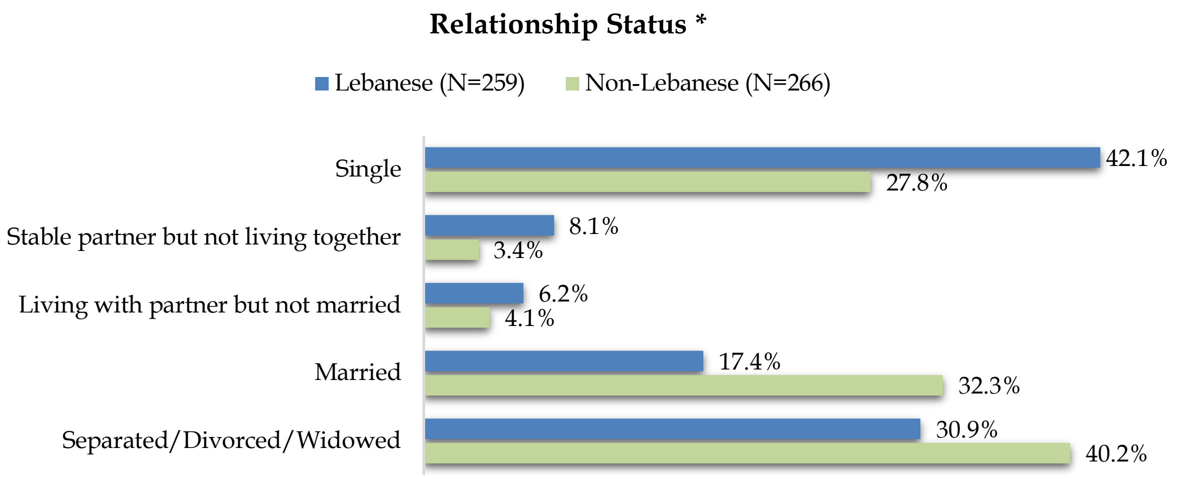

* Answers might not add up to 100 due to multiple possible answers.

Figure 12. Relationship Status of CSWs, by Nationality.

Condoms and Lubricants

This section focuses particularly on CSWs' access to and use of condoms and lubricants in different sexual acts.

Nearly all participants had heard of male condoms (85.2% of L-CSWs and 77.6% of NL-CSWs) and knew a source from where they could get one (94.0% of L-CSWs and 89.5% of NL-CSWs). The reported sources included pharmacies (89.8% of L-CSWs and 90.0% of NL-CSWs), among others.

Table 20 shows condom use during the last sex act with commercial partners. More L-CSWs (79.2%) than NL-CSWs (68.0%) (p=0.004) had used condom in their last vaginal or anal sexual contact with a commercial partner. The main causes of non-utilization were mostly attributed to not liking it, perceiving it as not available, or receiving more money from commercial partners, as seen in Figure 13.

CSWs were asked about the consistent condom use with commercial partners. Overall consistent condom use was the higher among L-CSWs (89.2%) than NL-CSWs (74.1%) (p<0.001).

Table 20. Access to Male Condoms for CSWs.

	Lebanese (N=259)		Non-Lebanese (N=266)	
	**N**	**%**	**N**	**%**
**Ever heard of a male condom**				
Yes	46	85.2%	66	77.6%

No	5	9.3%	7	8.2%
Don't know	2	3.7%	10	11.8%
No response	1	1.9%	2	2.4%
**Use during sex**				
Yes	36	78.3%	46	69.7%
No	9	19.6%	17	25.8%
Don't know	0	0.0%	1	1.5%
No response	1	2.2%	2	3.0%
**Knowing any place or person from where/whom male condoms can be obtained**				
Yes	236	94.0%	221	89.5%
No	13	5.2%	22	8.9%
No response	2	0.8%	4	1.6%
**Places or persons from where/whom male condoms can be obtained ***				
Shop	7	3.0%	6	2.7%
Pharmacy	212	89.8%	199	90.0%
Market	2	0.8%	5	2.3%
Clinic	6	2.5%	3	1.4%
Hospital	8	3.4%	5	2.3%
Family planning center	16	6.8%	7	3.2%
Bar/Guest house/Hotel	45	19.1%	38	17.2%
Peer educator	24	10.2%	36	16.3%
Friend	28	11.9%	39	17.6%
Non-governmental organization	18	7.6%	10	4.5%
National AIDS Control Program	22	9.3%	22	10.0%
Other	0	0.0%	0	0.0%
Don't know	0	0.0%	0	0.0%
No response	0	0.0%	0	0.0%
**Use with Commercial Partners**				
**Use during last vaginal or anal sex with a commercial partner**				
Yes	205	79.2%	181	68.0%
No	44	17.0%	61	22.9%
Don't remember	10	3.9%	16	6.0%
No response	0	0.0%	8	3.0%
**The person who suggested condom use that time**				
Yourself	63	30.7%	78	43.1%
The commercial partner	10	4.9%	13	7.2%
Joint decision	127	62.0%	69	38.1%
No response	5	2.4%	21	11.6%
**Condom is generally used with the commercial partners**				
Yes	231	89.2%	197	74.1%
No	25	9.7%	50	18.8%
No response	3	1.2%	19	7.1%
**Frequency of use with all commercial partners in the last 12 months**				
Every time	100	38.6%	85	32.0%
Almost every time	96	37.1%	79	29.7%

Sometimes	44	17.0%	67	25.2%
Never	12	4.6%	22	8.3%
Don't know	4	1.5%	9	3.4%
No response	3	1.2%	4	1.5%

* Answers might not add up to 100 due to multiple possible answers.

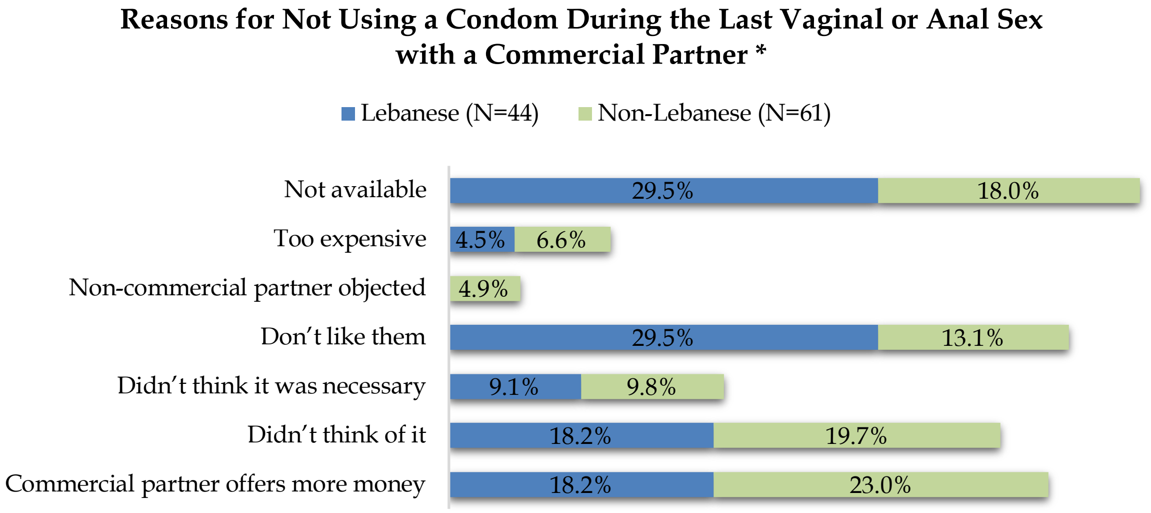

* Answers might not add up to 100 due to multiple possible answers.

Figure 13. Reasons for Not Using a Condom During the Last Vaginal or Anal Sex with a Commercial Partner for CSWs, by Nationality.

As exhibited in Table 21, more L-CSWs (53.3%) than NL-CSWs (44.7%) had heard about the female condom. Of those, 55% (63.8% of L-CSWs and 46.2% of NL-CSWs; p=0.005) had ever used it. Nearly all L-CSWs (95.5%) and NL-CSWs (96.4%) who used a female condom knew sources from where they could obtain one which consisted mainly of pharmacies (90.5% of L-CSWs and 90.6% of NL-CSWs).

Table 21. Access to Female Condoms for CSWs.

	Lebanese (N=259)		Non-Lebanese (N=266)	
	**N**	**%**	**N**	**%**
**Ever heard of a female condom**				
Yes	138	53.3%	119	44.7%
No	87	33.6%	106	39.8%
Don't know	32	12.4%	35	13.2%
No response	2	0.8%	6	2.3%
**Use during sex**				
Yes	88	63.8%	55	46.2%
No	49	35.5%	64	53.8%
Don't know	1	0.7%	0	0.0%
No response	0	0.0%	0	0.0%
**Knowing any place or person from where/whom female condoms can be obtained**				

Yes	84	95.5%	53	96.4%
No	4	4.5%	2	3.6%
No response	0	0.0%	0	0.0%
**Places or persons from where/whom female condoms can be obtained** *				
Shop	0	0.0%	1	1.9%
Pharmacy	76	90.5%	48	90.6%
Market	1	1.2%	0	0.0%
Clinic	0	0.0%	2	3.8%
Hospital	0	0.0%	2	3.8%
Family planning center	0	0.0%	0	0.0%
Bar/Guest house/Hotel	10	11.9%	3	5.7%
Peer educator	5	6.0%	5	9.4%
Friend	8	9.5%	7	13.2%
Non-governmental organization	0	0.0%	0	0.0%
National AIDS Control Program	0	0.0%	0	0.0%
Other	0	0.0%	0	0.0%
Don't know	0	0.0%	0	0.0%
No response	0	0.0%	0	0.0%

* Answers might not add up to 100 due to multiple possible answers.

Use of lubricants during anal sexual intercourse was more common among L-CSWs (70.3%) than NL-CSWs (57.9%) (p-0.003), as shown in Table 22. Commonly used lubricants included Vaseline (78.0% of L-CSWs and 77.3% of NL=CSWs), and K-Y Jelly (25.8% of L-CSWs and 8.4% of NL-CSWs; p<0.001).

The majority of those who did not use lubricant stated they did not like them (46.2% of L-CSWs and 33.7% of NL-CSWs). Objection of the partners and fear to use them were other obstacles mentioned by the respondents, as seen in Figure 14.

Nearly all participants knew places from where they could access lubricants (95.6% of L-CSWs and 92.2% of NL-CSWs). These sources consisted of pharmacies (90.2% of L-CSWs and 88.7% of NL-CSWs) and shops (12.1% of L-CSWs and 14.1% of NL-CSWs), among others.

Table 22. Access to Lubricants for CSWs.

	Lebanese (N=259)		Non-Lebanese (N=266)	
	**N**	**%**	**N**	**%**
**Use during anal intercourse with partners**				
No anal sex with partners	49	18.9%	52	19.5%
Yes	167	64.5%	144	54.1%
No	41	15.8%	65	24.4%
Don't know	0	0.0%	2	0.8%
No response	2	0.8%	3	1.1%
**Commonly used lubricant(s)** *				
Aqualube	26	15.6%	17	11.8%
Vaseline	134	80.2%	115	79.9%
Ky Jelly	42	25.1%	13	9.0%

Hand lotion	30	18.0%	23	16.0%
Vaginal gel	25	15.0%	18	12.5%
Baby oil	15	9.0%	24	16.7%
Butter	1	0.6%	5	3.5%
Cooking oil	5	3.0%	5	3.5%
Other	0	0.0%	0	0.0%
Don’t know	0	0.0%	0	0.0%
No response	1	0.6%	0	0.0%
**Frequency of use in the last 12 months**				
Always	54	32.3%	38	26.4%
Not always	110	65.9%	97	67.4%
Never	3	1.8%	1	0.7%
Don't know	0	0.0%	8	5.6%
No response	0	0.0%	0	0.0%
**Knowing any place or person from where/whom lubricants can be obtained**				
Yes	159	95.2%	133	92.4%
No	8	4.8%	10	6.9%
No response	0	0.0%	1	0.7%
**Places or persons from where/whom lubricants can be obtained ***				
Shop	18	11.3%	20	15.0%
Pharmacy	145	91.2%	118	88.7%
Market	3	1.9%	18	13.5%
Clinic	0	0.0%	2	1.5%
Hospital	1	0.6%	1	0.8%
Family planning center	2	1.3%	0	0.0%
Bar/Guest house/Hotel	13	8.2%	12	9.0%
Peer educator	12	7.5%	8	6.0%
Friend	16	10.1%	11	8.3%
Non-governmental organization	4	2.5%	0	0.0%
National AIDS Control Program	1	0.6%	0	0.0%
Other	0	0.0%	0	0.0%
Don't know	0	0.0%	0	0.0%
No response	0	0.0%	0	0.0%

* Answers might not add up to 100 due to multiple possible answers.

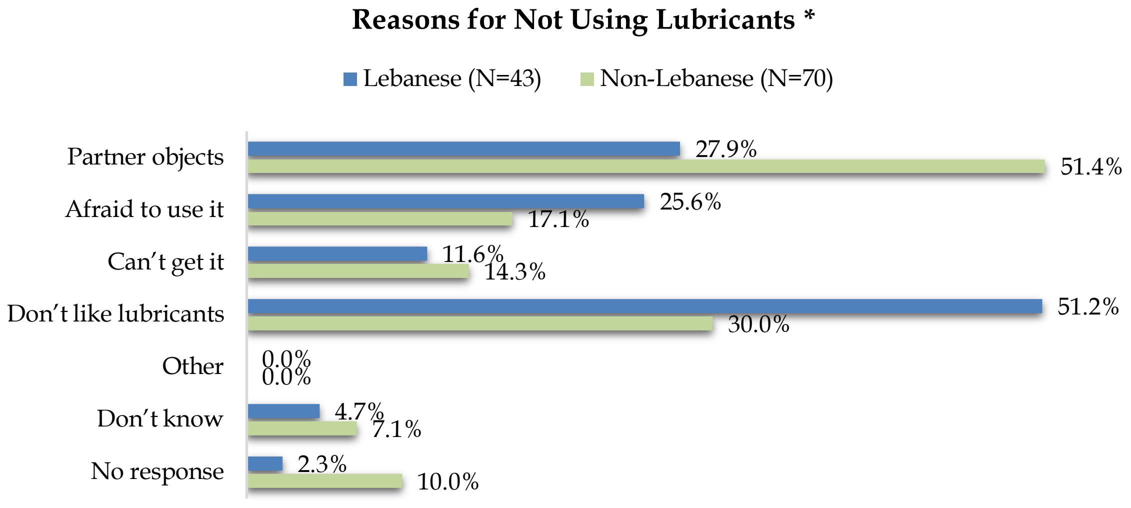

* Answers might not add up to 100 due to multiple possible answers.

Figure 14. Reasons for Not Using Lubricants for CSWs, by Nationality.

STIs

This section deals with the level of knowledge among CSWs regarding STIs. Along with HIV/AIDS awareness, knowledge about STIs is also crucial to reducing the risk of HIV transmission.

More than half (67.2% of L-CSWs and 52.6% of NL-CSWs; p=0.001) of the respondents heard of STIs and were aware of at least one of the STI symptoms in women, as summarized in Table 23. Yet, none of the respondents was aware of all symptoms of STIs in women. Most of the L-CSWs and NL-CSWs (58.0% and 52.9%, respectively) cited genital discharge as an STI symptom in women. The other most commonly cited symptoms were burning during urination, abdominal pain, foul smelling discharge and itching in genital area.

More L-CSWs (41.7%) than NL-CSWs (31.2%) (p=0.012) were able to recognize STI symptoms in males. A larger proportion of L-CSWs cited burning while urinating (55.2% of L-CSWs and 43.6% of NL-CSWs; p=0.041) and genital ulcers/sores (28.7% of L-CSWs and 25.0% of NL-CSWs) as STI symptoms in men. Genital discharge and anal ulcers/sores were cited by more NL-CSWs (45.0% and 17.1%, respectively) than L-CSWs (40.8% and 10.3%, respectively). Only 1 L-CSW had good knowledge of all the STI symptoms in men.

The CSWs were then asked if they had ever experienced symptoms such as genital discharge and genital ulcers in the past year. Less than half (40.9% of L-CSWs and 45.9% of NL-CSWs) of CSWs said that they had genital discharge, while 17.8% of L-CSWs and 28.9% of NL-CSWs (p=0.002) said they had experienced genital ulcers/sores in the past year. A larger proportion of NL-CSWs (25.9%)

than L-CSWs (16.2%) (p=0.006) had anal ulcers/sores in the past year. Likewise, anal discharge was reportedly experienced by more NL-CSWs (25.2%) than L-CSWs (20.8%).

As seen in Figure 15 and Figure 16, 44.4% of L-CSWs and 51.5% of NL-CSWs had reportedly experienced at least one symptom of STI in the past year while 10.0% of L-CSWs and 17.7% of NL-CSWs (p=0.012) had experienced all STI symptoms in the past 12 months.

Table 23. STIs among CSWs.

	Lebanese (N=259)		Non-Lebanese (N=266)	
	N	%	N	%
**Ever heard of illnesses that can be transmitted through sexual intercourse**				
Yes	174	67.2%	140	52.6%
No	80	30.9%	121	45.5%
No response	5	1.9%	5	1.9%
**Description of any STIs symptoms in women ***				
Abdominal pain	71	40.8%	58	41.4%
Genital discharge	101	58.0%	74	52.9%
Foul smelling discharge	86	49.4%	71	50.7%
Burning pain on urination	59	33.9%	40	28.6%
Genital ulcers/sores	41	23.6%	23	16.4%
Swellings in groin area	15	8.6%	13	9.3%
Itching	48	27.6%	42	30.0%
Other	2	1.1%	0	0.0%
No response	19	10.9%	28	20.0%
**Knowledge of all STIs symptoms in women ^**				
Yes	0	0.0%	0	0.0%
No	259	100.0%	266	100.0%
**Knowledge of at least one STI symptom in women ^**				
Yes	174	67.2%	140	52.6%
No	85	32.8%	126	47.4%
**Description of any STIs symptoms in men ***				
Genital discharge	71	40.8%	63	45.0%
Burning pain on urination	96	55.2%	61	43.6%
Genital ulcers/sores	50	28.7%	35	25.0%
Swellings in groin area	36	20.7%	25	17.9%
Can't retract foreskin	27	15.5%	21	15.0%
Ulcers/sores on the anus	18	10.3%	24	17.1%
Other	1	0.6%	1	0.7%
No response	36	20.7%	44	31.4%
**Knowledge of all STIs symptoms in men ‡**				
Yes	1	0.4%	0	0.0%
No	258	99.6%	266	100.0%
**Knowledge of at least one STI symptom in men ‡**				
Yes	108	41.7%	83	31.2%
No	151	58.3%	183	68.8%
**Genital discharge in the last 12 months**				
Yes	106	40.9%	122	45.9%
No	144	55.6%	136	51.1%

Don't know	5	1.9%	8	3.0%
No response	4	1.5%	0	0.0%
**Genital ulcer in the last 12 months**				
Yes	46	17.8%	77	28.9%
No	193	74.5%	171	64.3%
Don't know	14	5.4%	16	6.0%
No response	6	2.3%	2	0.8%
**Anal ulcer or sore in the last 12 months**				
Yes	42	16.2%	69	25.9%
No	196	75.7%	175	65.8%
Don't know	15	5.8%	17	6.4%
No response	6	2.3%	5	1.9%
**Anal discharge in the last 12 months**				
Yes	54	20.8%	67	25.2%
No	181	69.9%	171	64.3%
Don't know	18	6.9%	23	8.6%
No response	6	2.3%	5	1.9%

* Answers might not add up to 100 due to multiple possible answers.
^ Abdominal pain, genital discharge, foul smelling discharge, burning pain on urination, genital ulcers/sores, swellings in groin area, itching
‡ Genital discharge, burning pain on urination, genital ulcers/ sores, swellings in groin area, can't retract foreskin, ulcers/sores on the anus

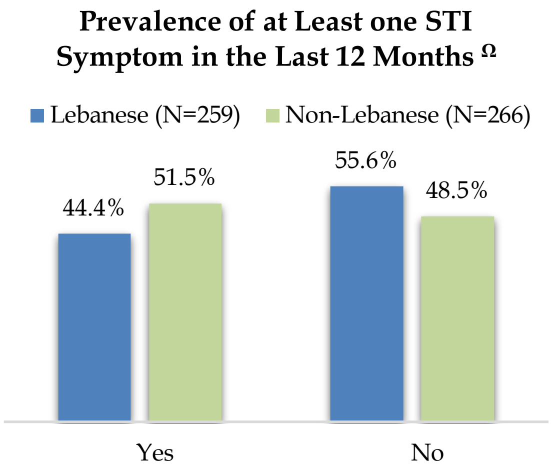

ΩGenital discharge, genital ulcers, anal ulcer, anal discharge in the last 12 months

Figure 15. Prevalence of at Least one STI Symptom in the Last 12 Months among CSWs, by Nationality.

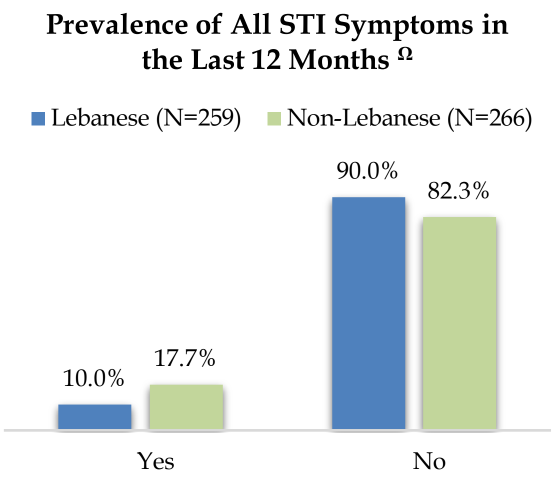

ΩGenital discharge, genital ulcers, anal ulcer, anal discharge in the last 12 months

Figure 16. Prevalence of All STI Symptoms in the Last 12 Months among CSWs, by Nationality.

Knowledge, Opinions, and Attitudes towards HIV/AIDS

This section deals with the level of knowledge among CSWs regarding HIV/AIDS and the prevalence of HIV among CSW participants.

More than 70.0% of participants heard of HIV/AIDS (71.8% of L-CSWs and 75.6% of NL-CSWs; p<0.001). More L-CSWs (18.5%) than NL-CSWs (15.9%) knew someone living with HIV/AIDS or who had died of an AIDS related illness. When asked about the type of relationship they shared with such individuals, 62.9% of L-CSWs and 39.4%of NL-CSWs said they were close friends, as displayed in Table 24.

Respondents were asked about measures to prevent HIV/AIDS. Their understanding of the major HIV/AIDS prevention measures was assessed, including abstinence from sex, being faithful to one sex partner, and consistent condom use. The majority of CSWs knew that consistent use of condoms (85.7% of L-CSWs and 82.2% of NL-CSWs) and being faithful to one partner (81.5% of L-CSWs and 85.6% of NL-CSWs) will reduce the risk of HIV/AIDS. More than half of CSWs stated that abstinence from sexual contact (55.6% of L-CSWs and 63.9% of NL-CSWs) was one of the ways of preventing HIV.

Additionally, 61.9% of L-CSWs and 60.1% of NL-CSWs knew that a person with STIs has an increased chance to be infected with HIV, 55.6% of L-CSWs and 45.7% NL-CSWs (p=0.049) knew that a healthy-looking person can be infected with HIV. However, a relatively smaller proportion of CSWs agreed that HIV cannot be transmitted while sharing meals with an HIV-infected person (49.2% of L-CSWs and 46.2% of NL-CSWs).

The respondents' perception on HIV/AIDS and its different modes of transmission were further tested with the help of certain probing questions. Almost all respondents knew the correct condom usage for each act of anal sex reduces the risk of HIV transmission (86.2% of L-CSWs and 84.6% of NL-CSWs) and that injecting with a previously used needle (84.7% of L-CSWs and 80.8% of NL-CSWs) will transmit the virus. However, a lower proportion of CSWs (55.0% of L-CSWs and 55.8% of NL-CSWs) believed that a woman with HIV/AIDS can transmit the virus to her new-born child through breastfeeding.

Also, 65.1% of L-CSWs and 73.6% of NL-CSWs were also aware about the risk of pregnant women with HIV/AIDS transmitting the virus to their children in the womb. Among those who were aware of the risk (N=123 L-CSWs; N=153 NL-CSWs), 77.4% L-CSWs and 59.9% NL-CSWs knew about antiretroviral therapy.

Furthermore, all participants, except for 1 L-CSW and 2 NL-CSWs, knew at least one way by which one can protect oneself against HIV or in which it cannot be transmitted, yet only 5.3% of L-CSWs and 2.4% of NL-CSWs had complete knowledge about HIV transmission, as seen in Figure 17 and Figure 18.

The availability of and awareness about confidential HIV testing allows people to undertake HIV tests promptly and without the fear of being exposed. More than half of CSWs (59.3% of L-CSWs and 72.6% of NL-CSWs; p=0.005) knew about the existence of a confidential HIV testing facility in their community.

More than half of the participants who reported engaging in commercial sex did not discuss STIs and HIV with their partners. This was mainly noted among NL-CSWs.

Table 24. HIV/AIDS among CSWs.

	Lebanese (N=259)		Non-Lebanese (N=266)	
	N	%	N	%
**Ever heard of HIV or the disease called AIDS**				
Yes	186	71.8%	201	75.6%
No	70	27.0%	58	21.8%
No response	3	1.2%	7	2.6%
**Knowing anyone who is infected with HIV or who has died of AIDS**				
Yes	35	18.5%	33	15.9%
No	114	60.3%	135	64.9%
Don't know	38	20.1%	39	18.8%
No response	2	1.1%	1	0.5%
**Having a close relative, close friend or partner who is infected with HIV or has died of AIDS ***				
A close relative	2	5.7%	8	24.2%
A close friend	22	62.9%	13	39.4%
A partner	5	14.3%	11	33.3%
No	9	25.7%	1	3.0%

No response	0	0.0%	1	3.0%
**Knowledge about HIV/AIDS**				
**People can protect themselves from HIV the virus that causes AIDS by using a condom correctly every time they have sex**				
Yes	162	85.7%	171	82.2%
No	19	10.1%	9	4.3%
Don't know	5	2.6%	24	11.5%
No response	3	1.6%	4	1.9%
**People can protect themselves from HIV by avoiding anal sex**				
Yes	127	67.2%	138	66.3%
No	39	20.6%	29	13.9%
Don't know	20	10.6%	35	16.8%
No response	3	1.6%	6	2.9%
**People can protect themselves from HIV by using a condom correctly every time they have anal sex**				
Yes	163	86.2%	176	84.6%
No	13	6.9%	6	2.9%
Don't know	13	6.9%	23	11.1%
No response	0	0.0%	3	1.4%
**A person can get HIV from mosquito bites**				
Yes	23	12.2%	43	20.7%
No	98	51.9%	71	34.1%
Don't know	64	33.9%	87	41.8%
No response	4	2.1%	7	3.4%
**A person can get HIV through saliva**				
Yes	44	23.3%	36	17.3%
No	79	41.8%	53	25.5%
Don't know	61	32.3%	111	53.4%
No response	5	2.6%	8	3.8%
**People can protect themselves from HIV by having one uninfected faithful sex partner**				
Yes	154	81.5%	178	85.6%
No	12	6.3%	4	1.9%
Don't know	19	10.1%	18	8.7%
No response	4	2.1%	8	3.8%
**People can protect themselves from HIV by abstaining from sexual intercourse**				
Yes	105	55.6%	133	63.9%
No	54	28.6%	36	17.3%
Don't know	25	13.2%	32	15.4%
No response	5	2.6%	7	3.4%
**A person can get HIV by sharing a meal with someone who is infected**				
Yes	31	16.4%	19	9.1%
No	93	49.2%	96	46.2%
Don't know	61	32.3%	85	40.9%
No response	4	2.1%	8	3.8%

**A person can get HIV by getting injections with a needle that was already used by someone else**				
Yes	160	84.7%	168	80.8%
No	4	2.1%	5	2.4%
Don't know	24	12.7%	35	16.8%
No response	1	0.5%	0	0.0%
**A healthy-looking person can be infected with HIV, the virus that causes AIDS**				
Yes	105	55.6%	95	45.7%
No	21	11.1%	35	16.8%
Don't know	60	31.7%	78	37.5%
No response	3	1.6%	0	0.0%
**A person with STIs has an increased chance to be infected with HIV**				
Yes	117	61.9%	125	60.1%
No	13	6.9%	5	2.4%
Don't know	58	30.7%	76	36.5%
No response	1	0.5%	2	1.0%
**A pregnant woman infected with HIV/AIDS can transmit the virus to her unborn child**				
Yes	123	65.1%	153	73.6%
No	10	5.3%	8	3.8%
Don't know	55	29.1%	43	20.7%
No response	1	0.5%	4	1.9%
**Actions that pregnant woman can do to reduce the risk of transmission of HIV to her unborn child ***				
Take medication (antiretroviral)	96	77.4%	94	59.9%
Other	3	2.4%	0	0.0%
Don't know	28	22.6%	56	35.7%
No response	0	0.0%	7	4.5%
**A woman with HIV/AIDS can transmit the virus to her newborn child through breastfeeding**				
Yes	104	55.0%	116	55.8%
No	11	5.8%	22	10.6%
Don't know	73	38.6%	70	33.7%
No response	1	0.5%	0	0.0%
**It is possible in the community for someone to get a confidential test to find out if they are infected with HIV**				
Yes	112	59.3%	151	72.6%
No	20	10.6%	14	6.7%
Don't know	54	28.6%	41	19.7%
No response	3	1.6%	2	1.0%
**Discussion of STIs and HIV/AIDS with Partners**				
**Ever discussed HIV/AIDS or STIs with any of the commercial partners**				
Yes, all	41	15.8%	43	16.2%
Yes, some	47	18.1%	23	8.6%
No, none	164	63.3%	189	71.1%
Don't know	6	2.3%	8	3.0%

No response	1	0.4%	3	1.1%

* Answers might not add up to 100 due to multiple possible answers.

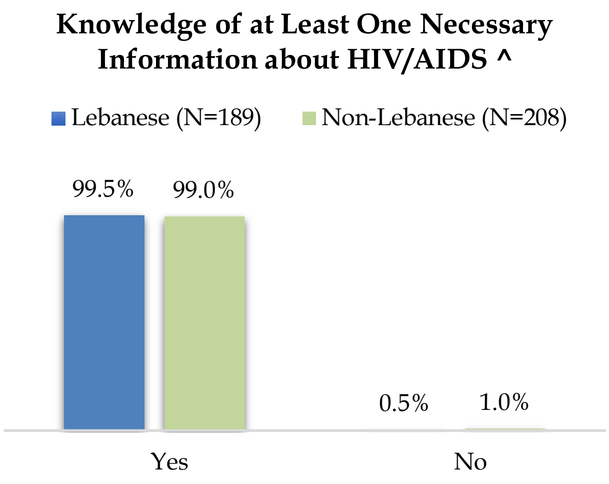

^ Using a condom can protect from HIV/AIDS, avoiding anal sex can protect from HIV/AIDS, having one faithful partner can protect from HIV/AIDS, abstaining from sex can protect from HIV/AIDS, HIV/AIDs cannot be caught from mosquito bites, HIV/AIDS cannot be caught through saliva, sharing a meal with an infected person cannot transmit HIV/AIDS, reusing a needle after someone else can transmit HIV/AIDS, a healthy looking person can be infected with HIV, a person with STIs has an increased chance to be infected with HIV/AIDS, a pregnant woman infected with HIV/AIDS can transmit the virus to her unborn child, a woman with HIV/AIDS can transmit the virus to her newborn child through breastfeeding

Figure 17. Knowledge of at Least One Necessary Information about HIV/AIDS among CSWs, by Nationality.

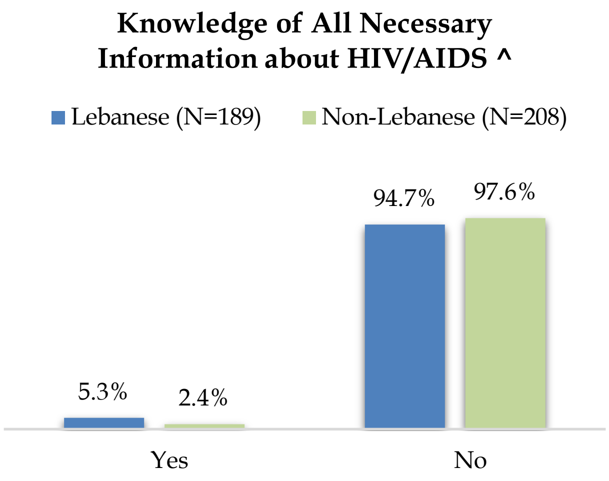

^ Using a condom can protect from HIV/AIDS, avoiding anal sex can protect from HIV/AIDS, having one faithful partner can protect from HIV/AIDS, abstaining from sex can protect from HIV/AIDS, HIV/AIDs cannot be caught from mosquito bites, HIV/AIDS cannot be caught through saliva, sharing a meal with an infected person cannot transmit HIV/AIDS, reusing a needle after someone else can transmit HIV/AIDS, a healthy looking person can be infected with HIV, a person with STIs has an increased chance to be infected with HIV/AIDS, a pregnant woman infected with HIV/AIDS can transmit the virus to her unborn child, a woman with HIV/AIDS can transmit the virus to her newborn child through breastfeeding

Figure 18. Knowledge of All Necessary Information about HIV/AIDS among CSWs, by Nationality.

Around one third of respondents (37.0% of L-CSWs and 32.2% of NL-CSWs) reported ever being tested for HIV, as demonstrated in Table 25. More than half of both L-CSWs (62.9%) and NL-CSWs (50.7%) took up the test voluntarily, while others were asked to test for HIV. Almost all of those tested received their results of which 2.9% of L-CSWs were found to be HIV-positive. The majority

of CSWs (58.6% of L-CSWs and 64.2% of NL-CSWs) had their most recent HIV test within the last one year. Others were tested more than one year before.

Additionally, more L-CSWs (10.4%) than NL-CSWs (5.3%) (p=0.028) reported ever being tested for syphilis of which 96.3% of L-CSWs and 78.6% of NL-CSWs had negative or non-reactive results.

Table 25. Access to HIV and Syphilis Testing among CSWs.

	Lebanese (N=259)		Non-Lebanese (N=266)	
	N	%	N	%
**Ever had an HIV test**				
Yes	70	37.0%	67	32.2%
No	116	61.4%	138	66.3%
No response	3	1.6%	3	1.4%
**Most recent HIV test**				
Within the past year	41	58.6%	43	64.2%
Between 1-2 years	18	25.7%	19	28.4%
Between 2-4 years	6	8.6%	3	4.5%
More than 4 years ago	3	4.3%	2	3.0%
Don't know	2	2.9%	0	0.0%
No response	0	0.0%	0	0.0%
**Nature of HIV testing**				
Voluntary	44	62.9%	34	50.7%
Required	26	37.1%	33	49.3%
No response	0	0.0%	0	0.0%
**Result of the last HIV test**				
HIV-negative	64	91.4%	57	85.1%
HIV-positive	2	2.9%	0	0.0%
Indeterminate	3	4.3%	2	3.0%
Didn't get the result	0	0.0%	6	9.0%
Don't know	0	0.0%	0	0.0%
No response	1	1.4%	2	3.0%
**Ever had a syphilis test**				
Yes	27	10.4%	14	5.3%
No	225	86.9%	242	91.0%
No response	7	2.7%	10	3.8%
**Result of the last syphilis test**				
Negative/Non-reactive	26	96.3%	11	78.6%
Positive/Reactive	1	3.7%	1	7.1%
Don't know	0	0.0%	2	14.3%
No response	0	0.0%	0	0.0%

Serological testing for HIV and syphilis was offered to all participants. Participants with reported positive HIV (reported a positive HIV status) were not be tested for HIV. Non-reactive or indeterminate tests were reported as negative, whereas participants showing reactive results were considered as positive.

Not all study participants were tested for HIV; 63 L-CSWs and 51 NL-CSWs did not seek HIV testing after completing the interview, as presented in Table 26. Of those who did seek testing, none was tested positive.

As with HIV testing, not all participants completing the interview agreed to be tested for syphilis. Of the 171 L-CSW tests, positive results were found for 2 (1.2%) individuals. All 175 NL-CSWs who sought syphilis testing had non-reactive results.

Table 26. Results of the HIV and Syphilis Rapid Tests for CSWs.

	Lebanese (N=259)		Non-Lebanese (N=266)	
	**N**	**%**	**N**	**%**
**Agreed to be tested for HIV**				
Yes	194	75.5%	215	80.8%
No	63	24.5%	51	19.2%
**Result of the HIV test**				
HIV-negative	194	100.0%	215	100.0%
HIV-positive	0	0.0%	0	0.0%
Indeterminate	0	0.0%	0	0.0%
**Agreed to be tested for syphilis**				
Yes	171	66.0%	175	65.8%
No	88	34.0%	91	34.2%
**Result of the syphilis test**				
Negative/Non-reactive	169	98.8%	175	100.0%
Positive/Reactive	2	1.2%	0	0.0%
Indeterminate	0	0.0%	0	0.0%

Thus, the prevalence of HIV in the CSW sample was 0.4%. In fact, only 0.8% of L-CSWs were found to be HIV-positive, as per Figure 19 and Figure 20.

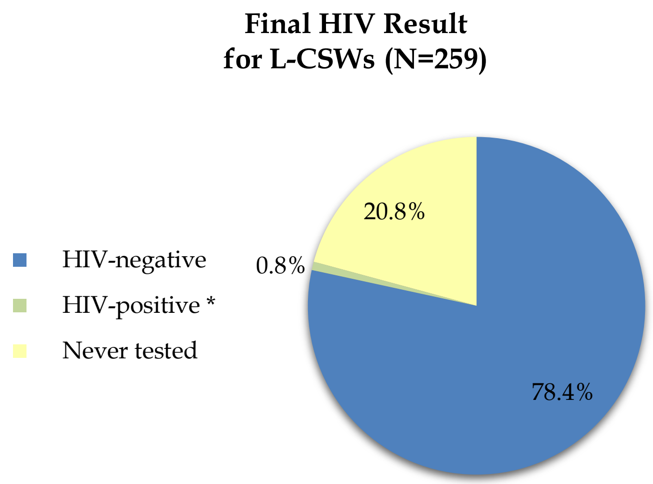

* Includes all those with reported positive HIV status and reactive or indeterminate result to the finger-prick rapid test for HIV.

Figure 19. Final HIV Result for Lebanese CSWs.

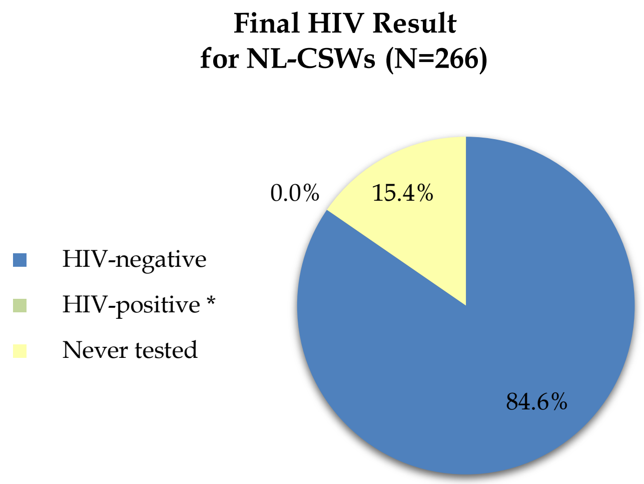

* Includes all those with reported positive HIV status and reactive or indeterminate result to the finger-prick rapid test for HIV.

Figure 20. Final HIV Result for Non-Lebanese CSWs.

Stigma, Discrimination and Violence

Many studies have shown that CSWs are also subjected to stigma, discrimination and violence because of their sexual acts. This study tried to find out if any of the study participants were subjected to violence and discrimination in the past year.

The questionnaire included nine items focusing on denial of opportunities to participants – housing, employment, legal, worship, medical care, and food service – and experiences of abuse – verbal, physical, and sexual – in the year prior to interview. Overall, NL- CSWs experienced slightly greater degrees of stigma than L-CSWs. As shown in Table 27, 29.3% of L-CSWs and 45.5% of NL-CSWs (p<0.001) were verbally abused, 21.6% of L-CSWs and 33.5% of NL-CSWs (p=0.002) were physically abused, and 31.3% of L-CSWs and 34.6% of NL-CSWs were sexually assaulted.

Moreover, more NL-CSWs than L-CSWs were denied employment (35.9% of L-CSWs and 47.0% of NL-CSWs; p=0.010) or housing (30.9% of L-CSWs and 46.6% of NL-CSWs; p<0.001).

Table 27. Stigma, Discrimination and Violence for CSWs.

	Lebanese (N=259)		Non-Lebanese (N=266)	
	N	%	N	%
**Been refused health care in the last 12 months**				
Yes	23	8.9%	22	8.3%
No	216	83.4%	228	85.7%
Don't know	7	2.7%	2	0.8%
No response	13	5.0%	14	5.3%
**Been refused employment in the last 12 months**				
Yes	93	35.9%	125	47.0%

No	154	59.5%	130	48.9%
Don't know	4	1.5%	3	1.1%
No response	8	3.1%	8	3.0%
**Been refused religious service in the last 12 months**				
Yes	36	13.9%	29	10.9%
No	194	74.9%	215	80.8%
Don't know	6	2.3%	4	1.5%
No response	23	8.9%	18	6.8%
**Been refused restaurant/bar service in the last 12 months**				
Yes	41	15.8%	43	16.2%
No	194	74.9%	206	77.4%
Don't know	1	0.4%	1	0.4%
No response	23	8.9%	16	6.0%
**Been refused housing in the last 12 months**				
Yes	80	30.9%	124	46.6%
No	162	62.5%	133	50.0%
Don't know	4	1.5%	0	0.0%
No response	13	5.0%	9	3.4%
**Been refused police assistance in the last 12 months**				
Yes	26	10.0%	27	10.2%
No	208	80.3%	220	82.7%
Don't know	6	2.3%	5	1.9%
No response	19	7.3%	14	5.3%
**Had verbal insults in the last 12 months**				
Yes	76	29.3%	121	45.5%
No	161	62.2%	136	51.1%
Don't know	4	1.5%	0	0.0%
No response	18	6.9%	9	3.4%
**Been hit, kicked or beaten in the last 12 months**				
Yes	56	21.6%	89	33.5%
No	184	71.0%	165	62.0%
Don't know	3	1.2%	1	0.4%
No response	16	6.2%	11	4.1%
**Forced to have sex with someone by sexual assault or rape in the last 12 months**				
Yes	81	31.3%	92	34.6%
No	160	61.8%	154	57.9%
Don't know	1	0.4%	1	0.4%
No response	17	6.6%	19	7.1%

Alcohol and Drug Use

Alcohol consumption and drug use are often associated with risky sexual behavior, leading to HIV and STI transmission. This section attempts to analyze the extent of drug use and alcohol consumption by CSW participants.

Use of Alcohol

More than one third of CSWs (29.0% of L-CSWs and 40.6% of NL-CSWs; $p=0.005$) did not drink alcohol one month prior to the assessment, followed by 27.8% of L-CSWs and 14.3% of NL-CSWs ($p<0.001$) who consumed alcohol less than once a week.

Moreover, 30.5% of L-CSWs and 33.1% of NL-CSWs reported rarely taking alcoholic drinks before having sex with partners, as exhibited in Table 28.

Use and Injection of Drugs

Overall, 14.1% of CSWs had used drugs in the past one month. A slightly higher proportion of L-CSWs (15.4%) than NL-CSWs (12.8%) reported doing so. Tranquilizers were the most popular drugs, used by 48.8% of L-CSWs and 48.6% of NL-CSWs. Other oral/inhaled drugs used by the respondents are listed in Table 28.

However, only a few respondents (1.2% of L-CSWs and 2.3% of NL-CSWs) had injected illicit drugs in the past month. CSWs mainly injected a combination of cocaine and heroin (25.0% of L-CSWs and 42.9% of NL-CSWs), while a minority injected tranquilizers, dextropropoxyphene, party drugs and barbiturates.

More than half of CSWs (56.1% L-CSWs and 65.7% NL-CSWs) used drugs up to 3 times during the past month. Similarly, the majority of CSWs (75.0% of L-CSWs and 85.7% of NL-CSWs) injected drugs up to 3 times in the past month.

Table 28. Alcohol and Drug Use among CSWs.

	Lebanese (N=259)		Non-Lebanese (N=266)	
	N	%	N	%
**Frequency of having drinks containing alcohol in the last one month**				
Every day	23	8.9%	17	6.4%
At least once a week	61	23.6%	25	9.4%
Less than once a week	72	27.8%	38	14.3%
Did not drink in the last 4 weeks	75	29.0%	108	40.6%
Other	0	0.0%	0	0.0%
Don't know	6	2.3%	21	7.9%
No response	22	8.5%	57	21.4%
**Frequency of taking alcoholic drinks before having sex with partners**				
Every time	46	17.8%	26	9.8%
Sometimes	73	28.2%	63	23.7%
Rarely	79	30.5%	88	33.1%
Never	42	16.2%	50	18.8%
Don't know	5	1.9%	6	2.3%
No response	14	5.4%	33	12.4%
**Use or injection of drugs in the last one month**				

Used drugs	40	15.4%	34	12.8%
Injected drugs	3	1.2%	6	2.3%
Used and injected drugs	1	0.4%	1	0.4%
No	196	75.7%	201	75.6%
Don't know	2	0.8%	3	1.1%
No response	17	6.6%	21	7.9%
**Types of drugs used in the last one month ***				
Heroin (not in combination with cocaine)	5	12.2%	1	2.9%
Cocaine (not in combination with heroin)	3	7.3%	0	0.0%
Heroin and cocaine together	7	17.1%	11	31.4%
Crack	0	0.0%	0	0.0%
Buprenorphine	3	7.3%	2	5.7%
Dextropropoxyphene	0	0.0%	0	0.0%
Amphetamines	2	4.9%	1	2.9%
Tranquilizers	20	48.8%	17	48.6%
Barbiturates	3	7.3%	0	0.0%
Party drugs	10	24.4%	8	22.9%
Other	2	4.9%	1	2.9%
Don't know	0	0.0%	3	8.6%
No response	0	0.0%	2	5.7%
**Types of drugs injected in the last one month ***				
Heroin (not in combination with cocaine)	0	0.0%	0	0.0%
Cocaine (not in combination with heroin)	0	0.0%	0	0.0%
Heroin and cocaine together	1	25.0%	3	42.9%
Crack	0	0.0%	0	0.0%
Buprenorphine	0	0.0%	0	0.0%
Dextropropoxyphene	0	0.0%	2	28.6%
Amphetamines	0	0.0%	0	0.0%
Tranquilizers	1	25.0%	2	28.6%
Barbiturates	0	0.0%	1	14.3%
Party drugs	1	25.0%	0	0.0%
Other	0	0.0%	0	0.0%
Don't know	0	0.0%	0	0.0%
No response	1	25.0%	1	14.3%
**Frequency of drug use in the last one month**				
Up to 3 times	23	56.1%	23	65.7%
Once or more weekly	11	26.8%	7	20.0%
Once or more daily	5	12.2%	3	8.6%
Don't know	2	4.9%	0	0.0%
No response	0	0.0%	2	5.7%
**Frequency of drug injection in the last one month**				
Up to 3 times	3	75.0%	6	85.7%
Once or more weekly	0	0.0%	1	14.3%
Once or more daily	1	25.0%	0	0.0%
Don't know	0	0.0%	0	0.0%
No response	0	0.0%	0	0.0%

* Answers might not add up to 100 due to multiple possible answers.

Global AIDS Monitoring Indicators

Knowledge about HIV Prevention

This indicator presents the percentage of people who correctly identify both ways of preventing the sexual transmission of HIV and reject major misconceptions about HIV transmission. It measures the progress towards universal knowledge of the essential facts about HIV transmission. It is constructed from responses to the following set of prompted questions:

1. Can the risk of HIV transmission be reduced by having sex with only one uninfected partner who has no other partners?
2. Can a person reduce the risk of getting HIV by using a condom every time they have sex?
3. Can a healthy-looking person have HIV?
4. Can a person get HIV from mosquito bites?
5. Can a person get HIV by sharing food with someone who is infected?

HIV Testing

This indicator presents the percentage of people who tested for HIV in the past 12 months or who know their current HIV status. It measures the progress in providing HIV testing services to members of key populations.

Condom Use

This indicator presents the percentage of people reporting using a condom with their most recent partner. It measures the progress in preventing exposure to HIV among MSM and CSWs through unprotected sex with partners.

HIV Prevalence

This indicator presents the percentage of people who test positive for HIV. It measures the progress in reducing HIV prevalence among key populations.

Table 29. Global AIDS Monitoring Indicators.

	MSM		CSWs	
	L-MSM (%)	NL-MSM (%)	L-CSWs (%)	NL-CSWs (%)
**Knowledge about HIV prevention**	42.0%	18.0%	15.0%	10.0%
**HIV testing**	58.0%	33.0%	22.0%	20.0%
**Condom use**	45.0%	42.0%	79.0%	68.0%
**HIV prevalence**	12.0%	3.0%	1.0%	0.0%

Population Size Estimation

This study suggested a mixture of methods to produce multiple estimates of the MSM and CSW population sizes in Lebanon:

Literature Review

A literature review leverages existing data to calculate KP size estimates. A literature search was performed to identify published reports and/or peer-reviewed scientific journal articles containing information on size estimations of MSM[13,14,15,16] and CSWs[15,16]. The estimates were presented as the mean numbers.

Enumeration

During the formative phase and mapping exercise, a comprehensive list of venues where the MSM and CSWs gather was compiled. A complete list of venues was created including the venues and the number of persons enumerated in each venue. Two approaches were adopted during the visit of each venue:

- Counted directly the number of the MSM or CSWs present in a venue;
- Asked individuals knowledgeable about the venue about the number of MSM or CSWs present at the venue.

The estimates were presented as the mean responses.

Unique Object Multiplier

A fixed number of a unique bracelet was distributed to eligible MSM and CSWs identified in the venues prior to the IBSS. Participants were then asked whether they were given a unique bracelet. Two parameters were used to estimate the population sizes: the numbers of MSM and CSWs who have received the unique bracelet divided by the proportions of MSM and CSWs in the IBBS that have reported receiving such objects. To strengthen accuracy and recall, the distribution of the bracelets was done shortly before the launch of the IBSS. MSM and CSWs were asked not to give the bracelet to anyone else and to hold on to the object for at least three months. The objects were

---

[13] Baral, S., Turner, R. M., Lyons, C. E., Howell, S., Honermann, B., Garner, A., ... & Millett, G. (2018). Population Size Estimation of Gay and Bisexual Men and Other Men Who Have Sex With Men Using Social Media-Based Platforms. JMIR public health and surveillance, 4(1).
[14] Adam, P. C., de Wit, J. B., Toskin, I., Mathers, B. M., Nashkhoev, M., Zablotska, I., ... & Rugg, D. (2009). Estimating levels of HIV testing, HIV prevention coverage, HIV knowledge, and condom use among men who have sex with men (MSM) in low-income and middle-income countries. JAIDS Journal of Acquired Immune Deficiency Syndromes, 52, S143-S151.
[15] Global, A. I. D. S. (2018). Monitoring 2018: Indicators for monitoring the 2016 United Nations Political Declaration on HIV and AIDS. Geneva: UNAIDS.
[16] UNAIDS. (2017). Country Factsheets - Lebanon | 2017. UNAIDS.

distributed by outreach workers who knew how to find these KPs. The estimates were presented as the mean responses.

Wisdom of Crowds

The wisdom of crowds method considers the perceptions of survey participants to produce an estimate of the number of the KPs in Lebanon. Participants in the IBBS were asked for their best guess on the number of the population size of MSM and CSWs living in Lebanon. The estimates were presented as the median responses.

Triangulation of Results

The numbers generated by the four methods were compiled providing upper and lower acceptability bounds, as shown in Table 30 and Table 31. These numbers were presented and discussed at a national stakeholders meeting.

Estimated Population Size for MSM

Table 30. Population Size Estimates for MSM.

	Population Size Estimates		
	**Lower Bound**	**Point Estimate**	**Upper Bound**
**Literature review ^**	-	16333	-
**Enumeration ^**	17866	19816	21765
**Unique object multiplier ^**	12300	13791	15283
**Wisdom of crowds ‡**	9250	16000	25000

^ Based on mean numbers
‡ Based on median numbers

Estimated Population Size for CSWs

Table 31. Population Size Estimates for CSWs.

	Population Size Estimates		
	**Lower Bound**	**Point Estimate**	**Upper Bound**
**Literature review ^**	-	4210	-
**Enumeration ^**	4080	4467	4855
**Unique object multiplier ^**	3544	3956	4368
**Wisdom of crowds ‡**	1000	4500	6000

^ Based on mean numbers
‡ Based on median numbers

LIMITATIONS OF THE STUDY

The findings of the IBSS should be interpreted in the light of certain limitations.

The qualitative component of the study relied on the perceptions and viewpoints of the participants of the KIIs. Audio taping the interviews posed a challenge considering the sensitive nature of the study and participants' acceptance to recording was respected. When recordings were not available, expressed perceptions and viewpoints of participants were harder to document by the note taker.

The quantitative component of this study relied on self-report, since it is practically impossible to collect data on sexual practices by direct observation. The validity of self-reported data, especially behavioral data such as sexual identity and sexual practices is sometimes questioned, as they are especially prone to social desirability bias (the more stigmatized the behavior, the more likely people are to lie about it) and are considered to be illegal in Lebanon. It is worthy to note that the extent to which people answer similar questions openly and truthfully depends mainly on the setting of the question, assurance of privacy and confidentiality, and the non-judgmental nature of the questions. All these details were taken into account and the interviewers were trained to collect data in an unbiased manner. Moreover, certain practices and behaviors might be subject to recall bias, particularly those requiring recall over 12 months. On the other hand, data collected through the questionnaires were not meant to answer every question on risk behavior among these population groups. They only addressed key behavioral questions and should be considered as one piece of the information needed to assess, advocate for, and develop effective intervention programs. Moreover, logistic regression analysis was seen to have very limited value due to the low prevalence of HIV-positive cases.

As for the PSE, this exercise generally cannot estimate the proportion of MSM and CSWs who are truly hidden and/or who do not even acknowledge that they are MSM or CSWs. These individuals may not be counted in any data source. Furthermore, the study was limited to KPs who are above 18 years and therefore these estimates do not include MSM and CSWs younger than 18. In this sense, there is a strong possibility that our estimates are likely an underestimation of the true MSM and CSWs' population sizes in Lebanon.

Finally, the fact that monetary incentives were given to participants to compensate their time, the study risked recruiting participants disguising as MSM or CSWs. However, the interviewers were well-trained on screening individuals to ensure all participants meet inclusion criteria. Also, the appropriate incentive amount was agreed upon in a way that would not meet the threshold of tempting people to fake eligibility.

RECOMMENDATIONS

The following recommendations are suggested:

Optimize STIs and HIV Knowledge and Testing Services among Key Populations

The greater majority of participants did not have good knowledge about HIV/AIDS transmission based on a composite scale of transmission modes. This provides evidence of the need for increased education about HIV risk and transmission for KPs.

Not knowing signs and symptoms of STI may also result in MSM and CSWs neither recognizing an infection on a sexual partner nor seeking advice and treatment when infected, thus increasing the risk of acquiring or transmitting STIs. Monitoring STIs among MSM and CSWs is essential as these infections are easily transmitted to sexual partners and associated with increased sexual HIV transmission. MSM and CSWs should routinely be screened and, if needed, treated, especially in HIV testing and couseling settings.

Multiple testing strategies targeting key populations should be undertaken by all stakeholders, including outreach testing using peer education approaches, and testing within health facility outpatient and inpatient settings. Mobile outreach STIs and HIV counseling and testing services should also be conducted.

Increase Access to and Use of Condoms among Key Populations

Condom use was not common among all participants, with inconsistencies in use between commercial and non-commercial partners. HIV programmes in Lebanon should consider increasing availability of condoms to target key populations through several strategies, such as the peer education programme, distributing condoms at VCT centres, outpatient and family planning clinics. Distribution of condoms and lubricants is an immediate and effective strategy, although less tailored to all sub-groups of the MSM and CSW populations. Those who do not gather into communities and do not identify or disclose their sexual behaviors will be impossible to reach with an intervention that implies condom/lubricant distribution and counseling. Alternatively, these populations could be targeted with innovative context tailored interventions, for example internet/mobile application based interventions.

Increase Advocacy Efforts and Promote MSM and CSWs' Rights

Efforts should made to increase advocacy addressing stigma and discrimination towards MSM and CSWs to create supportive communities. Media campaigns should be developed to address social norms of MSM and CSWs, stigma and discrimination and to encourage interpersonal and community-level dialogue to support change.

Another recommendation is to build the capacity and conduct awareness sessions on stigma and discrimination against KPs with social workers, health workers, and other concerned people working with MSM and CSWs; this in turn would help increase the number of beneficiaries accessing prevention services.

Implement Future IBBS Research

Future IBBS should be conducted in Lebanon to track the progress of performance on key HIV indicators and establish trends in prevalence for HIV and STIs. Such surveys will allow to measure the success of HIV programmes in Lebanon. It is also recommended that future studies be extended to other areas of Lebanon.

APPENDICES

Appendix A: Key Informant Interview Guide.

Appendix B: Key Informant Interview Informed Consent Form.

Appendix C: IBBS Instrument for MSM.

Appendix D: IBBS Instrument for CSWs.

Appendix E: Results of the Rapid Tests.

Appendix F: IBBS Informed Consent Form for MSM.

Appendix G: IBBS Informed Consent Form for CSWs.

Appendix H: Venue Enumeration Form for Population Size Estimation.

Appendix I: Unique Object Distribution List.

Appendix J: Unique Object Distribution Registration Book.

Appendix K: Results of the Bivariate Analysis

Appendix L: Fieldworkers' Training Report.

Appendix M: Training Package.

Appendix N: Training Agendas.

Appendix O: Training Evaluation Form.
