# Supplementary material for: Progress towards the 95–95–95 targets to end HIV by 2030 in Lebanon, 2023
Source: PLoS One. 2025 Jun 13;20(6):e0321868. doi: 10.1371/journal.pone.0321868 (PMC12165419; doi:10.1371/journal.pone.0321868)
Supplement: S4 File — Lebanon HIV IBBS 2015 final report. (PDF) [file pone.0321868.s004.pdf]

PROJECT CROSSROADS

Size Estimation, Risk Behavior Assessment, and Disease Prevalence among Key Populations in Lebanon

Project CROSSROADS
Size Estimation, Risk Behavior Assessment, and Disease Prevalence in Populations at High Risk for HIV Infection in Lebanon

Prepared for and property of: Middle East and North Africa Harm Reduction Association (MENAHRA), Beirut, Lebanon

Consultants:

Robert Heimer, PhD, Professor[1]
Kaveh Khoshnood, PhD, Associate Professor[1]
Forrest Crawford, PhD, Assistant Professor[1]
Fatma M. Shebl, MD, PhD, Assistant Professor[1]
Russell Barbour, PhD, Assoc. Research Scientist[1]
Danielle Khouri, MPH
Jacques Mokhbat, MD, Professor[2]

1) Yale School of Public Health and the Center for Interdisciplinary Research on AIDS New Haven, CT, USA.
2) Infectious Diseases, Rizk Hospital, Lebanese American University, Beirut, Lebanon.

15 June 2015

Contact Information;
Robert Heimer – robert.heimer@yale.edu
Kaveh Khoshnood – kaveh.khoshnood@yale.edu
Jacques Mokhbat – jacques.mokhbat@lau.edu.lb
Danielle Khouri – danielle.elkhoury@gmail.com

TABLE OF CONTENTS

Introduction 3

Part 1: Population Size Estimation 7

- Part 1A: Population Size Estimation for People Who Inject Drugs (PWID) 7
  - Population Size of PWID
  - Sociodemographics of the PWID Sample
  - Catchment Area for Sample of PWID
  - Estimate of PWID Population Density
- Part 1B: Population Size Estimation for Men Who Have Sex with Men (MSM) 13
  - Population Size of MSM
  - Sociodemographics of the MSM Sample
  - Catchment Area for Sample of MSM
  - Estimate of MSM Population Density

Part 2: Behavioral Risk Assessments 20

- Part 2A: PWID Sample 20
  - Frequency of Bloodborne Viral Disease Behavioral Risks
  - Bloodborne Viral Disease Prevalence – HIV and hepatitis C virus (HCV)
  - Behavioral Risks Associated with HCV Prevalence
  - Opioid Overdose Frequency and Associated Risks
- Part 2B: MSM Sample 27
  - Frequency of Sexual Behavioral Risks
  - HIV Prevalence
  - Behavioral Risks Associated with HIV Prevalence

Part 3: Access to Services 31

- HIV Treatment Cascade for MSM

Part 4: Stigma Experiences among PWID and MSM 33

Recommendations 36

Appendices 39

- Alternative Population Estimation
  - For PWID
  - For MSM

INTRODUCTION

This project report presents the research design for, data collection from, and analyses conducted on sampling from populations of people who inject drugs (PWID) and men who have sex with men (MSM) who reside in Lebanon. The authors of this report served as consultants to the Middle East and North Africa Harm Reduction Association (MENANRA). In this role, the consultants were responsible for organizing and training teams to sample individuals from the two hidden populations, collect data from sampled individuals through survey instruments, and provide access to testing for antibodies to HIV (for PWID and MSM) and to hepatitis C virus (for PWID only). Consultants were also responsible for preparation of valid and reliable databases containing the survey and serological data and for analysis of the data. Ethical approval for human subjects research was obtained from the Institutional Review Board at the Lebanese American University (IRB#: LAU.SOM.JM1.9/April/2015).

The consultant team and MENAHRA agreed that the best available sampling methodology to reach members of the two hidden populations and optimize the chances of obtaining a broad and diverse sample was one that relied on already existing social ties within each target population. The most commonly used sampling method that meets these criteria is Respondent-Driven Sampling (RDS). Over the past decade, RDS has become the standard recruiting method for reaching hidden populations. Studies using this method have generally (but not universally) been able to generate samples more quickly and at lower cost than studies using time-location or outreach worker driven sampling methods. RDS has produced useful samples from hidden populations in most regions of the world, but has been most especially effective in urban populations in which the number of social connections between individuals and across groups of individual is often heightened by geographical proximity. RDS uses a dual-incentive structure to recruit and enroll study participants. Individuals are compensated for the time they spend responding to interviewer's questions and rewarded for successfully recruiting other individuals from within the target population.

Another benefit of RDS is that subjects do not reveal identifying information about their social contacts. Thus, the privacy of non-participating population members is preserved. Instead, participants willing to serve as recruiters are given coupons that they can use to invite other eligible individuals to participate in the study. The recruiter distributes these to their eligible social contacts. Until an individual with a coupon responds to the information on the coupon and contacts the study, that individual remains unknown to study staff so that privacy is protected. At least one previous RDS study conducted in Lebanon suggested that RDS methods are acceptable among the hidden populations in the country.

Recruitment is initiated by identifying a few individuals within the hidden population. These individuals, called "seeds," are often known to study team members or are identified as they contact social welfare organizations that serve the hidden population. Once seeds are identified, recruited by direct contact with study team members,

enrolled, and have been interviewed, they are provided coupons to give to other members of the hidden population whom they know. When coupon holders agree to participate in the study and have completed the interview, they too are also given coupons to invite others to participate. In this project both PWID and MSM respondents were given four coupons. This method of engaging newly recruited participants to become recruiters is felt to facilitate access to the study to many different groups and networks within the hidden population. Individuals can be sampled and interviewed only once; thereafter they can serve as recruiters until their coupons have been exhausted.

In addition to survey data and subsequently collected serological data, data are obtained on the size and composition of each participant's egocentric social network. For nearly twenty years, this partial network information has tantalized researchers and public health professionals, and they had produced attempts at generating statistical analysis that sought to use the data to reveal features of the hidden population as a whole. These statistical approaches have generally used the network size data to adjust the estimates of important behavioral and biological parameters such as the frequency of risk behaviors, the prevalence of HIV, or the impact of structural level factors on individual level risk or prevalence. In particular, the usual estimator for the population mean (HIV prevalence, for example) simply weights each observation by the inverse of subjects' reported network degree. Unfortunately, standard statistical inferences from RDS-generated samples have proven inadequate and statistically suspect. Traditional weighting approaches to population inference from RDS data are controversial and suffer from bias and inflated variance. This weighting method is known to cause bias; its reliance on self-reported network degree can result in unreliable estimates with large variance.

In place of traditional weighting, we have applied recently developed statistical tools for population estimation from RDS that do not rely on assumptions about the weighting observations. Our methods take advantage of all available information from respondent-driven samples -- the full recruitment tree, the pattern of allocation and depletion of coupons, and the timing of recruitments -- to estimate population characteristics and the size of the hidden population.

The criteria used for defining the populations of interest are the same that have been applied in many international studies of hidden populations at highest risk for HIV infection. Initial criteria were assessed in a brief screening questionnaire. After obtaining informed consent, data from the longer survey were used to verify that all individuals who were screened and consented to participate were members of the population of interest. For MSM, the sole screening criterion asked individuals to report whether they had engaged in either anal or oral sex with another man in the last 12 months. For PWID, initial screening gave the interviewer two sets of qualifying criteria: if injection stigmata could be shown by the potential recruit or, if not, then self-report of injection in the past 3 months followed by a convincing description of the process of injecting drugs. For both groups, additional questions in the body of the questionnaire, administered after informed consent was obtained, asked more detailed questions on sexual

behaviors that could be used to verify if participant was truly a member of the hidden population of interest.

The questionnaires used in this study were adaptations of surveys used in many previous studies of hidden populations that seek to obtain behavioral data and correlate with serological data on bloodborne disease prevalence. Broadly speaking, such bio-behavioral studies have collected self-reported data from members of the hidden and linked these data to serological test results by testing conducting by study personnel. In this study, however, the study did not have the resources to directly test participants. Instead, after completing the interview, participants were referred to service providers offering HIV, HCV, and HBV testing.

The study was conducted by a team selected by the consultants with input on several levels from MENAHRA. First and foremost, MENAHRA identified non-governmental social welfare agencies that worked directly with PWID or MSM communities. Outreach workers from these agencies were invited to a training workshop that produced a final adaptation of a separate behavioral survey for each of the two populations. The major source for questionnaire content and phrasing of questions was the Integrated Bio-Behavioral Survey (IBBS) toolbox available from the University of San Francisco Global Health Sciences. In conjunction with the HIV Epidemiology section of the San Francisco Department of Public Health, Global Health Science provides free and publicly available tools for conducting international HIV surveillance. The result is a toolbox with surveys and other data collection tools that produce high-quality, user friendly, reliable and reproducible data. The surveys are designed to allow the users to inform policy and evaluate programs serving HIV-affected populations. The surveys in the toolbox can be easily adapted to local conditions and peculiarities of language, thus promoting the collection of reliable data.

In this study, the surveys for PWID and MSM from the toolbox were translated from English into locally colloquial Arabic and adapted for use in Lebanon by collaboration between the consultants and outreach workers from social welfare agencies serving the two hidden populations. Once initial adaptation was completed, outreach workers from different agencies were trained by the consultants to administer the survey, to collect all pertinent RDS recruitment data, and to undertake active referral to testing that was routinely conducted by their social welfare agency. Subsequent field-testing of the questionnaires led to changes incorporated into a final version that was used from the first day of data collection on 9 October 2014.

Sample recruitment and data collection for both populations ended on 15 February 2015. During this time, RDS yielded 292 MSM beginning with 19 seeds and 390 PWID beginning with 25 seeds. Active seed selection occurred both at the outset of data collection in October and again in mid-January after a slowdown in recruitment during the Christmas-New Years season.

Analysis of the data featured in this report has been divided into four discrete parts. The first part provides estimates for the size of the target populations sampled through

this project. Networks of PWID and MSM from different regions of Lebanon are likely to have been recruited at different rates and reached at different times during the four-month recruitment period. Discussion in this part of the report will focus on determining the most probable catchment area that the study covered. The second part of the report describes sociodemographic, health, and behavioral characteristics of the two samples. More detailed analysis explores associations of behavioral and sociodemographic factors with the prevalence of HIV antibodies among MSM and HCV antibodies among PWID and with the experience of drug overdose in the PWID sample. The third part explores access to HIV care among MSM since as the report will show HIV prevalence among sampled PWID is negligible. Using the limited self-reported data on HIV testing and service use, we will produce a graphical representation of the treatment cascade, which can be used to identify gaps where directed use of even limited HIV prevention and care funding can have a large impact on improving access to care. The fourth part takes advantage of questions on the survey that ask respondents to report on their personal experiences of stigma and discrimination. Identifying common areas in which members of hidden populations are subjected to emotional or physical abuse can assist the social welfare agencies in designing interventions to improve the well-being of the population they serve.

PART 1: POPULATION ESTIMATION

Basic Principles

Estimation of target population size from RDS survey data proceeds by analyzing features of the respondents' social network and extrapolating this network information to the whole population. The RDS recruitment process reveals links between each recruiter and the subject(s) they recruit. Social, drug use, or other links among individuals are not observed between two sampled individuals unless one of those individuals recruited the other. Since respondents report their network degree (the number of target population members they know), the number of links emanating from the sub-network of respondents to the rest of the population can be estimated. From this, the total size of the target population can be estimated. A full description of the method for estimating population from these data, the mathematics behind the estimation, and an applied example from field data can be found on-line at http://arxiv.org/pdf/1504.08349.pdf.

Part 1A: Population Size Estimation for People Who Inject Drugs

Recruitment dynamics

From 25 PWID seeds, n=390 subjects entered the study. Most subjects received 4 coupons. Recruitment in this study was rapid: the mean time between interview of a subject and recruitment of another subject was 11.3 (± 14.4) days. Among successful recruiters, the mean time to recruitment was 8.6 (± 11.3) days. Figure 1-1 shows the cumulative number of recruitments by study day.

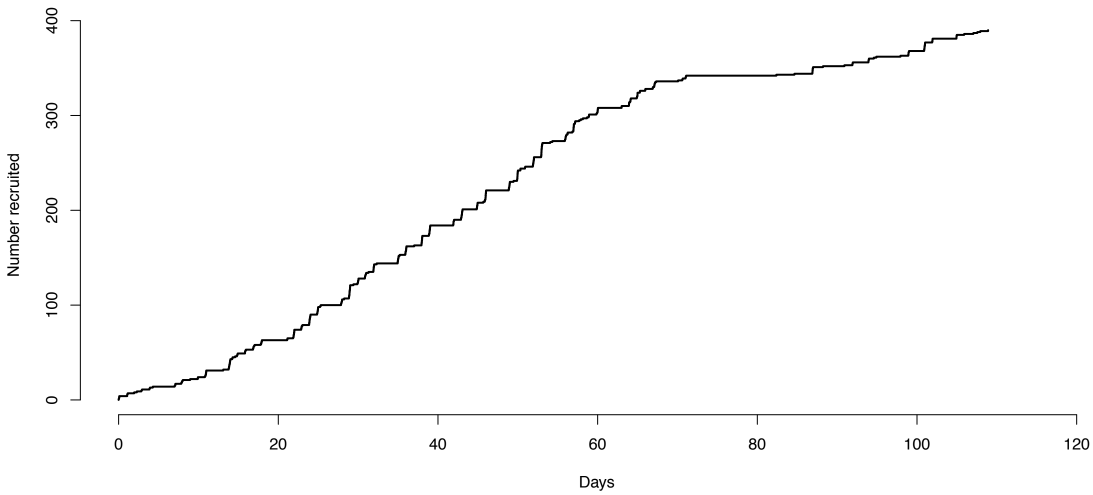

Figure 1-1. Cumulative number of recruitments by day of study

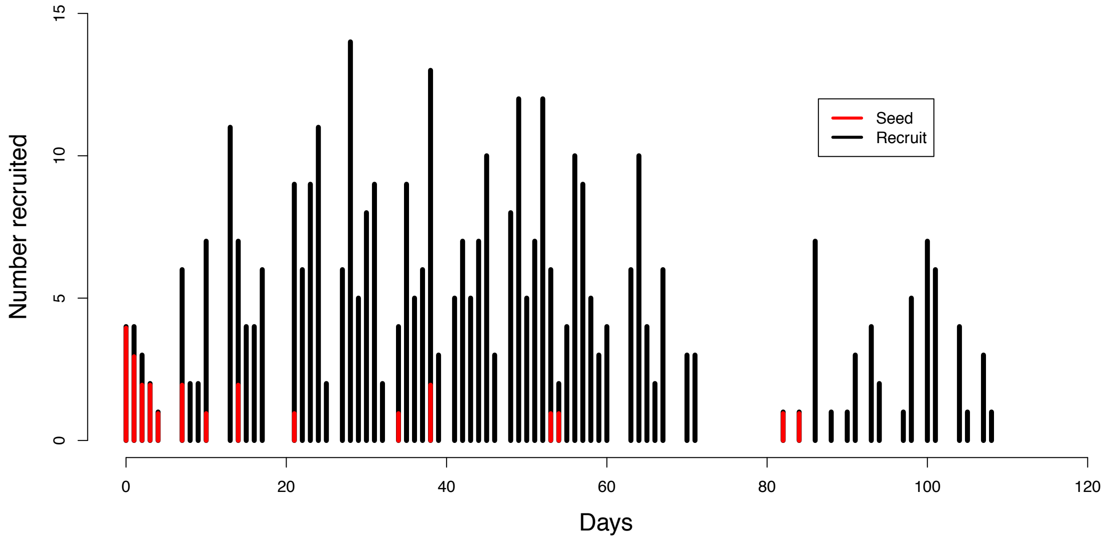

Figure1-1. Number of recruits per study day. Seed subjects are indicated in red.

Figure 2 shows the number of subjects interviewed on each day of the study, with seeds indicated in red. Breaks in recruitment occurred during weekends and holidays.

The mean number of subjects recruited by each subject was 0.9 (±1.5) subjects overall, and 2.7 (± 1.3) among subjects who recruited at least one other subject. The Table below shows the frequency of each number of recruits from each subject.

Table 1-1: Distribution of Coupon Redemption by PWID Study Participants

Number recruited	0	1	2	3	4
Frequency	256	37	22	16	59

<u>Individual network characteristics</u>

The mean network degree of subjects was 15.8 (± 17.5), and the maximum reported degree was 200. The mean network degree among successful recruiters was 19.5 (± 23.9). Some subjects reported degrees were less than the number of others linked to them in the recruitment tree. In these cases, reported degrees were replaced by inferred degrees based on their degree in the recruitment tree.

Figure 1-3 shows recruitment trees from the ten productive seeds. The recruitment pattern reveals that it was most common for participants who were successful recruiters to have all four of their coupons redeemed and this accounted for 236 (63%) of the sample recruited by other participants. The percentage of participants who had all four coupons redeemed did not decrease over the course of the study.

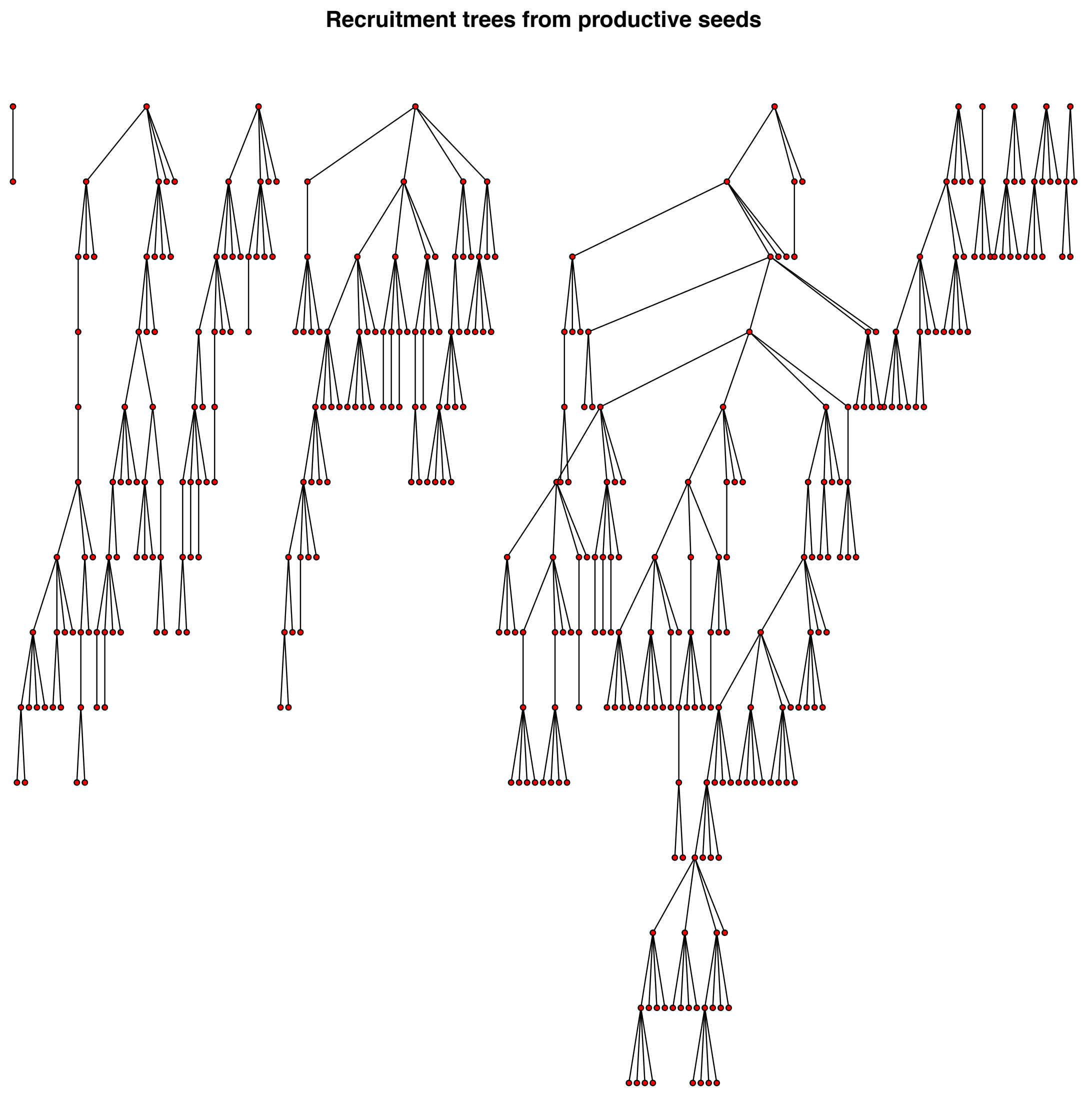

Figure 1-2. Recruitment trees from the ten productive PWID seeds.

<u>Population size estimates</u>

We estimate the size of the PWID population in the target region to be 3,114 (± 2302) people. The quantiles of the posterior distribution are given in the Table below.

Table 1-2: Distribution of Population Size Estimates

Quantile	2.5%	5%	10%	50%	90%	95%	97.5%
	611	703	853	2451	6331	7507	8842

Figure 4 shows a histogram of the posterior distribution of the population size, with the mean given in red.

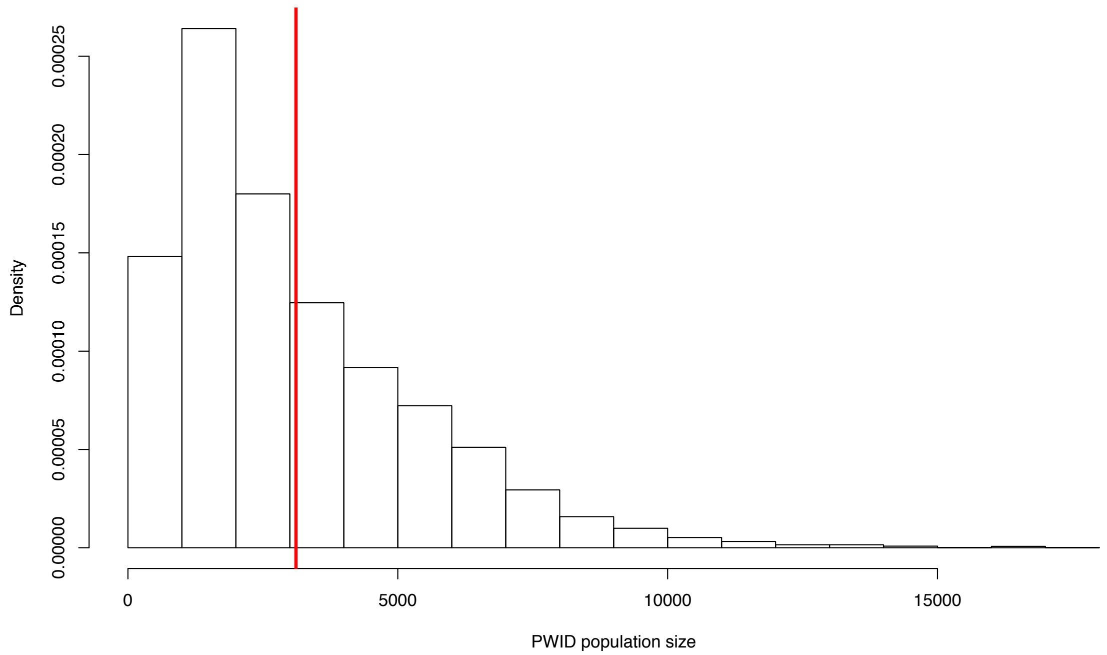

Figure 1-3. Histogram of the posterior distribution of PWID population size. The posterior mean is 3114, shown in red.

The 398 participants were drawn from among a population best estimated at 3,114 people. This is further evidence that the sampling did not reach a point at which finding unrecruited but eligible individuals would have become difficult.

The sociodemographic characteristics of the PWID recruited into the study are shown below in Table 1-3. Among the most important of the demographic variables is the low proportion of women in the sample: only 17 of the 390 individuals who enrolled in the study (4.4%). This is lower than the number we would expect upon examining the data collected from participants regarding their social network. Participants reported having face-to-face contact with an average 18.53±19.63 different people in the month prior to being interviewed. Of these, contacts with female PWID were 2.64±7.09, or 14.2% of their contacts. Thus, we conclude that women may be significantly under-represented in the sample. Another feature of the sample worth noting is that although the majority was poor, earning less than $500 per month, there was substantial residential stability, with a mean duration of 19.2±12.8 months at the same residence. We did not collect data that would indicate the proportion of the sample that considered itself homeless.

Table 1-3: PWID Demographics

Age	Mean<br>31.7	s.d.<br>13.3	Median<br>28	IQR<br>23, 35
	Number	Percent		
Women	17	4.4%		
Country of Birth				
Lebanon	370	95.6%		
Syria	9	2.3%		
Other	8	2.1%		
Educational Level				
Never completed primary school	89	23.0%		
Completed primary school	160	41.3%		
Completed secondary school	81	20.9%		
Any post-secondary	57	14.7%		
Employed or Earning an Income (yes)	140	36.2%		
Range of Income (reduced from 5 categories)				
≤$500	210	54.3%		
$501-2,000	103	26.6%		
>$2,000	2	0.5%		
Marital Status				
Currently married	51	13.2%		
Formerly married	44	11.4%		
Never Married	292	75.5%		
Not Residing in Beirut	47	12.1%		
Duration of Current Residence, in months	Mean<br>19.2	s.d.<br>12.8	Median<br>20	IQR<br>9, 30

Mapping of individual respondents' neighborhood of residence was used to visualize the spatial distribution of the sample. As seen in Figure 1-5 below, more than 85% of participants resided within the city limits of Beirut. Approximately 10% resided in neighborhoods just beyond the city limits and a few individuals resided along the coast to the north of Beirut.

Thus, it seems reasonable to conclude that the catchment area for PWID should be defined as greater Beirut and not the whole of Lebanon. Given the uncertainty in the actual population of the city, it is hard to provide a single point estimate for the density of PWID in the catchment area and even harder to provide an population-adjusted density estimate that limits the denominator to the size of the population with the age range of 18 to 45, the range for the vast majority of the PWID sample.

Part 1B: Population Size Estimation for Men Who Have Sex with Men

<u>Recruitment dynamics</u>

From 19 seeds, n=292 subjects entered the study. Most received 4 coupons. Recruitment was fast, but not as fast as in the PWID study. The mean time between interview of a subject and recruitment of another subject was 14.5 (± 14.5) days. Among successful recruiters, the mean time was 15.6 (± 14.8) days. Figure 1-6 shows the cumulative number of recruitments per day.

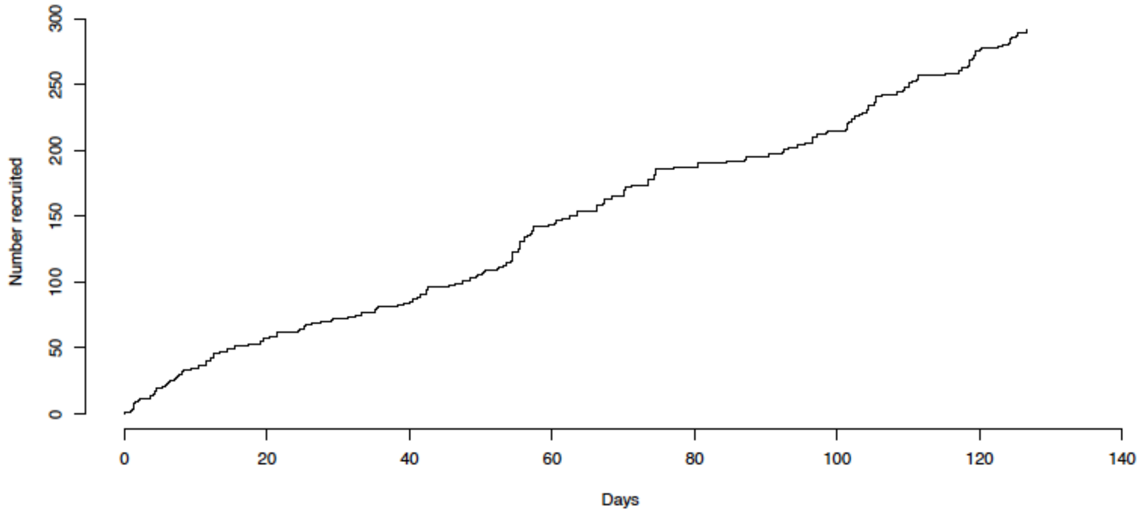

Figure 1-6. Cumulative number of MSM recruits by day of study.

Figure 1.7 shows the number of subjects interviewed on each day of the study, with seeds indicated in red. Short breaks from recruitment occurred during weekends and a longer break occurred during the Christmas-New Year holiday season. However, unlike PWID recruiting, there was no need to identify new seeds after the holiday in order for the recruitment rate to return to pre-holiday levels.

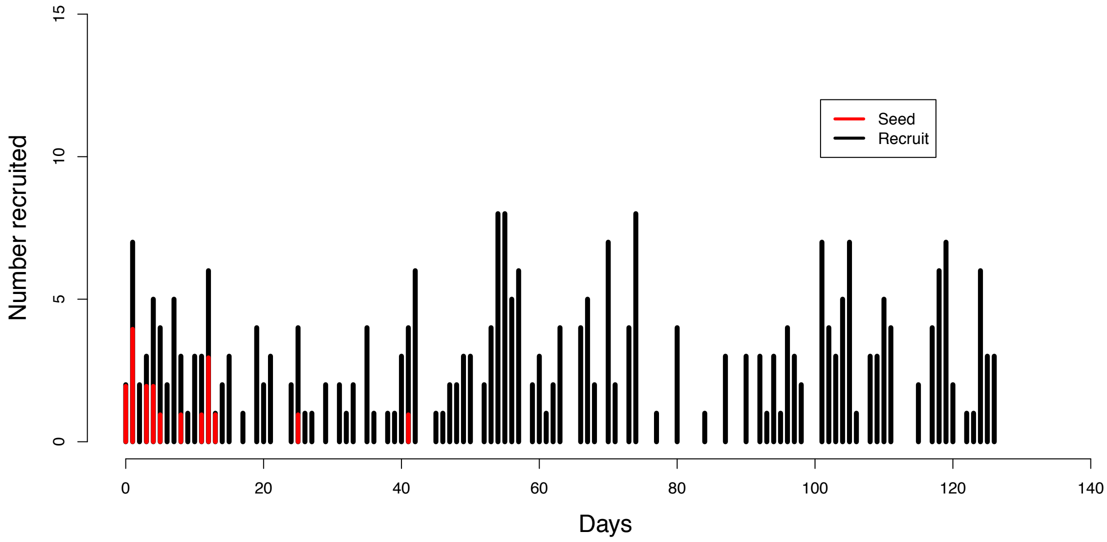

Figure 1-7. Number of recruits per study day. Seed subjects are indicated in red.

The mean number of subjects recruited by each subject was 0.9 (±1.3) subjects overall, and 2.1 (± 1.2) among subjects who recruited at least one other subject. The table below shows the frequency of each number of recruits from each subject. In contrast to PWID coupon redemption (in which nearly two-thirds of recruits were recruited by participants who had all four of their coupons redeemed), only 104 recruits (38% of the sample excluding seeds) were from respondents who had all four coupons redeemed.

Table 1-4: Distribution of Coupon Redemption by MSM Study Participants

Number Recruited	0	1	2	3	4
Frequency	163	54	32	17	26

Individual network characteristics

Reported MSM networks sizes were much larger than PWID network sizes. The mean network degree of subjects was 35.3 (± 46.8), and the maximum reported degree was 200. The mean network degree among successful recruiters was 37.8 (± 48.1). Some subjects' reported degrees were less than the number of others linked to them in the recruitment tree. In these cases, reported degrees were replaced by inferred degrees based on their degree in the recruitment tree.

Figure 1-8 shows a histogram of MSM subjects' reported degrees. It is interesting to note that larger network sizes reported by MSM compared to PWID did not result in a larger proportion of the recruitment occurring when all four coupons were redeemed. Figure 1-9 shows the recruitment trees.

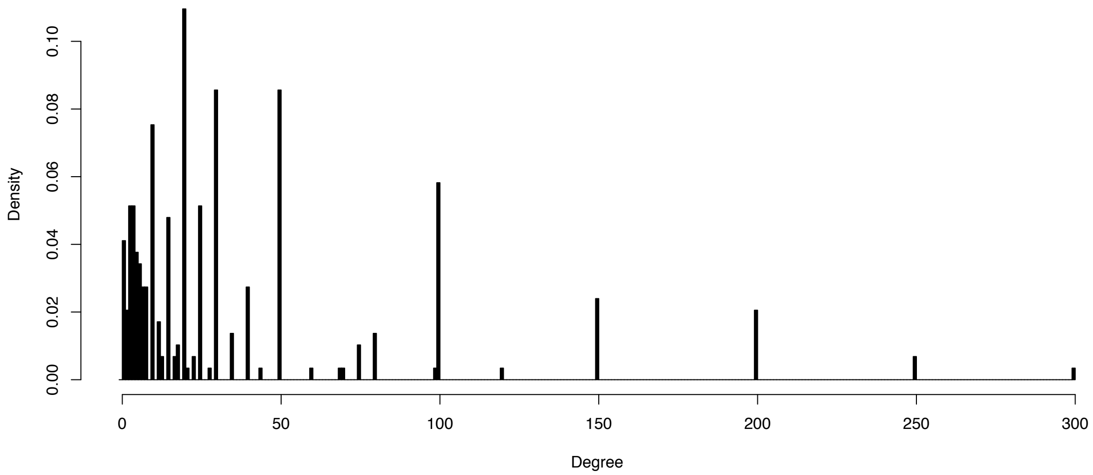

Figure 1-8. Histogram of MSM subjects' reported degrees.

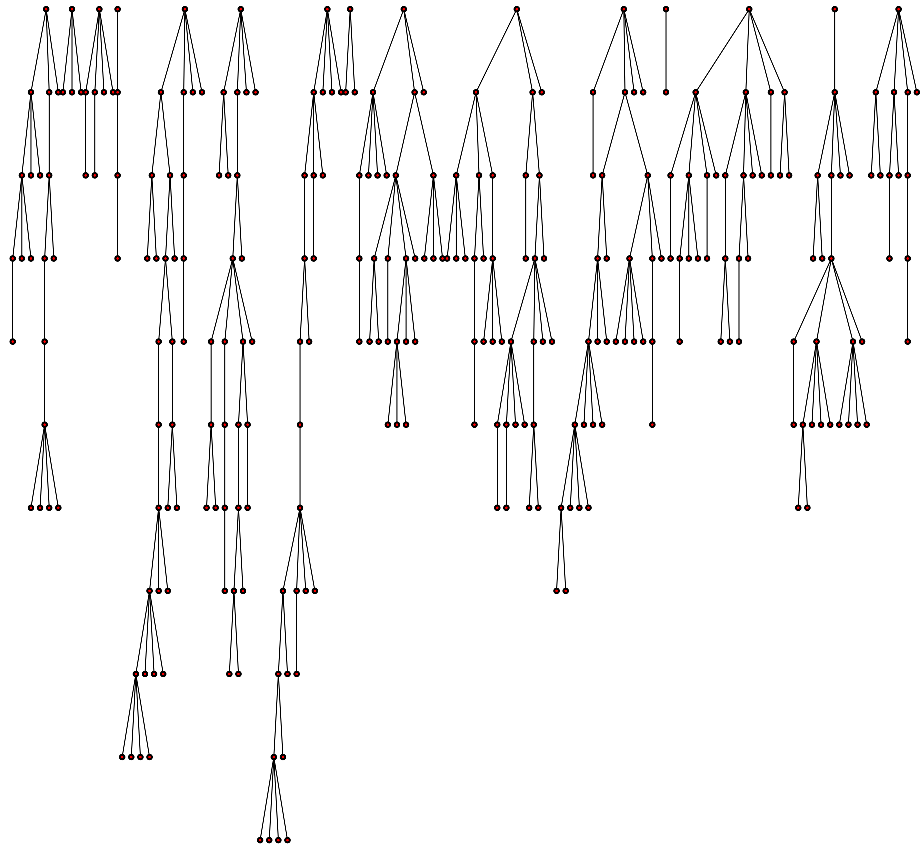

Figure 1-9. Recruitment trees from the productive seeds.

In keeping with the finding that fewer respondents had all four coupons redeemed, MSM chains were shorter but more numerous than the PWID chains. As with the PWID recruitment pattern, the percentage of participants who had all four coupons redeemed

did not decrease over the course of the study, suggesting that the population was not being depleted of eligible participants.

Population size estimates

We estimate the size of the MSM population in the target region to be 4220 (± 2192) people. The quantiles of the posterior distribution of population size estimates are given in the Table below:

Table 1-5: Distribution of Population Size Estimates

2.5%	5%	10%	50%	90%	95%	97.5%
2100	2243	2248	3615	6550	8304	11034

The posterior credible interval (2100, 11034) is wide, reflecting significant uncertainty about the true size of the target population. Figure 1-10 shows a histogram of the posterior distribution of MSM population size estimates, with the posterior mean shown in red.

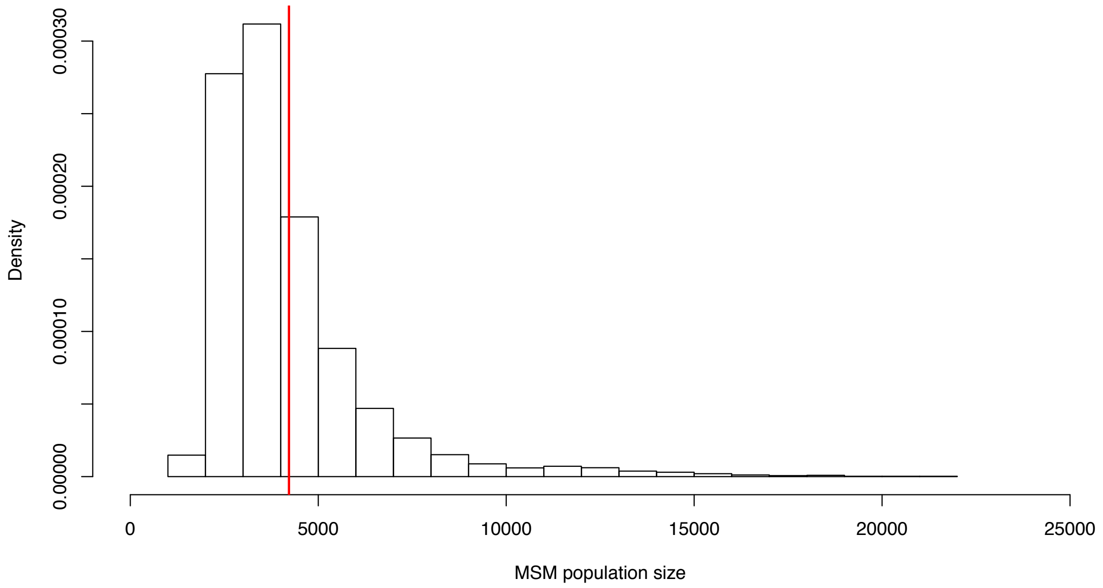

Figure 1-10. Histogram of posterior distribution of MSM population size estimates. The posterior mean is 4220, shown in red.

The sociodemographic characteristics of the PWID recruited into the study appears below in Table 1-6.

Table 1-6: MSM Sociodemographics

Age	Mean<br>27.1	s.d.<br>8.1	Median<br>25	IQR<br>21, 30
Country of Birth				
Lebanon	206	71%		
Syria	72	25%		
Iraq	2	1%		
Other	10	3%		
No Response	2	1%		
Educational Level				
Never attended school	3	1%		
Completed primary school	62	21%		
Completed secondary school	52	18%		
Some post-secondary	82	28%		
Completed post-secondary	90	31%		
Employed or Earning an Income (yes)	177	60%		
Range of Income (reduced from 5 categories)				
≤$500	112	38%		
$501-2,000	109	37%		
$2,001-$4,000	29	10%		
>$4,000	2	<1%		
No Response	40	14%		
Marital Status				
Currently married	10	3%		
Formerly married	15	5%		
Never Married	264	90%		
Not Residing in Beirut	0	0		
Duration of Current Residence, in months	Mean<br>70.1	s.d.<br>86.9	Median<br>27	IQR<br>5, 72

The responses to questions about residence reveal that all MSM participants resided in Beirut. The neighborhood distribution is shown in the map (Figure 1-11) reveals the highest concentrations in highly urbanized districts. Sociological considerations also suggest that the target population catchment area is not all MSM in Lebanon; instead, the true target population catchment area is probably the subset of MSM near Beirut for whom the offered compensation or the desire to participate is a sufficient incentive to participation.

One quarter of the respondents were Syrian-born. As a group, they had a lower HIV prevalence than their Lebanese counterparts, but higher rates of some HIV risk behaviors and higher levels of MSM-related discrimination and abuse (see Part 2B of this report). Referral patterns in the RDS recruitment chains suggest that Lebanese and Syrian interact with each, however Syrian MSM are more likely to recruit Lebanese MSM than Lebanese MSM are to recruit Syrians.

As with t[illegible]mple of PWID, it seems reasonable to conclude that the catchment area for MSM should be defined as greater Beirut and not the whole of Lebanon. Given the uncertainty in the actual population of the city, it is hard to provide a single point estimate for the density of MSM in the catchment area and even harder to provide an population-adjusted density estimate that limits the denominator to the size of the population with the age range of 18 to 45, the range for the vast majority of the MSM sample.

PART 2: BEHAVIORAL RISK ASSESSMENTS

Basic Principles

Self-reported answers to survey were compiled to determine the frequencies of individual risk behaviors and practices. Once the behaviors are quantified, correlations between these behaviors and negative outcomes of interest can be identified. In this report we focus on viral infections most commonly transmitted in the target populations and on opioid overdoses. It is important to determine the existence of correlations between behaviors and these negative outcomes since they can reveal opportunities for population-wide interventions to reduce risk. However, it also must be kept in mind that in exploring the associations between recent behaviors and disease prevalence, we cannot reach any conclusions about causality since the study design precluded collecting information about the temporal relationship between engaging in risky behaviors and the timing of disease acquisition.

Part 2A: Risk Behaviors and Bloodborne Viral Prevalence in the PWID Sample

For PWID, we concentrated on risks associated with unsafe injection and, consequently, transmission of bloodborne viruses. The survey posed questions about injection practices and syringe handling behaviors at several different timeframes. Some of the questions were asked about the participant's entire injection career. For example, such questions included ever injecting while incarcerated, ever experiencing a drug overdose, and length of the injection career. Some were asked about injection practices in the month prior to interview; these included, for instance, frequency of injection, types of drugs injected, and sources for syringes. Some were asked about the last three specific injection episodes. These questions focused on specific behaviors known to be associated with disease transmission including using a syringe previously used by another injector, injecting with a syringe with a detachable needle, and injecting drugs that had been apportioned by someone else's syringe. The full list of injection risk variables and the frequency at which these were reported appears in Table 2-1.

Several behaviors and injection practices associated with higher rates of HIV and HCV transmission were commonly reported by respondents. Among the risks that should be highlighted and for which prevention interventions should be attempted are:

- Injecting in prison, especially with a shared syringe;
- Injecting with others, especially when other people's syringes are used in the process of dissolving and apportioning drugs.

In addition to injection risks, the frequency and repeated nature of overdose among the sample should be addressed by local harm reduction agencies. More than two in five study respondents reported experiencing an overdose. This is similar to overdose rates found in most other parts of the world where opioids are the drugs most commonly injected.

Table 2-1: PWID Drug Use and Injection History and Current Practices

**Drug Use History**		
Age at First Injection	Mean ± s.d.<br>19.5+5.8	Median, IQR<br>18 (16,21)
Drug Injected First Time		
Heroin	332	85.8%
Cocaine	45	11.6%
Other	8	2.2%
Ever Arrested	292	75.5%
If Arrested, How Many Times	Mean ± s.d.<br>5.07+5.19	Median, IQR<br>3 (2,6.5)
Ever Incarcerated	291	75.2%
Ever Injected in Prison	48	12.4%
Ever Shared a Syringe While In Prison for those who ever injected in prison	12	25%
Ever Entered Substance Abuse Treatment	200	51.7%
If Ever, How Many Times	Mean ± s.d.<br>3.04+3.28	Median, IQR<br>2 (1,4)
Ever Experienced an Overdose	168	43.41%
If Any Overdoses, How Many	Mean ± s.d.<br>2.95+3.00	Median, IQR<br>2 (1,4)
**Current Drug Use**		
Days Injected, Last 30		
Every day	265	65.6%
About every other day	70	18.0%
1-3 times per week	43	11.0%
Less than once per week	18	4.6%
On the Day You Injected, How Many Times on Average	Mean ± s.d.<br>2.77+1.10	Median, IQR<br>3(2,4)

What Drugs Did You Inject, Last 3 Episodes		
Heroin	263	67.5%
Pharmaceutical Opioid	41	10.6%
Cocaine	70	17.9%
Other	17	4.3%
Normal Syringe Source		
Pharmacy	318	82.2%
Syringe Program	51	13.2%
Other Injector	13	3.4%
Other	4	1.0%
**Injection Practices**		
Did You Inject With Others		
Alone	267	68.0%
With one person	78	19.6%
With more than one person	40	8.3%
When Injecting with Others, How Many?	Mean ± s.d.<br>1.79±0.45	Median, IQR<br>2 (1,2)
Divided Drugs After Dissolved	68	17.4%
Divided Using Someone Else's Syringe	121	31.1%
Injected with a Syringe and a Detachable Needle	40	10.31%
Injected Intravenously	349	89.5%
First Time Use of the Syringe	280	71.7%
Used by Another Injector Before You	20	5.1%
If Use by Another Injector, Is This Person Infected with HCV	3	0.8% of all injections<br>16.4% of shared injections
Inject Outside Your Own Home	311	79.8%
Among Those Who Did Not Inject Alone, How Many Other People Injected with You When You Injected Outside Your Own Home	Mean ± s.d.<br>2.20±2.43	Median, IQR<br>1 (1,2)

Note: Where percentages add up to less than 100%, the missing fraction is a result of missing data.
Variables in italics are the averages obtained from questions asking about injection behaviors during the respondent's last three injections.

Prevalence of Bloodborne Viruses and Other Medical Problems in the PWID Sample

Serological testing for HIV, hepatitis C virus (HCV) and hepatitis B virus (HBV) was offered to all participants. Despite the existence of substantial rates of unsafe injection, HIV prevalence in the sample was very low. A total of 288 PWID participants (73.5%) reporting having been tested for HIV prior to enrolling in the study and none reported having tested positive. Not all study participants were tested for HIV; 51 individuals did not seek HIV testing after completing the interview. Of the 339 who did seek testing, there was only one HIV-positive test result. Thus, prevalence of HIV in the sample was 0.26%.

On the other hand, HCV testing identified a far higher rate of infection among PWID. As with HIV testing, not all participants completing the interview sought or obtained HCV testing. A total of 302 PWID participants (77.4%) were tested with 82 positive tests results. Two individuals who were not tested reported a previous HCV-positive test result. Thus, prevalence of HCV in the sample was 27.6% (84 positive results from 304 individuals).

Testing for HBV was also offered and again not all participants sought HBV testing. Of the 299 participants tests, positive results were found for 6 individuals (2.0%).

There was evidence of co-infection in the sample. Three of the six individuals who tested positive for HBV also tested positive for HCV. The one individual who tested positive for HIV tested negative for both HBV and HCV.

The frequency of other medical problems that PWID we asked about are presented in Table 2-2. Abscesses were more common than sexually transmitted infections in this population.

Table 2-2: Frequency of Medical Problems Reported by PWID

Abscess or Soft Tissue Infection, last year	60	15.5%
Number of Abscesses, last year, for those reporting any	Mean ± s.d. 4.4±3.5	Median, IQR 3 (2,5)
Was Medical Treatment Sought	13 of 60	21.7%
Medically Diagnosed Sexually Transmitted Infection, last year	52	13.4%
Received Treatment for a Diagnosed Sexually Transmitted Infection	19 of 52	36.5%
Refused health care because someone believed you inject drugs, last year	18	4.7%

Sexual activity in the sample of PWID was common. The data on the frequency of sexual intercourse are presented in Table 2-3. The data are separately reported for men and women. It is worth noting that a small proportion of the male PWID reported having

sex with another man ever (3.3% of the entire male sample) or in the year prior to enrollment (1.6% of those reporting any sexual activity in that year).

Table 2-3: PWID Sexual Behaviors and Risks

For Men Only		
Ever Had Vaginal or Anal Sex with a Woman	361	97.8%
Ever Had Anal Sex with a Man	12	3.3%
Vaginal or Anal Sex with a Woman, last year	270	73.2%
Number of Female Partners, last year<br>Only for those reporting ≥1	Mean ± s.d.<br>2.69±3.72	Median, IQR<br>1 (1,3)
Anal Sex with a Man, last year	6	1.6%
Number of Male Partners, last year<br>Only for those reporting ≥1	Mean ± s.d.<br>2.17±2.04	Median, IQR<br>1 (1,3)
No Condom Use, for those reporting ≥1 partner<br>Female Partners<br>Male Partners	 176/270<br>4/6	 65.2%<br>66.7%
Gave Sex for Money, Goods, or Service<br>Female Partners<br>Male Partners	 24/270<br>0	 8.9%
Received Sex for Money, Goods, or Service<br>Female Partners<br>Male Partners	 11/270<br>2/6	 4.1%<br>33.3%
For Women Only		
Ever Had Vaginal or Anal Sex with a Man	12	70.6%
Vaginal or Anal Sex with a Man, last year	8	47.1%
Number of Male Partners, last year<br>Only for those reporting ≥1	Mean ± s.d.<br>2.38±3.11	Median, IQR<br>1 (1,2)
No Condom Use, for those reporting ≥1 partner	3	37.5%
Gave Sex for Money, Goods, or Service	0	0%
Received Sex for Money, Goods, or Service	0	0%

We explored the sociodemographic and behavioral factors that correlated with testing positive for having been infected with HCV. This multivariate model is presented in Table 2-4. First, all of the bivariate association of each of the sociodemographic and behavioral factors with a positive HCV test was determined. A large number of statistically significant bivariate associations were detected, and these fell into the following categories:

- Age – older chronological age and earlier age of first injection;
- Work – being unemployed, having a lower monthly income, and having been born outside of Lebanon or Syria;
- Involvement with the criminal justice system – ever arrested, arrested more often, ever incarcerated, and having injected while incarcerated;
- Ever entering a substance abuse treatment program;
- Ever experiencing an overdose;

- Having been refused healthcare after being identified as a drug user;
- Diagnosed with a sexually transmitted infection in the past year; and
- Recent injection behaviors – having more injection partners, not using a new syringe to inject, and injecting with syringes with detachable needles.

When entered into a multivariate model, the four factors that remained significantly associated with having been infected with HCV are presented in Table 2.4.

Table 2-4: Multivariable logistic regression of HCV risk factors among PWID

	**OR***	**95% Wald Confidence Limits**		**P value**
Ever Incarcerated<br>Yes<br>No	 4.093<br>ref	1.154	14.510	0.0291
First Time Use of the Syringe<br>Yes<br>No	 0.432<br>ref	0.217	0.860	0.0169
Medically Diagnosed STI, last year<br>Yes<br>No	 8.004<br>ref	3.699	17.319	<.0001
Number of PWID whose name is known and contacted, last month	1.038	1.016	1.061	0.0007

Table notes -- abbreviations OR: odds ratio; STI: sexually transmitted infections

The model reveals that being incarcerated, reusing syringes, having a diagnosed STI, and being in contact with more PWID are associated with being HCV seropositive. It should be remembered that these associations do not tell us anything about causality, since the temporal relationships are unclear, that is, we cannot know if the exposure to HCV that resulted in its acquisition occurred before or after exposure to the associated factor. However, three of the four associations reinforce the common sense notion that incarceration, injection with non-sterile syringes, and a larger injection network are all part of the risk environment experienced by PWID.

<u>Opioid Overdose Frequency and Associated Risks</u>

An experience of opioid overdose was reported by 200 study participants (51.7%). Among those reporting any overdoses, a pattern of repeat overdoses was also reported by more than half. The mean was 2.95±3.00 per person and the median was 2, suggesting that a small number of people were experiencing many episodes. Indeed, eight people reported ten or more overdoses. However, these people were no more likely to be among the 32 (8.2%) who reported an overdose in the past year.

Bivariate analysis was conducted to determine factor associated with ever experiencing an overdose. Factors that were statistically significant at $p \leq 0.05$ included:

- Sociodemographics – born outside Lebanon, having more income
- Drug history – injecting at a younger age, have been incarcerated and sharing syringes in prison, having entered substance abuse treatment, HCV seropositive, not tested for HIV
- Injection behaviors – injecting more often, injecting alone, reusing syringes
- Social network – being in contact with more PWID, knowing them by name, more female PWID
- Having had sex with a woman, ever
- Soft tissue infection in the past year

Multivariate modeling of the data, presented in Table 2-5, shows few factors that remained significant in the model – ever being incarcerated, ever entering substance abuse treatment, have a larger network of PWID, and reusing syringes at last injection.

Table 2-5: Multivariable Logistic Regression of Overdose Risk Among PWID

	Odds Ratio	95% Wald Confidence Limits		P value
Ever Incarcerated<br>Yes<br>No	 2.96<br>ref	1.49	5.86	0.002
First Time Use of the Syringe<br>Yes<br>No	 0.59<br>ref	0.36	0.97	0.039
Ever in Substance Abuse Treatment<br>Yes<br>No	 3.57<br>ref	2.22	5.73	<.0001
Number of PWID encountered, last month	1.01	1.00	1.03	0.045

Part 2B: Risk Behaviors and Bloodborne Viral Prevalence in the MSM Sample

For MSM, we concentrated on risks associated with unsafe sex and, consequently, transmission of bloodborne viruses. The survey posed questions about sexual practices and behaviors at several different timeframes. Some of the questions were asked about the participant's entire sexual life from the first episode of sexual intercourse. For example, such questions included ever having sex with a man and ever with a woman, age at first anal sex with another man, and marital status. Some questions asked the respondent to consider the year prior to interview; these included, for instance, how many different men, consistent use of condoms, group sex, and transactional sex. Some were asked about the last three specific sexual encounters. These questions focused on attributes of the partner (age, sex, and nationality), the nature of the relationship (continual, occasional, or one-time), and specific behaviors known to be associated with disease transmission (unprotected sex, abuse of alcohol or other drugs, and transactional sex). The full list of risk variables and the frequency at which these were reported appears in Table 2-6.

Table 2-6: MSM Sexual Behaviors and Risks

Ever Had Vaginal or Anal Sex with a Woman	102	35.4%
Vaginal or Anal Sex with a Woman, last year	36	12.3%
Ever Had Anal Sex with a Man	283	98.6%
Age at First Sex with a Man	Mean ± s.d.<br>16.7±4.2	Median (IQR)<br>10 (2.5, 27.5)
Anal Sex with a Man, last year	283	98.6%
Number of Male Partners, last year<br>Only for those reporting ≥1	Mean ± s.d.<br>20.7± 1.6	Median IQR<br>10.0 2.5, 27.5
No Condom Use, for those reporting ≥1 partner<br>Female Partners<br>Male Partners	 20/36<br>184/283	 55.6%<br>65.0%
Gave Sex for Money, Goods, or Service<br>Female Partners<br>Male Partners	 7/36<br>13/283	30.6%<br>4.6%
Received Sex for Money, Goods, or Service<br>Female Partners<br>Male Partners	Number<br>11/36<br>83	10.8%<br>29.3%
Engaged in Group Sex, last year	 61	21.6%
Number of Group Sex Encounters, last year<br>Only for those reporting ≥1	Mean±s.d.<br>3.5±4.8	Median (IQR)<br>2 (1,3)
Condom Availability during Group Sex	44/55	81.8%
Respondents' Condom Use during Group Sex, last encounter<br>Never<br>Sometimes<br>All	 30/61<br>20/61<br>10/61	 49.2%<br>32.8%<br>16.4%

Drug Use during Group Sex, last encounter		
Alcohol	45	81.8%
Stimulants (cocaine or methamphetamine)	7	11.5%
Club drugs (e.g., ecstasy, GHB, ketamine)	9	14.8%
Others	4	6.6%
Location of Last Group Sex Encounter	Number	Percent
Club	1	1.6%
Hotel	13	21.3%
Private Residence	44	72.1%
Other	4	6.6%
Finding Sex Partners	Number	Percent
Mobile App	120	41.1%
Internet	53	18.2%
Bar	30	10.3%
Other	89	30.5%

Several features of MSM sexual life reported by respondents should be noted. The interesting sociodemographic features are among those listed in Table 1-6. First, one-quarter of the respondents were born in Syria. More data need to be collected to determine if Syrian-born MSM are recent arrivals to Lebanon or have been living in Lebanon since adolescence or earlier. Second, 25 participants reported having been or currently were married, and ten reported currently being married. The spouses in these cases were divided equally between men and women, and those with same-sex spouses insisted that the interviewer record their status as married.

Among the data on behavioral risks, the most interesting results were the use of electronic media to find sexual partners and the high prevalence of group sex. On-line searching for partners was reported by 173 (59.7%) MSM whereas face-to-face first contacts were less common. Only 53 MSM (18.3%) reported making contacts at venues such as bars or clubs, 16 (5.5%) on the street, and 4 (1.4%) at private parties.

More than one in five MSM (n=61, 21.0%) reported engaging in group-sex during the year prior to interview. The mean number of group sex encounters was 3.6 and the range was from 1-25. Data on condoms use were available for 55 of these events. Condoms were made available on 45 (81.8%) occasions but on 18 occasions not everyone used them. Reporting on respondent's own behaviors during group sex revealed that condom use was much less frequent. Nearly half (n=30, 49.2%) never used a condom and only 10 (16.4%) use a condom for all intercourse.

Alcohol and drug use data were available for 48 of the 61 group sex events. Alcohol was available at 38 (79.2%), stimulants such as methamphetamine were available at 6 (12.5%), and club drugs were available at 18 (37.5%). Multiple substances were available at 16 (33.3%). Individuals reported consuming alcohol and stimulants at similar rates (alcohol: 81.8% and stimulants: 11.5%), Club drugs were more readily available (37.5%) than were consumed (14.8%).

Some individuals appeared to be in committed relationship; 52 (17.9%) reported sex with only a single male in the year prior to interview. Among these 52, more than three-quarters (n=40, 76.9%) reported that this person was someone with whom they regularly had sex at a mean frequency of just more than once per week (55.6 times in the past year) with a range from once to 261 times. Condom use in this group of monogamous MSM was rare; only 7 of those men who reported having sex at least ten times in the past year with that single partner reported always using condoms. We identified eight partnerships with known discordant HIV status. In five of the eight, condoms were always used, in another two condoms were sometimes used one-third or three-quarters of the time), and in one condoms were never used.

Few MSM reported injection drug use. Only 5 (1.7%) had ever injected and only one had done so in the six months prior to interview.

Prevalence of Bloodborne Viruses and Other Medical Problems in the MSM Sample

Serological testing for HIV, HCV and HBV was offered to all participants. Many individuals (n=193) reported having been tested for HIV previously and 209 agreed to be tested after completing the interview. Fewer people were tested for HBV (n=51) or HCV (22). Three participants tested positive for HBV and none for HCV. The reason for the low acceptance rates of hepatitis testing was not investigated.

We analyzed sociodemographic and behavioral factors associated with HIV infection in the MSM sample using bivariate analysis. Factors that were statistically significant at $p \leq 0.05$ included:

- Sociodemographics – older age, higher educational achievement, and shorter duration at their current residence;
- Less sexual risk behaviors during the last three encounters – less unprotected sex, fewer individuals with multiple partners, less transactional sex, and less sex while intoxicated or high on drugs.

Generalized logistic regression modeling reduced the number of variables significantly associated with being HIV positive to just two. The full model is shown in the Table 2-7, including those factors not significant.

The inclusion in the model of the two variables – age, having fewer partners in the last year – is consistent with expectations. Since becoming infected occurs once in a lifetime, it is not surprising that if risks are essentially constant over time, then the older an individual becomes, the greater the chances of becoming infected. More curious is the finding that being HIV infected was associated with lower risk. However, it is consistent with the finding that 33 of 37 people who were HIV-infected knew of their status, and it is generally recognized that once individuals are given an HIV positive diagnosis they tend to reduce their sexual risk taking. And this is especially true for MSM populations studied in the U.S. and Western Europe. This trend seems also to be the case for MSM in this study, but the cross-sectional data we collected cannot be used to assign causality.

Table 2-7: Model of Factors Associated with Prevalent HIV Infection among MSM

	Estimate	Standard Error	z-value	p-value
Age	***0.771***	***0.024***	***3.245***	***0.00118***
More than Secondary School	0.250	0.629	0.398	n.s.
Higher Income	-0.001	0.197	-0.003	n.s
Multiple Partners, last year	***-1.014***	***0.433***	***-2.340***	***0.01929***
Any Unprotected Sex, last year	-0.423	0.438	-0.966	n.s.
Any Transactional Sex, last year	-0.533	0.549	-0.971	n.s.
Sex While Drug or High, last year	-0.182	0.514	-0.355	n.s.
Intercept	***-3.115***	***1.021***	***-3.052***	***0.00227***

PART 3: ACCESS TO HIV TESTING AND CARE

Table 3-1 displays test results for HIV testing among MSM participants, both prior to and as a follow-up to enrolling in the study. Although the HIV prevalence among MSM, of around 13%, is similar to that found in many Western European and North American urban MSM communities, analysis of the HIV treatment cascade suggests that Lebanon is doing better at diagnosing and treating HIV infection among MSM. Analysis of HIV testing data revealed the high rates of awareness of infection (almost 90%) and complete access to care for those who were aware of their infection. An indication of this access can be seen in the data regarding the last patient care visit: the median time since last visit was 1.7 months, the mean was 3.0 months, and 75% of patients had been seen within the previous four months.

Table 3-1: MSM Medical History and Current Health Status

Previously Tested for HIV	193	66.6%
Tested as Part of Enrolling in the Study	209	72.1%
Never Tested	9	3.1%
HIV Positive<br>Among Those Previously Tested<br>Tested as Part of PSE Study<br>Total HIV+	 33<br>4<br>37	 <br><br>12.6% of all MSM<br>13.1% of those ever tested
Ever Received HIV Care	33	89.2% of all HIV+<br>100% of those aware
Last HIV Patient Care Visit, in months	3.0	Median, IQR<br>1.7 (0 .6, 4.0)
Ever CD4+ Cell Count	33	89.2% all HIV+<br>100% of those in care
Ever Viral Load Test	33	89.2% all HIV+<br>100% of those in care
Ever Started ARVs	31	83.8% of all HIV+<br>93.9% of those in care
Now on ARVs	31	83.8% of all HIV+<br>93.9% of those in care

Another indicator of the strong linkage to medical care that exists in the MSM community comes from the data on sexually transmitted infections, presented in Table 3-2. While sexually transmitted infections (STIs) were common, almost all of those for which a diagnosis was received by the participant, treatment for it was received.

Table 3-2: Sexually Transmitted Infections Reported by MSM

Medically Diagnosed STIs, last year	53	18.2% of all MSM
Received Treatment for a Diagnosed STI	52	98.1% of those diagnosed

The results suggest that a large majority of those infected are aware of their HIV infection and are receiving treatments that lower the probability of subsequent HIV transmission. However, there will remain those who are infected, undiagnosed, and at risk for transmitting the virus to others. It is possible to estimate this number among MSM in the Beirut area. Our best estimate for the number of MSM in the area is 4,220. Our study reached 290 individuals, leaving 3,930 in a situation similar to that prior to our contacting them, that is, two-thirds will have already been tested and the frequency of detecting previously undiagnosed HIV infections is 4 in 209 (1.91%). If we accept the appropriateness of using a simple multiplier approach, we estimate that there are 75 HIV-positive individuals (credible range of 35 to 205) with undiagnosed infection and 81 (credible range of 37 to 222) who are aware of their infection but are not in care and remain potential transmitters.

PART 4: STIGMA EXPERIENCES AMONG PWID AND MSM

The two questionnaires included eight items focusing on denial of opportunities to participants – housing, employment, worship, medical care, and food service – and experiences of abuse – verbal, physical, and sexual – in the year prior to interview. On the whole, PWID experienced greater degrees of stigma. For instance, as shown in Table 4.1, nearly half reported verbal abuse (47.9%) and 30% reported denial of employment or housing. Only one-third (34.1%) of PWID reported no experience of stigma in the past year. In contrast, as shown in Table 4.2, verbal abuse was experienced by 22% fewer MSM (or 37.3% of MSM participants). More than half the MSM (51%) reported no experience within the past year in any of the eight stigma domains.

Table 4.1: PWID Stigma Variable Analysis

	Number	Percent	Only This
Refused Health Care	17	4.4	2
Refused Employment	118	30.3	18
Refused Religious Worship	8	2.0	0
Refused Restaurant or Bar Service	9	2.3	0
Refused Housing	114	29.2	17
Verbally Abused	187	47.9	36
Physically Assaulted	94	24.1	7
Sexually Assaulted	4	1.0	0

Table 4.2: MSM Stigma Variable Analysis

	Number	Percent	Only This
Refused Health Care	4	1.4	0
Refused Employment	43	14.8	4
Refused Religious Worship	13	4.5	0
Refused Restaurant or Bar Service	11	3.8	0
Refused Housing	34	11.6	0
Verbally Abused	109	37.3	21
Physically Assaulted	42	14.4	1
Sexually Assaulted	35	12.0	3

A more detailed look at the distribution of responses on the eight items by PWID participants revealed that only 80 people (20.5%) reported experience in only one of stigma domains. Reporting across multiple domains was common (Table 4.3). On the highest extreme, 3 people reported five different kinds of stigmatizing experiences. One person reported five different forms of stigmatizing: refusal of healthcare, employment, religious worship, restaurant/bar service, and employment, but not physical or verbal abuse and two people reported refusal of employment, housing, health care plus both physical and verbal abuse. All individuals reporting experiencing stigma in three or four domains reported being verbally abused among the domains.

Among the MSM, reporting of experiencing stigma across more than two domains was always associated with having been verbally abused. There was one person who reported experiences in six stigma domains: Being refused health care, employment, and housing and being verbally abuse, physically assaulted, and sexually assaulted. Other combinations associated with having been verbally abused are presented in Table 4.4

Table 4.3: PWID Reporting of Stigma across Multiple Domains

	Refused Employment + Refused Housing + Physically Assaulted + Refused health care	Refused Employment + Refused Housing + Physically Assaulted	Refused Employment + Refused Housing	Refused Employment	Refused Housing	Physically Assaulted
Been Verbally Abused	2	29	18	32	26	44

Table 4.4: MSM Reporting of Stigma across Multiple Domains

	Refused Employment + Refused Housing + Physically Assaulted + Sexually Assaulted	Refused Employment + Refused Housing + Physically Assaulted	Refused Employment + Physically Assaulted + Sexually Assaulted	Refused Employment + Physically Assaulted	Refused Housing + Sexually Assaulted
Been Verbally Abused	5	8	3	12	7
	Refused Employment	Refused Housing	Physically Assaulted	Sexually Assaulted	
Been Verbally Abused	16	4	4	5	

RECOMMENDATIONS

Our recommendations cover (1) policy and prevention and (2) future research and (3) outcomes assessment based on study results. We encourage MENAHRA, the participating NGOs, the National AIDS Coordinator for Lebanon, and other stakeholders to discuss these recommendations and develop an action plan based on their deliberations.

Policy and Prevention

<u>*For PWID*</u>

- HIV prevalence among PWID is relatively low, but the level of risk behaviors is quite high. Prevention programs should continue to engage with this population. Particular attention and efforts should be directed to reducing probability of injection in prisons with reused syringes.
- Experience with drug overdose was high among our sample of PWID, particularly among those with a history of incarceration, substance abuse treatment, a larger network of PWID, and reusing syringes at last injection. Overdose prevention and response programs, including those that train PWID to administer naloxone if they witness an opioid overdose, are urgently needed for PWID in Lebanon.
- The prevalence of HCV in the sample of PWIDs was quite high (about 28%). More aggressive prevention and testing efforts to reduce the risk of HCV infection among PWID, particularly those with history of incarceration history, reporting reuse of syringes, having a diagnosed STI and having contact with large group of other PWID are warranted. Exploration of expedited and discounted access to directly acting HCV antiviral medications should be undertaken as a way to supplement prevention programs for controlling the HCV epidemic.

<u>*For MSM*</u>

- MSM who are married to women are unlikely to use protection and therefore pose a significant transmission risk of HIV to their spouse and require specific interventions.
- Some MSM adamantly insisted that their same-sex partnership be considered marriage. In light of the increasing international acceptance of same-sex marriage, exploration of the potential for such acceptance in Lebanon is warranted.
- A significant minority (21%) of MSM reported engaging in group sex. Specific prevention programs to reduce HIV risk among MSM who engage in group sex is needed. Among the steps to consider are more active provision of condoms and increased availability of and counseling for pre-exposure prophylaxis (PrEP).

- The majority of MSM has been tested and is aware of their HIV status, but given the high rates of transmission risk behaviors, new infections will result unless enhanced prevention efforts are made to reach the estimated 75 HIV-positive individuals with undiagnosed infection and 81 who are aware of their infection but are not currently in care and remain potential transmitters.

<u>*For PWID and MSM*</u>

- High rates of experienced stigma still exist in both populations with verbal abuse most common among both groups, often in combination with other forms of stigmatizing behaviors. Efforts to increase tolerance should include improving society-wide understanding of the medical mature of addiction and the non-deviant nature of same-sex relationships for a considerable proportion in all human populations.
- Hepatitis B vaccination should be widely provided to both populations.

Future Research

- The study did not deliberately recruit PWID and MSM who are refugees from Syria, Iraq or other countries and now residing in Lebanon. However, a quarter of the MSM reported Syrian birth. Given the large populations of refugees and the known risk of displacement and social disruption on HIV risk, we recommend that future research purposefully recruit refugee populations at risk for HIV. The research study should examine the social networking between refugees and Lebanese nationals and the potential for HIV risk associated with these social networks.
- Women drug injectors were under-represented in our study. Future research should focus on (1) identifying if there is an even more hidden population of women drug injectors and (2) if there is, learning more about their specific HIV related risks, infection status, and treatment needs.
- The catchment area for our sample was greater Beirut and not the whole of Lebanon. Future research should recruit populations at high risk of HIV who reside outside of greater Beirut. Assessments should include recruitment schemes that begin with non-Beirut residents and study their social networks through more active social network analysis than is possible using RDS.
- The study showed the feasibility of using RDS to recruit relatively large samples of PWID and MSM in Lebanon. A future intervention research study can test the efficacy of a social-network based intervention for these groups at high risk of HIV and other blood-borne infections.

Outcomes Assessment

- Newly diagnosed cases of HIV (especially in the MSM community) and HCV (especially in the PWID community) should be actively tracked and contact tracing should be provided to actively search for new infections within the social networks of those newly diagnosed.
- If overdose prevention and response programs are created, a system for reporting naloxone administration by those trained should be established.
- If social marketing is undertaken to reduce stigma, repeat administration to affected populations of a battery of questions to assess experience is warranted to determine is the social marketing efforts are successful. Likewise, the impact of any social marketing campaign on the attitudes of the general population should be examined.

APPENDICES

Alternative Population Estimation

There is currently one other method that has been proposed for using RDS data to estimate hidden populations. Handcock et al. write that their method is based solely on the RDS data on degree, i.e., the self-reported number of people in the hidden population of interest that each respondent had come into contact with in a defined period prior to being interviewed as part of the sampling process. In their most recent publication on the method (Biometrics 71:248, 2015), they write;

> Conceptually, our approach is to leverage the information in the sequence of sample degrees, or numbers of network contacts to infer the size of the hidden population. Link-tracing network samples are generally more likely to sample nodes with more network connections, or higher degree. Therefore, we would expect higher-degree nodes to be more likely to be sampled earlier in the sampling process. As the target population becomes depleted, we would expect higher-degree nodes to be sampled earlier, and lower-degree nodes to be sampled later. Therefore, the rate of decrease in the degrees of sampled nodes over the course of the sample provides information on the size of the hidden population.

We applied their method and obtained the following estimates.

	Mean	SD	2.5%	97.5%
N (PWID)	673	604	430	1779
N (MSM)	401	47	335	517

These population sizes are quite low, and the lower 2.5% quantiles are hardly larger than the sample sizes in the PWID and MSM studies themselves. While there is no gold standard for estimating population sizes from RDS data, we do feel that the method proposed by Handcock et al. is likely to be less reliable than the one we used in the presenting population estimations in the body of this report. There are two reasons for this. First, their method relies solely on degree data and we have noted, based on our analysis of more than half a dozen samples from hidden populations reached using RDS, that reporting of degree is frequently erroneously. People have trouble accurately remember the true number of people from the hidden population with whom they have had person-to-person contact with in a given time period. We find multiple examples when people report (1) a smaller degree than the number of coupons they redeem, (2) an unrealistic large number of contacts, and (3) degree rounding to numbers such as 10, 20, or 50. Second, one of their key assumptions is that sampling without replacement (i.e., people cannot be recruited into the sample more than once) will lead to progressive depletion of people with larger degree. Since they have more contacts, they are likely to be recruited first. But our analysis of actual RDS datasets suggests little if any evidence for a temporal trend in such depletion.

References:

Handcock, M. S., Gile, K. J. & Mar, C. M. (2014), ‘Estimating hidden population size using respondent-driven sampling data’, Electronic Journal of Statistics 8(1): 1491–1521.

Handcock, M. S., Gile, K. J. & Mar, C. M. (2015), ‘Estimating the size of populations at high risk for HIV using respondent-driven sampling data’, Biometrics 71(1): 258-266.
