# Supplementary material for: Progress towards the 95–95–95 targets to end HIV by 2030 in Lebanon, 2023
Source: PLoS One. 2025 Jun 13;20(6):e0321868. doi: 10.1371/journal.pone.0321868 (PMC12165419; doi:10.1371/journal.pone.0321868)
Supplement: S5 File — Lebanon HIV IBBS 2008 final report. (PDF) [file pone.0321868.s005.pdf]

National AIDS Control Programme

"Mishwar"

AN INTEGRATED BIO-BEHAVIORAL SURVEILLANCE STUDY AMONG MOST AT RISK POPULATIONS IN LEBANON: FEMALE SEX WORKERS, INJECTING DRUG USERS, MEN WHO HAVE SEX WITH MEN, AND PRISONERS

Final Report

November 2008

Funded by the World Bank, IDF Grant for Strengthening the National HIV/AIDS Monitoring and Evaluation and Surveillance Systems in Lebanon.

ACKNOWLEDGEMENTS

This report has grown out of the collective experience of a number of people implementing an Integrated Bio-Behavioral Surveillance (IBBS) study among Most at Risk Populations in Lebanon: Female Sex Workers, Injecting Drug Users, Men who have Sex with Men, and Prisoners.

The National AIDS Control Programme, Ministry of Health, Lebanon would like to acknowledge the World Bank Group, which funded the study through the IDF Grant for Strengthening the National HIV/AIDS Monitoring and Evaluation and Surveillance Systems in Lebanon. Without the financial and technical assistance of the World Bank Group, this study could not have materialized. NAP specifically thanks Dr. Francisca Ayo Akala (Senior Public Health Specialist WB), and Dr. David Wilson (Lead Health Specialist WB) for providing guidance required to implement the study. NAP also extends a special thanks to the World Bank consultant, Dr. Sarah Hersey (IBBS Specialist).

NAP is very grateful for the work of the IDF Project Coordinator, Miss Danielle El-Khoury (Study Coordinator).

NAP gratefully acknowledges the work of the American University of Beirut, Faculty of Health Sciences-Research Team who carried out the research and implemented the study. The research team includes: Dr. Kassem Kassak, Health Management and Policy Dept. (Team Leader); Dr. Jocelyn DeJong, Epidemiology and Population Health Dept. (AIDS expert); Dr. Ziyad Mahfoud, Epidemiology and Population Health Dept. (Biostatistician); Dr. Rima Afifi, Health Behavior and Education Dept. (Monitoring and Evaluation); Dr. Sawsan Abdurahim, Health Behavior and Education Dept. (Health Behavior); Dr. Sami Ramia, Medical Laboratory (Virologist and Medical Laboratory expert); Miss Farah El-Barbir (Research Assistant); Miss Maguy Ghanem (Research Assistant); Miss Sarah Shamra (Medical Laboratory Instructor); and, Mr. Khalil Kreidiyyeh (Medical Laboratory Technician).

NAP would like to thank the NGOs involved in the Study, including AJEM, Dar Al Amal, HELEM, SKOUN, and Oum El-Nour.

In addition, NAP would like to thank the World Health Organization, the different National Ministries, HepAttitude +, Roche and all those who helped in supporting the implementation of the research.

Sincerely,

Mostafa El-Nakib, M.D., MPH
NAP Manager

2008 - National AIDS Control Programme. This publication was funded by the World Bank, IDF Grant for Strengthening the National HIV/AIDS Monitoring and Evaluation and Surveillance Systems in Lebanon, which is managed by The National AIDS Control Programme, Ministry of Public Health in Lebanon.

AUTHORS

Rima Afifi
Jocelyn DeJong
Farah El-Barbir
Danielle El-Khoury
Maguy Ghanem
Kassem Kassak
Khalil Kreidiyyeh
Ziyad Mahfoud
Sami Ramia
Sarah Shamra

Table of Contents

I. Introduction ………………………………………………………………… 6

A. Recent Statistics ………………………………………………………… 6

B. Objectives ……………………………………………………………….. 6

C. Name of the Study ……………………………………………………… 7

II. Methodology ……………………………………………………………... 7

A. Population Inclusion and Exclusion Criteria ………………………… 7

B. Partnering with NGOs …………………………………………………. 8

C. Simple Random Sampling ……………………………………………... 9

D. Respondent Driven Sampling (RDS) ………………………………….. 9

E. Sampling Size …………………………………………………………… 10

F. The Survey ……………………………………………………………….. 11

G. The Process of IBBS ……………………………………………………….12

III. Progress at the Population Level ……………………………………... 13

A. Prisoners ………………………………………………………………….. 13

B. Injecting Drug Users (IDUs) …………………………………………… 15

C. Men who Have Sex with Men (MSM) ……………………………..….. 18

D. Female Sex Workers (FSWs) ……………………………………..……19

IV. Progress at the NGO Level …………………………………………….. 21

A. AJEM ……………………………………………………………………… 21

B. Oum El Nour ……………………………………………………………... 22

C. Skoun ……………………………………………………………………… 23

D. Helem ……………………………………………………………………..... 24

E. Dar Al Amal ………………………………………………………..…… 25

F. SIDC ……………………………………………………………………….. 27

V. Main Findings and Discussion ………………………………………… 27

VI. Major Obstacles ……………………………………………………….... 44

A. Structural and Sociocultural Factors …………………………….…………..... 44
B. RDS Technical Difficulties ……………………………...………………. 45
C. NGO Limitations …………………………………………..……………. 47
D. Biological Testing Difficulties ………………………………..…………… 48
VII. Steps Taken to Increase Recruitment …………………………..………… 49
A. Increasing Financial Incentives ………………………………….……….. 49
B. Engaging the NGO Outreach Workers ……………………..………… 50
C. Engaging a Mobile Interviewer ………………………………...….…... 50
D. Encouraging Conducting Interviews Outside NGO Center ……………... 50
E. Drafting a Schedule for Laboratory Results ………………………….... 50
VIII. Conclusion and Lessons Learned ……………………..……………... 51
A. Conduct Formative Research ……………………………..…………... 51
B. Ensure a Full-time Dedicated Staff ……………………….…………….. 52
C. Meet the Seeds …………………………………………...…………... 52
D. Give Higher Financial Incentives ……………………………………... 52
Appendix A: Lab Results ……………………………………………………. 54
Appendix B: Preparatory Phase ……………………………………………….. 55
Appendix C: Weekly Recruitment Schedule ………………………………… 59
Appendix D: Comparison between Actual and Desired Sample Sizes; Number of Seeds...69
Appendix E: Graph of Sample Distribution by Population …………………………. 70
Appendix E: Graph of Recruitment Schedule by Week ……………………………… 70
Appendix F: Main Reasons for Participation in Mishwar ……………………………... 71
References …………………………………………………………………………… 72

I. Introduction

A. Recent Statistics

In December 2007, the National AIDS Control Program (NAP), Ministry of Public Health, in Lebanon released the most recent HIV and AIDS statistics. Up to the year 2007, there were 1056 reported cases of people living with HIV and AIDS although the level of the epidemic is still as low as <0.1% of the general population. The real numbers however are believed to be much higher, as there are several factors that lead to underestimation of HIV and AIDS cases in Lebanon. The stigma associated with getting tested is one major cause. Another is the fact that although HIV/AIDS is a reportable disease in Lebanon and case reporting is required, it is not implemented fully by all physicians.

Surveillance data is needed in Lebanon, particularly bio-behavioral surveillance data for several reasons. Our complete reliance on case reporting, coupled with limited funds and resources to tackle the problem of HIV and AIDS, both justify that need. Of particular importance is collecting baseline data for existing populations in the Lebanese community, namely men who have sex with men (MSM), injecting drug users (IDUs), female sex workers (FSWs) and prisoners. At a low level of prevalence, such as the case in Lebanon, it is essential to have bio-behavioral data on the "most at risk populations" (MARPs). The stigma that these populations suffer from prevents them from seeking the services that they might highly need. This, in addition to the risky nature of their behavior, calls for immediate assessment and intervention.

B. Objectives of the Study

This study's main aim was to provide a baseline estimate of the prevalence of HIV and AIDS among the four vulnerable populations: MSM, FSWs, IDUs, and prisoners. The combination of conducting a behavioral questionnaire along with a biological test for HIV helped us to better understand the behavioral risk factors and to link the risky behavior to the HIV status of participants. Prevalence of co-infection of Hepatitis B (HBV) and Hepatitis

C (HCV) with HIV was also explored among all populations. HBV and HCV prevalence were estimated for the entire IDU population (and not just the HIV positives) due to lack of data on HCV cases in Lebanon and the high risk of infection among drug users.

C. Name of the Study

The study was named "Mishwar". The partner NGOs voted on the name. The word "Mishwar" in Arabic means a trip or journey. They chose that term because it is has a positive connotation of "accompanying" most at risk individuals in their journey of life. Furthermore, the term Mishwar sounds like another Arabic term "Mashoura", which means counseling and guidance (in relevance to VCT).

II. Methodology

A. Population Inclusion and Exclusion Criteria

The characteristics of the populations from which we sampled are summarized in Table 1 which lists the inclusion criteria and Table 2 which lists the exclusion criteria:

Table 1: Inclusion Criteria

	MSM	Prisoners	FSW	IDU
Lebanese, Registered Palestinian, Foreign Residents	X	X	X	X
Gender	M	M	F	M+F
Lower Age limit	16 years	16 years	16 years	16 years
Frequency of engaging in risk Behavior	Last 12 months	All current prisoners (regardless of frequency of engaging in behavior inside the prison)	Last 12 months	Last 12 months
Knows HIV status	Yes + No	Yes + No	Yes + No	Yes + No

Geographic Location	Greater Beirut and Jounieh	All current adult prisoners in Roumyeh Prison and juveniles* in Roumyeh's correctional facility	Greater Beirut and Jounieh	Greater Beirut and Jounieh

* Juveniles: 16-17 years old

Table 2: Exclusion criteria

MSM	Prisoners	CSW	IDU
MSM who do not comprehend English or Arabic language	Prisoners who do not comprehend English or Arabic language<br><br>Prisoners in the psychiatry unit<br><br>Violent prisoners<br><br>Mentally unstable prisoners in regular units<br><br>Political prisoners	CSWs who do not comprehend English or Arabic language	Users who do not comprehend English or Arabic language<br><br>Users who are not in a lucid state of mind to give informed consent during the time of the interview

B. *Partnering with NGOs*

Lebanon is known to have a very active and powerful civil society; many NGOs work in the field of public health. Due to the sensitive nature of the study and of the targeted populations, the AUB research team decided to partner with NGOs for Mishwar. The NGOs that were chosen are well established and trusted by members of the MARPs with whom they have contact. In addition to deepening their knowledge of the populations they serve, Mishwar was a very good opportunity for the NGOs to build their capacities and improve their skills in research.

The partner NGOs were as follows:

Population	NGOs
**MSM**	**Helem*; SIDC***
**IDUs**	**Skoun; Oum El Nour; SIDC**
**FSWs**	**Dar Al Amal**
**Prisoners**	**AJEM***

*SIDC: Soins Infirmiers et Development Communautaire; HELEM: Himaya Lubnaniya Lil Methleeyeen; AJEM: Association de Justice et Misericorde.

C. *Sampling*

Two types of sampling were utilized for the study: simple random sampling for the prisoners, and respondent driven sampling (RDS) for the rest of the MARPs.

i) Simple Random Sampling

The sampling methodology that was used to sample prisoners in Roumyeh Prison Facility was simple random sampling. Participants were randomly sampled from the constantly updated list of prisoners in each cell. The lists were updated as new inmates were introduced overnight in some cells.

ii) Respondent Driven Sampling (RDS)

MSM, IDUs, and FSWs are considered hidden populations in Lebanon because of the illegal nature of their activities and the stigma associated with their lifestyles. RDS, a chain referral system, has shown to be a very effective sampling methodology for such hidden populations for which there is no sampling frame[1]. RDS is especially designed to avoid many of the biases and problems of other chain referral systems, such as snowballing.

Sampling began with "seeds" that were selected on a non-random basis to ensure that the

[1] Heckathorn, D.D. (1997). Respondent-driven sampling: a new approach to the study of hidden populations. *Social Problems, 44, 174-199*

research team included seeds with specific characteristics of the target population. Seed selection took place from the population of the beneficiaries of the partner NGOs. In order to diversify the seeds, when necessary, the team also selected active and motivated individuals from the target groups, but not in contact with the NGOs, to serve as seeds for the study. The sampling then proceeded in "waves" whereby the first wave included those referred by the seeds, wave "2" were those recruited by the first-wave participants and so on. Each seed was expected to generate at least four waves and to recruit only three peers which in turn would recruit a max of three other peers. In order for waves to propagate continuously, seeds were added progressively throughout the data collection period when needed. The study began with 8 seeds in most populations, and then, the team progressively started feeding seeds. With FSWs, a total of 15 seeds were used, whereas 17 seeds were used with MSM. With IDUs (with two NGOs involved), a total of 27 seeds were used. (See Appendix D).

D. Sample Size

Sample sizes were calculated to allow monitoring of behavioral and prevalence changes over time. Below is a table comparing the desired sample size to the actual sample size that was achieved and the number of seeds used in each group, as well as the sample used for RDS analysis..

Testing differences in prevalence and behavioral practices over time will be done with 80% power and a confidence level of 95%. The sample size required to perform these tests is based on the following formula:

$$n = \frac{D\left[Z_{.95}\sqrt{2\overline{P}(1-\overline{P})} + Z_{.80}\sqrt{P_1(1-P_1)+P_2(1-P_2)}\right]^2}{\left(P_2 - P_1\right)^2}$$

where $Z_{.95}$ and $Z_{.80}$ are the 95th and 80th percentiles of the standard normal distribution, P_1 and P_2 are the estimated prevalence at time 1 and time 2 respectively, $\overline{P}$ is the average of P_1 and P_2, and D is the design effect (usually 2 for RDS sampling).

Population	Desired Sample Size	Actual Sample Size (total number participants)	Actual as % of Desired Sample Size	Number of Seeds	Sample Used for RDS
**MSM**	**620**	**120**	**19%**	**17**	**101**
**IDUs**	**388**	**109**	**28%**	**27**	**109**
**FSWs**	**880**	**150**	**17%**	**15**	**107**
**Prisoners**	**600**	**608**	**101%**	**N/A**	**N/A**
**Total**	**2488**	**987**	**40%**		**317**

Lebanon is a country with very low HIV prevalence. When the power calculations were done, they yielded extremely large sample sizes. After the end of the 12 month period of data collection, due to time and financial constraints and slowed recruitment, the team was not able to further extend data collection so as to capture the desired sample size. Difficulties in recruitment are explained in detail in a later section of this report. Furthermore, although the actual sample sizes are below the projected numbers, they still allow us to form an idea about the prevalence of risky behavior among MARPs in Lebanon.

E. The survey

The research team drafted four different questionnaires, tailored to each of the MARPs. For drafting the questionnaires, the team relied on relevant IBBS manuals and questionnaires previously conducted around the world. NGOs pilot tested the questionnaires with members of their populations who gave their feedback on the content and the terminology used. The sections include demographics, sexual history, drug use, imprisonment, body tattoos, knowledge about HIV and attitude towards HIV and cultural stigma. The questionnaires were translated from English to Arabic and then back-translated to English to check for accuracy of meanings and content. The surveys were available in English and colloquial Arabic and administered face-to-face by an interviewee from the NGOs.

F. ***The process of IBBS***

Potential participants came to the NGO center or called the interviewer to agree on a place to meet outside. The participant's coupon was checked for validity. Checking coupon expiration dates took place only during the beginning of the study, but when recruitment proved to be slow, coupon expiration dates were ignored. The interviewer introduced the study and read the consent form to the participant. The participant was then asked to repeat the main points in the consent form to make sure that he/she was in a lucid state of mind. Following the verbal consent, the screening process took place. The participant had to answer questions depending on the population he/she belongs to in order to validate his/her membership to that group. Those questions were designed by the NGO staff after consultations with members of the populations. Determining eligibility was also crucial, and that was established by checking if all the eligibility factors apply to the potential participant. After the membership and eligibility of the participant were checked, the survey was administered.

Pretest counseling and post-test counseling based on the information in the questionnaires was followed, and the IEC material tailored to each specific group was given. If the participant consented to the blood tests, his/her finger was pricked and three drops of blood were extracted and put on filter paper to test for HIV and possibly for HBV and HCV. The interviewer answered participants' questions concerning the study and how and when to get the results. The exit interview was filled and the participant was given three coupons to recruit his/her peers. From an ethical perspective, the team did not think it was fair for anxious participants to wait for a period of two weeks before getting their HIV status results, so the participants were also offered rapid HIV tests. Participants who chose to do a rapid test were given the appropriate post-test counseling.

III. Progress of "Mishwar"

As explained previously, the study was launched on August 1st, 2007. Data collection ended on July 31st, 2008. The pace of recruitment during the phase of data collection was not consistent – being very slow at first, but picked up slightly with the effort of the NGOs outreach workers. Several factors contributed to increasing the pace of recruitment or slowing it down. The outreach workers were former members of the populations. They are NGO staff or volunteer individuals who helped in the recruitment process by providing information about the population and information about the study to the population. Not all the seeds that the NGOs chose were well connected or motivated to successfully recruit people to the study. Recruitment was especially slow around times of holidays and political unrest. During these fluctuations, AUB field coordinators were meeting with the NGO staff every other day and discussing solutions to the obstacles arising.

Given the slow recruitment, and evidence that participants were not returning to pick up their secondary incentives, the study team decided to increase the incentives at two points during the course of data collection. Increasing the primary and secondary financial incentives seemed to be very effective with FSWs, but not the other populations. For IDUs and MSM, the increase in recruitment was related not only to choosing very well connected seeds who had motivated peers, but also those whose networks were dense and elaborate. More detailed analysis of progress by MARP group and by NGO follows.

IV. Progress of "Mishwar" by MARP Group

A. *Prisoners*

The available literature on the risky behavior within Lebanese prisons is very limited. Earlier focus group discussions that NAP conducted with prisoners revealed that several risky behaviors take place inside the prison, including sharing sharp objects and needles[2]. In addition, many of the prisoners have not undergone HIV, HBV or HCV testing because

[2] Dabaghi, Lara and Abdallah, Ahmad M. The National AIDS Control Program: Rapid Situation Assessment on Drug Use and HIV/AIDS in Prison Setting. UNAIDS/UNODC, 2008.

sometimes the test kits were not available. Mishwar's objectives helped close the existing gap in knowledge regarding risky behaviors in prisons and the prevalence of HIV.

The prisoners' population is the population whose recruitment was the easiest because the sampling methodology used in the prison setting was simple random sampling. The desired sample size was 600 male prisoners, aged 16 and above. The team achieved a sample size of 608. The prisoners were given in-kind compensation for their participation in the study. The in-kind compensation consisted of a cotton t-shirt and a pair of shorts, whose purchase value was similar to the original value assigned to the primary incentive given to the participants from the other populations.

Nevertheless, despite the advantage in the simplicity of the sampling frame, there were many obstacles that the study faced in the prison. The sensitive nature of the questions, especially the sections relating to the risky behavior within the prison, may have induced socially desirable answers. This is a general limitation that we expected in all populations; however, it might be more prevalent in the prison because of the negative implications the answers might carry for different partners involved -- the AUB research team, the partner NGO, and the prisoners who were worried that prison authorities might review the questionnaires or the blood test results. Although the methodology was designed in a way to prevent tracing the questionnaires or the blood tests to the respondents, it was still an important concern for the team.

Several meetings preceded the launch of the study at the prison. The meetings included the prison authorities, the NGO AJEM, in addition to the AUB research team. During those meetings, the objectives of the study were explained, in addition to the sampling frame and methodology. Most importantly, however, the study team met with the police authorities to get a written agreement from them that would protect the prisoners' anonymity.

The study was launched on August 1, 2007. Data collection in Roumyeh Prison ended on February 29, 2008. The progress of the study within the prison was relatively steady and smooth without major complications. There were days when the security in the prison was tighter than usual due to some incident or conflict within the facility or in the country. During those times, the prison was inaccessible to the study interviewers; however, such events were rare.

Rapid tests were offered to all participants in the study, along with pretest counseling because of the difficulty of giving the results back to prisoners, especially because they cannot be traced. In order to maintain confidentiality, the original plan was as follows: participants who chose to do a rapid test and the result was positive were encouraged (but not forced) to attend the specific awareness sessions that were to be held in the prison, in collaboration with AJEM. During these awareness campaigns, their confirmatory HIV results were to be given out, in addition to HCV and HBV for the confirmed HIV positive cases. Appropriate post-test counseling was done in total privacy. The interviewers were extensively trained on counseling skills through attending a VCT training workshop organized by NAP and one-on-one training sessions by a special psychiatrist hired by the NAP. The team did not have to resort to that plan however, since there was only one HIV positive case. That participant had already been identified as HIV positive upon his entry to the prison and was already placed in the HIV ward even before the launch of the study.

B. IDUs

The study required recruiting 388 IDUs through the RDS methodology using the help of two NGOs, Oum El Nour and Skoun. Initially, SIDC, a third NGO, was involved. However, SIDC was having major difficulties in working with and recruiting IDUs and was successful in recruiting only two IDUs. SIDC was therefore released from any responsibility with recruitment of IDUs using the RDS methodology but, instead, was asked to do a mapping of the MARPs. The mapping was done in the event that the study team chose to use Time-Location sampling. SIDC mapped gathering places of IDU, MSM,

and FSWs in Greater Beirut and Jounieh area[3]. SIDC also took over giving out the results and doing post-test counseling for Oum El Nour's participants' who consented to giving three dry blood spots for HIV, HBV and HCV testing. Oum El Nour expressed its reluctance to do voluntary testing and counseling (VCT) and did not offer VCT services throughout the study. Participants who wished to have a rapid test were referred to SIDC.

In Lebanon, IDUs have very closed networks and shooting galleries are limited. These factors led to a difficult and slow recruitment process. The repeated police raids and activities related to apprehending drug users and dealers also slowed down the progress of the study with IDUs. There is very little published literature on IDUs in Lebanon, so the team mostly relied on a qualitative study of IVDUs and FSWs that two former public health students had prepared as part of their graduation requirements[4]. The study included information on characteristics of IDU sub-groups and where the drug use usually takes place. Other than the extensive knowledge of the population that the local NGOs, the team also used the report to help in the identification of the characteristics of seeds needed in terms of demographics, SES, and shooting behavior.

Recruitment of IDUs started on August 1, 2007 and ended on July 31, 2008. The team achieved a sample size of 109. The seeds were chosen by the NGOs themselves after the desired profiles were drafted in collaboration with the AUB team. Seeds were chosen according to different socioeconomic status and educational backgrounds. Also, their shooting behavior differed: some inject drugs in groups, some in pairs, others alone. The NGOs were unsuccessful in recruiting a female IDU as a seed.

At the suggestion of the study team, Oum El Nour NGO used a volunteer outreach worker who acted as an additional source of information for the study. He helped in recruiting

---

[3] Soins Iinfirmiers et Developpement Communautaire. (2008). Mapping for FSWs and IDUs Report. Beirut, Lebanon.

[4] Daher C. and Zayat D. (2007). A Matter of Life: Views, Perceptions, and Practices of Commercial Sex Workers and Intravenous Drug Users Regarding HIV/AIDS Risk Behaviors. AUB-FHS.

seeds for Mishwar from his acquaintances. This individual is a former drug user and is very well trusted by IDUs. Upon recruitment, peers contacted this outreach worker for further assurances and details about the study, and sometimes they asked him to accompany them to the NGO center. The outreach worker played a very important role in following up the RDS methodology and the recruitment process. As mentioned earlier, the outreach workers provided information about the population, and helped promote and explain the study to potential participants and answer their questions. Two months later, Skoun, also sought the help of an outreach worker to recruit participants into the study. Using the NGO outreach workers helped improve recruitment as indicated in Chart 2 in Appendix E.

Reasons for the initial slow recruitment are numerous, many of which are related to the nature of IDUs. IDUs are perceived by the NGOs as forgetful and very hard to trust or be trusted. For this reason, using an outreach worker improved the process of recruitment. These individuals' job was to make sure to emphasize to the participants that the coupons need to be distributed and to promote, within the IDU population, the importance of the study and its benefits.

The second aspect that discouraged IDUs from participating in the study and coming to the NGO center to do the interview was that both Skoun and Oum El Nour are rehabilitation centers. IDUs did not want contact with a rehab center as they perceived that this would cause pressure for them to quit using drugs. For this reason, the team agreed to conduct the study outside the NGO center. Skoun's outreach worker offered his apartment as a venue for conducting the research, and Oum El Nour unsuccessfully tried connecting with several community centers to conduct the interviews there.

Few IDUs redeemed their secondary incentive. Also, few IDUs came back to get their HIV, HBV, or HCV results. This is fairly alarming since 51% of IDUs tested positive for HCV. Furthermore, only one IDU was found to be HIV positive, but this person was a seed who

did not know their HIV sero-status before the study. Among those recruited by RDS, no HIV cases were identified, so the overall HIV population prevalence rate among IDUs is estimated to be 0%.

C. MSM

Recruitment of MSM group members to Mishwar was a difficult task. The desired sample size of MSM was 620 individuals. Helem and SIDC were the two NGOs that were responsible for recruiting the sample. SIDC only managed to recruit five MSMs into the study and later on withdrew from the data collection process.

In Lebanon, many activists are calling for openness towards the issue of freedom of sexual identity, Helem being such a movement. There are several venues, cafes, and restaurants that MSM frequent. However, despite all those factors, many MSM still feel uncomfortable disclosing their sexual identity, due to societal and religious constraints and hence do not seek the services of Helem nor of any other NGO catering to the MSM population. Therefore, in order to include these samples in the study, the AUB study team hired a mobile interviewer. This mobile interviewer was able to recruit new and different seeds than those in contact with Helem. The question "Have you participated in this study before?" was asked in the screening process to avoid duplication. This trained interviewer conducted the questionnaire with the subpopulation which is not in contact with Helem, thus increasing the study's coverage scope of the population.

Helem targeted its own population for potential seeds. The seeds differed in SES and educational level. Also, some of the seeds work in Hammamat (Turkish baths), and are also involved in sex work. Clients of Hammamat were also recruited as seeds.

It is worth noting that the perceptions as to whether an HIV test acted as an incentive to participate in the study differed among the different participants. Many of the participants, in particular the ones who saw the mobile interviewer for the study, were primarily

motivated to participate by the HIV rapid test. They were eager to know their HIV status and that was incentive enough for them to participate in the study. However, according to the Manager of Helem, many of the participants who were being recruited by Helem were highly discouraged to enroll because of the stigma within their own networks associated with being tested for HIV. Upon discussing the issue with Helem staff and Dr. Mokhbat, the President of the Lebanese AIDS Society, both emphasized the importance of the social network for MSM and how getting tested for HIV could easily ruin social relations among MSM. In the exit interview, the participants were asked about their reason for participating in the study, to which the majorty of MSM who actually participated answered that the HIV test was the primary reason, followed by peer influence. The monetary incentive was not a highly significant factor for the MSM, as Helem staff told us most of the participants donated the money they got paid as incentives to the NGO itself. (Refer to Appendix F for Table on reasons for participation in Mishwar)

Of the recruited 120 participants, 101 underwent the DBS for HIV. Of those, only one tested positive for HIV. He was properly counseled and followed up by the NGO and NAP. Adjusting for RDS, the estimated population prevalence rate among MSM is 3.6% (see tables below).

D. FSWs

The population of female sex workers was the hardest to recruit to the study. The sample calculation required the recruitment of 880 eligible members into the study, all to be recruited through one NGO: Dar Al Amal. Although the recruitment process was slow towards the beginning, it picked up rapidly in May, since the staff at Dar Al Amal recruited a well-connected seed who works in hotels, and the interviews were carried out in those hotels- instead of the NGO center, which some FSWs were reluctant to visit.

Several factors explain why recruitment in the FSWs category was very difficult. The high level of competition between the SWs themselves affects the nature of social interaction

between the members (a similar experience was noted from the RDS study by Family Health International in Egypt). FSWs do not interact frequently and do not attempt to expand their social circles. This slowed down the propagation of RDS in this group.

Another reason why recruitment was very difficult is that not all FSWs go to Dar Al Amal center for services. This is especially true for high class escorts. Dar Al Amal staff resorted to seeds working in hotels and private apartments to recruit high class FSWs as seeds in the study. The rest of the seeds included FSWs from different nationalities and backgrounds, and were mostly beneficiaries of Dar Al Amal.

The use of an outreach worker was employed to enhance recruitment for this group. This outreach worker was a former SW who knows the population well. She made sure that coupons were being properly distributed and recruited FSWs contacted her in case they needed further information regarding the study. During the month of November, the staff at Dar Al Amal contacted two pimps who could help in introducing other potential seeds and recruit more participants into the study; unfortunately, the pimps were not collaborative. Several months later, Dar Al Amal was able to recruit a well connected seed that provided access to a dense network of SWs who work in hotels. The interviews with these participants were done outside Dar Al Amal premises.

The HIV rapid test seemed to be a motivating incentive to participate in the study for the FSWs. Peer influence also seemed to be an important factor in this study. Raising the value of the incentive coincided with the introduction of a very well connected seed caused a sudden surge of recruitment. NGO staff claim that increasing the incentive was pivotal in the progress of the study (see Appendix F)

Of the 150 FSWs recruited, 107 underwent DBS for HIV. Of those, no cases of HIV were identified among the FSW, for an overall sample prevalence rate of 0%.

IV. Progress of "Mishwar" at the NGO Level

A. AJEM

The IBBS study taking place in the prison followed the same guidelines as the other NGOs, with the only difference being the sampling methodology. In the prison, the team chose simple random sampling based on updated prisoners list. The participants were recruited from the four different buildings in the prison. The only prisoners not eligible for the study were the juveniles under 16 years of age for ethical reasons. Also, accessing political prisoners is prohibited, so they were excluded from the study. The study team also decided to exclude prisoners in the psychiatry unit since the quality of the information they would provide may be compromised. Finally, prisoners who did not comprehend English or Arabic languages were also excluded from the study.

The sample of participants was divided among the four buildings in the prison facility. Daily updated coded lists of the inmates were given to the NGO staff to sample from randomly, since inmates were sometimes introduced into the cells overnight. The prisoners were brought out to a private room in the building's common hall where the questionnaire and blood tests were done.

As in the other settings, a rapid test was offered on the spot because once the interview was done and the dry blood spots were taken, it was not feasible to trace the participant to give him the results. Participants who chose to do a rapid test and the result was unconfirmed were encouraged (but not forced) to attend the specific awareness sessions that were held in the prison, in collaboration with AJEM. During these awareness campaigns, their confirmatory HIV results were given out, in addition to HCV and HBV for the confirmed HIV positive cases. Appropriate post-test counseling was done in total privacy. Relevant and tailored IEC material was handed out to participants. The prisoner has to present his questionnaire's ID number and his mother's name at the awareness sessions to verify his identity and obtain his respective result. No HIV positive cases were reported, except for one participant who was sampled from the HIV positive ward of the prison.

To ensure proper counseling, a special counselor hired by NAP met with AJEM staff that was responsible for conducting the study inside the prison and had a one-on-one training session on counseling. This method was applied to all partner NGOs. Access to the prison was interrupted on few occasions due to security problems. During the month of December, the study progress was slower than usual due to the holiday season. The work in the prison was finished by the end of February, 2008.

B. *Oum El Nour*

Oum El Nour was one of the NGOs that started out on a steady pace but then the progress of the study at their center started declining. Oum El Nour was the first NGO that used one of its volunteers to work as an outreach worker. But even this aspect of the study faced some problems. The area that the outreach worker was targeting to obtain potential seeds was made inaccessible by a dominant political party. The outreach volunteer was constantly harassed, especially when the community discovered that he was affiliated to an opposing political force. He decided to relocate to other areas to contact potential IDU seeds, however that was not easy as it took much more time to build trust with those communities. In an attempt to enhance recruitment, Oum El Nour staff tried contacting an IDU rehabilitation center in Beirut area to try to refer seeds to the study; however, that attempt failed. With the progress of the study, there was a high turnover of outreach workers. The first worker fell ill and the second worker did not prove to be efficient in increasing recruitment. That factor slowed down the process. When the AUB research team asked the NGO staff and the seeds on what they think the problems might be, they summarized them as follows:

- The forgetful nature of IDUs
- The remote location of Oum El Nour
- The low financial incentive and compensation
- The taboo surrounding HIV and drug use
- The fact that the NGO is a rehabilitation center
- Intermittent police activity and raids

Another problem emerged when Oum El Nour refused to act as a VCT center and refused to do the post-test counseling for participants. This is due to a previous incident a few years back when one IDU who was in an incoherent state of mind attacked the NGO staff. Persistent stigma surrounding HIV also discouraged this NGO from conducting counseling at its site. Therefore, SIDC became the NGO doing the post-test counseling for the participants who gave three dry blood spots, and also did the rapid tests instead of Oum El Nour.

C. Skoun

Skoun was another of the NGOs assigned the task of recruiting IDUs to the study. Skoun is an outpatient clinic and does not do outreach activities, and so, unlike Oum El Nour, launching the study and finding seeds was very hard for the staff at first. In addition to that obstacle, the staff of Skoun experienced high staff turnover in the months of August to October due to the unfortunate political and social instability that Lebanon was going through. This delayed the progress of the study because the AUB research team had to retrain the new staff on the study. The constant change in staff also might have discouraged some participants from recruiting new people to the study because the follow up process was not assigned to a fixed, but rather continually changing staff. AUB tried to mediate a joined effort between Skoun and Oum El Nour to refer seeds to the former, but the plan failed due to the competitive nature of NGOs in Lebanon.

Towards the end of October, an active new staff member working on the study contacted an ex-user in Dahyeh area (an area in the Southern Suburbs of Beirut) who works as a volunteer in Skoun and used his services as an outreach worker. That was when the recruitment picked up. Skoun's outreach worker was also changed once, but only for a short period of time, then the former outreach worker resumed his job.

In this NGO too, as with other NGOs, some of the interviews were being conducted outside the center, at the level of participant's homes or the outreach worker's apartment. It was

clarified however, to Skoun and the other NGOs, that when conducting the study outside the center, they did so at their own convenience and risk.

The recruitment rate in Skoun was highly affected by the political instability of the country, since the areas where many of the individuals from the networks concerned were based were the same disenfranchised areas that were witnessing conflict and instability.

Two areas of concern were discussed among the AUB research team. First was the safety of the interviewers who were conducting the interviews outside the NGO centers. This applied to Skoun and all other NGOs as well. Another concern was Skoun's initiative of giving away free clean needles with the primary incentive upon completion of the questionnaire. Giving away free clean needles in Lebanon is still perceived negatively, and it was crucial for the research team at AUB to highlight to Skoun's staff the importance of explaining to the participants that the distribution of the clean needles was a Skoun initiative and not related to the study in any way.

D. Helem

Helem is an NGO that serves MSM in the Lebanese community. The staff includes motivated individuals who are eager to make a change in the society. They are well known by the MSM community. However, here too the team faced the same problem also faced with other NGOs, namely that not all MSM like to go to Helem's center or be associated with its activities. For this reason, the research team decided to hire a mobile interviewer. One of the seeds informed the team that the eligibility criteria made it hard to recruit some MSM because many were actually aware of the risk of HIV infection in relation to their behavior. For that reason they did not and had not practiced unprotected anal sex unless they were in a long term monogamous relationship with a single partner. This slowed down recruitment because this sub-group's HIV risk perception was low and they did not see any added benefit to them out of participating in the study. The research team believes

that the premise that, while it might be true, should not be generalized and is based on personal observation rather than any wider evidence.

Several techniques were adopted by Helem to try and increase the recruitment rate for the study. The research team thought of contacting Dr. Mokhbat, who is an infectious disease physician, the president and founder of the Lebanese AIDS Society and who treats HIV patients, to try and recruit seeds from his patient pool. The negotiations did not yield a positive result, because while the majority of his patients are infected with sexually transmitted diseases and HIV, they are not necessarily members of the vulnerable groups under study. Dr. Mokhbat also believed that with the context of the small and tight networks the MSM have, an RDS study focusing on HIV could be difficult. One successful recommendation was to have some seeds recruited in the *Hammamat*, or the Turkish baths in Beirut. These are places where MSM usually go. There is also the presence of male SWs. Some of the clients and the staff working in those H*ammamat* refused to go to the NGO center, so a big portion of the interviews were held in the field.

During the data collection phase, Helem launched and greatly marketed their VCT services. This was another project funded by the World Bank and supervised by NAP. This affected recruitment negatively as the VCT services were competing effectively with Mishwar over participants, in that a free rapid HIV test was offered. Individuals who wished to get tested found it much easier to get tested as a VCT beneficiary rather than answer a lengthy questionnaire before getting tested. The team was also facing some administrative problems with Helem. Despite their dedicated staff and their positive attitude, one of the individuals working on Mishwar lacked the rigor needed for RDS. Upon meeting with Helem's director, that individual was replaced on the study.

E. Dar Al Amal

As mentioned previously, Dar Al Amal is the NGO that works with FSWs. The recruitment was slowest at that NGO center. One of the main reasons was the competitive nature of the

FSWs and their networks. The degree of communication between different circles of FSWs was always kept at a minimum, which slowed down recruitment drastically. Also, police activity was on the rise during the months of October and November, and the FSWs were at risk of being apprehended. The option of using the help of an outreach worker was given to the NGO, but the outreach worker did not continue working throughout the data collection phase. As noted above, upon further discussions, the NGO staff contacted two pimps who could help in the identification of seeds but, unfortunately, these ultimately decided to withdraw and refused to help in recruitment.

After identifying a seed who works in hotels and is of high SES, the staff of the Dar Al Amal conducted some of the interviews, mostly with high class FSWs, at the different hotels and bars in which the SWs worked; this because some of the SWs did not want to visit the NGO center. They also established contact with FSW seeds who work in low class motels and apartments. Some of the interviews were being conducted outside the center, others within the NGO center. During the month of October, the center was overloaded with other projects and was undergoing renovation. This slowed the progress of the study as well. The FSW population was proving to be the hardest to recruit, until a very well connected seed was enrolled in the study. Her network was connected to another FSW network which facilitated recruitment.

None of the sample of FSWs tested positive for HIV. It is worth noting that the FSWs that were recruited from hotels and certain bars get tested regularly for HIV, and would be deprived from their work permit as "artists" if they were found to be HIV positive. "Moreover, in Lebanon, for foreigners to get a residence permit, they must undergo an HIV test."] In view of the fact that a high proportion of the sample of FSWs were foreigners resident in Lebanon, these factors might bias our sample.

F. SIDC

SIDC is an active NGO that serves IDUs and MSM. It is known for its outreach programs as well, which makes it exceptional in Lebanon. The partnership with SIDC was supposed to boost recruitment rates of MSM and IVDUs. This did not turn out to be the case however. SIDC was overloaded with other projects and had a shortage of staff and internal managerial problems. SIDC also expressed skepticism about the study's methodology. Its level of commitment declined gradually. Several meetings were held with SIDC's General Manager; however these meetings were not successful at keeping a high level of motivation for the study.

Recruitment was initially slow and then it stopped completely. Some of the reasons that were given to justify the slow pace were that the seeds were leaving the study without prior notice even before recruiting any new individual to the study; another was the increased frequency of police activity against MSM and IDUs.

Subsequently, SIDC took over the counseling for Oum El Nour. SIDC was also assigned the task of conducting a mapping of locations of the vulnerable populations (MSM, IDUs, and FSWs) to be used in future studies, as discussed above.

V. Main Findings and Discussion

Analyses of Prisoners' data

N=608 (consent missing for 8 subjects)

The age range of prisoners was 17-70 with a mean of 31.5 years and a standard deviation of 9.8 years. The majority were Lebanese (65.3%) and single (57.9%). There was only one positive (.16%) case of HIV (that person was selected from the cell where all HIV positive cases are assigned). That person was negative for both hepatitis B and C.

As for risky behaviors while still outside the prison, a quarter of the prisoners said that they had sex while drunk and three-quarters of those admitted to not using a condom during their last sex act. Moreover, 12% of the prisoners did inject drugs and a third of those had shared needle while doing so. In addition, around 8% had engaged in anal sex with a man.

When asked about risky behaviors while in the prison, the numbers dropped dramatically. Only one prisoner reported having sex while drunk and that he did not use a condom. Also, only one prisoner reported injecting drugs and stated that he did not share the needle with anyone else. On the other hand, only 2.6% had engaged in anal sex with a man and that prevalence dropped to 1.3% when asking about anal sex in the past month; however; what is alarming is that all anal sex was performed without using condoms.

As for knowledge, almost all prisoners (97.5%) have heard about HIV but only a third of them (29.1%) have been ever tested for it.

A quarter of the prisoners perceived themselves at a risk of HIV because they exchanged sex partners (22.8%), did not always use condoms during sex (55.0%), injected drugs (8.1%) or engaged in anal sex (1.3%). As for the other three quarters who perceived themselves at not being at risk of HIV, their reasons were that they had clean partner (24.4%), always used condoms (21.9%), did not engage in anal sex (0.4%), had sex with wife only (21.7%), always took precautions (4.5%) or were not sexually active (11.2%).

Prisoners' knowledge on prevention methods was high on the following: sharing needles (89.8%), using condoms during vaginal sex (86.1%), having one partner (88.3%), and using condoms during anal sex (76.4%). However, a large proportion of the prisoners thought that mosquitoes (64%) and using public toilets (42.6%) pose risks of transmission of HIV.

Finally, only one third of the prisoners had received information on HIV in the last year.

Variable	n (%)
Age group<br>17-19<br>20-29<br>30-39<br>40-49<br>50-59<br>60-70	 36 (5.9%)<br>281 (46.2%)<br>172 (28.3%)<br>82 (13.5%)<br>31 (5.1%)<br>6 (1.0%)
Nationality<br>Lebanese<br>Non-Lebanese	 397 (65.3%)<br>211 (34.7%)
Marital Status<br>Single<br>Married<br>Divorced<br>Separated<br>Widowed	 352 (57.9%)<br>210 (34.5%)<br>35 (5.8%)<br>2 (0.3%)<br>9 (1.5%)
HIV positive	1 (0.16%)

Risky Behaviors	Before entering the prison	While in Prison
Had sex while drunk	151 (24.8%)	1 (0.16%)
Did not Used a condom during the last sex act while drunk	115/151 (76.7)	1/1 (100%)
Ever injecting	73 (12.0%)	1 (0.16%)
Ever shared needle while injecting drug	25/73 (34.2%)	0/1 (0%)
Ever had anal sex with a man	51 (8.4%)	16 (2.6%)
Anal sex with man in last month	--------	8 (1.3%)
Did not use condom in anal sex last month	--------	8/8 (100%)

Knowledge	n(%)
Heard about HIV	593 (97.5%)
Ever tested for HIV	175 (29.1%)
Perceive oneself at risk for HIV	149 (24.5%)
Reasons for perceiving oneself at risk of HIV	
Often changes sex partner	34/149 (22.8%)
Do not always use condoms	82/149 (55.0%)
Use injecting drugs	12/149 (8.1%)
Anal sex	2/149 (1.3%)
Reasons for not perceiving oneself at risk of HIV	
Partner is clean	109/447 (24.4%)
Always uses condoms	98/447 (21.9%)
Does not have anal sex	2/447 (0.4%)
Sex only with wife	97/447 (21.7%)
Take precautions	20/447 (4.5%)
Not sexually active	50/447 (11.2%)
Received information on HIV in the last year	177 (29.2%)
Can not tell that someone have HIV by just looking at them	365 (62.5%)
Having sex with only one partner reduces the risk of HIV	514 (88.3%)
Can not get infected with HIV from using public toilet seats	334 (57.4%)
Using condoms during vaginal sex reduces prevent HIV transmission	502 (86.1%)
Misqitos do not transmit HIV	210 (36.0%)
Sharing needles while injecting drugs increases risk of HIV	522 (89.8%)

Using condoms during anal sex prevents HIV transmission	446 (76.4%)

Analysis of Female Sex Worker (FSW)

N = 107

No cases of HIV were identified in the sample of FSWs, for an overall sample prevalence rate of 0%.

The majority of the sample of SWs was ever-married, at 55%.

Less than a fifth (18%) of the sample was found to be Lebanese citizens, although all reside in Lebanon.

Concerning risk behaviors, there are several worrying findings. Firstly, over half of the sample (57%) reported a relatively low age at first sexual intercourse, being between the ages of 11 and 18. Secondly, the overwhelming majority of the sample (97%) had had more than 5 clients within the last month.

Reported condom use, however, was generally high, with 100% of respondents reporting they used a condom during the last time they had anal sex with a client, 98% the last time they had sexual intercourse with a non-regular client and 94% the last time they had sexual intercourse with a regular client. An exception to this trend was for the last time they had sexual intercourse with a regular client, when condom use drops to 43%.

A clear majority (79%) of FSWs had been tested for HIV, and 88% of these had been tested within the last year and 99% of these had obtained their HIV results.

Figures given in the table below indicated the prevalence of correct knowledge about each of the questions. Knowledge about HIV was highest about the HIV risks associated sharing needs for injecting drugs (91%), using condoms during vaginal sex (88%) and having only

sexual partner (77%). Two-thirds of respondents knew that one cannot tell if someone has HIV by looking at them and slightly less than that knew that using condoms during anal sex prevents HIV transmission. Lower figures (under 50%) were obtained about whether mosquitoes can transmit HIV (47%) and whether one can get HIV from using public toilet seats (35%).

Female Sex Workers – Selected Results of RDS Survey

Variable	Prevalence Adjusted for RDS P% (95% CI)
Ever married	55% (42%, 67%)
Lebanese citizenship	18% (8%, 30%)
Age at first sexual intercourse	
11-18	57%
19-25	43%
How many clients did you have sex with in the last month?	
<5	3%
≥5	97%
HIV positive	0%
Ever had anal sex with a client	3% (1%, 6%)
Used condom during the last time she had anal sex with a client.	100%
Used a condom in the Last time you had sexual intercourse with a non regular Client.	98% (96%, 100%)
Used a condom in the Last time you had sexual intercourse with a regular partner (non-Client).	94% (92%, 99%)
Used a condom in the Last time you had sexual intercourse with a regular Client.	43% (9%, 80%)
Ever tested for HIV	79% (69%, 89%)
When was the last time you did the HIV test	
Within last year	88% (80%, 96%)
1-2 years ago	5% (1%, 11%)
3-5 years ago	1% (0%, 3%)
More than 5 years ago	5% (1%, 11%)
Got their HIV test results	99% (98%, 100%)

Knowledge questions	Prevalence
Can not tell that someone have HIV by just looking at them	66% (57%, 76%)
Having sex with only one partner reduces the risk of HIV	77% (65%, 89%)
Can not get infected with HIV from using public toilet seats	35% (26%, 44%)
Using condoms during vaginal sex reduces prevent HIV transmission	88% (81%, 94%)
Mosquitoes do not transmit HIV	47% (36%, 57%)
Sharing needles while injecting drugs increases risk of HIV	91% (83%, 96%)
Using condoms during anal sex prevents HIV transmission	64% (54%, 74%)

Analysis of Injecting Drug Users

N = 109

As shown in the table below, a preliminary analysis was conducted of socio-demographic, risk behaviors and HIV, Hepatitis B and C prevalence for the sample of IDUs recruited through respondent-driven sampling (RDS) in Lebanon.

One IDU was found to be HIV-positive, but this person was a seed who did not know their HIV serostatus before the study. Among those recruited by RDS no HIV cases were identified, so the overall HIV population prevalence rate among IDUs is estimated to be 0%.

There were 56 cases of Hepatitis C identified, for an overall Hepatitis C sample prevalence of 51% but adjusting for RDS the population prevalence rate is estimated to be 49%.

Three Hepatitis B cases were identified, for an overall Hepatitis B sample prevalence of 2.8% but adjusting for RDS the population prevalence rate is estimated to be 6%.

Less than a third of the sample reported being ever-married (31%), with 8% having been divorced, 3% separated and 3% widowed.

The overwhelming majority of the IDU sample consisted of Lebanese nationals (93%).

Less than a fifth of IDUs (17%) reported sharing needles during their last injection.

More than two-thirds of the sample reported a young age of sexual initiation, being between 12 and 17.

Almost half of IDUs reported having bought sex and 17% reported having sold sex.

About a third of the sample (31%) reported having no regular non-commercial female sex partners, while two-thirds had less than 5 such partners and only 3% had 5 or more such partners.

Less than half of the sample (43%) reported using a condom during the last sexual intercourse with a non-commercial regular sex partner.

Figures on knowledge about HIV given in the table below indicated the prevalence of correct knowledge about each of the questions. A very high percentage of the sample (97%) reported knowing that sharing needles while injecting drugs increases the risk of HIV transmission. Similarly, knowledge about the lower risk associated with having only one partner, using condoms during vaginal sex and during anal sex were high. Less than half of the sample reported knowing that one cannot get HIV from using public toilets (43%) and that mosquitoes do not transmit HIV (30%).

Analysis of IVDU data – Selected Results from RDS Survey

**Variable**	**Prevalence Adjusted for RDS P% (95% CI)**
Ever married	31% (14%, 52%)
Marital Status	
Single	69% (45%, 86%)
Married	18% (7%, 36%)
Divorced	8% (0.4%, 23%)
Separated	3% (0.2%, 7%)
Widowed	3% (0%, 8%)
Lebanese citizenship	93% (85%, 99%)
HIV positive	0%
HBV positive	6% (0%, 8%)
HCV positive	49% (29%, 70%)
Sharing needle during last injection	17% (8%, 27%)
Age at first sexual intercourse	
12-17	68%
18-27	32%
Bought sex	47.1% (31%, 66%)
Sold Sex	17% (3%, 30%)
Number of Regular non commercial female sex partner	
0	31% (18%, 47%)
<5	66% (50%, 80%)
5 or more	3% (0%, 7%)
Used condom with in the last sex act with non commercial regular sex partner	43% (20%, 68%)

Analysis of the MSM data – RDS study

Total Sample size = 120

Number of seeds = 17

Number of subjects that did not enter the analysis because they are missing coupon =2

Final sample size used for RDS = 101

Only 5 % of the sample of MSMs were ever-married, with 3% currently married and 2% separated. Less than three-quarters of the sample were Lebanon (73%).

One person in the sample was found to be HIV-positive, and adjusting for RDS the population prevalence rate is estimated to be 3.6%.

Over half of the sample had had anal sexual intercourse before their 18th birthday and more than a third (36%) had sold anal sex. About two-thirds (62%) of the sample reported having no noncommercial sex partners, but over a third reported up to but less than five non-commercial sex partners.

Worryingly, more than half of the sample reported not using a condom during their last anal sex with a non-commercial sex partner. However, two thirds of the sample reported using a condom during their last anal sex with a non-commercial occasional sex partner

The majority of the sample (61%) reported having up to but less than 5 non-commercial occasional sex partners but 39% reported having more than 5.

Less than a quarter of the sample (24%) reported having had an HIV test, and of these 46% reported having the test in the last year and 55% in the last two years. All those who had a test reported receiving their results.

Knowledge about HIV was relatively high (all scores but one in the 90% and 80%) with the highest scores (indicating correct knowledge) about whether one can tell a person with HIV by looking at them and the risk of sharing needs in injecting drugs. The lowest score (70%), however, was obtained for having only one sexual partner reduces the risk of HIV.

Analysis of the MSM data – RDS study

Variable	Prevalence RDS
Ever Married	5% (1%, 11%)
Current Marital Status	
Single	95% (88%, 99%)
Married	3% (0%, 9%)
Separated	2% (0%, 5%)
Lebanese	73% (50%, 90%)
HIV positive	3.6% (0%, 6%)
Age at first anal sexual intercourse	
<18	54% (39%, 63%)
18 or more	46% (37%, 61%)
Sold Anal Sex	36% (18%, 56%)
Number of non-commercial sex partners	
0	62% (44%, 81%)
<5	37% (18%, 56%)
5 or more	1% (0%, 2.5%)
Condom use during last anal sex with non commercial sex partner	
Yes	47% *
No	53% *
Number of non-commercial occasional sex partners	
<5	61% (34%, 78%)
5 or more	39% (22%, 66%)
Condom use during last anal sex with non commercial occasional sex partner	
Yes	66% (46%, 80%)
No	34% (20%, 54%)

* Numbers are too small to compute a confidence interval

HIV

Variable	Prevalence (95% CI)
Ever Tested for HIV	24% (11%, 40%)
Test date<br>Within last year<br>1-2 years ago<br>More than 2 years ago	 36% (4%, 53%)<br>55% (8%, 91%)<br>9% (0%, 61%)
Got their test results	100%

Knowledge

Correct Knowledge	Prevalence (95%)
Can not tell that someone have HIV by just looking at them	93% (86%, 98%)
Having sex with only one partner reduces the risk of HIV	70% (56%, 83%)
Can not get infected with HIV from using public toilet seats	88% (75%, 96%)
Using condoms during vaginal sex reduces prevent HIV transmission	83% (70%, 92%)
Mosquitoes do not transmit HIV	85%, (75%, 93%)
Sharing needles while injecting drugs increases risk of HIV	89% (72%, 99%)
Using condoms during anal sex prevents HIV transmission	88% (78%, 97%)

**Knowledge questions**	**Prevalence (RDS)**
Can not tell that someone have HIV by just looking at them	78% (64%, 90%)
Having sex with only one partner reduces the risk of HIV	88% (74%, 98%)
Can not get infected with HIV from using public toilet seats	43% (27%, 59%)
Using condoms during vaginal sex reduces prevent HIV transmission	86% (77%, 94%)
Misqitos do not transmit HIV	30% (17%, 43%)
Sharing needles while injecting drugs increases risk of HIV	97% (96%, 99.7%)
Using condoms during anal sex prevents HIV transmission	84% (75%, 91%)

VI. Major Obstacles Facing "Mishwar"

This section discusses some of the obstacles that were faced with all the NGOs

A. *Structural and Sociocultural Factors*

- ✓ *Taboo and stigma associated with HIV/AIDS*: The taboo nature of HIV and AIDS is a major obstacle that was faced in this study. This context of taboo creates stigma against individuals, thus causing them to be hesitant about getting tested or participating in the study. In an attempt to address this issue, the research team decided to stop presenting the study as an HIV and AIDS study, but rather as a study on sexual behavior, and emphasized that getting tested for HIV was optional. One incident that only highlights the stigma associated with HIV is the case of an NGO involved in this study that refused to do HIV counseling to someone who tested HIV positive, despite the intensive training in ethics and counseling that its staff had received at the beginning of the study.

- ✓ *Low risk perception:* The risk perception in the general population is relatively low. In fact, according to a 2004 KAP study done in Lebanon, 67.5% of the respondents perceived no chance of contracting HIV[5]. This problem has not been tackled by the AUB research team for this particular study, however, there continues to be awareness campaigns related to HIV and AIDS, HBV, and HCV. Such campaigns are held at the national level, or are the result of the efforts and initiatives of numerous active NGOs in the Lebanese society.

- ✓ *Tight and closed networks*: The networks formed by the vulnerable populations are described as small and tightly-knit. An individual's social relations may be greatly sabotaged if his or her peers discover that this individual is getting tested for HIV or

[5] National AIDS Control Programme in Lebanon (2004) Knowledge, Attitude, Beliefs and Practices of the Lebanese Population Concerning HIV/AIDS. Ministry of Public Health and WHO. Beirut, Lebanon.

suspects that person of being HIV positive. This is especially true in the case of MSM. This was confirmed to the team by the discussions with Dr. Jacques Mokhbat (who treats many AIDS patients in Lebanon and is the President of the Lebanese AIDS Society). Emphasizing that HIV testing was optional and maintaining anonymity and confidentiality helped tackle this problem with some degree of success.

- ✓ *Political instability*: The political instability that Lebanon has been suffering from during the course of data collection slowed down the project markedly. The general political insecurity made people limit their movement within the city. NGOs located in areas that are remote from Greater Beirut were not frequently visited by the participants because of the dangers of bombs or explosions on highways and roads. Also, the disenfranchised areas where many of the NGOs networks of potential participants are based are the same areas that were witnessing most of the conflict and struggle.

B. RDS Technical Difficulties

- ✓ *Locating seeds for the study*: The problem of finding seeds for the study was an obstacle that the team faced. The seeds that the team was seeking needed to fit a certain character profile so as to be able to fulfill their tasks as required. Several steps were taken to introduce motivated and well-connected seeds. For different reasons, there were times when the seeds relocated without informing the NGOs. In one case, a seed selected by an NGO relocated to Syria without the knowledge of the NGO. Seeds who were not well connected or motivated to get involved in the study, or those who relocated away from Beirut, did not produce extended waves. Some NGOs contacted community centers to seek help in referring and recruiting seeds to the study. The highly competitive nature of the NGOs in Lebanon, especially the partner NGOs, prevented collaboration in the area of seed referral among each other. At times, personal acquaintances of the research team who were eligible for the study were referred as seeds. The AUB team gave each NGO (except AJEM in the prisons) the option of choosing a staff member to act as an outreach worker. This person needed to

be well known and trusted by the community. She/he would act as an additional source of information for the study and would suggest seeds to the study team and closely monitor the recruitment process.

- ✓ *Slow recruitment*: The recruitment process of the study went through very slow periods. To help find out the reason(s) for this, the AUB research team met with the seeds of the study. Potential scenarios of why recruitment was slow were explored. One of the identified problems was the very low incentive; another was that the recruiters were not emphasizing the importance of the study to their peers. After nine months of data collection, the NGOs launched VCT services at their centers. This also compromised the level of recruitment in Mishwar. The holidays and the month of Ramadan caused the progress to slow down at times.

- ✓ *Low financial incentive*: Discussions with the seeds and the NGO staff indicated that the financial incentive offered for recruitment was low. The team intentionally chose to give low incentive in order not to induce coercion. Also, the team had a limited sum of money to allocated incentives among the originally large calculated sample size. This did not allow for higher incentives. In Mishwar, we noticed that participants usually did not come back to claim their secondary incentive. In October 2007, the financial incentive for recruitment was increased from $2 to 3.5$ per recruitment, and later in March 2008 the primary incentive was raised to $10 and the secondary incentive to $6.7 per recruitment. As explained earlier, the increase of the financial incentive produced a boost in recruiting FSWs, but for MSM, the money was not as significant, as most of the MSM were donating the money back to Helem.

- ✓ *Launching NGO VCT services:* During the progress of the study, Helem launched their VCT services. This further slowed down recruitment as individuals preferred the VCT to RDS because the process was much easier for them than to participate in a study and recruit peers, especially since the incentive was low.

C. NGO Limitations

- ✓ *Decreasing level of NGO commitment*: The RDS process requires a highly motivated and fully dedicated staff to carry out the study. The NGOs that AUB partnered with for this study are understaffed and overloaded with work and projects. Their level of commitment and the time they gave for the study was not as high as the AUB team had expected. The AUB team, realizing the importance of keeping the NGOs motivated for the study, held meetings with them on a regular basis. During these meetings, potential problems were identified and addressed. The AUB study coordinators visited the NGOs every other day to pick up the filled questionnaires, perform quality control checks on the forms, and discuss any problems or emerging obstacles.

- ✓ *NGOs' limited outreach activities*: One of the main difficulties that the team faced in the study is that the majority of the partner NGOs does not do extensive outreach activities. For instance, both NGOs serving the IDU population focus their efforts more on rehabilitation than on outreach. This factor had the effect of limiting the size of the population that the NGOs serve, their knowledge of the population and their ability to suggest seeds.

- ✓ *Stigma of being associated with the NGOs*: Many individuals did not seek NGO services and do not wish to be seen there so as not to be associated with that NGO nor their vulnerable population. One NGO that the team partnered with to recruit IDUs is a rehabilitation institution. Many participants refused to go to the center to conduct the interview because they were afraid they might be coerced to join rehab services. Another factor that prevented some participants from going to the NGO centers was that the opening hours were not always suitable. The research team decided to deal with these obstacles by adopting two techniques. First, it was agreed that the interviewer and the potential participant had the freedom to meet at a place of mutual

convenience outside the NGO center. Also, the team hired a trained mobile interviewer who was willing to meet and conduct the interviews with the participants at flexible hours and locations.

- ✓ *Weak counseling skills*: Not all the NGOs had a staff that was well qualified to carry out HIV pretest and post-test counseling. For that reason, an extensive four day workshop took place with certified counselors from NAP and SIDC, explaining the principles of counseling prior to launching the study. Afterwards, a certified psychologist/counselor was hired by the National AIDS Program to visit the NGOs and carry out one-to-one training sessions on counseling. The sessions were supervised by an AUB research team member.

D. Biological Testing Difficulties

- ✓ *HCV test kits cost efficiency*: The kits for HCV that use dry blood spots are very expensive. That is why weekly or bi-weekly runs of the test were not feasible. For that purpose, a schedule that specifies the dates when HBV and HCV results would be issued was drafted and distributed to the NGOs.

- ✓ *Waiting time for HIV confirmatory tests*: The AUB laboratories had initially promised to deliver the HIV confirmatory runs every other week based on a higher expected recruitment rate. Those were even delayed longer due to the slow recruitment process. From an ethical perspective, concerns were raised, as the AUB research team discussed the anxiety of an unconfirmed case and the participant's burden of waiting for two weeks until a confirmatory result was issued. Hence, a set schedule for the confirmatory runs, and the HBV and HCV tests was drafted. Also, rapid HIV tests were offered to all participants. The unconfirmed cases were sent to contracted laboratories that issued the result within three waiting days. The rapid tests were provided only to the participants who consented to the DBS.

VII. Steps Taken to Increase Recruitment

The progress of the study was not as easy as anticipated. Mishwar's study team meet weekly to discuss the emerging obstacles and to think of strategies to increase the recruitment process. The following steps were taken to increase recruitment. It is worth noting that in addition to the steps described below, the team held a series of meetings with the seeds and participants to try and anticipate problems and obstacles to overcome them.

A. Increasing the Financial Incentive

The AUB research team, during the preparatory phase for the study, decided on an incentive level of $6.7 for the primary incentive, and $2 per recruitment for the secondary incentive. The team had two main concerns in mind when discussing the issue of incentives. The first concern was the issue of giving IDUs money and how they might use that money to buy drugs. A literature review showed that IDUs, like any other population, do not necessarily use the money to buy drugs. Money may be used to meet the general needs of IDUS[6]. That, in addition to the respect for an IDU's rights and sense of autonomy, the research team agreed to give the monetary incentives to members of that population. Coercion was the other issue to consider for all MARPs. The team did not put a high value incentive so that low-income individuals have free and informed choice as to whether to participate in the study and were not influenced unduly or coerced by the level of payment.

Upon meeting with the NGO staff members and with the seeds, the team noticed that the issue of low incentive was a major factor that was slowing the recruitment process. In October, the secondary incentive was raised from $2 to $3.5. That was not sufficient however, especially with the increasing public transportation and living costs. Towards March, the primary incentive was increased to $10, and the secondary incentive to $6.7 per recruitment.

---

[6] Semaan S, Santibanez S, Garfein RS, Heckathorn DD, Des Jarlais DC. Ethical and regulatory considerations in HIV prevention studies employing respondent-driven sampling. *Int J Drug Policy*. 2008. doi:10.1016/j.drugpo.2007.12.006.

B. *Engaging the NGO Outreach Workers*

The NGO outreach workers' involvement in the project was very efficient. These outreach workers were either NGO staff or NGO volunteers, and were known and trusted by the individuals who used the NGOs and their services. However, it was left to the NGO to decide whether or not they wanted to employ an outreach worker to promote the study among the respective populations (Helem did not use an outreach worker). The main concerns when engaging an outreach worker were coercion and the breaching of confidentiality. Coercion was avoided because the recruited participants were the ones contacting the outreach workers, and not the opposite. Furthermore, the outreach workers were not paid based on the recruitment rates. As for confidentiality of participants, it was maintained since the outreach workers were members of the NGO research team.

C. *Engaging a Mobile Interviewer*

When using the mobile the interviewer, the team was able to increase the catchment area because it was able to access the population that did not use the NGO services. The mobile interviewer was employed with the MSM population because their recruitment rate was the slowest and Helem was the only NGO working with that population.

D. *Encouraging Conducting Interviews Outside NGO Centers*

As discussed previously, many individuals were reluctant to go to the NGO centers to participate in the study. For that reason, the team agreed to the option of conducting the interviews at a place of mutual consent outside the NGO center.

E. *Drafting a Schedule for Lab Results*

For reasons of cost-efficiency when recruitment proved to be slow, it was not practical to conduct the HIV, HCV, or HBV tests weekly. For that reason, a schedule that specifies the dates for the HIV, HCV, and HBV runs was drafted and distributed to the NGOs. This increased the credibility of the study for both the NGOs and the population.

VIII. Conclusion and Lessons Learned

On July 31, 2008, the phase of data collection ended, having stretched over 12 months. Knowing that the study was the first IBBS study in Lebanon to be conducted using RDS as a sampling methodology, Mishwar was a great learning opportunity for the team and NGOs. It was an eye opener to the little available information on Lebanon's MARPs. The literature that the team relied on pertained to other developing countries whose context may vary greatly from the Lebanese context. However, it is always important to keep in mind that this study established base-line data for surveillance that was previously nonexistent. Based on the team's experience in Mishwar, we make the following recommendations:

A. Conduct Formative Research

Conducting formative research is a crucial step that needs to be done prior to implementing an RDS sampling methodology. The assumption in the Lebanese case was that the NGOs already knew the targeted populations. However, that was not entirely true. The density and dynamics of the networks were unknown to both the research team and the NGOs. Also, the composition of the population was neither very clear to the study team nor to the NGOs. This is why the team had difficulties in anticipating the rate of the recruitment and selecting appropriate seeds. This knowledge gap also led the research team and the NGO staff to make speculations about the populations that were not necessarily always true.

Formative research may consist of qualitative interviews with members of the populations. In-depth interviews, focus groups, and observations can give the team a clear idea as to what motivates the population, what are its concerns and potential recruitment within networks. Classes and hierarchies within populations and networks, in addition to characteristics of the MARP networks are depicted through formative research. Mapping activities also can help in understanding the geographical spread of the population and may aid in planning the logistics of the study.

B. Ensure a Full-time Dedicated Staff

RDS is a sampling process that requires a highly motivated staff working on full-time basis. Individuals must be highly qualified, with excellent communication skills. Due to the abundance of forms that need to be filled out in the course of one interview, there needs to be an interviewer and a quality control officer who check that all forms have been filled correctly. These two individuals need to be well organized, thorough, and rigorous. In our case, we were working with NGOs whose members and volunteers were already overloaded with projects.

C. Meet the Seeds

As explained before, the AUB research team partnered with different NGOs. The team believed that the populations did not trust the AUB team as much as they did the NGOs; hence the choice of seeds was left to them. The AUB research team did not meet those seeds prior to the study launch for confidentiality purposes. Meetings with the seeds took place during the course of data collection, when the team wanted to inquire about possible reasons for the slow recruitment. Arranging a meeting between the research team and the seeds before the study in order motivate them and explain to them the study and its benefits proved to be crucial.

D. Give Higher Financial Incentives

In Mishwar's experience, we assumed that the free HIV testing would be a main incentive for the research subjects to participate. However, increasing the incentive was probably a more effective way of speeding up recruitment. Lebanon's living costs have been increasing steadily, and the incentives should account for not only transportation costs, but also the time and effort the participant has to put into participating in the study. Unless the incentive is carefully calculated to make the participation efforts worthwhile, recruitment will remain low.

IX. CONCLUSION

In conclusion, Mishwar was a great learning opportunity for the NGOs and the study team alike. Although the desired sample sizes were not reached, the study team was able to produce baseline prevalence data for Lebanon's MARPs. Also, implementing the RDS methodology in Lebanon for the first time had its advantages. First, the team was able to identify potential obstacles that may arise in case another team decides to replicate the study, and went further to suggest possible solutions to these problems. Furthermore, thanks to RDS, some of the NGOs reached populations that we were not connected to them prior to the study. Another positive outcome that came out of the study is that the NGOs and the study team now have better knowledge of the MARPs in Lebanon, in terms of their geographical distribution and characteristics. Finally, the study succeeded in fostering the skills of the NGOs, especially their research and counseling skills.

Appendix A

Lab Results

Lab Results for HIV, Hep B, Hep C- Study Sample

Total Number of Samples with Dried Blood Spots (DBS):

Men Having Sex with Men (MSM)	**101**
Injecting Drug Users (IDUs)	**109**
Female Sex Workers (FSWs)	**107**
Prisoners	**580**
Total	**897**

HIV Positive Samples:

1 IDU
1 Prisoner
1 MSM
**Total: 3**

Hepatitis Sample Prevalence Rates

	Hepatitis C		Hepatitis B	
Group	Count	Percentage	Count	Percentage
IDUs	56 / 109	**51 %**	3 / 109	**2.8 %**

Appendix B

<u>Preparatory Phase</u>

The study was launched on August 1, 2007. The AUB research team visited the partner NGOs and provided them with the necessary forms, material, questionnaires, and the needed guidance to start the process of data collection. Preceding the launch however, a series of meetings, trainings, and workshops took place. These are described below.

A. Preliminary Meetings with NGO Managers

In the process of involving the NGOs in the course of the study, the AUB team members met frequently with the General Managers of the potential partner NGOs during the months of February and March 07. These meetings took place at the NAP office in Beirut. The purpose behind the meetings was to explain the objectives of the study and the benefits of participating in the study for the future in terms of capacity building and coordination between the different stakeholders working in the field of HIV/AIDS. Later on, the AUB team met with the NGOs managers and staff on individual basis for further discussions and explanations.

B. Proposal Approval by the Institutional Review Board (IRB)

One of the major steps that we had to establish was to get the IRB's approval for the study. Over the month of July 07, the AUB research team was discussing the ethical issues and other problems that may arise in the study and possible scenarios to deal with those issues. The team made sure that all ethical dilemmas were mentioned and adequately addressed in the protocol. The protocol was read and revisited by all team members before it was sent to the IRB for review. A few weeks later we were granted the approval. It should be mentioned that ethical issues and dilemmas arose continuously during the course of the study and the team deliberated over them intensively so as to minimize harm to participants from the MARPs and in a difficult political context.

C. Drafting Seeds Profile and Population Characteristics

Prior to launching the study, it was very important to establish the different characteristics of the population. That way, seeds can be selected to represent the different strata of the population. To be able to achieve that task, weekly meetings during the month of July 07 took place between the NGO staff working on the study and the AUB research team. The NGOs' knowledge of their population formed a base for drafting the seeds' profiles. For the FSW and IDU, the AUB research team also referred to a research project that AUB MPH students had conducted a few months before which included focus group discussions and in-depth interviews with members of these populations.

D. Conducting Trainings and Workshops

Six different training sessions and workshops were conducted at AUB between the months of June and July 07. The NGO representatives attending the meetings were chosen by the NGOs internally to carry out the study at the NGO sites.

During the first two encounters between the NGO teams and the AUB research team, an overall introduction of the study was given. The objectives of the study were explained in detail. Later, the questionnaires were reviewed. The NGOs gave their feedback regarding the content and the structure of the questionnaires. In addition, the NGOs provided us with the common colloquial terminology used by the populations, which was later incorporated into the questionnaires.

The following training session was on communication skills. Due to the sensitivity of the topics explored in the study, the AUB research team thought it was necessary to provide the staff of the NGOs with tips and guidelines for communicating appropriately and effectively with the participants.

The two training sessions that followed covered the RDS methodology. A scientific presentation was given, explaining the significance of the RDS methodology, how it works,

data collection flow, and why it is the sampling method of choice in this particular research. Later, the forms that were going to be used in the study were distributed, reviewed, and the participants were taught how to fill them out.

Another training session covered the finger pricking methodology for the HIV, HCV, and HBV tests. The staff was given all the equipment needed and they were given a live demonstration of finger pricking and later practiced the technique among each other.

An intensive workshop was given to tackle ethical issues and stigma that are bound to surface as the study proceeds. Case studies were given and discussed, highlighting the basic ethical principles of beneficence, autonomy and justice. The importance of the consent form was emphasized.

Finally, a four day workshop on Voluntary Counseling and Testing (VCT) was held. Trained counselors from SIDC and NAP addressed such topics as stigma involved with HIV and its effect on populations most at risk, principles of VCT, pre and post-test counseling, in addition to the referral system of medical services.

It is also worth mentioning that in addition to these training sessions that were done in groups, frequent individual trainings took place at the NGO centers to make sure that the tracking methodology of RDS and the questionnaires are fully understood. These extra training sessions were especially necessary given the high staff turnover that took place at some NGO centers during the months of August and September 07.

E. *Piloting the Questionnaires*

After the changes in the language and content of the questionnaires were made, each of the partner NGOs was given three questionnaires to pilot test. This provided additional insights on improving the questionnaires, which were provided in the English and colloquial Arabic language.

F. *Conducting Formative Research*

Conducting extensive formative research was not feasible due to many reasons. First, there is a total lack of published studies describing the networks of the target populations. Time constraints prevented the team from doing qualitative studies with members of the populations to better understand the networks. To make up for the gap, the research team decided to partner with the NGOs who have better knowledge of the characteristics of the populations. However, despite the very limited literature available on the vulnerable populations we are working with, and the complete lack of literature on RDS in Lebanon, ongoing reviews on international literature were conducted to help overcome many of the obstacles that we faced in the study. RDS methodology and its use internationally were intensively researched. The literature provided the AUB research team with alternatives in case needed.

G. *Holding Weekly Meetings*

Since the beginning of the study, the AUB research team has met weekly. During those meetings, the progress of the study was discussed, and arising problems were dealt with.

Appendix C

Weekly Recruitment Schedule

August/ September 2007

Name of the NGO/ Target Population	Week 1 (6- 12 Aug)	Week 2 (13-19Aug)	Week 3 (20-26Aug)	Week 4 (27 Aug-2 Sep)	Week 5 (3- 9 Sep)	Week 6 (10-16 Sep)
DAR AL AMAL		21<br>22<br>221<br>23<br>24		222<br>223		
**Total number of CSWs**	0	5	0	2	0	0
HELEM	31<br>32<br>33<br>34<br>35<br>36	37	311<br>331<br>332<br>333<br>362<br>361<br>38<br>381	3113<br>312		3111<br>3112
SIDC		11<br>111	1111<br>12	1113		
**Total number of MSM**	6	3	10	3	0	2
OUM El NOUR	41<br>411<br>412<br>42<br>43	413<br>44	421<br>422<br>4111	432<br>4121		4212<br>442
SKOUN	51<br>52		53			
SIDC						11
**Total number of IVDUs**	7	2	4	2	0	3
**Total number of participants**	13	10	14	7	0	5

September/ October 2007

Name of the NGO/ Population	Week 7 (17- 23 Sep)	Week 8 (24- 30 Sep)	Week 9 (1-7 Oct)	Week 10 (8-14 Oct)	Week 11 (15- 21 Oct)	Week 12 (22-28 Oct)
DAR AL AMAL						**25**<br>**26**<br>**27**
**Total number of CSWs**	**0**	**0**	**0**	**0**	**0**	**3**
HELEM						
SIDC						
**Total number of MSM**	**0**	**0**	**0**	**0**	**0**	**0**
OUM El NOUR			**42122**<br>**42123**			
SKOUN			**54**<br>**55**	**541**<br>**542**<br>**56**	**543**<br>**5413**<br>**54131**<br>**57**	**58**<br>**581**<br>**582**<br>**583**<br>**5831**
SIDC			**12**			
**Total number of IVDUs**	**0**	**0**	**5**	**3**	**4**	**5**
**Total number of participants**	**0**	**0**	**5**	**3**	**4**	**8**

October/ November/ December 2007

Name of the NGO/ Population	Week 13 (29 Oct- 4 Nov)	Week 14 (5- 11 Nov)	Week 15 (12- 18 Nov)	Week 16 (19-25 Nov)	Week 17 (26 Nov- 2 Dec)	Week 18 (3- 9 Dec)
DAR AL AMAL	28<br>281	251<br>2511	261<br>2613		252<br>2521<br>25211	25212<br>25213<br>252131<br>252132
**Total number of CSWs**	2	2	2	0	3	4
HELEM	39		310<br>3101<br>3102	391<br>3103<br>31011<br>31012		31013<br>31021<br>31022<br>31023<br>3911
SIDC						
**Total number of MSM**	1	0	3	4	0	5
OUM El NOUR		45<br>46	451<br>452<br>453	41113<br>421221<br>421222	4523	4522
SKOUN		5411<br>54113<br>5812*	5832<br>5833<br>58331<br>583313<br>583311		54111<br>541112	54112<br>541121<br>5411211
SIDC						
**Total number of IVDUs**	0	4	8	3	3	4
**Total number of participants**	3	6	13	7	6	13

* One Non Eligible candidate at Skoun (drug user but not injections)

December 2007

Name of the NGO/ Population	Week 19 (10- 16 Dec)	Week 20 (17- 23 Dec)	Week 21 (24 - 31Dec)			
DAR AL AMAL	252133<br>252121					
**Total number of CSWs**	**2**	**0**	**0**			
HELEM	3912					
Mobile Interviewer		71				
SIDC						
**Total number of MSM**	**1**	**1**	**0**			
OUM El NOUR	45222<br>461		41112<br>411123			
SKOUN	5411213<br>5411212		59			
SIDC						
**Total number of IVDUs**	**4**	**0**	**3**			
**Total number of participants**	**7**	**1**	**3**			

January/February 2008

Name of the NGO/ Target Population	Week 22 (1- 6 Jan)	Week 23 (7- 13 Jan)	Week 24 (14-20 Jan)	Week 25 (21 – 27 Jan)	Week 26 (28 Jan-3 Feb)	Week 27 (4-10 Feb)
DAR AL AMAL						
**Total number of CSWs**	**0**	**0**	**0**	**0**	**0**	**0**
HELEM			**311**<br>**3111**<br>**3112**<br>**3113**<br>**31112**<br>**31123**<br>**31121**	**31133**	**311331**	**311332**<br>**311333**
Mobile Interviewer		**711**	**712**<br>**713**		**7131**<br>**7113**	
SIDC						
**Total number of MSM**	**0**	**1**	**9**	**1**	**3**	**2**
OUM El NOUR			**4613**	**4111231**<br>**41112311**		
SKOUN	**510**<br>**511**				**512**<br>**54112111**	**5112**
SIDC						
**Total number of IVDUs**	**2**	**0**	**1**	**2**	**2**	**1**
**Total number of participants**	**2**	**1**	**10**	**3**	**5**	**3**

February/March 2008

**Name of the NGO/ Target Population**	**Week 28** (11-17 Feb)	**Week 29** (18- 24 Feb)	**Week 30** (25 Feb-2 Mar)	**Week 31** (3 – 9 Mar)	**Week 32** (10 - 16 Mar)	**Week 33** (17-23 Mar)
DAR AL AMAL	**29**<br>**291**<br>**292**<br>**293**<br>**2911**<br>**2913**<br>**2921**<br>**29211**<br>**292111**<br>**29212**	**29213**<br>**292112**		**292131**<br>**292133**<br>**2921331**<br>**292132**<br>**2921322**<br>**2921323**<br>**2921321**<br>**29213222**<br>**29213312**	**292121**<br>**2921213**<br>**292113**<br>**292122**	**210**<br>**2103**<br>**21031**<br>**2102**<br>**210313**<br>**21032**<br>**210311**<br>**210312**<br>**2103121**
**Total number of CSWs**	**10**	**2**	**0**	**9**	**4**	**9**
HELEM		**31123**<br>**311231**<br>**311232**<br>**31111**<br>**311122**	**3113323**			
Mobile Interviewer		**7132**<br>**7133**<br>**71333**	**71321**<br>**71322**<br>**713222**<br>**71323**	**713221**<br>**713223**<br>**7132231**<br>**7132233**<br>**72**<br>**71322213**	**73**<br>**7132211**	
SIDC						
**Total number of MSM**	**0**	**8**	**5**	**6**	**2**	**0**
OUM El NOUR		**463**				
SKOUN	**513**	**5121**<br>**5132**		**514**<br>**515**		
SIDC						
**Total number of IVDUs**	**1**	**3**	**0**	**2**	**0**	**0**
**Total number of participants**	**11**	**13**	**5**	**17**	**6**	**9**

March/April/May 2008

**Name of the NGO/ Target Population**	**Week 35** (24-30 Mar)	**Week 36** (31 Mar- 6 Apr)	**Week 37** (7-13 Apr)	**Week 38** (14-20 Apr)	**Week 39** (21-27 Apr)	**Week 40** (28 Apr- 4 May)
DAR AL AMAL		**2921221**<br>**29212212**<br>**292122121**<br>**292122122**<br>**292122123**<br>**2921221232**	**211**	**213**<br>**2131**<br>**2132**<br>**2133**<br>**21331**		**213333**<br>**213332**<br>**213331**<br>**2133323**<br>**2133322**<br>**2133321**
**Total number of CSWs**	**0**	**6**	**1**	**5**	**0**	**6**
HELEM						
Mobile Interviewer			**713233**<br>**721**<br>**7132332**			
SIDC						
**Total number of MSM**	**0**	**0**	**3**	**0**	**0**	**0**
OUM El NOUR						
SKOUN		**516**	**5151**<br>**5161**<br>**5162**<br>**5163**		**51622**<br>**51623**	
SIDC						
**Total number of IVDUs**	**0**	**1**	**4**	**0**	**2**	**0**
**Total number of participants**	**0**	**7**	**8**	**5**	**2**	**6**

May/June 2008

**Name of the NGO/ Target Population**	**Week 41** (5-11 May)	**Week 42** (12-18 May)	**Week 43** (19-25 May)	**Week 44** (26 May-1 June)	**Week 45** (2-8 June)	**Week 46** (9-15 June)
DAR AL AMAL	**21333211<br>2133311<br>2133312<br>21333221<br>21333212<br>2133313<br>21333223<br>21333222<br>21333233<br>21333213<br>21333231<br>21333232**			**213333<br>21333111<br>213332123**	**213332223<br>213332122<br>2133321221<br>213332212<br>21333121<br>21333123<br>21333122<br>21333121<br>213332211<br>213331213**	**213332232<br>213332311<br>2133321331<br>213332133<br>2133321332<br>2133323212<br>21333213321<br>213332313<br>213332312<br>213332321<br>213332323**
**Total number of CSWs**	**12**	**0**	**0**	**3**	**10**	**11**
HELEM						
SIDC						
**Total number of MSM**	**0**	**0**	**0**	**0**	**0**	**0**
OUM El NOUR		**47<br>471<br>473<br>4711<br>4712<br>48**	**4713**	**472<br>47131**	**47132<br>47133<br>4721<br>471323<br>471321**	
SKOUN		**517**	**541123<br>5411233<br>518<br>516233**			
SIDC						
**Total number of IVDUs**	**0**	**7**	**5**	**3**	**5**	**0**
**Total number of participants**	**0**	**7**	**8**	**6**	**15**	**11**

June/July 2008

Name of the NGO/ Target Population	Week 47 (16-22 June)	Week 48 (23-29 June)	Week 49 (30 June-6 July)	Week 50 (7-13 July)	Week 51 (14-20 July)	Week 52 (21-27 July)
DAR AL AMAL	**213332113<br>2133321133<br>213332221<br>2133322213<br>2133322211<br>2133322212<br>213332132**	**2133321323<br>2133321132<br>213332131<br>2133321311<br>213332222<br>213332121<br>2133321213<br>21333212133<br>21333132<br>21333212132<br>21333133<br>2133321212<br>2133321211<br>213332213<br>21333231<br>2133323312<br>2133323311<br>213332233**		**21333211331<br>21333211221<br>2133321133<br>21333222131<br>21333222132<br>21333212133<br>213332221331<br>213332221333<br>213332221332<br>213332121321**		
**Total number of CSWs**	**7**	**18**	**0**	**10**	**0**	**0**
HELEM	**312<br>3121<br>3122<br>3123<br>31211<br>31212<br>31213<br>31221<br>31222**	**312311<br>312312<br>312313<br>312121<br>312111<br>312112<br>312113**		**312322<br>312323<br>312321**	**312331<br>312332<br>312333**	**313<br>3131<br>3132<br>3133<br>31331<br>31332<br>31333**

	**31223**<br>**31231**<br>**31232**<br>**31233**					
SIDC						
**Total number of MSM**	**13**	**7**	**0**	**6**	**3**	**7**
OUM El NOUR	**4722**<br>**471322**<br>**47221**<br>**4723**	**471311**				
SKOUN						**519**
SIDC						
**Total number of IVDUs**	**4**	**1**	**0**	**0**	**0**	**1**
**Total number of participants**	**24**	**27**	**0**	**16**	**3**	**8**

Appendix D

Table 1: Comparative representation of the requested sample size and the actual numbers of participants recruited in Mishwar

Population	Desired Sample Size	Actual Sample Size (Survey Completed)	% of desired	Number of Blood Samples	# of seeds
MSM	620	120	19%	101	17
IDUs	388	109	28%	109	27
FSWs	880	150	17%	107	15
Prisoners	600	608	101%	580	
Total	2488	987	40%	897	

Appendix E

Chart 1: Distribution by Population

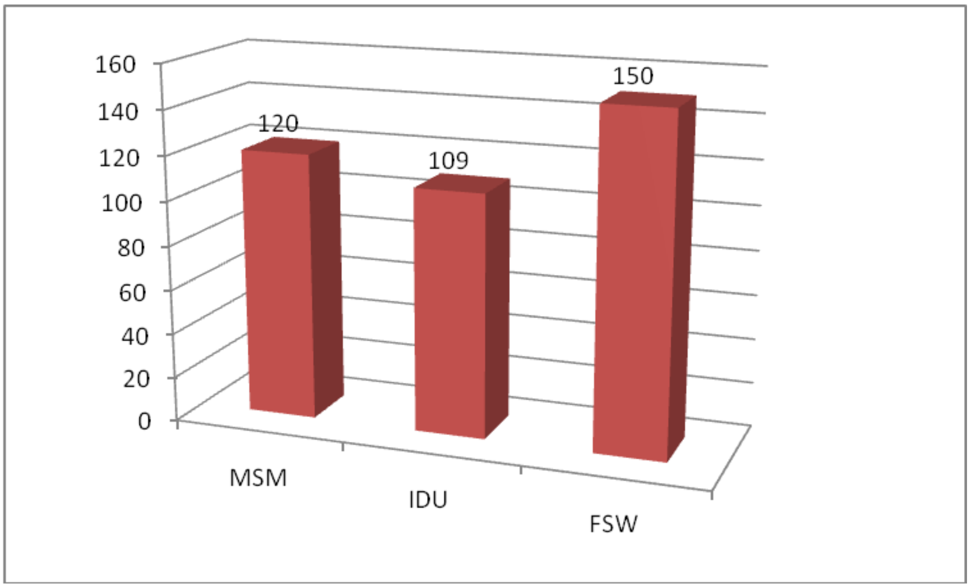

Chart 2: Distribution by Week

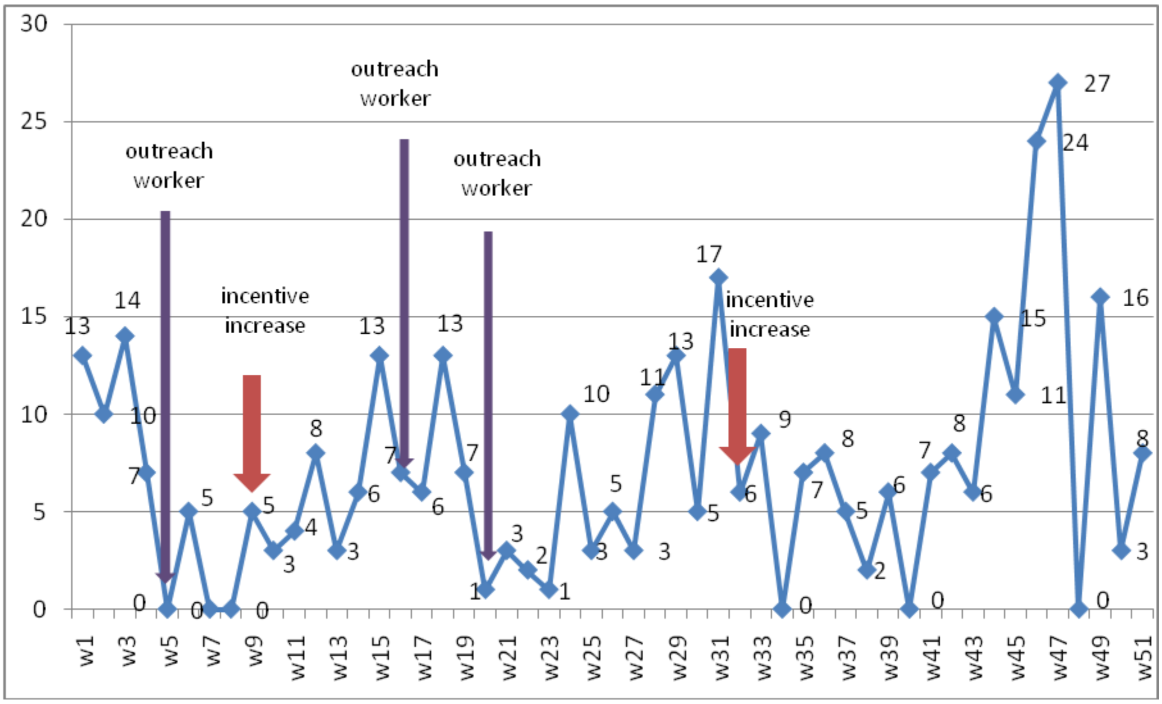

Appendix F

Table: Main Reason for Participation in Mishwar

Main reason for participation	Financial incentives	HIV test	Because of my friends	Study is interesting	Spare/free time	Other (treatment in IDU case)
MSM PRE INCENTIVE RAISE	4.8% (3/62)	40.3% (25/62)	22.6% (14/62)	32.3% (20/62)	0%	0%
MSM POST INCENTIVE RAISE	34.6% (18/52)	34.6% (18/52)	23.1% (12/52)	7.7% (4/52)	0%	0%
FSW PRE INCENTIVE RAISE	30.3% (10/33)	42.4% (14/33)	18.2% (6/33)	9.1% (3/33)	0%	0%
FSW POST INCENTIVE RAISE	4.5% (5/111)	48.6% (54/111)	45% (50/111)	1.8% (2/111)	0%	0%
IDU PRE INCENTIVE RAISE	13.9% (10/72)	52.8% (38/72)	1.4% (1/72)	16.7% (12/72)	0%	15.3% (11/72)
IDU POST INCENTIVE RAISE	38.2% (13/34)	38.2% (13/34)	5.9% (2/34)	2.9% (1/34)	2.9% (1/34)	11.8%(4/34)

References:

1. Heckathorn, D.D. (1997). Respondent-driven sampling: a new approach to the study of hidden populations. *Social Problems, 44, 174-199*
2. Dabaghi, L. and Abdallah, M.A. (2008). The National AIDS Control Program: Rapid Situation Assessment on Drug Use and HIV/AIDS in Prison Setting. UNAIDS/UNODC.

3. Soins Iinfirmiers et Developpement Communautaire. (2008). Maping for FSW and IDU Report. Beirut, Lebanon.

4. Daher C. and Zayat D. (2007). A Matter of Life: Views, Perceptions, and Practices of Commercial Sex Workers and Intravenous Drug Users Regarding HIV/AIDS Risk Behaviors. AUB-FHS.

5. National AIDS Control Programme in Lebanon. (2004). Knowledge, Attitude, Beliefs and Practices of the Lebanese Population Concerning HIV/AIDS. Ministry of Public Health and WHO. Beirut, Lebanon.

6. Semaan S., Santibanez S., Garfein R.S., Heckathorn D.D., Des Jarlais D.C. (2008). Ethical and regulatory considerations in HIV prevention studies employing respondent-driven sampling. *Int J Drug Policy*. doi:10.1016/j.drugpo.2007.12.006.
