# Supplementary material for: Progress towards the 95–95–95 targets to end HIV by 2030 in Lebanon, 2023
Source: PLoS One. 2025 Jun 13;20(6):e0321868. doi: 10.1371/journal.pone.0321868 (PMC12165419; doi:10.1371/journal.pone.0321868)
Supplement: S6 File — Lebanon HIV country fact sheet by UNAIDS 2022. (PDF) [file pone.0321868.s006.pdf]

Country factsheets

LEBANON | 2022

HIV and AIDS Estimates

**Adults and children living with HIV**	**2600 [2200 - 3100]**
Adults aged 15 and over living with HIV	2600 [2200 - 3100]
Women aged 15 and over living with HIV	<500 [<500 - <500]
Men aged 15 and over living with HIV	2400 [1900 - 2800]
Children aged 0 to 14 living with HIV	... [... - ...]
**Adult aged 15 to 49 HIV prevalence rate**	**<0.1 [<0.1 - 0.1]**
Women aged 15 to 49 HIV prevalence rate	<0.1 [<0.1 - <0.1]
Men aged 15 to 49 HIV prevalence rate	0.2 [0.1 - 0.2]
HIV prevalence among young women	<0.1 [<0.1 - <0.1]
HIV prevalence among young men	<0.1 [<0.1 - <0.1]
**Adults and children newly infected with HIV**	**... [... - ...]**
Adults aged 15 and over newly infected with HIV	... [... - ...]
Women aged 15 and over newly infected with HIV	... [... - ...]
Men aged 15 and over newly infected with HIV	... [... - ...]
Children aged 0 to 14 newly infected with HIV	... [... - ...]
**HIV incidence per 1000 population (adults 15-49)**	**... [... - ...]**
HIV incidence per 1000 population (all ages)	... [... - ...]
**Adult and child deaths due to AIDS**	**<100 [<100 - <100]**
Deaths due to AIDS among adults aged 15 and over	<100 [<100 - <100]
Deaths due to AIDS among women aged 15 and over	<100 [<100 - <100]

Deaths due to AIDS among men aged 15 and over	<100 [<100 - <100]
Deaths due to AIDS among children aged 0 to 14	... [... - ...]
**Orphans due to AIDS aged 0 to 17**	**<1000 [<1000 - 1100]**

Epidemic transition metrics

Percent change in new HIV infections since 2010	...
Percent change in AIDS-related deaths since 2010	-25
Incidence : prevalence ratio	...
Incidence : mortality ratio	...

HIV testing and treatment cascade

**People living with HIV**	**2600 [2200 - 3100]**
**People living with HIV who know their status**	**2300**
Percent of people living with HIV who know their status	86 [72 - >98]
**People living with HIV who are on ART**	**2100**
Percent of people living with HIV who are on ART	80 [67 - 94]
**People living with HIV who have suppressed viral loads**	**2000**
Percent of people living with HIV who have suppressed viral loads	76 [63 - 89]

Antiretroviral therapy (ART)

**Coverage of adults and children receiving ART (%)**	**80 [67 - 94]**
Adults aged 15 and over receiving ART	80 [67 - 95]

Women aged 15 and over receiving ART	67 [60 - 75]
Men aged 15 and over receiving ART	82 [67 - 98]
Children aged 0 to 14 receiving ART	... [... - ...]
**Number of adults and children receiving ART (#)**	**2109**
Adults aged 15 and over receiving ART	2105
Women aged 15 and over receiving ART	181
Men aged 15 and over receiving ART	1924
Children aged 0 to 14 receiving ART	4

Elimination of vertical transmission

**Coverage of pregnant women who receive ARV for PMTCT (%)**	**... [... - ...]**
Pregnant women who received ARV for PMTCT (#)	6
Pregnant women needing ARV for PMTCT (#)	... [... - ...]
**Final vertical transmission rate including during breastfeeding**	**... [... - ...]**
**New HIV infections averted due to PMTCT (%)**	**... [... - ...]**
Number of HIV-exposed children who are uninfected	... [... - ...]

Sex workers

Population size estimate (#)	4300	Region: National; Method: Unique object multiplier, Wisdom of crowd, Enumeration, and Literature review ; Source: IBBS 2018
HIV prevalence (%)		Source: Behavioral surveillance, 2018
Condom use (%)	79.2	Source: Behavioral surveillance, 2018

Men who have sex with men

Population size estimate (#)	16 500	Region: National; Method: Unique object multiplier, Wisdom of crowd, Enumeration, and Literature review ; Source: IBBS 2018
HIV prevalence (%)	12	Source: Behavioral surveillance, 2018
HIV testing and status awareness (%)	92.3	Source: Behavioral surveillance, 2018
Condom use (%)	52.2	Source: Behavioral Surveillance Survey

Combination prevention

People receiving pre-exposure prophylaxis (PrEP) (#)	192

HIV expenditure

**Total country-reported HIV expenditure (USD)**	**9 500 000**	**Source:GAM_2023**
HIV expenditure from domestic public sources (USD)		Source:GAM_2023
HIV expenditure from domestic private sources (USD)		Source:GAM_2023
HIV expenditure from international sources (USD)	9 500 000	Source:GAM_2023

Hepatitis and HIV

Proportion of people coinfected with HIV and HCV starting HCV treatment	100	Source: Clinical records of health-care facilities 2017
